# Synthesis, Reactions, and Agrochemical Studies of New 4,6-Diaryl-2-hydrazinylnicotinonitriles

**DOI:** 10.3390/ijms262411874

**Published:** 2025-12-09

**Authors:** Victor V. Dotsenko, Vladislav K. Kindop, Vyacheslav K. Kindop, Renat G. Achmiz, Arina G. Levchenko, Polina G. Dakhno, Azamat Z. Temerdashev, Yu-Qi Feng, Quan-Fei Zhu, Eva S. Daus, Igor V. Yudaev, Yuliia V. Daus, Alexander V. Aksenov, Nicolai A. Aksenov, Inna V. Aksenova

**Affiliations:** 1 Department of Organic Chemistry and Technologies, Kuban State University, 149 Stavropolskaya St., 350040 Krasnodar, Russia; vlad.kindop@mail.ru (V.K.K.); slavakindop@mail.ru (V.K.K.); renat989898@gmail.com (R.G.A.); levchenko.arin@yandex.ru (A.G.L.); p.dahno@yandex.ru (P.G.D.); eva.daus.2008@gmail.com (E.S.D.); 2 Department of Chemistry, North Caucasus Federal University, 1a Pushkin St., 355017 Stavropol, Russia; aaksenov@ncfu.ru (A.V.A.); radioanimation@rambler.ru (N.A.A.); inna-aksenova00@rambler.ru (I.V.A.); 3 Department of Analytical Chemistry, Kuban State University, 149 Stavropolskaya St., 350040 Krasnodar, Russia; temerdashevaz@gmail.com; 4 School of Bioengineering and Health, Wuhan Textile University, 1 Sunshine Avenue, Jiang Xia District, Wuhan 430200, China; yqfeng@whu.edu.cn (Y.-Q.F.); qf_zhu@whu.edu.cn (Q.-F.Z.); 5 Faculty of Energetics, Kuban State Agrarian University, 13 Kalinina St., 350044 Krasnodar, Russia; etsh1965@mail.ru (I.V.Y.); zirochka2505@gmail.com (Y.V.D.)

**Keywords:** malononitrile, unsaturated ketones, Michael adducts, 2-bromo-3-cyanopyridines, 2-hydrazinylpyridines, hydrazones, bromination, 2,4-D herbicide safeners

## Abstract

This work aimed to synthesize new derivatives of 2-hydrazinylpyridine-3-carbonitrile and to investigate their biological activity as safeners for the 2,4-D herbicide. The new 2-hydrazinylnicotinonitriles were obtained in high yields (up to quantitative) under mild conditions (25 °C, dioxane) by treating 4,6-diaryl-2-bromo-3-cyanopyridines with hydrazine hydrate. The latter were synthesized by brominating 2-(3-oxo-1,3-diarylpropyl)malononitriles, the Michael adducts, which are readily available from 1,3-diarylpropenones (chalcones) and malononitrile. An unusual side product of the bromination/carbocyclization was isolated and characterized; it consisted of co-crystals of 3-benzoyl-4-hydroxy-4-phenyl-2,6-di-(p-tolyl)cyclohexane-1,1-dicarbonitrile and 3-benzoyl-5-bromo-4-hydroxy-4-phenyl-2,6-di-(p-tolyl)cyclohexane-1,1-dicarbonitrile at a ~4:6 ratio. The new 2-hydrazinylnicotinonitriles react with halogen-containing aromatic aldehydes to form the corresponding hydrazones. The biological activity of the new nicotinonitriles was examined for their function as 2,4-D antidotes. It was found that, under laboratory conditions, eight of the synthesized compounds exhibited a notable antidote effect against 2,4-D on sunflower seedlings.

## 1. Introduction

**Figure 1:**
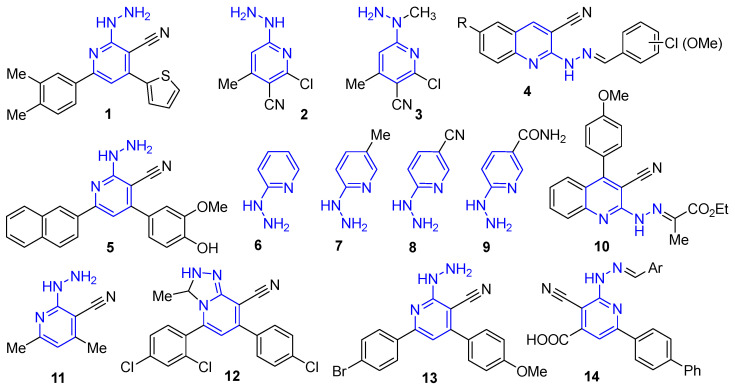
Biologically active and practically useful 2-hydrazinylpyridines.

2-Hydrazinylpyridines represent a promising class of compounds with diverse, though not yet fully realized, potential [1]. According to recent data, 2-hydrazinylpyridines **1** are of interest as antioxidants and antimicrobial agents [2]. Compounds **2** and **3** serve as intermediates in the synthesis of wheat growth regulators [3,4] (Figure 1). Hydrazones **4** exhibit antimicrobial action, with MIC values ranging from 12.5 to 25 μg/mL [5]. Nicotinonitrile **5** demonstrated significant activity against the breast cancer cell line MCF-7 in in vivo experiments [6]. The unsubstituted 2-hydrazinylpyridine **6** is used for inhibiting lysyl oxidase-2 (LOXL2) as an alternative to phenyl hydrazine [7,8], for preparing coordination compounds with various metals [9,10,11,12,13,14,15], and as an analytical reagent for derivatization in the determination of steroid hormones [16,17,18,19,20,21]. Recently, 2-hydrazinylpyridine **6** and 2-hydrazinyl-5-methylpyridine **7** were proposed as twin derivatization reagents for the rapid determination of α,β-unsaturated aldehydes in vegetable oils [22]. Hydrazines **6** and **7**, in combination with nicotinonitrile **8**, were recently proposed for identifying potential lung cancer carbonyl biomarkers in human plasma samples [23]. 6-Hydrazinylnicotinamide **9** is used to produce bifunctional coupling agents for ^99m^Tc labeling of small biomolecules [24,25]. In recent years, condensation products of 2-hydrazinylpyridine with carbonyl compounds have been proposed as chemosensors for detecting SO_4_^2−^ and CO_3_^2−^ [26], Cu^2+^ and Co^2+^ [27], Al^3+^ and OH^−^ [28], and Hg^2+^ and Au^3+^ [29,30,31], as viscosity-sensitive fluorescent probes [32], and as aggregation-induced emission dyes suitable for fluorescence imaging of cellular organelles [33]. Compound **10** showed 100% tumor inhibition, comparable to the standard drug Vincristine, and it also exhibited tremendous activity toward *Leishmania major* [34]. Cytotoxic activity has also been reported for 2-hydrazinylnicotinonitrile **11 [35]**, high fungicidal activity for compound **12** [36], and antimicrobial effects for nicotinonitrile **13** [37] and hydrazones **14** [38] (Figure 1). It is also worth mentioning that 2-hydrazinylpyridines and 2-hydrazinylnicotinonitriles are precursors to several practically important classes of heterocyclic compounds, such as 1,2,4-triazolo[4,3-*a*]pyridines [39,40,41,42,43,44], pyrazolo[3,4-*b*]pyridines [45,46,47,48,49,50,51,52,53,54,55], and pyrido-1,2,4-triazines [56].

Continuing our research in the chemistry of nicotinonitriles with agrochemical applications [57,58,59,60,61,62,63,64,65] on one hand, and the search for new derivatizing agents for UHPLC-HRMS determination of metabolites in biological fluids [66,67,68,69] on the other, we aimed to prepare new 4,6-diaryl-2-hydrazinylnicotinonitriles, study some of their reactions, and evaluate the biological activity of the products as antidotes for the herbicide 2,4-D.

**Figure 2:**
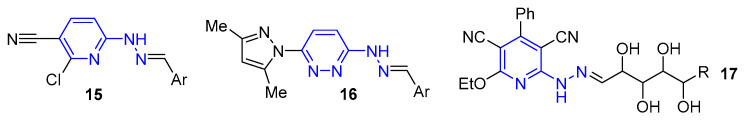
(2-Hetaryl)hydrazones with agrochemical activity.

Despite their potential, 4,6-diaryl-2-hydrazinylnicotinonitriles have not yet found application in analytical chemistry or agrochemistry. These compounds are expected to be more selective derivatizing agents for carbonyl compounds than the currently used 2-hydrazinylpyridine derivatives [16,17,18,19,20,21,22,23]. Support for their potential in agrochemistry comes from reports on analogous structures. Some related compounds **15** are known herbicide safeners and rice growth stimulants [70,71], others, like compounds **16,** are growth regulators for winter wheat [72], and hydrazones **17** exhibit antifungal activity against *Aspergillus flavus* and *A. niger* [73] (Figure 2). Nevertheless, 4,6-diaryl-2-hydrazinylnicotinonitriles remain a scarcely studied group of compounds, and their practical application has not been reported in the literature to date.

**Scheme 1:**
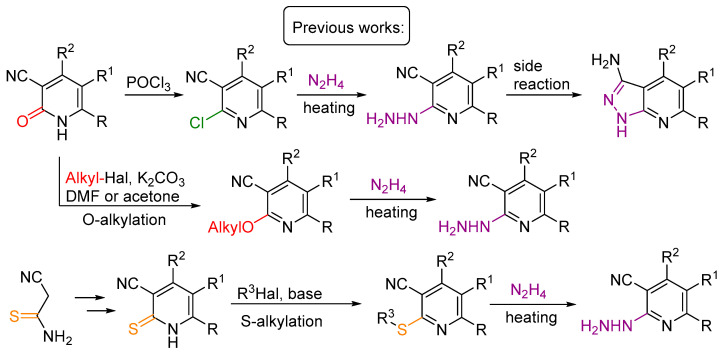
The reported synthetic approaches to 2-hydrazinylnicotinonitriles.

The primary methods for the synthesis of 2-hydrazinylnicotinonitrile derivatives are based on the hydrazinolysis of 2-chloro-3-cyanopyridines [6,34,35,36,37,38,74,75,76,77,78,79,80,81,82,83,84,85,86,87,88,89,90,91,92] or 2-alkoxy-3-cyanopyridines [73,92,93,94,95,96]. An alternative route involves the nucleophilic displacement of a sulfur-containing group in 2-mercaptonicotinonitrile derivatives [5,92,97,98,99,100] (Scheme 1). It is important to note certain limitations associated with these established synthetic pathways. A significant drawback is the multi-step nature of the synthesis for 2-hydrazinyl-3-cyanopyridines. The required starting 2-alkoxy-, 2-chloro-, or 2-alkylthio nicotinonitrile precursors are themselves predominantly obtained from starting materials that already contain the pre-formed nicotinonitrile core structure.

**Scheme 2:**
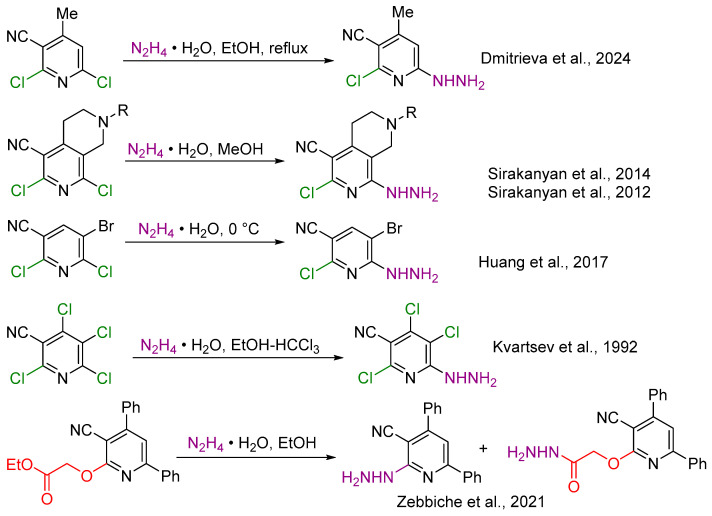
Competing processes in hydrazinolysis reactions with chloro/alkoxy-substituted nicotinonitriles (see Refs. [3,101,102,103,104,105]).

It should be noted that the chlorine atom or the RS group at the 2 position of a 3-cyanopyridine molecule is a relatively poor nucleofuge. Thus, it is known that in the case of 2,6-dichloronicotinonitriles, the halogen atom at the C-6 position is substituted first during hydrazinolysis [3,101,102,103,104] (Scheme 2). Furthermore, when hydrazinolysis is performed on esters of 3-cyanopyridine-2-(thio/oxy)acetic acid, the main product is often not the 2-hydrazinylnicotinonitrile but the corresponding carboxylic acid hydrazide (for example, [93,94,105]).

**Scheme 3:**
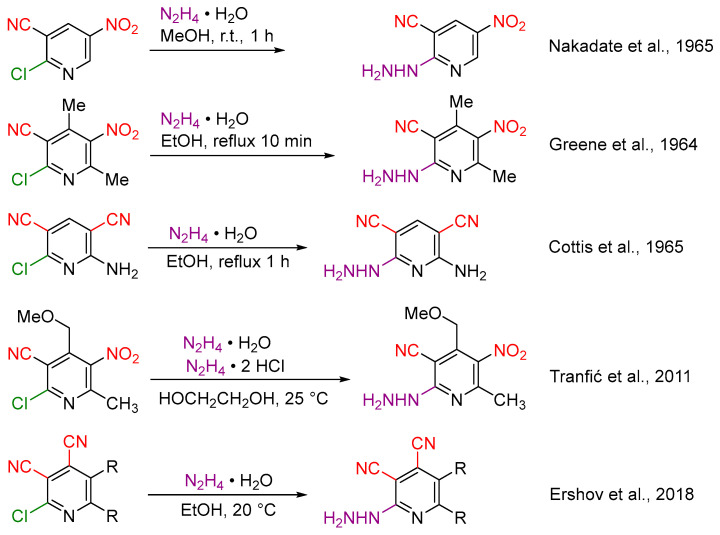
The reactions of highly electrophilic 2-chloronicotinonitriles with hydrazine (see Refs. [106,107,108,109,110]).

The hydrazinolysis reaction of 2-chloronicotinonitriles generally requires heating—for instance, refluxing in ethanol for 4–18 h [83,84,85,86,87,88,89,90,91,92], refluxing in DMF for 6 h [35], refluxing in dioxane for 3–12 h [74,75,76,77,78,79,80], or heating in 1-butanol at 110–112 °C [82]. In contrast, under relatively mild conditions (20–25 °C or with brief heating), the reaction between 2-chloropyridines and hydrazine occurs only if the pyridine ring contains two or more strong electron-withdrawing substituents [106,107,108,109,110] (Scheme 3).

**Scheme 4:**
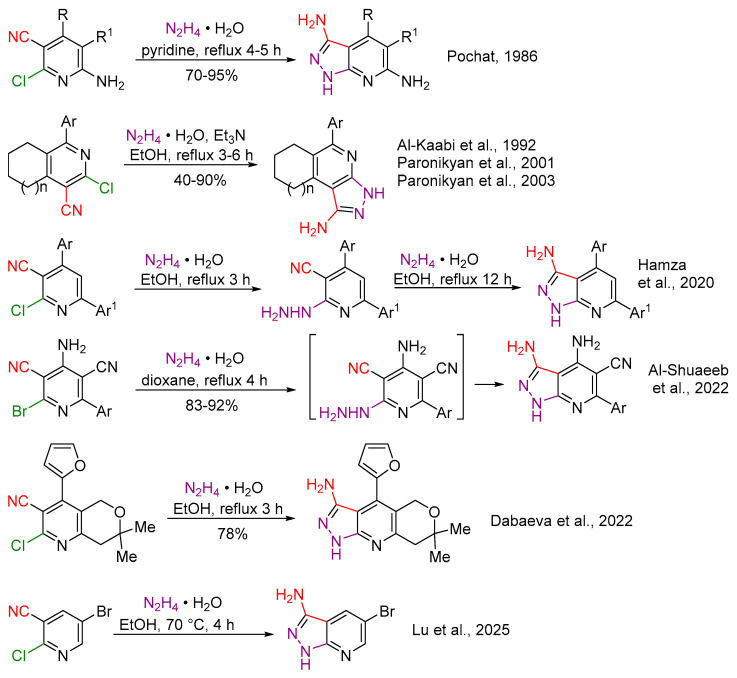
The formation of 3-amino-1*H*-pyrazolo[3,4-*b*]pyridines in the reactions of hydrazine hydrate with 2-halonicotinonitriles (see Refs. [90,111,112,113,114,115,116,118]).

However, the use of harsher reaction conditions promotes an intramolecular cyclization between the hydrazine fragment and the cyano group, yielding well-known 3-amino-1*H*-pyrazolo[3,4-*b*]pyridines [45,46,47,48,49,50,51,52,53,54,55]. This side reaction commonly accompanies or even competes with the formation of 2-hydrazinylnicotinonitriles. Moreover, in many cases, the isolation of 2-hydrazinyl-3-cyanopyridines is often impossible, and pyrazolo[3,4-*b*]pyridines are obtained as the sole products. Typical examples that demonstrate this pathway can be found in references [90,111,112,113,114,115,116,117,118] (Scheme 4).

**Scheme 5:**
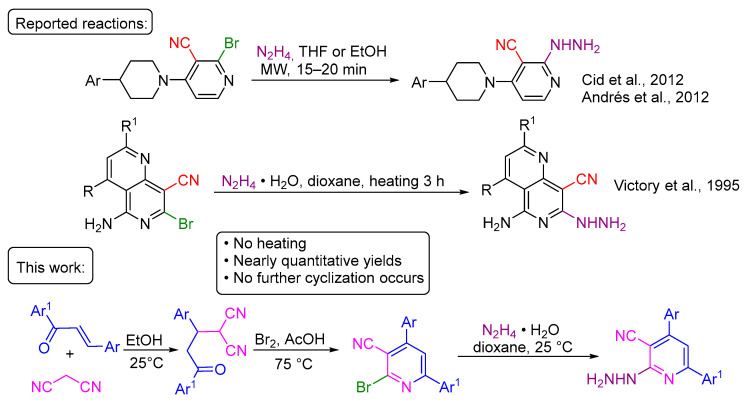
The preparation of 2-hydrazinylnicotinonitriles from 2-bromo-3-cyanopyridines (see Refs. [78,119,120]).

Given the aforementioned drawbacks and limitations of existing methods, developing new and optimized approaches for synthesizing 2-hydrazinylnicotinonitriles is highly desirable. This work presents an efficient route to 4,6-diaryl-2-hydrazinyl-3-cyanopyridines based on the reaction of 4,6-diaryl-2-bromo-3-cyanopyridines with hydrazine hydrate under mild conditions. The starting 2-bromo-3-cyanopyridines are readily available in high yields from inexpensive, commercially available acyclic precursors in only two steps (Scheme 5). In general, literature reports on the use of 2-bromo-3-cyanopyridines for the synthesis of 2-hydrazinylnicotinonitriles are limited to isolated examples [78,119,120] (Scheme 5). Notably, the reaction with 2-bromopyridines proceeds more rapidly and in higher yields compared to their 2-chloropyridine analogs.

## 2. Results and Discussion

### 2.1. Synthesis

**Figure 3:**
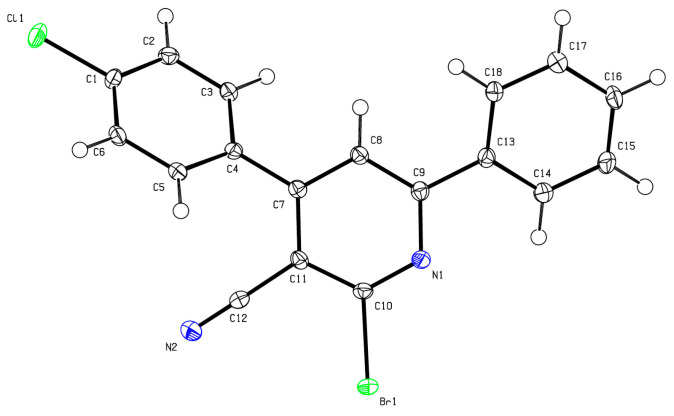
ORTEP drawing of the X-ray structure for 2-bromo-4-(4-chlorophenyl)-6-phenyl-nicotinonitrile **15a** with a numbering system (not the IUPAC standard) and ellipsoids with 50% probability (CCDC deposition number 2499675).

**Scheme 6:**
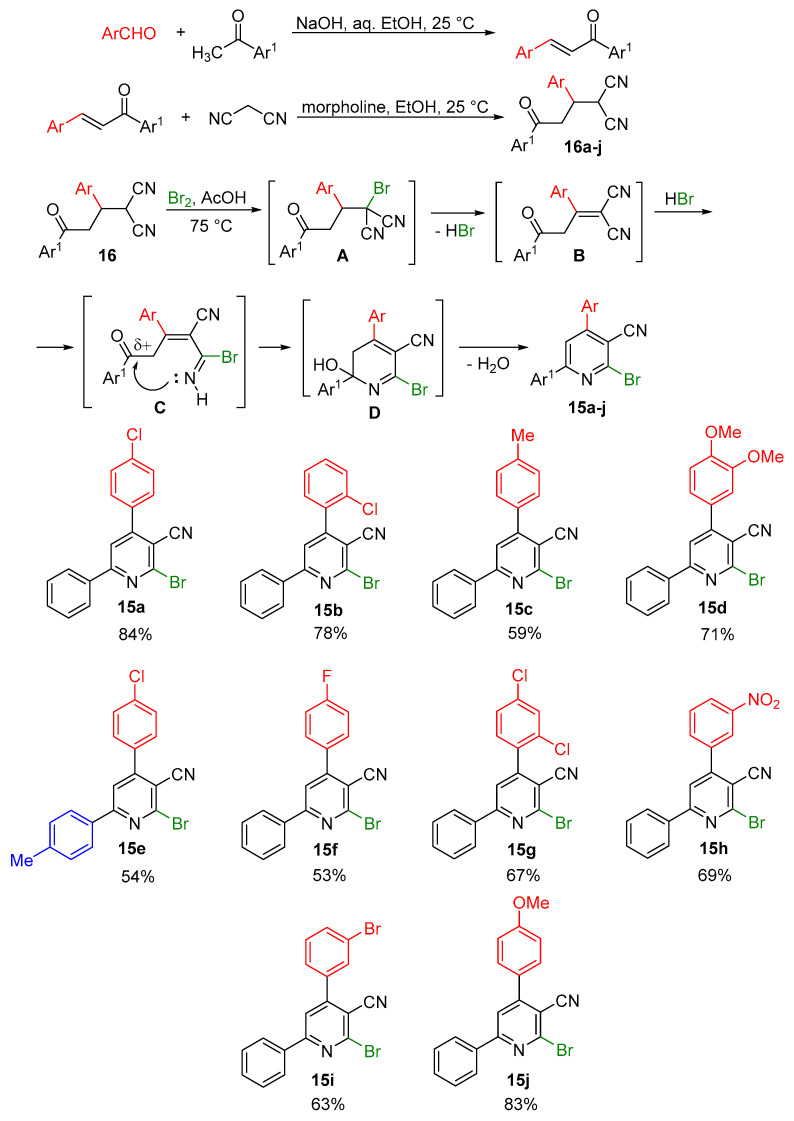
Synthesis of 4,6-diaryl-2-bromo-3-cyanopyridines **15**.

The starting 4,6-diaryl-2-bromo-3-cyanopyridines **15** were synthesized according to a modified procedure initially described by Shestopalov and Sharanin in papers [121,122]. Briefly, readily available α,β-unsaturated ketones (chalcones) were reacted with malononitrile in the presence of catalytic amounts of morpholine to yield the Michael adducts—δ-ketodinitriles **16a–j**. These intermediates were then treated with a solution of bromine in AcOH under gentle heating to afford 2-bromopyridines **15a–j** in 53–84% yields (Scheme 6). The reaction mechanism involves the initial formation of bromination products—2-bromo-2-(3-oxo-1,3-diarylpropyl)malononitriles **A**. Dehydrobromination of bromides **A**, followed by the addition of HBr to the cyano group of the unsaturated nitrile **B**, gives imidoyl bromides **C**. These intermediates subsequently undergo cyclization to form 2,3-dihydropyridine species **D**, which readily aromatize under the reaction conditions to give the 2-bromopyridines (Scheme 6). The structures of the bromides **15** were verified by FT-IR and NMR spectroscopy. Additionally, the structure of 2-bromo-4-(4-chlorophenyl)-6-phenylnicotinonitrile **15a** was definitively confirmed through single-crystal X-ray diffraction analysis, as shown in Figure 3.

**Figure 4:**
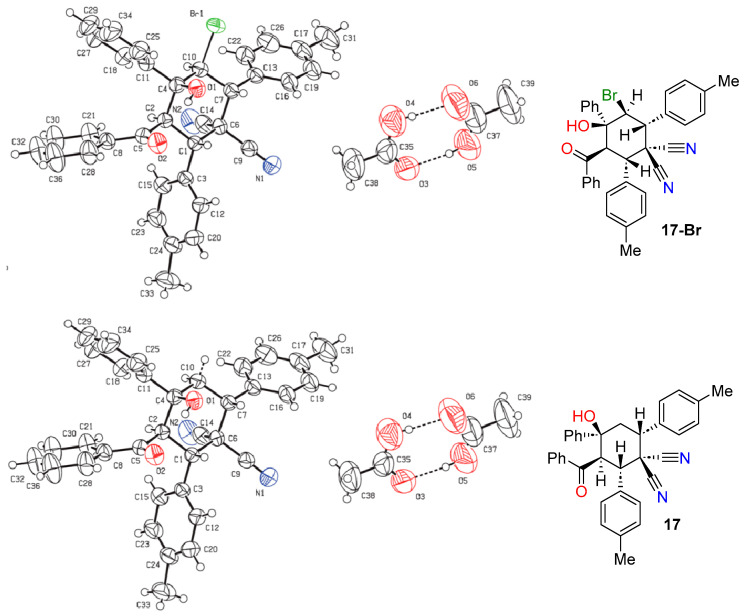
ORTEP drawing of the X-ray structure for (2S*,3R*,4S*,6R*)-3-benzoyl-5-bromo-4-hydroxy-4-phenyl-2,6-di-*p*-tolylcyclohexane-1,1-dicarbonitrile **17-Br** (upper image) and (2S*,3R*,4S*,6R*)-3-benzoyl-4-hydroxy-4-phenyl-2,6-di-*p*-tolylcyclohexane-1,1-dicarbonitrile **17** (bottom image) with solvate molecules of AcOH. Ellipsoids are given with 50% probability (CCDC deposition number 2499676).

Interestingly, during an attempt to isolate additional crops of 4,6-diaryl-2-bromo-3-cyanopyridine **15** by slow evaporation of the acetic acid mother liquor, small amounts of crystalline products were obtained in several cases. However, the IR spectra of these products indicated the presence of a ketone C=O group and non-conjugated cyano groups (C_sp_^3^–C≡N) in the region of ν 2250 cm^−1^. At the same time, the absorption bands for the conjugated cyano group, characteristic of compounds **15** (ν 2222–2230 cm^−1^), were absent from the spectra. X-ray structural analysis of the crystalline solid obtained by slow concentration from the mother liquor of compound **15c** revealed a substitutional disorder and showed that the isolated product consisted of co-crystals of (2S*,3R*,4S*,6R*)-3-benzoyl-4-hydroxy-4-phenyl-2,6-di-*p*-tolylcyclohexane-1,1-dicarbonitrile **17** and (2S*,3R*,4S*,6R*)-3-benzoyl-5-bromo-4-hydroxy-4-phenyl-2,6-di-*p*-tolylcyclohexane-1,1-dicarbonitrile **17-Br** at a ~4:6 ratio as a solvate with AcOH (Figure 4).

**Scheme 7:**
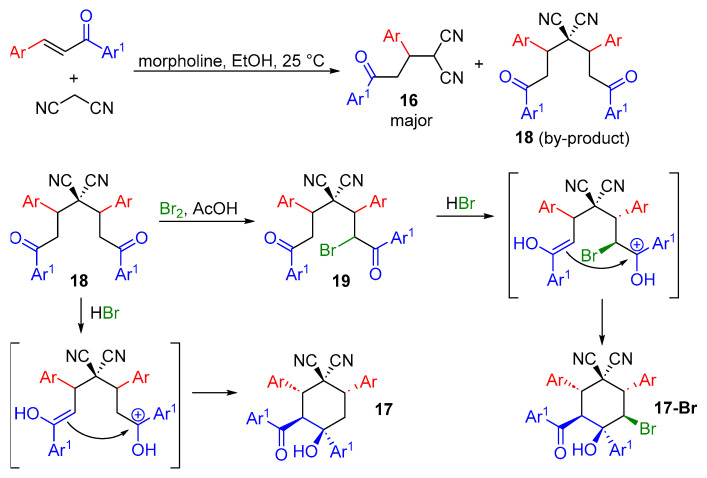
A plausible mechanism for the formation of cyclohexanols **17** and **17-Br**.

We propose the following pathway for the formation of this unusual product. During the reaction of chalcones with malononitrile, alongside the formation of the δ-ketodinitriles **16**, by-products **18**—the Michael adducts with a 2:1 stoichiometry—are also generated (Scheme 7). The formation of such *bis*-adducts under analogous conditions has been reported in the literature [123,124,125]. Bromination of *bis-*adducts **18** yields the only possible α-bromoketones **19**. Subsequently, both the adducts **18** and their brominated derivatives **19** undergo an acid-catalyzed aldol-type addition, leading to the cyclohexanols **17** and **17-Br**. Bromination likely precedes cyclization, as bromination of compounds like **17** would be expected to proceed with different regioselectivity.

The observed stereochemistry of the products appears to result from steric repulsion between the bulky aryl and benzoyl substituents and stabilization provided by the *cis-*orientation of the OH and C=O benzoyl groups, the latter being facilitated by hydrogen bonding during the cyclization.

It is important to note that compound **17-Br** and related brominated analogs are not reported in the literature, in contrast to the well-documented compounds **17** [123,124,125,126,127,128,129,130,131,132,133,134,135,136,137,138,139,140]. Nevertheless, the reactions and properties of this class of compounds remain largely unexplored. While further research in this area is warranted, it lies beyond the scope of the current study and will be examined in detail in future works.

**Scheme 8:**
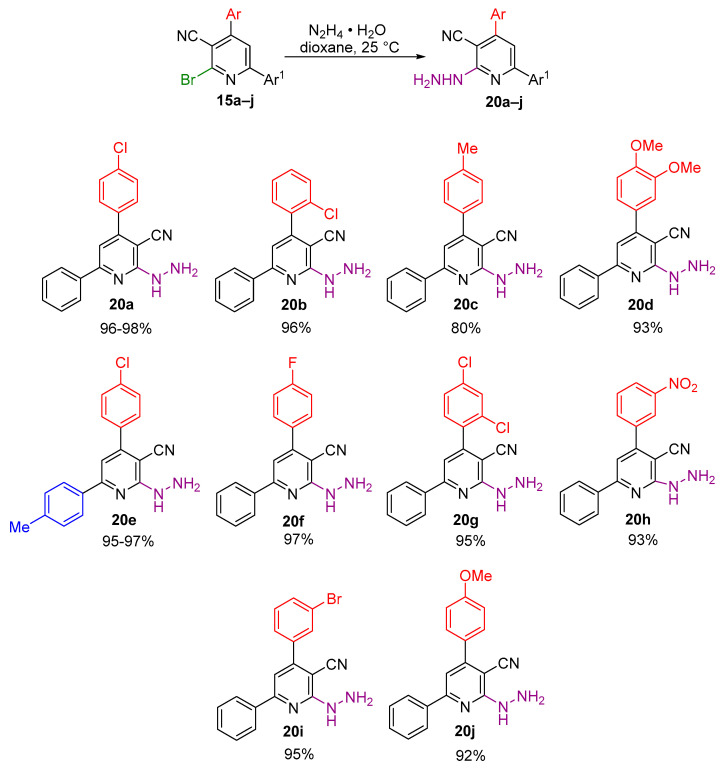
Synthesis, diversity, and yields of 2-hydrazinylnicotinonitriles **20**.

The prepared 4,6-diaryl-2-bromonicotinonitriles **15a–j** react with hydrazine hydrate in 1,4-dioxane under mild conditions (25 °C) to form the expected 4,6-diaryl-2-hydrazinyl-3-cyanopyridines **20a–j**. The reaction proceeds in high yields (80–97%) (Scheme 8), with no observed formation of further cyclization products—specifically, 3-amino-1*H*-pyrazolo[3,4-*b*]pyridines—in any of the cases. FT-IR, NMR, and HRMS spectral data were used to confirm the structures of the synthesized compounds **20**.

Compounds **20** are yellow powders, typically sparingly soluble in water, Et_2_O, or hydrocarbons and soluble in hot EtOH, dioxane, DMSO, or DMF.

The FT-IR spectra of 2-hydrazinylnicotinonitriles **20** exhibited absorption bands for the conjugated cyano group at ν 2206–2212 cm^−1^. In addition, two strong bands corresponding to the NH–NH_2_ fragment vibrations were observed at ν 3411–3302 cm^−1^ and 3207–3326 cm^−1^. In the ^1^H NMR spectra of hydrazines **20a–j**, the protons of the primary amino group appeared as a very broad singlet at δ 4.58–4.76 ppm. The signals of C(5)H and –NH– protons appeared as sharp singlets at δ 7.25–7.44 ppm and as broadened peaks at δ 8.27–8.55 ppm, respectively.

The ^13^C NMR spectra of 3-cyano-2-hydrazinylpyridines **20** showed characteristic signals for the following carbons: C-3 (δ 86.2–88.4 ppm), C-5 (δ 109.2–109.7 ppm), cyano group (δ 115.5–116.7 ppm), pyridine C-4 (δ 152.6–155.3 ppm), C-6 (δ 156.4–158.0 ppm), and C-2 (δ 160.2–161.0 ppm).

**Scheme 9:**
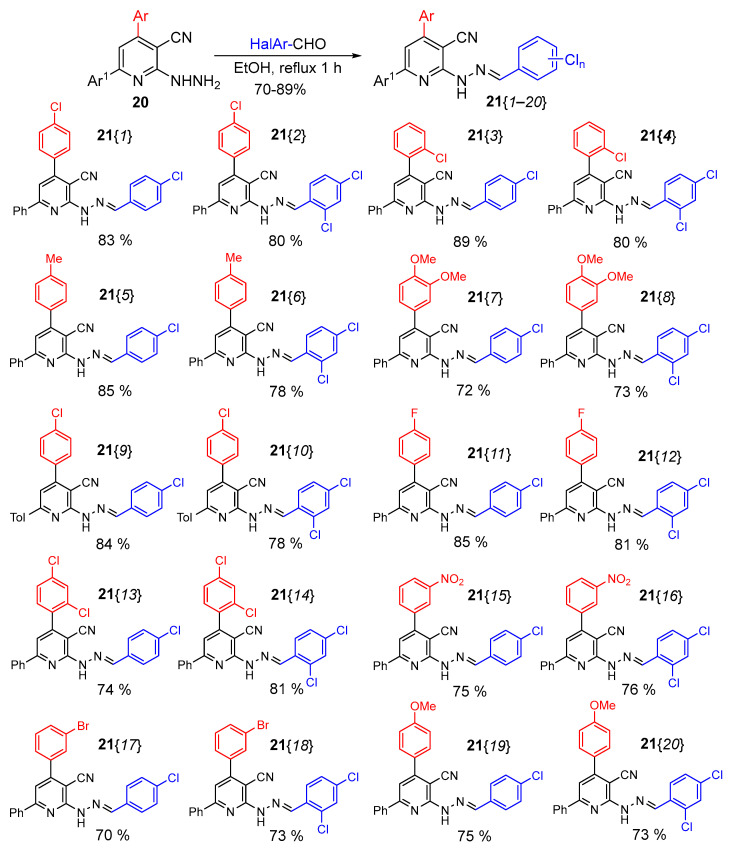
Synthesis, diversity, and yields of hydrazones **21**.

We investigated the properties of the synthesized compounds. Given the potential of hydrazinylnicotinonitriles and their derivatives in agrochemistry (see, e.g., [3,4,5,38,70,71,72,73,141]), we prepared a series of new hydrazones **21** by reacting compounds **20** with aromatic aldehydes (Scheme 9).

It is well-known that halogenated compounds play an important role in agrochemistry [142,143,144,145,146,147,148,149]. This can be explained by the physico-chemical effects (such as steric and electronic effects, C–Hal bond polarity, thermal and metabolic stability, etc.) that arise from the introduction of halogens or halogen-containing substituents into biologically active molecules, such as insecticides, acaricides, plant growth regulators, and herbicide safeners. Thus, in each area of crop protection (fungicides, insecticides, or herbicides), 12 halogen-containing products (~60%) are among the 20 best-selling agrochemicals [144]. As of 2020, out of 48 new agrochemicals provisionally approved by the International Organization for Standardization (ISO), 39 (~81%) contain halogen atoms as substituents [145]. The greater availability of chlorine compared to other halogens accounts for the prevalence of chlorinated agrochemicals [146,147,148,149].

On the other hand, pyridine is the most important heterocyclic scaffold in agrochemistry [150]. (Poly)halogenated pyridines exhibit pronounced activity and are widely represented on the market [150,151,152].

For this reason, we selected chlorinated aromatic aldehydes (4-chlorobenzaldehyde and 2,4-dichlorobenzaldehyde) to prepare a small library of new hydrazones **21**. The synthesis, diversity, and yields of the resulting products **21** are presented in Scheme 9.

Hydrazones **21** are yellow powders that are moderately soluble in EtOH and 1,4-dioxane, and readily soluble in acetone, DMF, or DMSO. The FT-IR spectra of hydrazones **21** exhibit absorption bands assigned to N–H stretching vibrations in the region of ν 3178–3327 cm^−1^. The bands corresponding to conjugated C≡N groups were observed at ν 2206–2218 cm^−1^. In the ^1^H NMR spectra of the hydrazones derived from 4-chlorobenzaldehyde, the proton signals of the –CH=N– group appear at δ 8.11–8.14 ppm, while the NH signals are observed at δ 11.73–11.97 ppm. In the derivatives of 2,4-dichlorobenzaldehyde, the corresponding signals are found at δ 8.48–8.56 ppm and δ 11.95–12.16 ppm, respectively.

### 2.2. Agrochemical Studies

The prepared compounds were evaluated as herbicide antidotes against herbicide 2,4-dichlorophenoxyacetic (2,4-D). Though this widely used herbicide is not considered significantly toxic to humans [153], its application can still have negative side effects, such as the inhibition of crop growth, potentially reducing yields by 15–60%. To mitigate these effects and improve crop yields, some agrochemicals known as herbicide safeners, or antidotes, are used. These antidotes (for reviews, see [154,155,156,157,158]) protect crops by neutralizing phytotoxins without reducing the herbicide’s effectiveness against weeds. Typically, herbicide safeners appear to be non-toxic to cultivated plants and can sometimes stimulate plant growth.

Following the reported procedures [159,160,161,162], we investigated the efficacy of the new nicotinonitriles as 2,4-D antidotes. The experiments were performed on sunflower seedlings of the Master cultivar. The antidote effect (*A*) was calculated as the ratio of the hypocotyl or root length of seedlings treated with both the herbicide and an antidote to the length of those treated with 2,4-D alone, as defined in Equation (1). Further details are provided in the Section 3.

*A* = (*V*_exp_/*V*_ref_) × 100%, (1)

where *V*_exp_ is the organ length (in mm) of seedlings treated with both 2,4-D herbicide and an antidote and *V*_ref_ is the organ length of the reference seedlings treated with 2,4-D only.

We found that several of the novel 2-hydrazinylpyridines **20** and hydrazones **21** demonstrated a pronounced antidote effect in laboratory experiments (Table 1).

As shown in Table 1, 4-chlorophenyl derivative **20a** is the most active compound among the 2-hydrazinyl pyridines **20**. Compound **20a** counteracts the negative effect of 2,4-D by 34–86% on sunflower seedling roots and by 30–46% on hypocotyls. Other notable compounds include hydrazines **20b,f,i**, which demonstrate significant antidote activity on seedling roots (up to 80% effect), though their impact on hypocotyls is less consistent.

Among the hydrazones **21**, compounds with 3-bromophenyl substituents unexpectedly showed the highest activity. Specifically, hydrazone **21**{*17*} reduced the phytotoxic effects of the herbicide on hypocotyls by 38–52% and on sunflower roots by 61–86%. Hydrazone **21**{*18*} reduced the negative effect on hypocotyls by 27–52% and on roots by 59–97%. Hydrazones **21**{*1,2*} bearing 4-chlorophenyl substituents primarily influenced root growth (50–84% antidote effect) but exhibited only moderate activity on hypocotyls.

## 3. Materials and Methods

The ^1^H, ^13^C, and ^13^C DEPTQ NMR spectra were recorded in DMSO-d_6_ or acetone-d_6_ solutions using a Bruker AVANCE-III HD spectrometer (Bruker, Göttingen, Germany) operating at 400.40 MHz for ^1^H and 100.61 MHz for ^13^C nuclei or a JEOL JNM-ECA-400 spectrometer (JEOL, Tokyo, Japan) operating at 399.78 MHz for ^1^H and 100.53 MHz for ^13^C nuclei. Residual solvent signals were used as internal standards, set at 2.49 ppm for ^1^H and 39.50 ppm for ^13^C in DMSO-d_6_. Elemental analyses were performed with a Carlo Erba 1106 Elemental Analyzer (Carlo Erba, Milan, Italy). FTIR spectra were acquired on a Bruker Vertex 70 spectrometer (Bruker, Ettlingen, Germany) using an ATR device or on an Infraspec FSM 2201 spectrometer (Infraspec, Saint-Petersburg, Russia) using KBr pellets or Nujol mulls. Single-crystal X-ray diffraction data were collected on an Agilent SuperNova, Dual, Cu at home/near, Atlas diffractometer (Agilent Technologies, Santa Clara, CA, USA). High-resolution mass spectra (HRMS) were obtained using a Bruker MaXis Impact spectrometer (Bruker, Bremen, Germany) with electrospray ionization and HCO_2_Na–HCO_2_H calibration. Samples were dissolved in acetonitrile at 37–38 °C under ultrasonication. NMR, FTIR, and HRMS spectral charts, along with X-ray analysis data, are provided in the Electronic Supplementary Materials (Figures S1–S104 and Tables S1–S15).

Reaction progress and compound purity were monitored by TLC on Sorbfil-A plates (Imid Ltd., Krasnodar, Russia) using solvent systems of Me_2_CO–hexane (2:1), Me_2_CO–PhMe (3:2), HCCl_3_–PhMe (1:1), or AcOEt–light petroleum (1:1). Visualization was achieved under UV light and with iodine vapor. All reagents and solvents were purchased from BioInLabs (Rostov-on-Don, Russia) and used without further purification.

**4,6-Diaryl-2-bromo-3-cyanopyridines** (**15a–j**) (Scheme 6) were prepared as follows.

*A. Synthesis of 2-(1,3-diaryl-3-oxopropyl)malononitriles (δ-ketodinitriles)* **16**.

The corresponding chalcone (0.01 mol) was dissolved or suspended in EtOH (20 mL) at 25 °C. With stirring, malononitrile (0.75 g, 0.0114 mol) was added, followed after 1–2 min by one drop of morpholine. The resulting mixture was vigorously stirred at 25 °C, and the reaction progress was monitored by TLC (eluent: acetone–toluene, 3:2). After 1–1.5 h, complete consumption of the chalcone and formation of the target Michael adduct **16** were observed. Depending on the structure of the starting chalcone, the product either crystallized directly from the reaction mixture or precipitated upon the addition of cold water (5–10 mL). The precipitated product was used in the next step without purification. If analytically pure samples were required, the Michael adducts **16** could be recrystallized from EtOH.

*B. Bromination of the Michael adducts* **16**.

The corresponding δ-ketodinitrile **16** was dissolved in glacial AcOH (~100 mL per 5 g of ketodinitrile) with gentle heating (to about 60 °C). A solution of an equimolar amount of Br_2_ in 10 mL of glac. AcOH was added dropwise to the resulting light-yellow transparent solution with vigorous stirring. The bromine solution must be added at a rate that prevents the reaction temperature from exceeding 75 °C. After approximately half of the bromine has been added, the solution decolorizes. Almost immediately after decolorization, intensive precipitation of the 2-bromopyridine **15** begins. If the reaction mixture solidifies and stirring becomes impossible, the mixture should be diluted with hot AcOH. After the complete addition of bromine, stirring was continued for another 1.5 h. The mixture was then cooled to 25 °C, and the solid of 2-bromopyridine was filtered off and washed sequentially with AcOH and aq. EtOH until the washings were neutral with petroleum ether. The resulting 2-bromonicotinonitriles **15** were obtained as fluffy light-yellow powders or fibrous crystals. The 2-bromonicotinonitriles **15** were used in the next step without purification. If analytically pure samples were required, the crude product was either recrystallized from AcOH or, in cases of insufficient solubility, the suspension was boiled in AcOH for 20–30 min.

*2-Bromo-4-(4-chlorophenyl)-6-phenylnicotinonitrile* **15a** was isolated in 84% yield, m.p. 188–189 °C (Lit. [122]: m.p. 231–232 °C (from MeNO_2_)). FTIR (KBr), ν_max_, cm^−1^: 2222 (C≡N). ^1^H NMR (400 MHz, DMSO-*d*_6_): 7.50–7.57 (m, 4H, H Ar), 7.66–7.69 (m, 3H, H Ar), 7.80–7.84 (m, 2H, H Ar), 8.22–8.28 (m, 2H, H Ar). ^13^C DEPTQ NMR (101 MHz, DMSO-*d*_6_): 115.1 (C-3), 116.4 (C≡N), 119.8 * (C-5), 127.8 * (2 CH Ar), 129.0 * (2 CH Ar), 129.2 * (2 CH Ar), 130.5 * (CH Ph), 130.9 * (2 CH Ar), 134.1 (C Ar), 135.4 (C Ar), 135.1 (C Ar), 144.3 (C–Br), 155.0 (C-4), 159.4 (C-6). * Negatively phased signals. Elemental Analysis: found, %: C, 58.60; H, 2.91; N, 7.40. C_18_H_10_BrClN_2_ (M 369.65). Calculated, %: C, 58.49; H, 2.73; N, 7.58.

*2-Bromo-4-(2-chlorophenyl)-6-phenylnicotinonitrile* **15b** was isolated in 78% yield, m.p. 185–186 °C (Lit. [122]: m.p. 187–188 °C (from AcOH)). FTIR (KBr), ν_max_, cm^−1^: 2230 (C≡N). NMR spectral data were identical to those reported in [122].

*2-Bromo-4-(4-methylphenyl)-6-phenylnicotinonitrile* **15c** was isolated in 59% yield, m.p. 191–191 °C. FTIR (nujol), ν_max_, cm^−1^: 2224 (C≡N). NMR spectral data were identical to those reported in [163].

*2-Bromo-4-(3,4-dimethoxyphenyl)-6-phenylnicotinonitrile* **15d** was isolated in 71% yield, m.p. 194–195 °C. FTIR (KBr), ν_max_, cm^−1^: 2227 (C≡N).

*2-Bromo-4-(4-chlorophenyl)-6-(4-methylphenyl)nicotinonitrile* **15e** was isolated in 54% yield, m.p. 187–188 °C (Lit. [122]: m.p. 282–283 °C (from dioxane)). FTIR (KBr), ν_max_, cm^−1^: 2222 (C≡N). NMR spectral data were identical to those reported in [122].

*2-Bromo-4-(4-fluorophenyl)-6-phenylnicotinonitrile* **15f** was isolated in 53% yield, m.p. 181–183 °C (Lit. [164]: m.p. 184–187 °C). FTIR (KBr), ν_max_, cm^−1^: 2222 (C≡N). NMR spectral data were identical to those reported in [164].

*2-Bromo-4-(2,4-dichlorophenyl)-6-phenylnicotinonitrile* **15g** was isolated in 67% yield, m.p. 180–181 °C (Lit. [122]: m.p. 160–161 °C (from MeNO_2_)). Spectral data were identical to those reported in [122].

*2-Bromo-4-(3-nitrophenyl)-6-phenylnicotinonitrile* **15h** was isolated in 69% yield, m.p. 186–188 °C. FTIR (KBr), ν_max_, cm^−1^: 2222 (C≡N).

*2-Bromo-4-(3-bromophenyl)-6-phenylnicotinonitrile* **15i** was isolated in 63% yield, m.p. 191–192 °C. FTIR (KBr), ν_max_, cm^−1^: 2222 (C≡N).

*2-Bromo-4-(4-methoxyphenyl)-6-phenylnicotinonitrile* **15j** was isolated in 83% yield, m.p. 192–195 °C (Lit. [122]: m.p. 218–218 °C (from MeNO_2_)). Spectral data were identical to those reported in [122,164].

**4,6-Diaryl-2-hydrazinyl-3-cyanopyridines** (**20a–j**) (Scheme 8) were prepared as follows. The corresponding 2-bromonicotinonitrile **15a–j** (5.5–6 mmol) was dissolved in 1,4-dioxane (~15 mL per 2 g of 2-bromopyridine **15**). An excess of hydrazine hydrate (25–30 mmol) was added to the solution formed. The resulting emulsion was stirred vigorously at 25 °C. The reaction progress was monitored by TLC (eluent: chloroform–toluene, 1:1). Complete conversion was typically achieved within 5–6 h, though in some cases, stirring was continued for 24 h. Upon reaction completion, dioxane was removed under reduced pressure at ambient temperature. The residue was triturated with cold water to remove hydrazine and hydrazinium hydrobromide. The precipitate was filtered off and washed with water and then with petroleum ether, yielding hydrazines **20** as yellow powders. For purification, the compounds can be recrystallized from MeOH or dioxane.

*4-(4-Chlorophenyl)-2-hydrazinyl-6-phenylnicotinonitrile* **20a** was isolated in 97% yield, m.p. 160–162 °C. FTIR (nujol), ν_max_, cm^−1^: 3304 br (N–H), 2206 (C≡N). ^1^H NMR (400 MHz, DMSO-*d*_6_): 4.67 (br s, 2H, NH_2_), 7.32 (s, 1H, H-5), 7.47–7.51 (m, 3H, H Ph), 7.62 (d, ^3^*J* = 8.7 Hz, 2H, H-3, and H-5, 4-ClC_6_H_4_), 7.69 (d, ^3^*J* = 8.7 Hz, 2H, H-3, and H-5, 4-ClC_6_H_4_), 8.23–8.26 (m, 2H, H Ph), 8.41 (br s, 1H, NH). ^13^C DEPTQ NMR (101 MHz, DMSO-*d*_6_): 86.3 (C-3), 109.2 * (C(5)H), 116.2 (C≡N), 127.5 * (2 CH Ar), 128.6 * (2 CH Ar), 128.8 * (2 CH Ar), 130.3 * (CH Ph), 130.4 * (2 CH Ar), 134.5 (C^1^ Ar), 135.7 (C^1^ Ar), 137.5 (C–Cl), 154.0 (C-4), 158.0 (C–6), 160.8 (C–NH). * Negatively phased signals. HRMS (ESI) *m/z*: calculated for C_18_H_14_ClN_4_ [M + H]^+^: 321.090699, found 321.0891 (Δ 4.98 ppm). Elemental Analysis: found, %: C, 67.36; H, 4.21; N, 17.40. C_18_H_13_ClN_4_ (M 320.78). Calculated, %: C, 67.40; H, 4.09; N, 17.47.

*4-(2-Chlorophenyl)-2-hydrazinyl-6-phenylnicotinonitrile* **20b** was isolated in 96% yield, m.p. 158–160 °C. FTIR (nujol), ν_max_, cm^−1^: 3315, 3250 br (N–H), 2208 (C≡N). ^1^H NMR (400 MHz, DMSO-*d*_6_): 4.62 (br s, 2H, NH_2_), 7.25 (s, 1H, H-5), 7.47–7.54 (m, 7H, H Ar), 7.64 (dd, ^3^*J* = 8.2 Hz, ^4^*J* = 1.4 Hz, 1H, H Ar), 8.21–8.24 (m, 2H, H Ph), 8.49 (br s, 1H, NH). ^13^C NMR (101 MHz, DMSO-*d*_6_): 88.3 (C-3), 109.7 (C(5)H), 115.5 (C≡N), 127.4 (2 CH Ar), 127.5 (CH Ar), 128.7 (2 CH Ar), 129.7 (CH Ar), 130.3 (CH Ph), 130.6 (CH Ar), 130.9 (CH Ar), 131.3 (C Ar), 136.2 (C Ar), 137.3 (C Ar), 153.4 (C-4), 157.8 (C–6), 160.2 (C–NH). Elemental Analysis: found, %: C, 67.29; H, 4.24; N, 17.38. C_18_H_13_ClN_4_ (M 320.78). Calculated, %: C, 67.40; H, 4.09; N, 17.47. HRMS (ESI) *m/z*: calculated for C_18_H_14_ClN_4_ [M + H]^+^: 321.090699, found 321.0904 (Δ 0.93 ppm).

*2-Hydrazinyl-4-(2-methylphenyl)-6-phenylnicotinonitrile* **20c** was isolated in 80% yield, m.p. 163–165 °C. FTIR (nujol), ν_max_, cm^−1^: 3315, 3267 br (N–H), 2206 (C≡N). ^1^H NMR (400 MHz, DMSO-*d*_6_): 2.38 (s, 3H, Me), 4.59 (br s, 2H, NH_2_), 7.29 (s, 1H, H-5), 7.48–7.50 (m, 3H, H Ph), 7.56 (d, ^3^*J* = 7.8 Hz, 2H, H Ar), 8.23–8.25 (m, 2H, H Ph), 8.31 (br s, 1H, NH). ^13^C NMR (101 MHz, DMSO-*d*_6_): 20.9 (Me), 88.4 (C-3), 109.3 (C(5)H), 116.4 (C≡N), 127.4 (2 CH Ar), 128.3 (CH Ar), 128.6 (2 CH Ar), 129.3 (CH Ar), 130.2 (CH Ph), 134.0 (C–Me), 137.6 (C^1^ Ar), 139.4 (C^1^ Ar), 155.2 (C-4), 157.7 (C–6), 160.9 (C–NH). Elemental Analysis: found, %: C, 75.90; H, 5.44; N, 18.66. C_19_H_16_N_4_ (M 300.37). Calculated, %: C, 75.98; H, 5.37; N, 18.65.

*2-Hydrazinyl-4-(3,4-dimethoxyphenyl)-6-phenylnicotinonitrile* **20d** was isolated as solvate with dioxane (2:1) in 93% yield, m.p. 152–155 °C. FTIR (nujol), ν_max_, cm^−1^: 3314, 3279 br (N–H), 2212 (C≡N). ^1^H NMR (400 MHz, DMSO-*d*_6_): 3.55 (br s, dioxane), 3.83 (s, 3H, MeO), 3.84 (s, 3H, MeO), 4.69 (br s, 2H, NH_2_), 7.11 (d, ^3^*J* = 7.8 Hz, 1H, H Ar), 7.23–7.27 (m, 2H, H Ar), 7.34 (s, 1H, H-5), 7.48–7.50 (m, 3H, H Ph), 8.24–8.27 (m, 3H, H Ph, and NH overlapped). ^13^C NMR (101 MHz, DMSO-*d*_6_): 55.6 (2 C, 2 MeO), 66.4 (CH_2_O dioxane), 86.3 (C-3), 109.3 (C(5)H), 111.6 (CH Ar), 112.1 (CH Ar), 116.7 (C≡N), 121.2 (CH Ar), 127.5 (2 CH Ph), 128.6 (2 CH Ph), 129.0 (C^1^ Ar), 130.1 (CH Ph), 137.7 (C^1^ Ph), 148.6 (C–OMe), 150.1 (C–OMe), 155.0 (C-4), 157.6 (C–6), 161.0 (C–NH). HRMS (ESI) *m/z*: calculated for C_20_H_19_N_4_O_2_ [M + H]^+^: 347.150801, found 347.1503 (Δ 1.44 ppm).

*4-(4-Chlorophenyl)-2-hydrazinyl-6-(4-methylphenyl)nicotinonitrile* **20e** was isolated in 96% yield, m.p. 160–162 °C. FTIR (nujol), ν_max_, cm^−1^: 3400, 3326 br (N–H), 2210 (C≡N). ^1^H NMR (400 MHz, DMSO-*d*_6_): 2.38 (s, 3H, Me), 4.60 (br s, 2H, NH_2_), 7.31 (s, 1H, H-5), 7.35 (d, ^3^*J* = 7.8 Hz, 2H, H Ar), 7.53–7.57 (m, 4H, H Ar, two doublets overlapped), 8.29 (d, ^3^*J* = 8.2 Hz, 2H, H Ar), 8.34 (br s, 1H, NH). ^13^C NMR (101 MHz, DMSO-*d*_6_): 20.9 (Me), 86.7 (C-3), 109.2 (C(5)H), 116.3 (C≡N), 128.4 (2 CH Ar), 128.6 (2 CH Ar), 129.25 (2 CH Ar), 129.27 (2 CH Ar), 133.8 (C–Me), 135.0 (C–Cl), 136.4 (C^1^ Ar), 139.5 (C^1^ Ar), 155.3 (C-4), 156.4 (C–6), 160.8 (C–NH). HRMS (ESI) *m/z*: calculated for C_19_H_15_ClN_4_ [M + H]^+^: 335.106349, found 335.1061 (Δ 0.74 ppm).

*4-(4-Fluorophenyl)-2-hydrazinyl-6-phenylnicotinonitrile* **20f** was isolated in 97% yield, m.p. 153–155 °C. FTIR (nujol), ν_max_, cm^−1^: 3302, 3246 br (N–H), 2208 (C≡N). ^1^H NMR (400 MHz, DMSO-*d*_6_): 4.59 (br s, 2H, NH_2_), 7.31 (s, 1H, H-5), 7.37–7.41 (m, 2H, H Ar), 7.48–7.50 (m, 3H, H Ph), 7.71–7.74 (m, 2H, H Ar), 8.24–8.26 (m, 2H, H Ph), 8.39 (br s, 1H, NH). ^13^C NMR (101 MHz, DMSO-*d*_6_): 86.5 (C-3), 109.4 (C(5)H), 115.7 (d, ^2^*J*_C–F_ = 21.1 Hz, 2 CH Ar), 116.3 (C≡N), 127.5 (2 CH Ph), 128.6 (2 CH Ph), 130.2 (C(4)H Ph), 130.9 (d, ^3^*J*_C–F_ = 8.6 Hz, 2 CH Ar), 133.3 (d, ^4^*J*_C–F_ = 2.9 Hz, C^1^ Ar), 137.5 (C^1^ Ph), 154.2 (C-4), 157.9 (C–6), 160.8 (C–NH), 161.7, 164.2 (d, ^1^*J*_C–F_ = 247.3 Hz, C–F). HRMS (ESI) *m/z*: calculated for C_18_H_14_FN_4_ [M + H]^+^: 305.120249, found 305.1205 (Δ −0.82 ppm).

*4-(2,4-Dichlorophenyl)-2-hydrazinyl-6-phenylnicotinonitrile* **20g** was isolated in 95% yield, m.p. 142–143 °C. FTIR (nujol), ν_max_, cm^−1^: 3307, 3246 br (N–H), 2210 (C≡N). ^1^H NMR (400 MHz, DMSO-*d*_6_): 4.62 (br s, 2H, NH_2_), 7.26 (s, 1H, H-5), 7.47–7.49 (m, 3H, H Ar), 7.53 (d, ^3^*J* = 8.2 Hz, 1H, H-6 Ar), 7.61 (dd, ^3^*J* = 8.2 Hz, ^4^*J* = 1.8 Hz, 1H, H-5 Ar), 7.85 (d, ^4^*J* = 1.8 Hz, 1H, H-3 Ar), 8.20–8.23 (m, 2H, H Ph), 8.55 (br s, 1H, NH). ^13^C NMR (101 MHz, DMSO-*d*_6_): 88.0 (C-3), 109.6 (C(5)H), 115.5 (C≡N), 127.4 (2 CH Ar), 127.8 (CH Ar), 128.7 (2 CH Ar), 129.2 (CH Ar), 130.4 (CH Ph), 132.0 (CH Ar), 132.6 (C^1^ Ar), 134.7 (C–Cl), 135.2 (C–Cl), 137.2 (C^1^ Ph), 152.6 (C-4), 157.9 (C–6), 160.2 (C–NH). HRMS (ESI) *m/z*: calculated for C_18_H_13_Cl_2_N_4_ [M + H]^+^: 355.051727, found 355.0516 (Δ 0.36 ppm).

*2-Hydrazinyl-4-(3-nitrophenyl)-6-phenylnicotinonitrile* **20h** was isolated as solvate with dioxane (2:1) in 93% yield, m.p. 157–159 °C. FTIR (nujol), ν_max_, cm^−1^: 3312, 3245 br (N–H), 2210 (C≡N), 1535 (NO_2 asymm_), 1350 (NO_2 symm_). ^1^H NMR (400 MHz, DMSO-*d*_6_): 3.55 (s, CH_2_O, dioxane), 4.76 (br s, 2H, NH_2_), 7.44 (s, 1H, H-5), 7.48–7.50 (m, 3H, H Ph), 7.62–7.63 (m, 1H, H Ar), 7.83–7.86 (m, 1H, H Ar), 8.12–8.20 (m, 2H, H Ar), 8.26–8.29 (m, 2H, H Ph), 8.53 (br s, 1H, NH). ^13^C NMR (101 MHz, DMSO-*d*_6_): 66.4 (dioxane), 88.3 (C-3), 109.4 (C(5)H), 116.1 (C≡N), 123.4 (CH Ar), 124.3 (CH Ar), 127.6 (2 CH Ph), 128.6 (2 CH Ph), 128.8 (CH Ar), 130.2 (C(4)H Ph) 130.4 (CH Ar), 135.3 (C^1^ Ar), 137.4 (C^1^ Ph), 147.8 (C–NO_2_), 155.6 (C-4), 158.2 (C–6), 160.8 (C–NH). HRMS (ESI) *m/z*: calculated for C_18_H_14_N_5_O_2_ [M + H]^+^: 332.114750, found 332.1143 (Δ 1.36 ppm).

*4-(3-Bromophenyl)-2-hydrazinyl-6-phenylnicotinonitrile* **20i** was isolated as solvate with dioxane (1:1) in 97% yield, m.p. 165–166 °C. FTIR (nujol), ν_max_, cm^−1^: 3309, 3207 br (N–H), 2210 (C≡N). ^1^H NMR (400 MHz, DMSO-*d*_6_): 3.55 (s, CH_2_O, dioxane), 4.66 (br s, 2H, NH_2_), 7.36 (s, 1H, H-5), 7.48–7.54 (m, 4H, H Ar), 7.66–7.75 (m, 2H, H Ar), 7.86–7.87 (m, 1H, H Ar), 8.26–8.28 (m, 2H, H Ph), 8.43 (br s, 1H, NH). ^13^C NMR (101 MHz, DMSO-*d*_6_): 66.4 (dioxane), 86.4 (C-3), 109.4 (C(5)H), 116.1 (C≡N), 121.9 (C–Br), 127.6 (2 CH Ph), 127.7 (CH Ar), 128.6 (2 CH Ph), 130.3 (C(4)H Ph) 130.8 (CH Ar), 131.1 (CH Ar), 132.4 (CH Ar), 137.5 (C^1^ Ph), 139.1 (C^1^ Ar), 153.6 (C-4), 158.0 (C–6), 160.7 (C–NH). HRMS (ESI) *m/z*: calculated for C_18_H_14_BrN_4_ [M + H]^+^: 365.040182, found 365.0398 (Δ 1.05 ppm).

*2-Hydrazinyl-4-(4-methoxyphenyl)-6-phenylnicotinonitrile* **20j** was isolated in 92% yield, m.p. 141–143 °C. FTIR (nujol), ν_max_, cm^−1^: 3390, 3321 br (N–H), 2208 (C≡N). ^1^H NMR (400 MHz, DMSO-*d*_6_): 3.83 (s, 3H, MeO), 4.58 (br s, 2H, NH_2_), 7.10 (d, ^3^*J* = 8.7 Hz, 3H, H-3, and H-5, 4-MeOC_6_H_4_), 7.29 (s, 1H, H-5), 7.48–7.50 (m, 3H, H Ph), 7.64 (d, ^3^*J* = 8.7 Hz, 3H, H-2 and H-6, 4-MeOC_6_H_4_), 8.23–8.26 (m, 2H, H Ph), 8.28 (br s, 1H, NH). ^13^C NMR (101 MHz, DMSO-*d*_6_): 55.4 (MeO), 86.2 (C-3), 109.2 (C(5)H), 114.2 (2 CH Ar), 116.6 (C≡N), 127.4 (2 CH Ph), 128.6 (2 CH Ph), 128.9 (C^1^ Ar), 130.0 (2 CH Ar), 130.1 (CH Ph), 137.7 (C^1^ Ph), 154.8 (C-4), 157.7 (C–6), 160.5 (C–OMe), 161.0 (C–NH). HRMS (ESI) *m/z*: calculated for C_19_H_17_N_4_O [M + H]^+^: 317.140236, found 317.1399 (Δ 1.06 ppm).

**Synthesis of 2-(2-arylidenehydrazinyl)-4,6-diarylnicotinonitriles 21{*1–20*}. General procedure**. Into a 10 mL vial, 0.5 mmol of the corresponding hydrazine **20**, an aromatic aldehyde (0.5 mmol), and 5 mL of EtOH were placed. The reaction mixture was heated under reflux with vigorous stirring for 1–1.5 h (conversion was monitored by TLC, eluent: chloroform/toluene, 1:1). The reaction mixture was cooled, and the product was precipitated by adding cold water. The resulting precipitate of hydrazone **21** was filtered off and purified by recrystallization from an appropriate solvent (MeOH, EtOH, or 1,4-dioxane). Hydrazones **21** are fine crystalline powders, ranging from light yellow to bright yellow in color, and are soluble in acetone, 1,4-dioxane, lower alcohols, and DMSO.

*2-[2-(4-Chlorobenzylidene)hydrazinyl]-4-(4-chlorophenyl)-6-phenylnicotinonitrile* **21**{*1*} was isolated in 83% yield. FTIR (nujol), ν_max_, cm^−1^: 3265 br (N–H), 2218 (C≡N). ^1^H NMR (400 MHz, DMSO-*d*_6_): 7.47 (d, ^3^*J* = 8.7 Hz, 2H, H-3, and H-5, 4-ClC_6_H_4_), 7.50–7.55 (m, 4H, H-5, and 3H Ph overlapped), 7.63 (d, ^3^*J* = 8.7 Hz, 2H, H-3, and H-5, 4-ClC_6_H_4_), 7.73 (d, ^3^*J* = 8.7 Hz, 2H, H-2, and H-6, 4-ClC_6_H_4_), 7.82 (d, ^3^*J* = 8.7 Hz, 2H, H-2, and H-6, 4-ClC_6_H_4_), 8.14 (br s, 1H, –CH=), 8.18–8.20 (m, 2H, H Ph), 11.86 (br s, 1H, NH). ^13^C NMR (101 MHz, DMSO-*d*_6_): 89.6 (C-3), 115.8 (C(5)H), 117.4 (C≡N), 127.4 (2 CH Ar), 128.4 (2 CH Ar), 128.6 (2 CH Ar), 128.7 (2 CH Ar), 128.8 (2 CH Ar), 130.6 (CH Ph), 130.9 (2 CH Ar), 133.3, 133.6, 134.4, 136.3 (C^1^ Ar), 136.8 (C^1^ Ar), 155.8 (C-4), 158.1 (C–6), 159.9 (C–NH). Elemental Analysis: found, %: C, 67.66; H, 3.77; N, 12.60. C_25_H_16_Cl_2_N_4_ (M 443.33). Calculated, %: C, 67.73; H, 3.64; N, 12.64. HRMS (ESI) *m/z*: calculated for C_25_H_17_Cl_2_N_4_ [M + H]^+^: 443.083027, found 443.0808 (Δ 5.03 ppm).

*2-[2-(2,4-Dichlorobenzylidene)hydrazinyl]-4-(4-chlorophenyl)-6-phenylnicotinonitrile* **21**{*2*} was isolated in 80% yield. FTIR (nujol), ν_max_, cm^−1^: 3260 br (N–H), 2210 (C≡N). ^1^H NMR (400 MHz, DMSO-*d*_6_): 7.43–7.53 (m, 7H, H-5, and H Ar overlapped), 7.62–7.69 (m, 3H, H Ar), 8.18–8.23 (m, 3H, 2H Ph, H Ar), 8.50 (br s, 1H, –CH=), 12.06 (br s, 1H, NH). ^13^C NMR (101 MHz, DMSO-*d*_6_): 89.6 (C-3), 115.3 (C(5)H), 117.5 (C≡N), 127.4 (2 CH Ar), 127.8 (CH Ar), 128.0 (CH Ar), 128.4 (2 CH Ar), 128.6 (2 CH Ar), 128.8 (2CH Ar), 129.3 (CH Ar), 130.6 (C(4)H Ph), 131.3, 132.9, 134.1, 135.8, 136.4, 136.6, 136.7, 156.2 (C-4), 158.0 (C–6), 160.3 (C–NH). HRMS (ESI) *m/z*: calculated for C_18_H_14_ClN_4_ [M + H + H_2_O–ArCHO]^+^: 321.090699, found 321.0891 (Δ 4.99 ppm). Elemental Analysis: found, %: C, 62.9; H, 3.30; N, 11.63. C_25_H_15_Cl_3_N_4_ (M 477.77). Calculated, %: C, 62.85; H, 3.16; N, 11.73.

*2-[2-(4-Chlorobenzylidene)hydrazinyl]-4-(2-chlorophenyl)-6-phenylnicotinonitrile* **21**{*3*} was isolated in 89% yield. FTIR (nujol), ν_max_, cm^−1^: 3314 br (N–H), 2208 (C≡N). ^1^H NMR (400 MHz, DMSO-*d*_6_): 7.44 (d, ^3^*J* = 8.7 Hz, 2H, H-3, and H-5, 4-ClC_6_H_4_), 7.48 (s, 1H, H-5), 7.51–7.57 (m, 6H, H Ar), 7.65–7.67 (m, 1H, H Ar), 7.79 (d, ^3^*J* = 8.7 Hz, 2H, H-2, and H-6, 4-ClC_6_H_4_), 8.14 (br s, 1H, –CH=), 8.16–8.19 (m, 2H, H Ph), 11.91 (br s, 1H, NH). ^13^C NMR (101 MHz, DMSO-*d*_6_): 89.1 (C-3), 112.5 (C(5)H), 116.7 (C≡N), 127.3 (2 CH Ar), 127.4 (CH Ar), 128.4 (2 CH Ar), 128.7 (2 CH Ar), 128.8 (2 CH Ar), 129.5 (CH Ar), 130.6 (CH Ph), 130.7 (CH Ar), 130.8 (CH Ar), 131.4, 133.6, 133.9, 136.4 (C^1^ Ar), 136.7 (C^1^ Ar), 140.5, 155.5 (C-4), 156.1 (C–6), 158.0 (C–NH). HRMS (ESI) *m/z*: calculated for C_25_H_17_Cl_2_N_4_ [M + H]^+^: 443.083027, found 443.0803 (Δ 6.16 ppm).

*2-[2-(2,4-Dichlorobenzylidene)hydrazinyl]-4-(2-chlorophenyl)-6-phenylnicotinonitrile* **21**{*4*} was isolated in 80% yield. FTIR (nujol), ν_max_, cm^−1^: 3298 br (N–H), 2214 (C≡N). ^1^H NMR (400 MHz, acetone-*d*_6_): 7.35 (dd, ^3^*J* = 8.7 Hz, ^4^*J* = 1.8 Hz, 1H, H-5, 2,4-ClC_6_H_3_), 7.49 (s, 1H, H-5), 7.50–7.57 (m, 7H, H Ar), 7.62–7.64 (m, 1H, H Ar), 8.18–8.20 (m, 2H, H Ph), 8.36 (d, ^3^*J* = 8.7 Hz, 1H, H-6, 2,4-ClC_6_H_3_), 8.57 (br s, 1H, –CH=), 11.00 (br s, 1H, NH). ^13^C NMR (101 MHz, acetone-*d*_6_): 91.3 (C-3), 114.0 (C(5)H), 117.2 (C≡N), 128.1 (2 CH Ar), 128.2 (2 CH Ar), 128.5 (CH Ar), 129.5 (CH Ar), 126.6 (2 CH Ar), 130.0 (CH Ar), 130.5 (CH Ph), 131.3 (CH Ar), 131.5 (2CH Ar), 132.4, 132.9, 134.2, 135.6, 137.7, 137.9, 156.7 (C-4), 156.9 (C–6), 159.3 (C–NH). HRMS (ESI) *m/z*: calculated for C_25_H_16_Cl_3_N_4_ [M + H]^+^: 477.044055, found 477.0415 (Δ 5.36 ppm).

*2-[2-(4-Chlorobenzylidene)hydrazinyl]-4-(4-methylphenyl)-6-phenylnicotinonitrile* **21**{*5*} was isolated in 85% yield. FTIR (nujol), ν_max_, cm^−1^: 3273 br (N–H), 2208 (C≡N). ^1^H NMR (400 MHz, DMSO-*d*_6_): 2.41 (s, 3H, Me), 7.36 (d, ^3^*J* = 7.5 Hz, 2H, H Tol), 7.41–7.56 (m, 6H, H Ar, and H-5 overlapped), 7.60 (d, ^3^*J* = 7.5 Hz, 2H, H Tol), 7.82 (d, ^3^*J* = 7.8 Hz, 2H, H-2, and H-6, 4-ClC_6_H_4_), 8.14 (br s, 1H, –CH=), 8.17–8.21 (m, 2H, H Ph), 11.77 (br s, 1H, NH). HRMS (ESI) *m/z*: calculated for C_26_H_20_ClN_4_ [M + H]^+^: 423.137649, found 423.1357 (Δ 4.6 ppm).

*2-[2-(2,4-Dichlorobenzylidene)hydrazinyl]-4-(4-methylphenyl)-6-phenylnicotinonitrile* **21**{*6*} was isolated in 78% yield. FTIR (nujol), ν_max_, cm^−1^: 3312, 3292 (N–H), 2218 (C≡N). ^1^H NMR (400 MHz, DMSO-*d*_6_): 2.40 (s, 3H, Me), 7.36 (d, ^3^*J* = 7.8 Hz, 2H, H Tol), 7.39–7.46 (m, 1H, H Ar), 7.50 (s, 1H, H-5), 7.51–7.56 (m, 3H, H Ph), 7.60 (d, ^3^*J* = 7.8 Hz, 2H, H Tol), 7.67–7.69 (m, 1H, H Ar), 8.17–8.21 (m, 2H, H Ph), 8.23 (d, ^3^*J* = 8.7 Hz, 1H, H Ar), 8.51 (br s, 1H, –CH=), 12.01 (br s, 1H, NH). ^13^C NMR (101 MHz, DMSO-*d*_6_): 21.5 (Me), 86.6 (C-3), 111.5 (C(5)H), 116.3 (C≡N), 127.9 (2 CH Ar), 128.4 (CH Ar), 129.4 (2 CH Ar), 129.7 (2 CH Ar), 129.8 (CH Ar), 130.0 (2 CH Ar), 130.7 (CH Ph), 131.0 (CH Ar), 131.9, 133.4, 134.5, 134.6, 137.0, 137.4, 141.8, 153.0 (C-4), 157.1 (C–6), 158.4 (C–NH). Elemental Analysis: found, %: C, 68.33; H, 4.11; N, 12.19. C_26_H_18_Cl_2_N_4_ (M 457.36). Calculated, %: C, 68.28; H, 3.97; N, 12.25. HRMS (ESI) *m/z*: calculated for C_25_H_17_Cl_2_N_4_ [M + H–Me]^+^: 443.083027, found 443.0801 (Δ 6.6 ppm).

*2-[2-(4-Chlorobenzylidene)hydrazinyl]-4-(3,4-dimethoxyphenyl)-6-phenylnicotinonitrile* **21**{*7*} was isolated in 72% yield. FTIR (nujol), ν_max_, cm^−1^: 3277 br (N–H), 2206 (C≡N). ^1^H NMR (400 MHz, DMSO-*d*_6_): 3.84 (s, 3H, MeO), 3.86 (s, 3H, MeO), 7.12 (d, ^3^*J* = 8.7 Hz, 1H, H Ar), 7.28 (d, ^3^*J* = 8.7 Hz, 1H, H Ar), 7.30 (s, 1H, H Ar), 7.46 (d, ^3^*J* = 8.3 Hz, 2H, H Ar), 7.50–7.53 (m, 4H, H-5, H Ar overlapped), 7.82 (d, ^3^*J* = 8.3 Hz, 1H, H Ar), 8.14 (br s, 1H, –CH=), 8.18–8.21 (m, 2H, H Ph), 11.73 (br s, 1H, NH). ^13^C NMR (101 MHz, DMSO-*d*_6_): 55.6 (MeO), 55.7 (MeO), 87.5 (C-3), 111.5 (C(5)H), 112.4 (CH Ar), 112.6 (CH Ar), 117.8 (C≡N), 121.7 (C Ar), 127.3 (2 CH Ar), 128.3 (2 CH Ar), 128.7 (4 CH Ar), 129.3 (CH Ar), 130.3 (CH Ph), 133.5, 134.0, 137.1, 137.4, 140.2, 148.5 (C–OMe), 150.0 (C–OMe), 156.8 (C-4), 157.3 (C–6), 157.8 (C–NH). HRMS (ESI) *m/z*: calculated for C_27_H_22_ClN_4_O_2_ [M + H]^+^: 469.143129, found 469.1417 (Δ 3.05 ppm).

*2-[2-(2,4-Dichlorobenzylidene)hydrazinyl]-4-(3,4-dimethoxyphenyl)-6-phenylnicotinonitrile* **21**{*8*} was isolated in 73% yield. FTIR (nujol), ν_max_, cm^−1^: 3260 br (N–H), 2206 (C≡N). ^1^H NMR (400 MHz, DMSO-*d*_6_): 3.84 (s, 3H, MeO), 3.86 (s, 3H, MeO), 7.11 (d, ^3^*J* = 8.2 Hz, 1H, H Ar), 7.28 (dd, ^3^*J* = 8.2 Hz, ^4^*J* = 1.8 Hz, 1H, H Ar), 7.30 (d, ^4^*J* = 1.8 Hz, 1H, H Ar), 7.45 (dd, ^3^*J* = 8.2 Hz, ^4^*J* = 1.8 Hz, 1H, H Ar), 7.51–7.54 (m, 4H, H-5, H Ph overlapped), 7.65 (d, ^4^*J* = 1.8 Hz, 1H, H Ar), 8.17–8.20 (m, 2H, H Ph), 8.24 (d, ^3^*J* = 8.2 Hz, 1H, H Ar), 8.51 (br s, 1H, –CH=),), 11.95 (br s, 1H, NH). ^13^C NMR (101 MHz, DMSO-*d*_6_): 55.6 (MeO), 55.7 (MeO), 87.7 (C-3), 111.5 (C(5)H), 112.6 (CH Ar), 112.8 (CH Ar), 117.8 (C≡N), 121.8 (C Ar), 127.3 (2 CH Ar), 127.8 (CH Ar), 128.0 (CH Ar), 127.6 (2 CH Ar), 129.2 (CH Ar), 130.4 (CH Ph), 131.4 (CH Ar), 132.8, 134.0, 136.4, 136.9, 148.5 (C–OMe), 150.0 (C–OMe), 156.6 (C-4), 157.2 (C–6), 157.8 (C–NH). HRMS (ESI) *m/z*: calculated for C_27_H_22_ClN_4_O_2_ [M + H–Cl]^+^: 469.14314, found 469.1408 (Δ 4.99 ppm).

*2-[2-(4-Chlorobenzylidene)hydrazinyl]-4-(4-chlorophenyl)-6-(methylphenyl)nicotinonitrile* **21**{*9*} was isolated in 84% yield. FTIR (nujol), ν_max_, cm^−1^: 3269 br (N–H), 2218 (C≡N). ^1^H NMR (400 MHz, DMSO-*d*_6_): 2.39 (s, 3H, Me), 7.34 (d, ^3^*J* = 7.8 Hz, 2H, H Tol), 7.43–7.45 (m, 3H, H Ar, and H-5 overlapped), 7.55–7.58 (m, 4H, H Ar), 7.80 (d, ^3^*J* = 8.7 Hz, 2H, H Ar), 8.11 (br s, 1H, –CH=), 8.18 (d, ^3^*J* = 8.7 Hz, 2H, H Ar), 11.77 (br s, 1H, NH). ^13^C NMR (101 MHz, DMSO-*d*_6_): 20.9 (Me), 87.8 (C-3), 112.3 (C(5)H), 117.5 (C≡N), 123.27 (2 CH Ar), 128.32 (2 CH Ar), 128.7 (2 CH Ar), 128.8 (2 CH Ar), 129.0 (2 CH Ar), 129.1 (2 CH Ar), 133.5, 134.0, 134.1, 135.2, 135.7, 139.2, 140.3, 156.5 (C-4), 156.7 (C–6), 157.5 (C–NH). Elemental Analysis: found, %: C, 68.36; H, 4.09; N, 12.22. C_26_H_18_Cl_2_N_4_ (M 457.36). Calculated, %: C, 68.28; H, 3.97; N, 12.25. HRMS (ESI) *m/z*: calculated for C_26_H_19_Cl_2_N_4_ [M + H]^+^: 457.098677, found 457.0967 (Δ 4.33 ppm).

*2-[2-(2,4-Dichlorobenzylidene)hydrazinyl]-4-(4-chlorophenyl)-6-(methylphenyl)nicotinonitrile* **21**{*10*} was isolated in 78% yield. FTIR (nujol), ν_max_, cm^−1^: 3306 br (N–H), 2210 (C≡N). ^1^H NMR (400 MHz, DMSO-*d*_6_): 2.39 (s, 3H, Me), 7.30 (s, CH Ar), 7.34 (d, ^3^*J* = 7.8 Hz, 2H, H Ar), 7.42 (br d, ^3^*J* = 8.7 Hz, 1H, H-5, 2,4-Cl_2_C_6_H_3_), 7.49–7.54 (m, 4H, H Ar), 7.63 (d, ^4^*J* = 1.8 Hz, 1H, H-3, 2,4-Cl_2_C_6_H_3_), 8.18 (d, ^3^*J* = 7.8 Hz, 2H, H Ar), 8.27 (d, ^3^*J* = 8.7 Hz, 1H, H Ar), 8.48 (br s, 1H, –CH=), 11.98 (br s, 1H, NH). ^13^C NMR (101 MHz, DMSO-*d*_6_): 20.9 (Me), 86.7 (C-3), 112.7 (C(5)H), 117.5 (C≡N), 127.7 (CH Ar), 128.0 (CH Ar), 128.4 (2 CH Ar), 128.6 (2 CH Ar), 129.1 (2 CH Ar), 129.3 (2 CH Ar), 131.3 (CH Ar), 132.8, 134.0, 134.1, 135.3, 135.7, 136.5, 139.4, 156.5 (C-4), 156.4 (C-6), 159.1 (C–NH). Elemental Analysis: found, %: C, 63.38; H, 3.62; N, 11.33. C_26_H_17_Cl_3_N_4_ (M 491.80). Calculated, %: C, 63.50; H, 3.48; N, 11.39. HRMS (ESI) *m/z*: calculated for C_19_H_16_ClN_4_ [M + H + H_2_O–ArCHO]^+^: 335.106349, found 335.1046 (Δ 5.2 ppm).

*2-[2-(4-Chlorobenzylidene)hydrazinyl]-4-(4-fluorophenyl)-6-phenylnicotinonitrile* **21**{*11*} was isolated in 85% yield as solvate with EtOH (1:1). FTIR (nujol), ν_max_, cm^−1^: 3479 br (OH of EtOH), 3323 br (N–H), 2208 (C≡N). ^1^H NMR (400 MHz, DMSO-*d*_6_): 1.04 (t, ^3^*J* = 7.1 Hz, 3H, C**H**_3_CH_2_OH), 3.40–3.46 (m, 2H, CH_3_C**H**_2_OH), 4.35 (t, ^3^*J* = 5.0 Hz, 3H, CH_3_CH_2_O**H**), 7.38–7.42 (m, 2H, H Ar), 7.46 (d, ^3^*J* = 8.5 Hz, 2H, H Ar), 7.50 (s, 1H, H-5), 7.51–7.53 (m, 3H, H Ph), 7.75–7.78 (m, 2H, H Ar), 7.82 (d, ^3^*J* = 8.5 Hz, 2H, H Ar), 8.14 (br s, 1H, –CH=), 8.17–8.20 (m, 2H, H Ph), 11.81 (br s, 1H, NH). ^13^C NMR (101 MHz, DMSO-*d*_6_): 18.6 (**C**H_3_CH_2_OH), 56.0 (CH_3_**C**H_2_OH), 87.6 (C-3), 112.5 (C(5)H), 115.5 (d, ^2^*J*_C–F_ = 21.1 Hz, C(3)H C(5)H, 4-FC_6_H_4_), 117.5 (C≡N), 127.4 (2 CH Ph), 128.4 (2 CH Ph), 128.8 (4 CH, 4-ClC_6_H_4_), 130.5 (C(4)H Ph), 131.3 (d, ^3^*J*_C–F_ = 8.6 Hz, C(2)H C(6)H, 4-FC_6_H_4_), 133.5 (d, ^4^*J*_C–F_ = 3.8 Hz, C^1^ 4-FC_6_H_4_), 133.6, 134.0, 136.9, 140.4, 156.5 (C-4), 156.7 (C–6), 158.0 (C–NH), 161.8, 164.2 (d, ^1^*J*_C–F_ = 239 Hz, C–F). HRMS (ESI) *m/z*: calculated for C_25_H_17_ClFN_4_ [M + H]^+^: 427.112577, found 427.1131 (Δ −1.23 ppm); calculated for C_18_H_14_FN_4_ [M + H + H_2_O–ArCHO]^+^: 305.120249, found 305.1203 (Δ −0.17 ppm).

*2-[2-(2,4-Dichlorobenzylidene)hydrazinyl]-4-(4-fluorophenyl)-6-phenylnicotinonitrile* **21**{*12*} was isolated in 81% yield as solvate with EtOH (1:1). FTIR (nujol), ν_max_, cm^−1^: 3458 br (OH of EtOH), 3327 br (N–H), 2206 (C≡N). ^1^H NMR (400 MHz, DMSO-*d*_6_): 1.04 (t, ^3^*J* = 7.1 Hz, 3H, C**H**_3_CH_2_OH), 3.40–3.46 (m, 2H, CH_3_C**H**_2_OH), 4.36 (t, ^3^*J* = 5.0 Hz, 3H, CH_3_CH_2_O**H**), 7.37–7.44 (m, 3H, H Ar), 7.51–7.52 (m, 4H, H Ph, and H-5 overlapped), 7.64 (br s, 1H, H-3, 2,4-Cl_2_C_6_H_3_), 7.74–7.78 (m, 2H, H Ar), 8.17–8.19 (m, 2H, H Ph), 8.22 (d, ^3^*J* = 8.7 Hz, 1H, H-6, 2,4-Cl_2_C_6_H_3_), 8.50 (br s, 1H, –CH=), 12.03 (br s, 1H, NH). ^13^C NMR (101 MHz, DMSO-*d*_6_): 18.6 (**C**H_3_CH_2_OH), 56.0 (CH_3_**C**H_2_OH), 87.9 (C-3), 112.9 (C(5)H), 115.5 (d, ^2^*J*_C–F_ = 22.1 Hz, C(3)H C(5)H, 4-FC_6_H_4_), 117.6 (C≡N), 127.4 (2 CH Ph), 127.8 (CH Ar), 128.0 (CH Ar), 128.8 (2 CH Ph), 129.2 (CH Ar), 130.6 (C(4)H Ph), 131.3 (d, ^3^*J*_C–F_ = 8.6 Hz, C(2)H C(6)H, 4-FC_6_H_4_), 132.9 (C Ar), 133.4 (d, ^4^*J*_C–F_ = 2.9 Hz, C^1^ 4-FC_6_H_4_), 134.1, 136.6, 136.8, 143.0, 156.4 (C-4), 156.5 (C–6), 158.0 (C–NH), 161.7, 164.2 (d, ^1^*J*_C–F_ = 247.3 Hz, C–F). HRMS (ESI) *m/z*: calculated for C_18_H_14_FN_4_ [M + H + H_2_O–ArCHO]^+^: 305.120249, found 305.1185 (Δ 5.73 ppm).

*2-[2-(4-Chlorobenzylidene)hydrazinyl]-4-(2,4-dichlorophenyl)-6-phenylnicotinonitrile* **21**{*13*} was isolated in 74% yield. FTIR (nujol), ν_max_, cm^−1^: 3304 (N–H), 2218 (C≡N). ^1^H NMR (400 MHz, DMSO-*d*_6_): 7.44 (d, ^3^*J* = 8.5 Hz, 2H, H Ar), 7.50–7.54 (m, 4H, H Ph, and H-5 overlapped), 7.58–7.64 (m, 2H, H Ar), 7.79 (d, ^3^*J* = 8.5 Hz, 2H, H Ar), 7.87 (d, ^4^*J* = 1.4 Hz, 1H, H Ar), 8.13 (s, 1H, –CH=), 8.15–8.18 (m, 2H, H Ph), 11.97 (br s, 1H, NH). ^13^C NMR (101 MHz, DMSO-*d*_6_): 88.9 (C-3), 112.5 (C(5)H), 116.8 (C≡N), 127.3 (2 CH Ar), 127.7 (CH Ar), 128.4 (2 CH Ar), 128.8 (2 CH Ar), 128.9 (2 CH Ar), 129.1 (CH Ar), 130.7 (C(4)H Ph), 132.1 (CH Ar), 132.7 (CH Ar), 133.7, 133.9, 134.7, 135.4, 136.6, 140.7, 154.5 (C-4), 156.1 (C-6), 158.2 (C–NH). HRMS (ESI) *m/z*: calculated for C_25_H_16_Cl_3_N_4_ [M + H]^+^: 479.04111, found 477.0390 (Δ 4.4 ppm);

*2-[2-(2,4-Dichlorobenzylidene)hydrazinyl]-4-(2,4-dichlorophenyl)-6-phenylnicotinonitrile* **21**{*14*} was isolated as solvate with 1,4-dioxane (1:1) in 81% yield. FTIR (nujol), ν_max_, cm^−1^: 3178 (N–H), 2216 (C≡N). ^1^H NMR (400 MHz, DMSO-*d*_6_): 3.55 (br s, 8H, dioxane), 7.42 (dd, ^3^*J* = 8.7 Hz, ^4^*J* = 1.8 Hz, 1H, H-5, 2,4-Cl_2_C_6_H_3_), 7.52–7.54 (m, 3H, H Ph), 7.56 (br s, 1H, H-5), 7.60 (d, ^3^*J* = 8.2 Hz, 1H, H-6, 2,4-Cl_2_C_6_H_3_), 7.63 (dd, ^3^*J* = 8.2 Hz, ^4^*J* = 1.8 Hz, 1H, H-5, 2,4-Cl_2_C_6_H_3_), 7.67 (d, ^4^*J* = 1.8 Hz, 1H, H-3, 2,4-Cl_2_C_6_H_3_), 7.87 (d, ^4^*J* = 1.8 Hz, 1H, H-3, 2,4-Cl_2_C_6_H_3_), 8.16–8.18 (m, 3H, 2 H Ph, and H-6 2,4-Cl_2_C_6_H_3_ overlapped), 8.52 (br s, 1H, –CH=), 12.16 (br s, 1H, NH). ^13^C NMR (101 MHz, DMSO-*d*_6_): 66.3 (OCH_2_ dioxane), 89.2 (C-3), 112.9 (C(5)H), 116.7 (C≡N), 127.3 (2 CH Ar), 127.69 (CH Ar), 127.74 (CH Ar), 127.9 (CH Ar), 128.9 (2 CH Ar), 129.1 (CH Ar), 129.2 (CH Ar), 130.7 (C(4)H Ph), 131.2 (CH Ar), 132.1 (CH), 132.7, 132.9, 134.2, 134.7, 135.3, 136.5, 136.9, 154.4 (C-4), 155.9 (C-6), 158.2 (C–NH). Elemental Analysis: found, %: C, 58.11; H, 3.64; N, 9.30. C_25_H_14_Cl_4_N_4_ × C_4_H_8_O_2_ (M 600.32). Calculated, %: C, 58.02; H, 3.69; N, 9.33. HRMS (ESI) *m/z*: calculated for C_18_H_13_Cl_2_N_4_ [M + H + H_2_O–ArCHO]^+^: 355.051727, found 355.0501 (Δ 4.58 ppm).

*2-[2-(4-Chlorobenzylidene)hydrazinyl]-4-(3-nitrophenyl)-6-phenylnicotinonitrile* **21**{*15*} was isolated in 75% yield. FTIR (nujol), ν_max_, cm^−1^: 3308 br (N–H), 2208 (C≡N), 1531 (NO_2 asymm_), 1356 (NO_2 symm_). ^1^H NMR (400 MHz, acetone-*d*_6_): 7.43 (d, ^3^*J* = 8.2 Hz, 2H, H Ar), 7.49–7.54 (m, 3H, H Ar), 7.64 (s, 1H, H Ar), 7.87–7.92 (m, 3H, H Ar), 8.17–8.21 (m, 3H, 2 H Ph, and H Ar overlapped), 8.43 (dd, ^3^*J* = 8.1 Hz, ^4^*J* = 1.8 Hz, 1H, H Ar), 8.58–8.59 (m, 1H, H-2 3-NO_2_C_6_H_4_), 8.66 (br s, 1H, –CH=), 10.79 (br s, 1H, NH). ^13^C NMR (101 MHz, acetone-*d*_6_): 89.2 (C-3), 113.5 (C(5)H), 117.7 (C≡N), 124.7 (CH Ar), 124.9 (CH Ar), 128.3 (2 CH Ar), 129.5 (2 CH Ar), 129.6 (4 CH Ar), 129.9 (CH Ar), 130.8 (C(4)H Ph), 130.9 (CH Ar), 131.4 (CH Ar), 135.1, 135.2, 136.2, 138.0, 141.6, 149.1 (C–NO_2_), 156.3 (C-4), 159.6 (C-6), 161.5 (C–NH). Elemental Analysis: found, %: C, 66.08; H, 3.64; N, 15.33. C_25_H_16_ClN_5_O_2_ (M 453.89). Calculated, %: C, 66.16; H, 3.55; N, 15.43. HRMS (ESI) *m/z*: calculated for C_18_H_12_N_3_O_3_ [M + H + H_2_O–ArCH = N-NH_2_]^+^: 318.087867, found 318.0856 (Δ 7.1 ppm); calculated for C_36_H_23_N_6_O_6_ [2M + H + 2H_2_O–2ArCH = N-NH_2_]^+^: 635.167909, found 635.1648 (Δ 4.9 ppm); calculated for C_36_H_22_N_6_O_6_Na [2M + Na + 2H_2_O–2ArCH = N-NH_2_]^+^: 657.149854, found 657.1463 (Δ 5.4 ppm).

*2-[2-(2,4-Dichlorobenzylidene)hydrazinyl]-4-(3-nitrophenyl)-6-phenylnicotinonitrile* **21**{*16*} was isolated in 76% yield. FTIR (nujol), ν_max_, cm^−1^: 3294 br (N–H), 2210 (C≡N), 1526 (NO_2 asymm_), 1350 (NO_2 symm_). ^1^H NMR (400 MHz, DMSO-*d*_6_): 7.44 (d, ^3^*J* = 8.7 Hz, 1H, H Ar), 7.50–7.56 (m, 4H, H Ar, and H-5 overlapped), 7.68 (br s, 1H, H Ar), 7.83–7.88 (m, 2H, H Ar), 8.17–8.21 (m, 3H, 2 H Ph, and H Ar overlapped), 8.41 (d, ^3^*J* = 8.2 Hz, 1H, H Ar), 8.53–8.55 (m, 2H, H-Ar, and –CH= overlapped), 12.13 (br s, 1H, NH). Elemental Analysis: found, %: C, 61.40; H, 3.24; N, 14.28. C_25_H_15_Cl_2_N_5_O_2_ (M 488.33). Calculated, %: C, 61.49; H, 3.10; N, 14.34. HRMS (ESI) *m/z*: calculated for C_18_H_12_N_3_O_3_ [M + H + H_2_O–ArCH = N-NH_2_]^+^: 318.087867, found 318.0854 (Δ 7.75 ppm); calculated for C_36_H_23_N_6_O_6_ [2M + H + 2H_2_O–2ArCH = N-NH_2_]^+^: 635.167909, found 635.1647 (Δ 5.05 ppm).

*4-(3-Bromophenyl)-2-[2-(4-chlorobenzylidene)hydrazinyl]-6-phenylnicotinonitrile* **21**{*17*} was isolated in 70% yield. FTIR (nujol), ν_max_, cm^−1^: 3300 br (N–H), 2214 (C≡N). ^1^H NMR (400 MHz, DMSO-*d*_6_): 7.38–7.42 (m, 1H, H Ar), 7.46 (d, ^3^*J* = 8.5 Hz, 2H, H Ar), 7.51–7.55 (m, 4H, H Ph, and H-5 overlapped), 7.69–7.76 (m, 2H, H Ar), 7.82 (d, ^3^*J* = 8.5 Hz, 2H, H Ar), 7.89–7.90 (m, 1H, H Ar), 8.14 (br s, 1H, –CH=), 8.19–8.21 (m, 2H, H Ph), 11.84 (br s, 1H, NH). ^13^C NMR (101 MHz, DMSO-*d*_6_): 87.4 (C-3), 112.4 (C(5)H), 117.4 (C≡N), 121.7 (C–Br), 127.4 (2 CH Ar), 128.1 (2 CH Ar), 128.4 (2 CH Ar), 128.7 (2 CH Ar), 129.1 (CH Ar), 130.0 (CH Ar), 130.6 (C(4)H Ph), 131.4 (CH Ar), 132.2 (CH Ar), 133.6, 133.9, 136.8, 139.3, 140.4, 155.8 (C-4), 156.6 (C-6), 158.1 (C–NH). Elemental Analysis: found, %: C, 61.43; H, 3.44; N, 11.38. C_25_H_16_BrClN_4_ (M 487.79). Calculated, %: C, 61.56; H, 3.31; N, 11.49. HRMS (ESI) *m/z*: calculated for C_18_H_14_BrN_4_ [M + H + H_2_O–ArCHO]^+^: 365.040182, found 365.0398 (Δ 7.07 ppm); calculated for C_25_H_17_BrClN_4_ [M + H]^+^: 489.03047, found 489.0272 (Δ 6.69 ppm);

*4-(3-Bromophenyl)-2-[2-(2,4-dichlorobenzylidene)hydrazinyl]-6-phenylnicotinonitrile* **21**{*18*} was isolated in 73% yield. FTIR (nujol), ν_max_, cm^−1^: 3234 br (N–H), 2218 (C≡N). 7.39–7.43 (m, 2H, H Ar), 7.51–7.55 (m, 4H, H Ph, and H-5 overlapped), 7.63 (d, ^4^*J* = 1.8 Hz, 1H, H Ar), 7.70–7.75 (m, 2H, H Ar), 7.88–7.90 (m, 1H, H Ar), 8.18–8.23 (m, 3H, H Ph, H Ar), 8.51 (br s, 1H, –CH=), 12.06 (br s, 1H, NH). ^13^C NMR (101 MHz, DMSO-*d*_6_): 87.7 (C-3), 112.8 (C(5)H), 116.7 (C≡N), 121.7 (C–Br), 127.4 (2 CH Ph), 127.8 (CH Ar), 128.0 (CH Ar), 128.8 (2 CH Ph), 129.1 (CH Ar), 129.2 (CH Ar), 130.0 (CH Ar), 130.6 (C(4)H Ph), 131.7 (CH Ar), 132.3 (CH Ar), 128.1 (2 CH Ar), 128.4 (2 CH Ar), 128.7 (2 CH Ar), 129.1 (CH Ar), 130.0 (CH Ar), 130.6 (C(4)H Ph), 131.4 (CH Ar), 132.2 (CH Ar), 132.8, 133.9, 134.1, 136.7, 139.2, 143.6, 155.8 (C-4), 156.8 (C-6), 158.1 (C–NH). Elemental Analysis: found, %: C, 57.57; H, 3.06; N, 10.60. C_25_H_15_BrCl_2_N_4_ (M 522.23). Calculated, %: C, 57.50; H, 2.90; N, 10.73. HRMS (ESI) *m/z*: calculated for C_25_H_17_Cl_2_N_4_ [M + H–Br]^+^: 443.083027, found 443.0804 (Δ 5.9 ppm); calculated for C_18_H_14_BrN_4_ [M + H + H_2_O–ArCHO]^+^: 365.040182, found 365.0373 (Δ 7.9 ppm).

*2-[2-(4-Chlorobenzylidene)hydrazinyl]-4-(4-methoxyphenyl)-6-phenylnicotinonitrile* **21**{*19*} was isolated in 75% yield. FTIR (nujol), ν_max_, cm^−1^: 3269 br (N–H), 2218 (C≡N). ^1^H NMR (400 MHz, DMSO-*d*_6_): 3.84 (s, 3H, MeO), 7.10 (d, ^3^*J* = 8.7 Hz, 2H, H-3 H-5 4-MeOC_6_H_4_), 7.44–7.47 (m, 3H, H Ar, and H-5 overlapped), 7.50–7.53 (m, 3H, H Ph), 7.67 (d, ^3^*J* = 8.7 Hz, 2H, H-Ar), 7.82 (d, ^3^*J* = 8.7 Hz, 2H, H-Ar), 8.13 (br s, 1H, –CH=), 8.16–8.18 (m, 2H, H Ph), 11.73 (br s, 1H, NH). ^13^C NMR (101 MHz, DMSO-*d*_6_): 55.3 (MeO), 87.4 (C-3), 112.3 (C(5)H), 113.9 (2 CH Ar), 117.8 (C≡N), 127.3 (2 CH Ph), 128.3 (2 CH Ar), 128.8 (4 CH Ar), 129.2 (CH), 130.3 (C(4)H Ph), 130.4 (2 CH Ar), 133.5, 134.1, 137.0, 140.1, 156.9 (C-4), 157.1 (C–OMe), 157.8 (C-6), 160.4 (C–NH). Elemental Analysis: found, %: C, 71.08; H, 4.42; N, 12.75. C_26_H_19_ClN_4_O (M 438.92). Calculated, %: C, 71.15; H, 4.36; N, 12.77. HRMS (ESI) *m/z*: calculated for C_26_H_20_ClN_4_O [M + H]^+^: 439.132564, found 439.1297 (Δ 6.5 ppm).

*2-[2-(2,4-Dichlorobenzylidene)hydrazinyl]-4-(4-methoxyphenyl)-6-phenylnicotinonitrile* **21**{*20*} was isolated in 73% yield. FTIR (nujol), ν_max_, cm^−1^: 3287 br (N–H), 2208 (C≡N). ^1^H NMR (400 MHz, DMSO-*d*_6_): 3.84 (s, 3H, MeO), 7.10 (d, ^3^*J* = 8.8 Hz, 2H, H-3 H-5 4-MeOC_6_H_4_), 7.42–7.44 (m, 1H, H Ar), 7.49–7.51 (m, 4H, 3 H Ph, and H-5 overlapped), 7.64–7.68 (m, 3H, H Ar), 8.15–8.17 (m, 2H, H Ph), 8.22–8.25 (m, 1H, H Ar), 8.49 (br s, 1H, –CH=), 11.97 (br s, 1H, NH). ^13^C NMR (101 MHz, DMSO-*d*_6_): 55.4 (MeO), 87.7 (C-3), 112.7 (C(5)H), 114.0 (2 CH Ar), 117.9 (C≡N), 127.4 (2 CH Ph), 127.8 (CH Ar), 128.0 (CH Ar), 128.8 (2 CH Ph), 129.1 (CH), 129.2 (CH Ar), 130.4 (C(4)H Ph), 130.5 (2 CH Ar), 131.4, 132.8, 134.0, 136.3, 136.9, 156.7 (C-4), 157.0 (C–OMe), 157.8 (C-6), 160.4 (C–NH). Elemental Analysis: found, %: C, 65.94; H, 3.97; N, 11.77. C_26_H_18_Cl_2_N_4_O (M 473.36). Calculated, %: C, 65.97; H, 3.83; N, 11.84. HRMS (ESI) *m/z*: calculated for C_25_H_17_Cl_2_N_4_ [M + H–MeO]^+^: 443.083027, found 443.0809 (Δ 4.8 ppm).

*X-ray studies of crystals of 2-bromo-4-(4-chlorophenyl)-6-phenylnicotinonitrile (**15******a****)*.

Single crystals of 2-bromo-4-(4-chlorophenyl)-6-phenylnicotinonitrile (**15a**) were isolated from mother liquor (AcOH solution). A suitable crystal was selected and studied on a SuperNova, Dual, Cu at home/near, AtlasS2 diffractometer. The crystal was kept at 100.00 (11) K during data collection. Using Olex2 [165], the structure was solved using the SHELXT [166] structure solution program using Intrinsic Phasing and then refined with the SHELXL [167] refinement package using Least Squares minimization. The crystals of **15a** (C_18_H_10_BrClN_2_, M =369.64 g/mol) were monoclinic, space group P2_1_/c (no. 14), *a* = 15.2876(4) Å, *b* = 7.21330(10) Å, *c* = 15.3758(4) Å, *β* = 118.885(3)°, V = 1484.61(7) Å^3^, Z = 4, T = 100.00(11) K, μ(Cu Kα) = 5.392 mm^−1^, and *D*_calc_ = 1.654 g/cm^3^, and 10,591 reflections were measured (6.604° ≤ 2Θ ≤ 152.484°), of which 3098 were unique (*R*_int_ = 0.0382, *R*_sigma_ = 0.0316), which were used in all calculations. The final *R*_1_ was 0.0323 (*I* > 2σ(I)), and *wR*_2_ was 0.0865 (all data). A full set of crystallographic data has been deposited in the Cambridge Crystallographic Data Center (CCDC Deposition Number 2499675).

X-ray studies of co-crystallized (2S*,3R*,4S*,6R*)-3-benzoyl-5-bromo-4- hydroxy-4-phenyl-2,6-di-p-tolylcyclohexane-1,1-dicarbonitrile **17-Br** and (2S*,3R*,4S*,6R*)- 3-benzoyl-4-hydroxy-4-phenyl-2,6-di-p-tolylcyclohexane-1,1-dicarbonitrile **17**, solvate with AcOH.

Single crystals of C_39_H_37.39_Br_0.61_N_2_O_6_ (**17** + **17-Br**) were crystallized by slow evaporation of filtrate (glac. AcOH) that remained after the separation of 2-bromonicotinonitrile **15c**. A suitable crystal was selected and studied on a SuperNova, Dual, Cu at home/near, AtlasS2 diffractometer. The crystal was kept at 293(2) K during data collection. Using Olex2 [165], the structure was solved with the SHELXT [166] structure solution program using Intrinsic Phasing and refined with the SHELXL [167] refinement package using Least Squares minimization. The co-crystals **17** + **17-Br** (C_39_H_37.39_Br_0.61_N_2_O_6_, *M* = 678.84 g/mol) were triclinic, space group P-1 (no. 2), *a* = 11.7101(2) Å, *b* = 12.1777(2) Å, *c* = 13.1548(3) Å, *α* = 105.5935(16)°, *β* = 95.2886(16)°, *γ* = 91.2975(15)°, *V* = 1796.97(6) Å^3^, *Z* = 2, *T* = 293(2) K, μ(Cu Kα) = 1.436 mm^−1^, and *Dcalc* = 1.255 g/cm^3^, and 26,097 reflections were measured (7.014° ≤ 2Θ ≤ 152.508°), of which 7469 were unique (*R*_int_ = 0.0156, R_sigma_ = 0.0143), which were used in all calculations. The final *R*_1_ was 0.0527 (*I* > 2σ(I)), and *wR*_2_ was 0.1366 (all data). A full set of crystallographic data has been deposited in the Cambridge Crystallographic Data Center (CCDC Deposition Number 2499676).

### Herbicide-Safening Effect Studies

The evaluation of the antidote effect was performed on sunflower seedlings (cv. Master) according to the reported procedure [159,160,161,162] as follows. Sunflower seedlings having 2–4 mm long embryo roots were treated with a solution of 2,4-D (10^−3^% by weight) for 1 h to achieve 40–60% inhibition of hypocotyl growth. Following exposure to the herbicide, the germinated seeds were rinsed with distilled water and transferred to a solution containing either compound **20** or **21** (applied at weight concentrations of 10^−2^, 10^−3^, 10^−4^, or 10^−5^% representing the “herbicide + antidote” test series). After one hour of immersion, the seedlings were again rinsed with distilled water and positioned on paper strips (10 × 75 cm), with 20 seeds per strip. The strips were subsequently rolled and placed into beakers containing 50 cm^3^ of distilled water. A reference group, designated for the “herbicide-only” series, was treated with a 2,4-D solution (10^−3^%) for one hour, followed by a one-hour immersion in water. Seeds for the “control” series were maintained in water for the entire two-hour period. All solutions were kept at a constant temperature of 28 °C. Subsequently, the samples were transferred to a thermostat for a 72 h incubation period at 28 °C. Each experimental variant was carried out in triplicate, with 20 seeds per replication. The obtained data are presented in Table 1.

## 4. Conclusions

In summary, we have developed a method for the synthesis of new 2-hydrazinylnicotinonitriles, prepared a series of hydrazones, and investigated their biological activity. A key advantage of this new synthetic approach is the use of 4,6-diaryl-2-bromo-3-cyanopyridines in the reaction with hydrazine, instead of the less reactive 2-chloro-, 2-alkoxy-, 2-mercapto-, or 2-(alkylthio)nicotinonitriles. This allows the reaction with N_2_H_4_ to proceed under milder conditions without heating to give products in nearly quantitative yields. Further benefits include the absence of the side reaction leading to 3-aminopyrazolo[3,4-*b*]pyridines. The starting 4,6-diaryl-2-bromo-3-cyanopyridines are readily available and can be prepared in high yield in two steps starting from cheap acyclic precursors. First, malononitrile and 1,3-diarylpropenones (chalcones) were reacted to afford intermediate Michael adducts. The latter were brominated with Br_2_ in AcOH to give 2-bromonicotinonitriles. During an attempt to isolate additional crops of bromopyridine from the mother liquor, we isolated and characterized by X-ray diffraction a crystalline by-product resulting from a tandem bromination–carbocyclization of a Michael *bis*-adduct. This compound was identified as a previously unreported derivative of 2,6-diaryl-3-benzoyl-5-bromo-4-hydroxy-4-phenylcyclohexane-1,1-dicarbonitrile.

To explore the agrochemical potential, a small library of hydrazones was synthesized by reacting the new 2-hydrazinylnicotinonitriles with chlorine-substituted aromatic aldehydes. Laboratory experiments on sunflower seedlings revealed that four samples from the series of 2-hydrazinylnicotinonitriles and four samples of arylhydrazones exhibit a significant herbicide safening effect against 2,4-D.

The most active compounds were 4-(4-chlorophenyl)-2-hydrazinyl- 6-phenylnicotinonitrile **20a**, which reduced the negative effect of the 2,4-D herbicide by 34–86% on roots and by 30–46% on hypocotyls and 4-(3-bromophenyl)-2-[2-(4-chlorobenzylidene)hydrazinyl]-6-phenylnicotinonitrile **21**{*17*}, which reduced the negative effect on sunflower seedling hypocotyls by 38–52% and on roots by 61–86%.

## Figures and Tables

**Table 1:** Protective effects of the most active compounds **20**,**21** against 2,4-D herbicide.

N	Pyridine	Plant Organ	Antidote Effect (*A*) at Different Concentrations, % ^1^			
			10^−2^	10^−3^	10^−4^	10^−5^
1	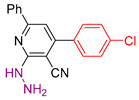 **20a**	roots	141	134	178	186
		hypocotyls	135	134	146	130
2	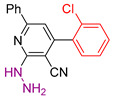 **20b**	roots	132	113	116	136
		hypocotyls	140	97	93	96
3	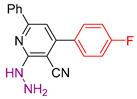 **20f**	roots	130	93	140	134
		hypocotyls	111	73	102	98
4	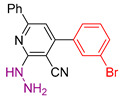 **20i**	roots	170	130	180	138
		hypocotyls	104	117	92	88
5	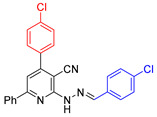 **21**{*1*}	roots	154	154	150	184
		hypocotyls	123	112	101	132
6	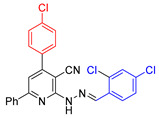 **21**{*2*}	roots	163	173	167	152
		hypocotyls	156	119	119	122
7	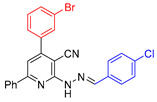 **21**{*17*}	roots	186	171	168	161
		hypocotyls	148	152	138	145
8	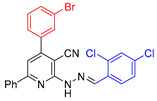 **21**{*18*}	roots	188	197	180	159
		hypocotyls	146	152	127	137

^1^ The differences are reliable at *p* = 0.95.

## Data Availability

The original contributions presented in this study are included in the article/Supplementary Materials. Further inquiries can be directed to the corresponding author.

## Supplementary Materials

The following supporting information can be downloaded at https://www.mdpi.com/article/10.3390/ijms262411874/s1.
