# Supplementary material for: Synthesis, Reactions, and Agrochemical Studies of New 4,6-Diaryl-2-hydrazinylnicotinonitriles
Source: Int J Mol Sci. 2025 Dec 9;26(24):11874. doi: 10.3390/ijms262411874 (PMC12733256; doi:10.3390/ijms262411874)
Supplement: Supplementary file 1 [file ijms-26-11874-s001.zip › ijms-4015745-supplementary.pdf]

Electronic Supplementary Material file

Synthesis, reactions and agrochemical studies of new 4,6-diaryl-2-hydrazinonicotinonitriles

Victor V. Dotsenko[1,2,*], Vladislav K. Kindop[1], Vyacheslav K. Kindop[1], Renat G. Achmiz[1], Arina G. Levchenko[1], Polina G. Dakhno[1], Azamat Z. Temerdashev[3], Yu-Qi Feng[4], Quan-Fei Zhu[4], Eva S. Daus[1], Igor V. Yudaev[5], Yuliia V. Daus [5], Alexander A. Aksenov[2], Nicolai A. Aksenov[2] and Inna V. Aksenova[2]

[1]Department of Organic Chemistry and Technologies, Kuban State University, 149 Stavropolskaya St., 350040 Krasnodar, Russia; victor_dotsenko_@mail.ru (V.V.D.); vlad.kindop@mail.ru (Vl.K.K.); slavakindop@mail.ru (V.K.K.); renat989898@gmail.com (A.R.G.); levchenko.arin@yandex.ru (L.A.G.); p.dahno@yandex.ru (D.P.G.); eva.daus.2008@gmail.com (E.S.D.).
[2]Department of Chemistry, North Caucasus Federal University, 1a Pushkin St., 355017 Stavropol, Russia; aaksenov@ncfu.ru (A.A.A.), radioanimation@rambler.ru (N.A.A.); inna-aksenova00@rambler.ru (I.V.A.)
[3]Department of Analytical Chemistry, Kuban State University, 149 Stavropolskaya St., 350040 Krasnodar, Rus-sia; temerdashevaz@gmail.com (A.Z.T.)
[4]School of Bioengineering and Health, Wuhan Textile University, 1 Sunshine Avenue, Jiang Xia District, 430200 Wuhan, Hubei, China, yqfeng@whu.edu.cn (Y.Q.F.); qf_zhu@whu.edu.cn (Q.F.Z.)
[5]Faculty of Energetics, Kuban State Agrarian University, 13 Kalinina St., 350044 Krasnodar, Russia; etsh1965@mail.ru (I.V.Y.); zirochka2505@gmail.com (Y.V.D.)
*****Correspondence: victor_dotsenko_@mail.ru (V.V.D.).

Table of contents (selected spectral and X-ray data)

Figure S1. FTIR spectrum (nujol) of 2-bromo-3-cyanopyridine 15a 5
Figure S2. ^{1}H NMR spectrum of 2-bromo-3-cyanopyridine 15a, DMSO-d_6 (400 MHz) 5
Figure S3. FTIR spectrum (KBr) of 2-bromo-3-cyanopyridine 15b 6
Figure S4. FTIR spectrum (nujol) of 2-bromo-3-cyanopyridine 15c 6
Figure S5. ^{1}H NMR spectrum of 2-bromo-3-cyanopyridine 15c, DMSO-d_6 (400 MHz) 7
Figure S6. ^{13}C NMR DEPTQ spectrum of 2-bromo-3-cyanopyridine 15c, DMSO-d_6 (101 MHz) 7
Figure S7. FTIR spectrum (KBr) of 2-bromo-3-cyanopyridine 15d 8
Figure S8. FTIR spectrum (KBr) of 2-bromo-3-cyanopyridine 15e 8
Figure S9. FTIR spectrum (KBr) of 2-bromo-3-cyanopyridine 15f 9
Figure S10. FTIR spectrum (nujol) of 2-hydrazino-3-cyanopyridine 20a 9
Figure S11. ^{1}H NMR spectrum of 2-hydrazino-3-cyanopyridine 20a, DMSO-d_6 (400 MHz) 10
Figure S12. ^{13}C DEPTQ NMR spectrum of 2-hydrazino-3-cyanopyridine 20a, DMSO-d_6 (101 MHz) 10
Figure S13. HRMS spectrum of 2-hydrazino-3-cyanopyridine 20a 11
Figure S14. FTIR spectrum (nujol) of 2-hydrazino-3-cyanopyridine 20b 11
Figure S15. ^{1}H NMR spectrum of 2-hydrazino-3-cyanopyridine 20b, DMSO-d_6 (400 MHz) 12
Figure S16. ^{13}C NMR spectrum of 2-hydrazino-3-cyanopyridine 20b, DMSO-d_6 (101 MHz) 12
Figure S17. HRMS spectrum of 2-hydrazino-3-cyanopyridine 20b 13
Figure S18. FTIR spectrum (nujol) of 2-hydrazino-3-cyanopyridine 20c 13
Figure S19. ^{1}H NMR spectrum of 2-hydrazino-3-cyanopyridine 20c, DMSO-d_6 (400 MHz) 14
Figure S20. ^{13}C NMR spectrum of 2-hydrazino-3-cyanopyridine 20c, DMSO-d_6 (101 MHz) 14
Figure S21. FTIR spectrum (nujol) of 2-hydrazino-3-cyanopyridine 20d 15

Figure S22. ^{1}H NMR spectrum of 2-hydrazino-3-cyanopyridine 20d, DMSO-d_6 (400 MHz)15
Figure S23. ^{13}C NMR spectrum of 2-hydrazino-3-cyanopyridine 20d, DMSO-d_6 (101 MHz)16
Figure S24. HRMS spectrum of 2-hydrazino-3-cyanopyridine 20d........16
Figure S25. FTIR spectrum (nujol) of 2-hydrazino-3-cyanopyridine 20e........17
Figure S26. ^{1}H NMR spectrum of 2-hydrazino-3-cyanopyridine 20e, DMSO-d_6 (400 MHz)17
Figure S27. ^{13}C NMR spectrum of 2-hydrazino-3-cyanopyridine 20e, DMSO-d_6 (101 MHz)18
Figure S28. HRMS spectrum of 2-hydrazino-3-cyanopyridine 20e........18
Figure S29. FTIR spectrum (nujol) of 2-hydrazino-3-cyanopyridine 20f........19
Figure S30. ^{1}H NMR spectrum of 2-hydrazino-3-cyanopyridine 20f, DMSO-d_6 (400 MHz) 19
Figure S31. ^{13}C NMR spectrum of 2-hydrazino-3-cyanopyridine 20f, DMSO-d_6 (101 MHz)20
Figure S32. HRMS spectrum of 2-hydrazino-3-cyanopyridine 20f........20
Figure S33. FTIR spectrum (nujol) of 2-hydrazino-3-cyanopyridine 20g........21
Figure S34. ^{1}H NMR spectrum of 2-hydrazino-3-cyanopyridine 20g, DMSO-d_6 (400 MHz)21
Figure S35. ^{13}C NMR spectrum of 2-hydrazino-3-cyanopyridine 20g, DMSO-d_6 (101 MHz)22
Figure S36. HRMS spectrum of 2-hydrazino-3-cyanopyridine 20g........22
Figure S37. HRMS spectrum of 2-hydrazino-3-cyanopyridine 20h........22
Figure S38. FTIR spectrum (nujol) of 2-hydrazino-3-cyanopyridine 20h........23
Figure S39. FTIR spectrum (nujol) of 2-hydrazino-3-cyanopyridine 20i........23
Figure S40. HRMS spectrum of 2-hydrazino-3-cyanopyridine 20i........24
Figure S41. FTIR spectrum (nujol) of 2-hydrazino-3-cyanopyridine 20j........24
Figure S42. HRMS spectrum of 2-hydrazino-3-cyanopyridine 20j........24
Figure S43. ^{1}H NMR spectrum of 2-hydrazino-3-cyanopyridine 20j, DMSO-d_6 (400 MHz) 25
Figure S44. ^{13}C NMR spectrum of 2-hydrazino-3-cyanopyridine 20j, DMSO-d_6 (101 MHz)25
Figure S45. FTIR spectrum (nujol) of hydrazone 21{*1*}........26
Figure S46. ^{1}H NMR spectrum of hydrazone 21{*1*}, DMSO-d_6 (400 MHz)........26
Figure S47. HRMS spectrum of hydrazone 21{*1*}........27
Figure S48. FTIR spectrum (nujol) of hydrazone 21{*3*}........27
Figure S49. HRMS spectrum of hydrazone 21{*3*}........27
Figure S50. ^{1}H NMR spectrum of hydrazone 21{*3*}, DMSO-d_6 (400 MHz)........28
Figure S51. ^{13}C NMR spectrum of hydrazone 21{*3*}, DMSO-d_6 (101 MHz)........28
Figure S52. FTIR spectrum (nujol) of hydrazone 21{*4*}........29
Figure S53. ^{1}H NMR spectrum of hydrazone 21{*4*}, acetone-d_6 (400 MHz)........29
Figure S54. ^{13}C NMR spectrum of hydrazone 21{*4*}, DMSO-d_6 (101 MHz)........30
Figure S55. HRMS spectrum of hydrazone 21{*4*}........30
Figure S56. HRMS spectrum of hydrazone 21{*5*}........30
Figure S57. FTIR spectrum (nujol) of hydrazone 21{*5*}........31
Figure S58. FTIR spectrum (nujol) of hydrazone 21{*6*}........31
Figure S59. ^{1}H NMR spectrum of hydrazone 21{*6*}, DMSO-d_6 (400 MHz)........32
Figure S60. HRMS spectrum of hydrazone 21{*6*}........32
Figure S61. FTIR spectrum (nujol) of hydrazone 21{*7*}........33
Figure S62. ^{1}H NMR spectrum of hydrazone 21{*7*}, DMSO-d_6 (400 MHz)........33
Figure S63. HRMS spectrum of hydrazone 21{*7*}........34
Figure S64. FTIR spectrum (nujol) of hydrazone 21{*8*}........34
Figure S65. HRMS spectrum of hydrazone 21{*8*}........34

Figure S66. ^{1}H NMR spectrum of hydrazone 21{*8*}, DMSO-d_6 (400 MHz) 35
Figure S67. ^{13}C NMR spectrum of hydrazone 21{*8*}, DMSO-d_6 (101 MHz) 35
Figure S68. FTIR spectrum (nujol) of hydrazone 21{*9*} 36
Figure S69. ^{1}H NMR spectrum of hydrazone 21{*9*}, DMSO-d_6 (400 MHz) 36
Figure S70. ^{13}C NMR spectrum of hydrazone 21{*9*}, DMSO-d_6 (101 MHz) 37
Figure S71. HRMS spectrum of hydrazone 21{*9*} 37
Figure S72. FTIR spectrum (nujol) of hydrazone 21{*11*}, solvate with EtOH 1 : 1 38
Figure S73. ^{1}H NMR spectrum of hydrazone 21{*11*}, solvate with EtOH 1 : 1, DMSO-d_6 (400 MHz) 38
Figure S74. ^{13}C NMR spectrum of hydrazone 21{*11*}, solvate with EtOH 1 : 1, DMSO-d_6 (101 MHz) 39
Figure S75. HRMS spectrum of hydrazone 21{*11*} 39
Figure S76. FTIR spectrum (nujol) of hydrazone 21{*12*}, solvate with EtOH 1 : 1 40
Figure S77. ^{1}H NMR spectrum of hydrazone 21{*12*}, solvate with EtOH 1 : 1, DMSO-d_6 (400 MHz) 40
Figure S78. ^{13}C NMR spectrum of hydrazone 21{*12*}, solvate with EtOH 1 : 1, DMSO-d_6 (101 MHz) 41
Figure S79. HRMS spectrum of hydrazone 21{*12*} 41
Figure S80. FTIR spectrum (nujol) of hydrazone 21{*13*} 42
Figure S81. ^{1}H NMR spectrum of hydrazone 21{*13*}, DMSO-d_6 (400 MHz) 42
Figure S82. ^{13}C NMR spectrum of hydrazone 21{*13*}, DMSO-d_6 (101 MHz) 43
Figure S83. HRMS spectrum of hydrazone 21{*13*} 43
Figure S84. HRMS spectrum of hydrazone 21{*14*} 43
Figure S85. FTIR spectrum (nujol) of hydrazone 21{*14*}, solvate with dioxane 1 : 1, ... 44
Figure S86. ^{1}H NMR spectrum of hydrazone 21{*14*}, solvate with dioxane 1 : 1, DMSO-d_6 (400 MHz) 44
Figure S87. ^{13}C NMR spectrum of hydrazone 21{*14*}, solvate with dioxane 1 : 1, DMSO-d_6 (101 MHz) 45
Figure S88. FTIR spectrum (nujol) of hydrazone 21{*15*} 45
Figure S89. ^{1}H NMR spectrum of hydrazone 21{*15*}, acetone-d_6 (400 MHz) 46
Figure S90. ^{13}C NMR spectrum of hydrazone 21{*15*}, acetone-d_6 (101 MHz) 46
Figure S91. HRMS spectrum of hydrazone 21{*15*} 47
Figure S92. FTIR spectrum (nujol) of hydrazone 21{*16*} 47
Figure S93. HRMS spectrum of hydrazone 21{*16*} 48
Figure S94. FTIR spectrum (nujol) of hydrazone 21{*17*} 48
Figure S95. FTIR spectrum (nujol) of hydrazone 21{*19*} 49
Figure S96. ^{1}H NMR spectrum of hydrazone 21{*19*}, DMSO-d_6 (400 MHz) 49
Figure S97. ^{13}C NMR spectrum of hydrazone 21{*19*}, DMSO-d_6 (101 MHz) 50
Figure S98. HRMS spectrum of hydrazone 21{*19*} 50
Figure S99. FTIR spectrum (nujol) of hydrazone 21{*20*} 51
Figure S100. ^{1}H NMR spectrum of hydrazone 21{*20*}, DMSO-d_6 (400 MHz) 51
Figure S101. ^{13}C NMR spectrum of hydrazone 21{*20*}, DMSO-d_6 (101 MHz) 52
Figure S102. HRMS spectrum of hydrazone 21{*20*} 52

Figure S103. ORTEP drawing of X-ray structure for 2-bromo-4-(4-chlorophenyl)-6-phenylnicotinonitrile (15a) with 50% probability (CCDC deposition number 2499675) 53
Figure S104. Microphoto images of the single crystal of 2-bromo-4-(4-chlorophenyl)-6-phenylnicotinonitrile 15a 53
Table S1. Crystal data and structure refinement for 2-bromo-4-(4-chlorophenyl)-6-phenylnicotinonitrile (15a) 53
Table S2. Fractional Atomic Coordinates (×10^4) and Equivalent Isotropic Displacement Parameters (Å^2×10^3) for 2-bromo-4-(4-chlorophenyl)-6-phenylnicotinonitrile (15a). 55
Table S3. Anisotropic Displacement Parameters (Å^2×10^3) for 2-bromo-4-(4-chlorophenyl)-6-phenylnicotinonitrile (15a) 56
Table S4. Bond Lengths for 2-bromo-4-(4-chlorophenyl)-6-phenylnicotinonitrile (15a). 56
Table S5. Bond Angles for 2-bromo-4-(4-chlorophenyl)-6-phenylnicotinonitrile (15a) 57
Table S6. Torsion Angles for 2-bromo-4-(4-chlorophenyl)-6-phenylnicotinonitrile (15a) 57
Table S7. Hydrogen Atom Coordinates (Å×10^4) and Isotropic Displacement Parameters (Å^2×10^3) for 2-bromo-4-(4-chlorophenyl)-6-phenylnicotinonitrile (15a) 58
Figure S105. ORTEP drawings of X-ray structures for co-crystallized (*2S*,3R*,4S*,6R**)-3-benzoyl-5-bromo-4-hydroxy-4-phenyl-2,6-di-*p*-tolylcyclohexane-1,1-dicarbonitrile 17-Br (upper image) and (*2S*,3R*,4S*,6R**)-3-benzoyl-4-hydroxy-4-phenyl-2,6-di-*p*-tolylcyclohexane-1,1-dicarbonitrile 17 (bottom image), solvate with AcOH. Ellipsoids are given with 50% probability (CCDC deposition number 2499676). 59
Figure S106. Microphoto images of the single crystal of 17 + 17-Br co-crystals 60
Table S8. Crystal data and structure refinement for co-crystals of cyclohexanols 17 + 17-Br60
Table S9. Fractional Atomic Coordinates (×10^4) and Equivalent Isotropic Displacement Parameters (Å^2×10^3) for co-crystals of cyclohexanols 17 + 17-Br. 60
Table S10. Anisotropic Displacement Parameters (Å^2×10^3) for co-crystals of cyclohexanols 17 + 17-Br. 62
Table S11. Bond Lengths for co-crystals of cyclohexanols 17 + 17-Br 63
Table S12. Bond Angles for co-crystals of cyclohexanols 17 + 17-Br. 64
Table S13. Torsion Angles for co-crystals of cyclohexanols 17 + 17-Br 65
Table S14. Hydrogen Atom Coordinates (Å×10^4) and Isotropic Displacement Parameters (Å^2×10^3) for co-crystals of cyclohexanols 17 + 17-Br. 66
Table S15. Atomic Occupancy for co-crystals of cyclohexanols 17 + 17-Br 67

Figure S1. FTIR spectrum (nujol) of 2-bromo-3-cyanopyridine 15a

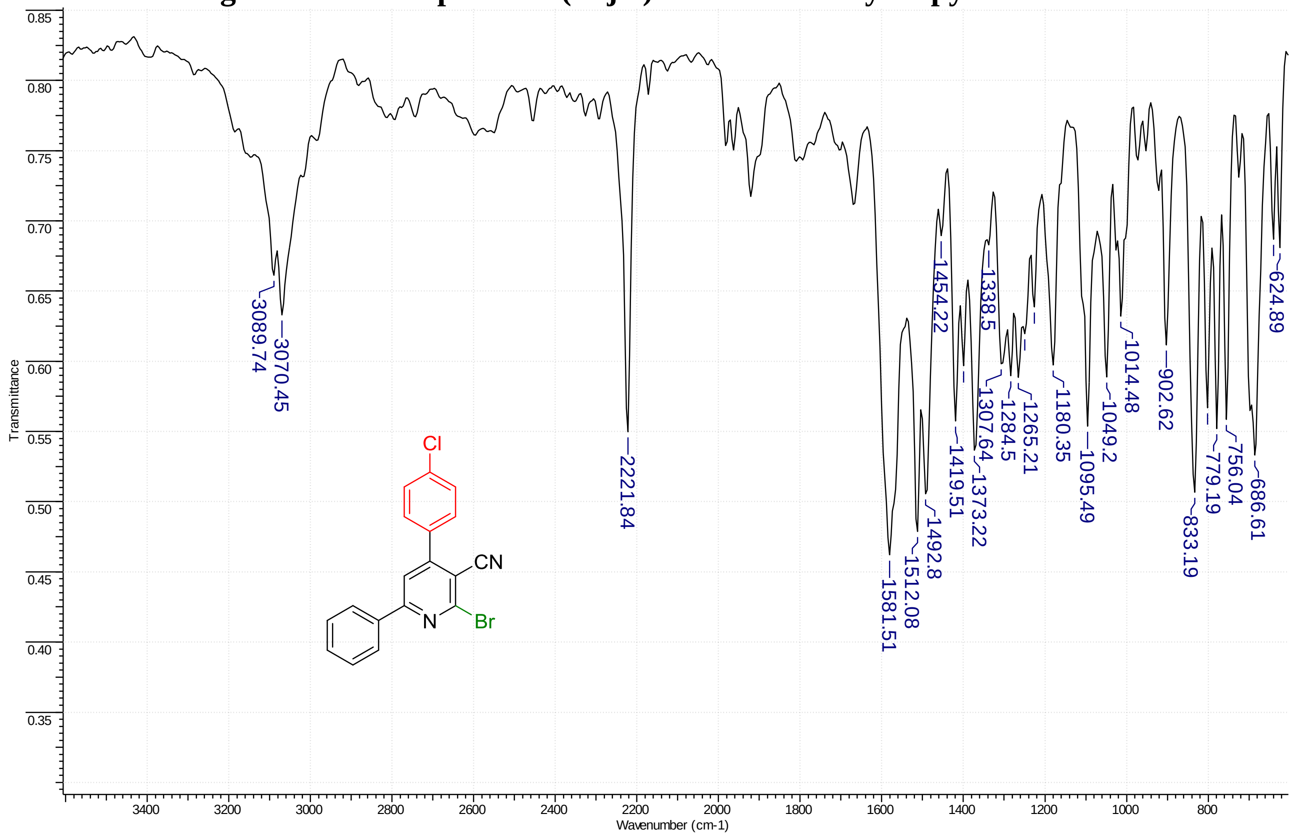

Figure S2. ^{1}H NMR spectrum of 2-bromo-3-cyanopyridine 15a, DMSO-d_6 (400 MHz)

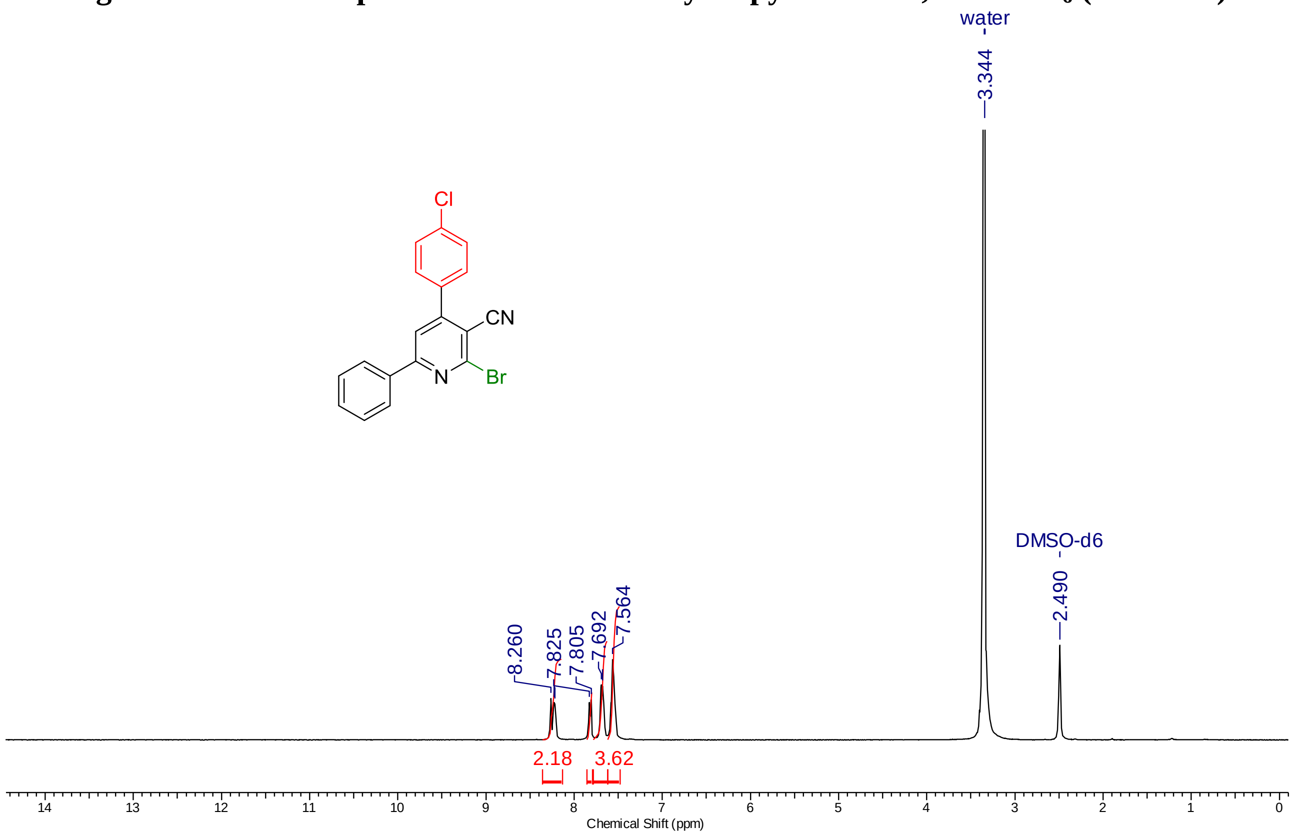

Figure S3. FTIR spectrum (KBr) of 2-bromo-3-cyanopyridine 15b

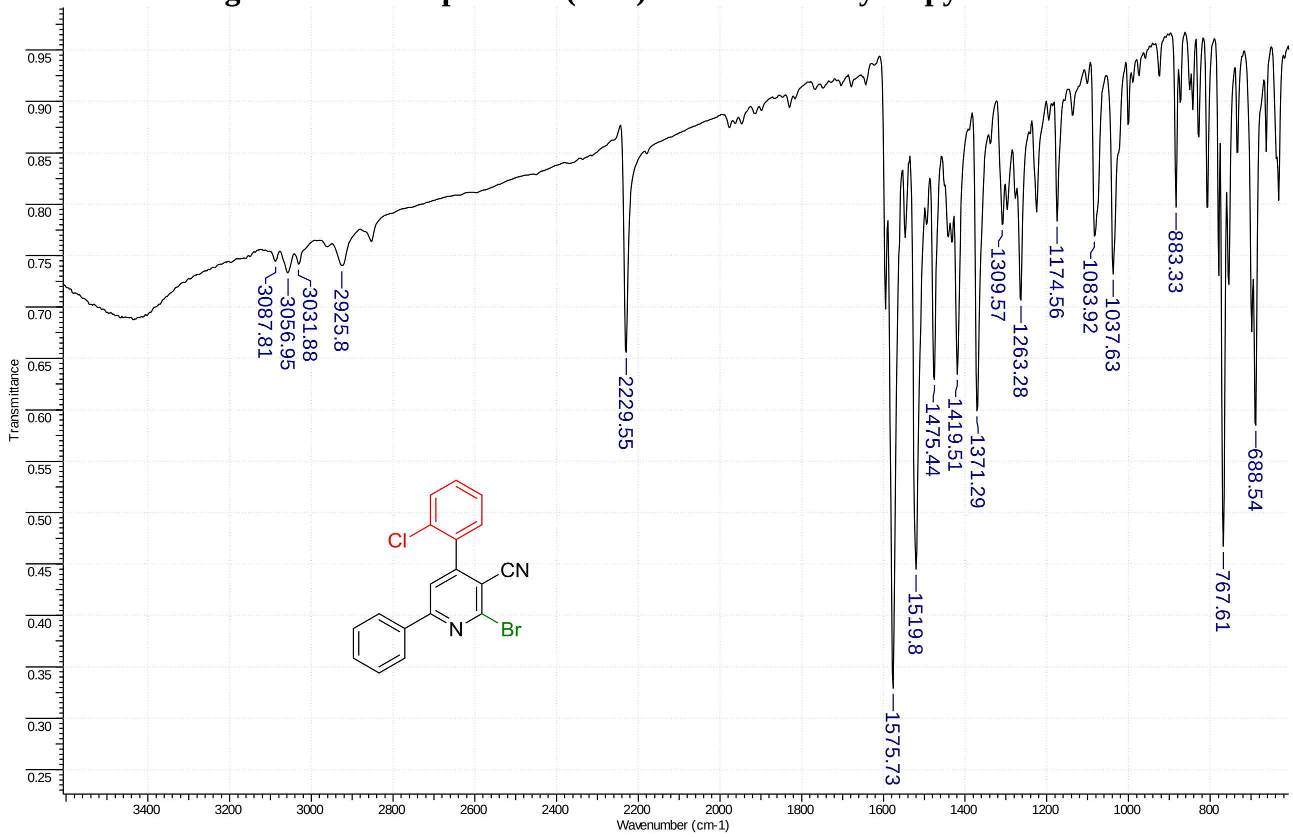

Figure S4. FTIR spectrum (nujol) of 2-bromo-3-cyanopyridine 15c

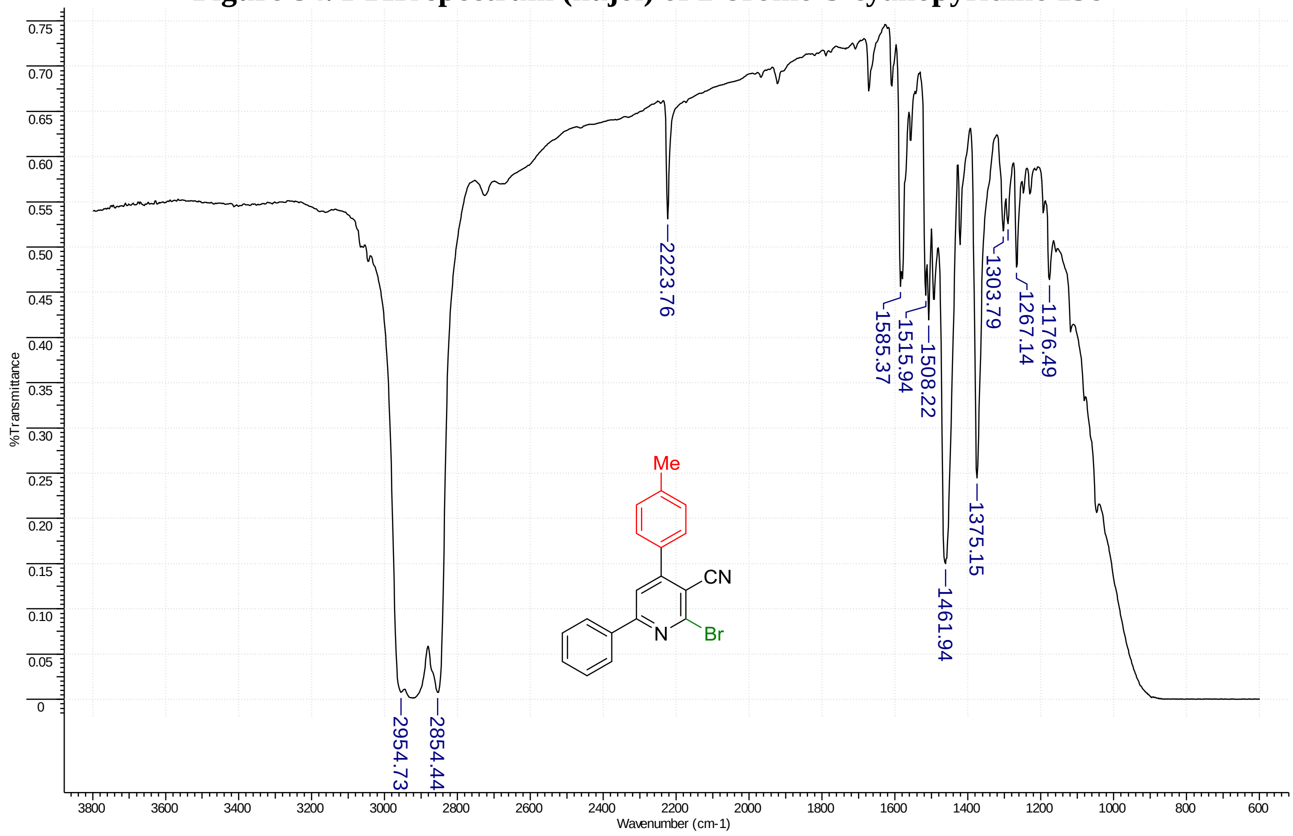

Figure S5. ^{1}H NMR spectrum of 2-bromo-3-cyanopyridine 15c, DMSO-d$_6$ (400 MHz)

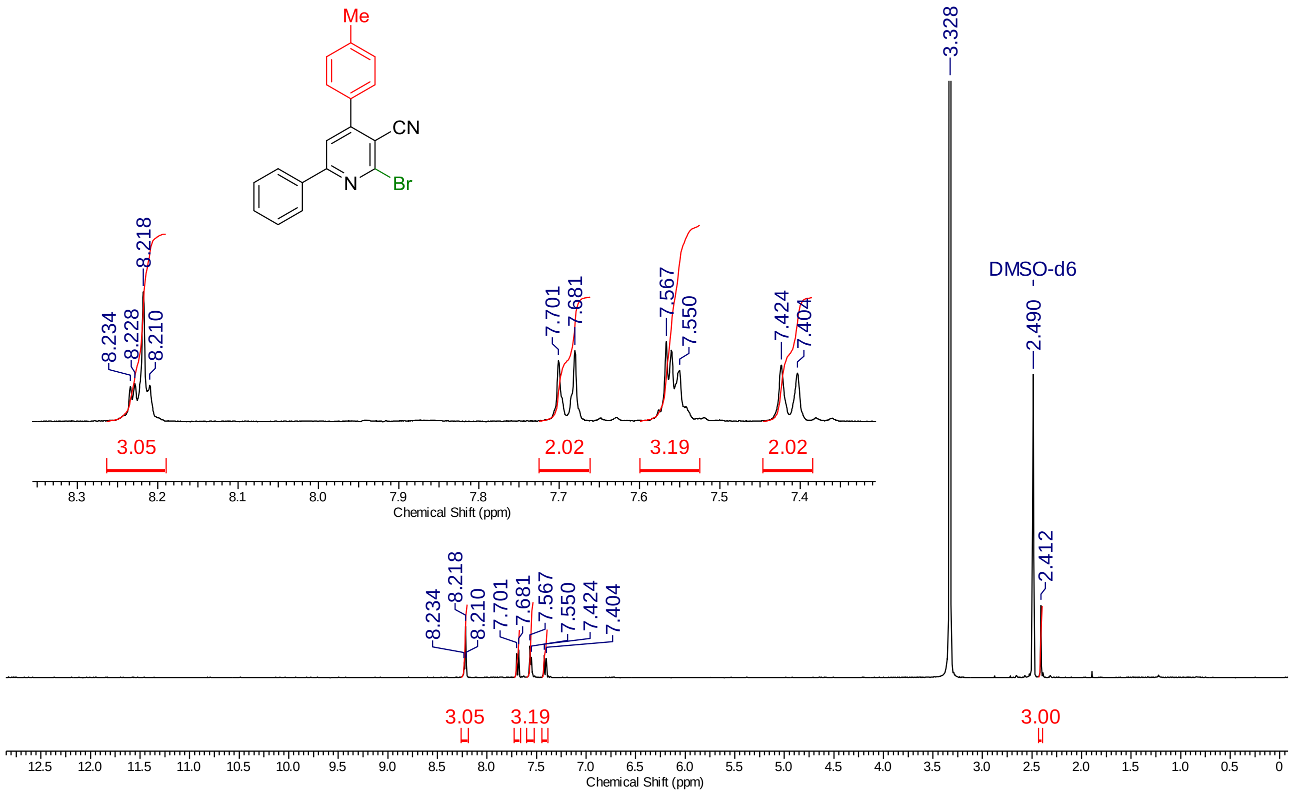

Figure S6. ^{13}C NMR DEPTQ spectrum of 2-bromo-3-cyanopyridine 15c, DMSO-d$_6$ (101 MHz)

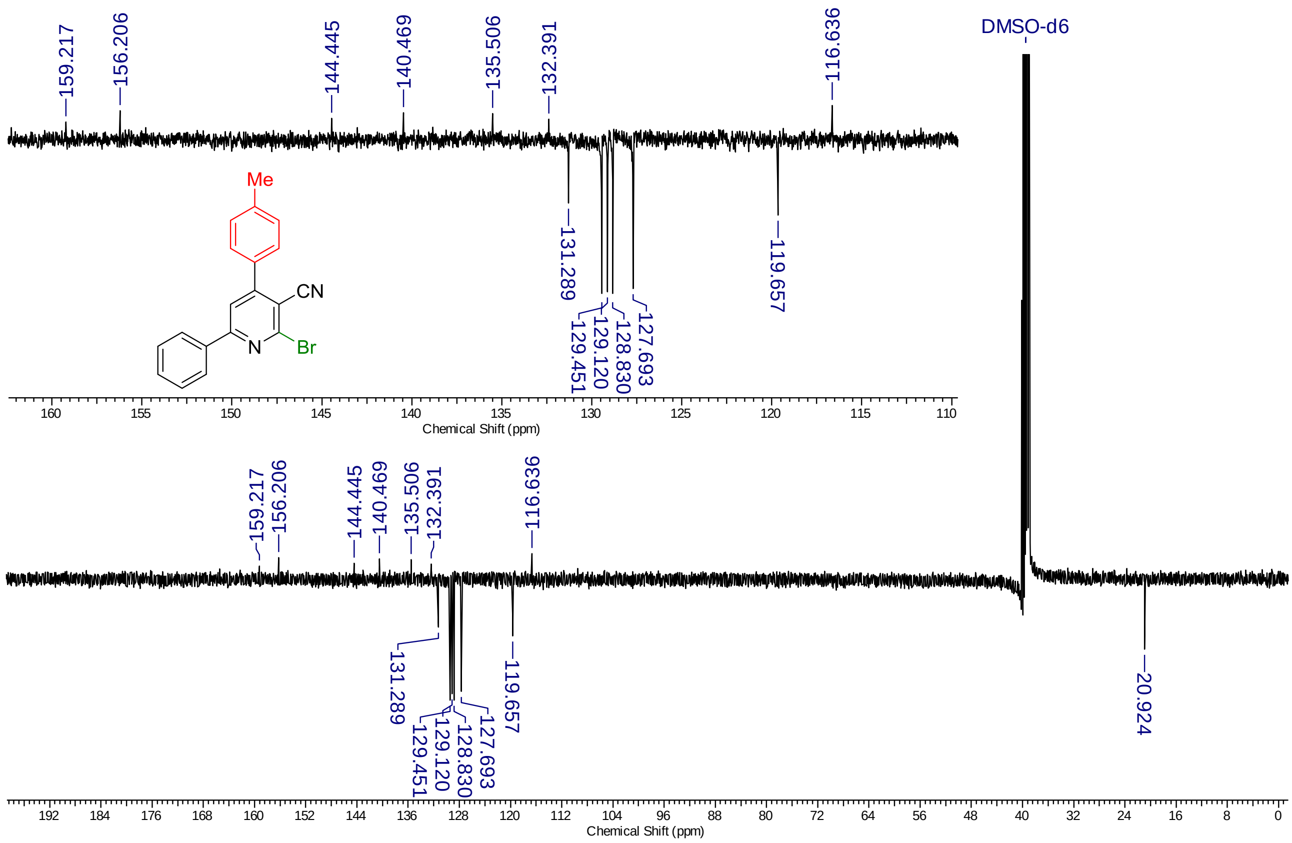

Figure S7. FTIR spectrum (KBr) of 2-bromo-3-cyanopyridine 15d

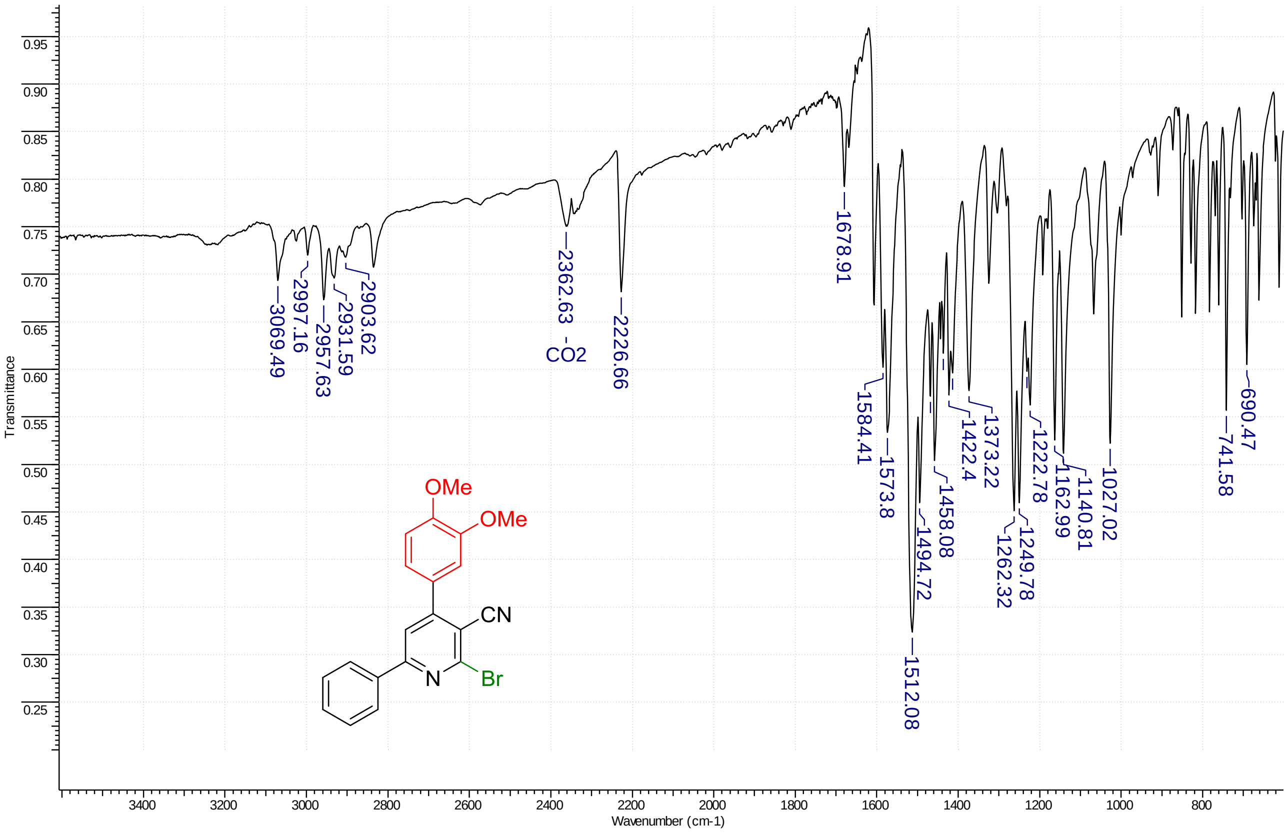

Figure S8. FTIR spectrum (KBr) of 2-bromo-3-cyanopyridine 15e

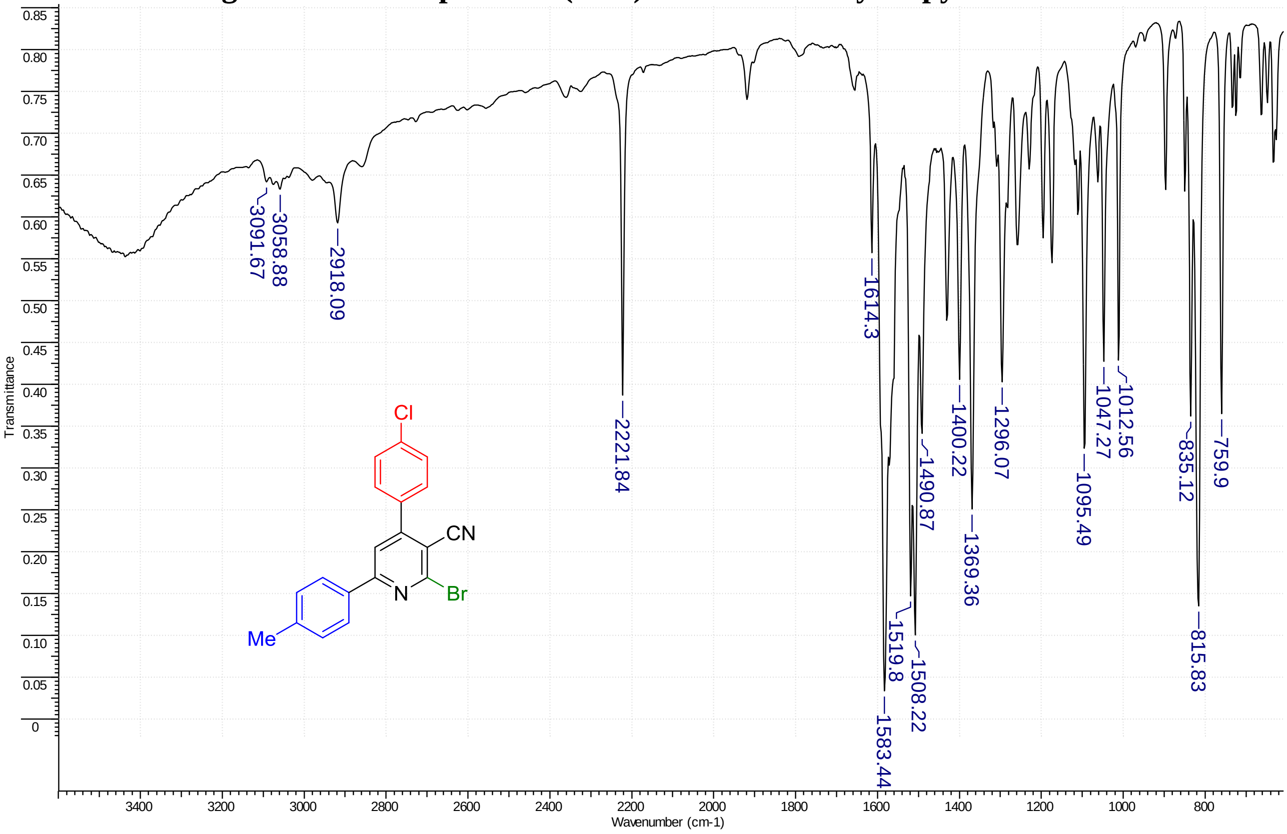

Figure S9. FTIR spectrum (KBr) of 2-bromo-3-cyanopyridine 15f

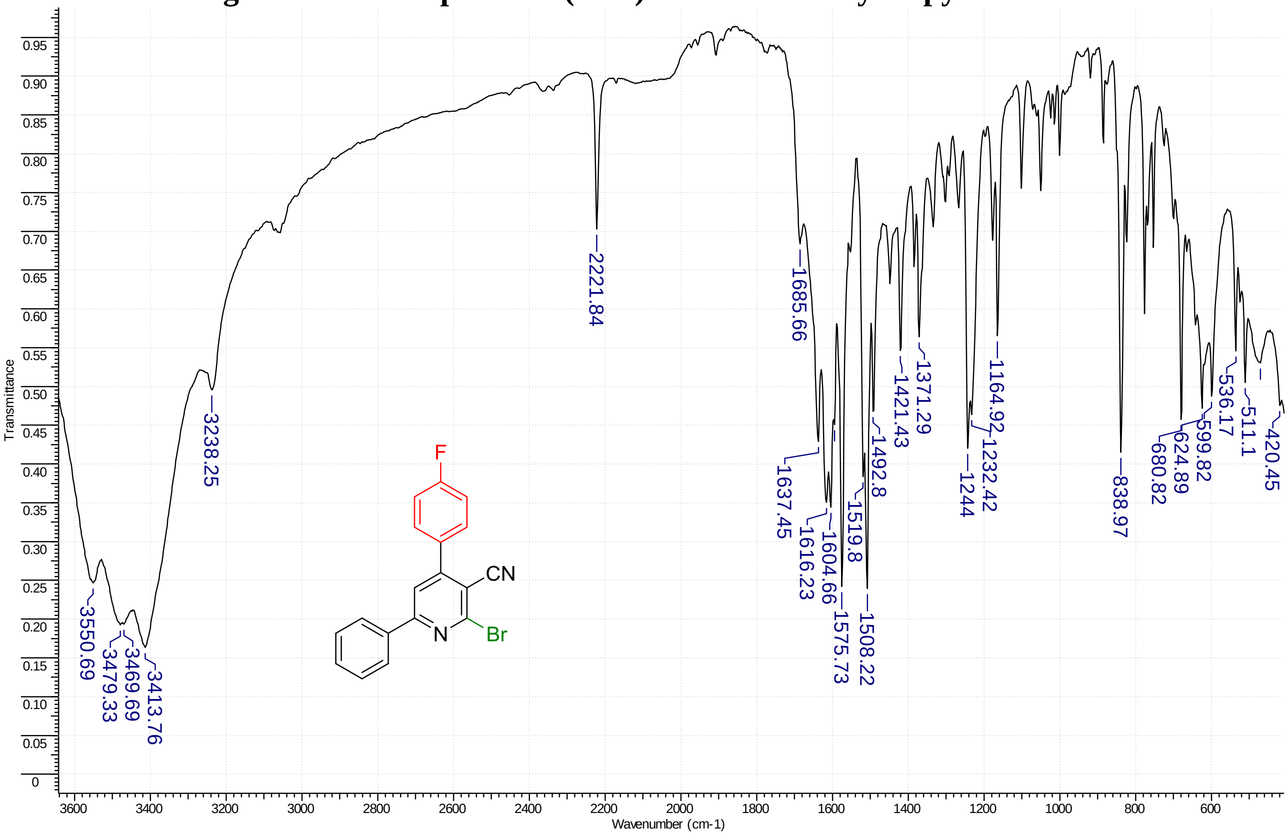

Figure S10. FTIR spectrum (nujol) of 2-hydrazino-3-cyanopyridine 20a

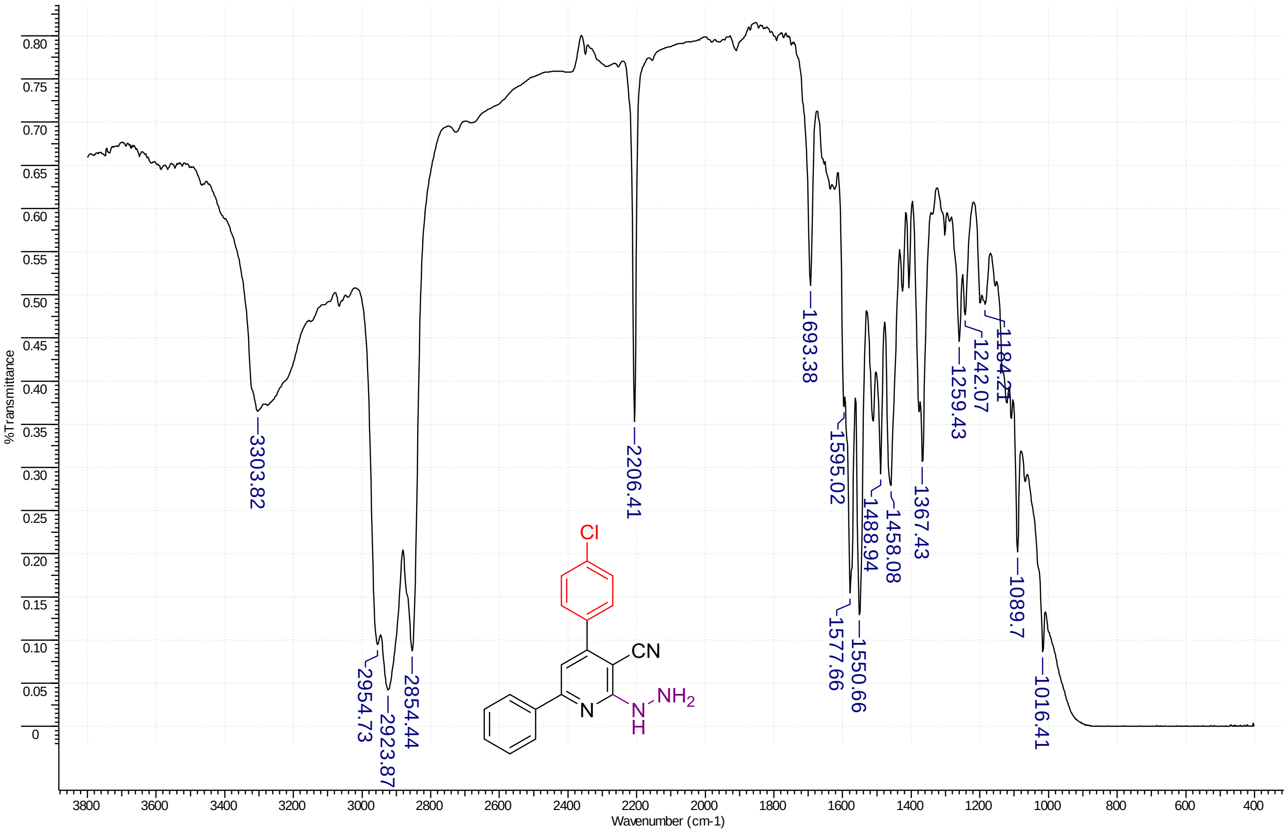

Figure S11. ^{1}**H NMR spectrum of 2-hydrazino-3-cyanopyridine 20a, DMSO-d$_6$ (400 MHz)**

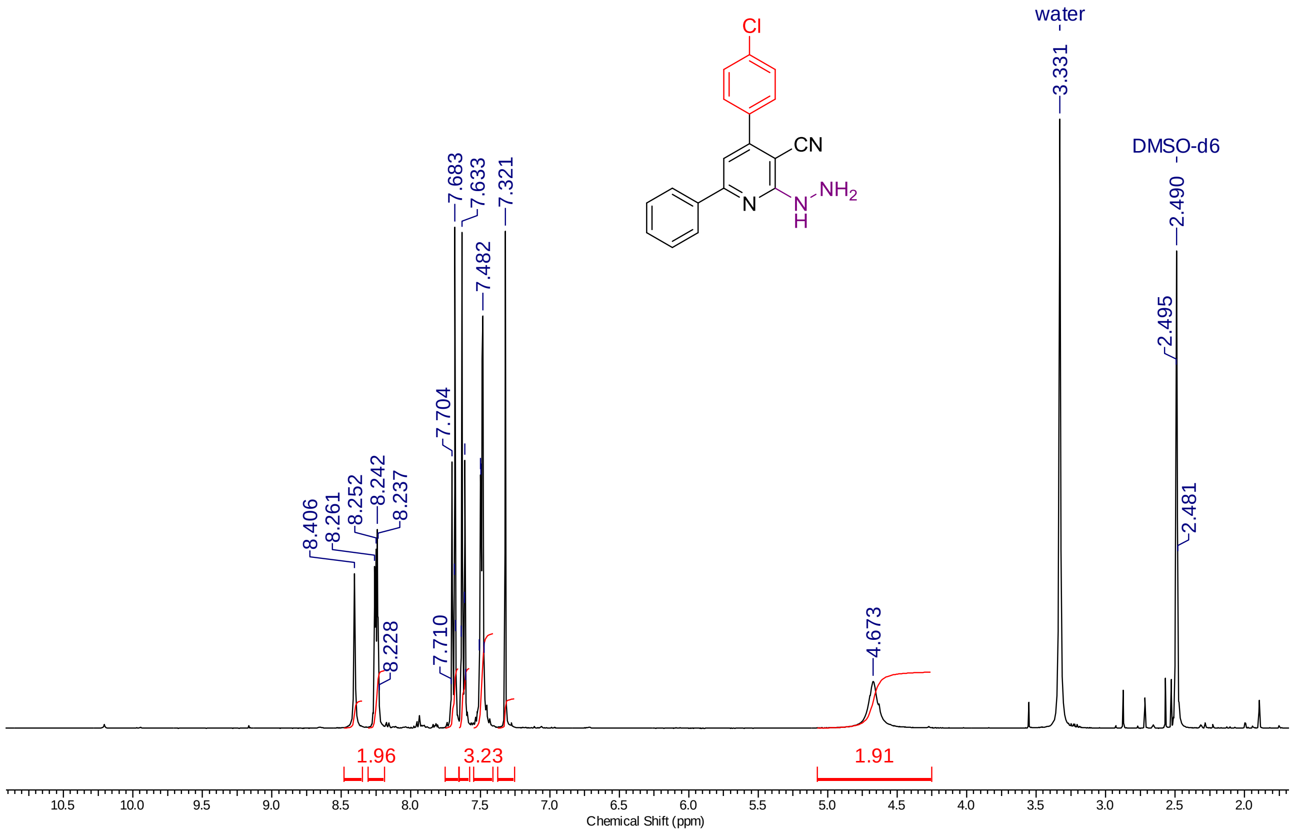

Figure S12. ^{13}**C DEPTQ NMR spectrum of 2-hydrazino-3-cyanopyridine 20a, DMSO-d$_6$ (101 MHz)**

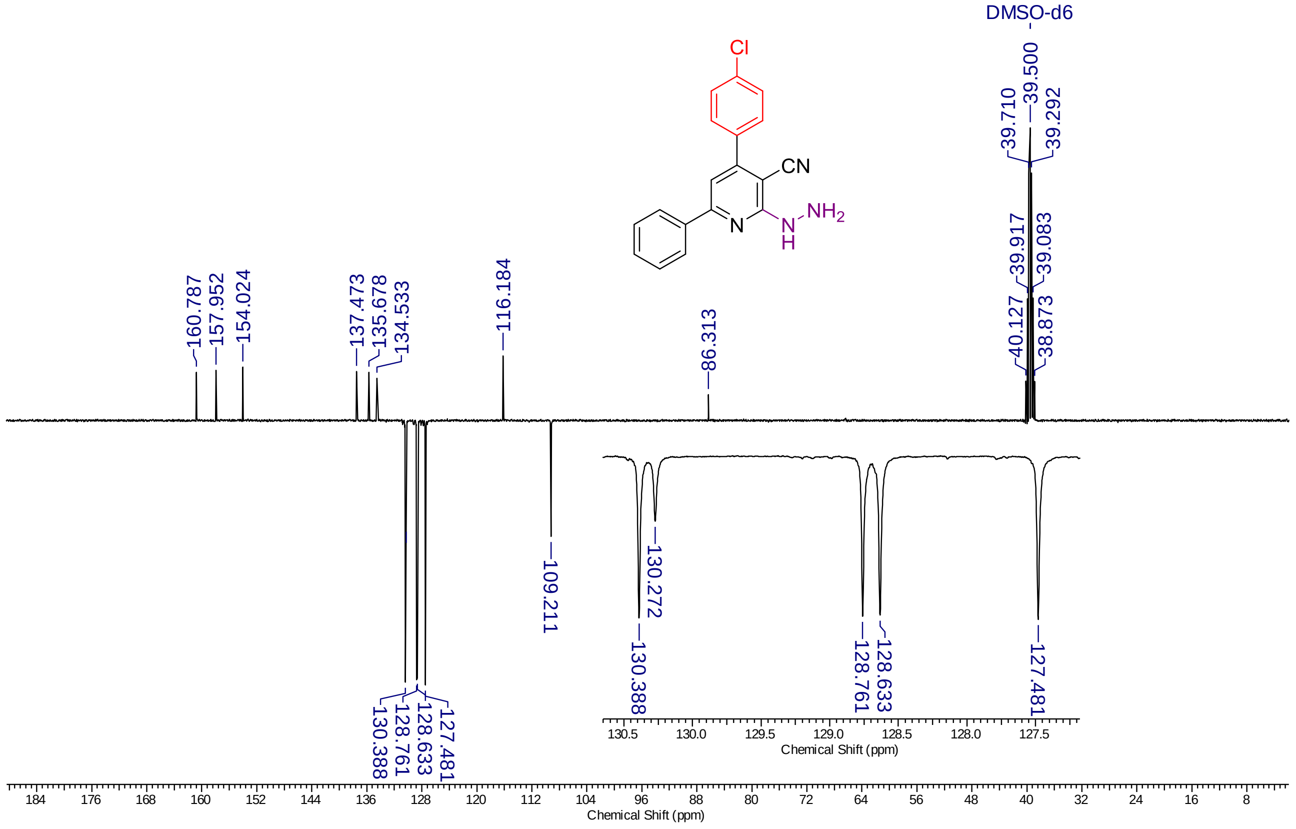

Figure S13. HRMS spectrum of 2-hydrazino-3-cyanopyridine 20a

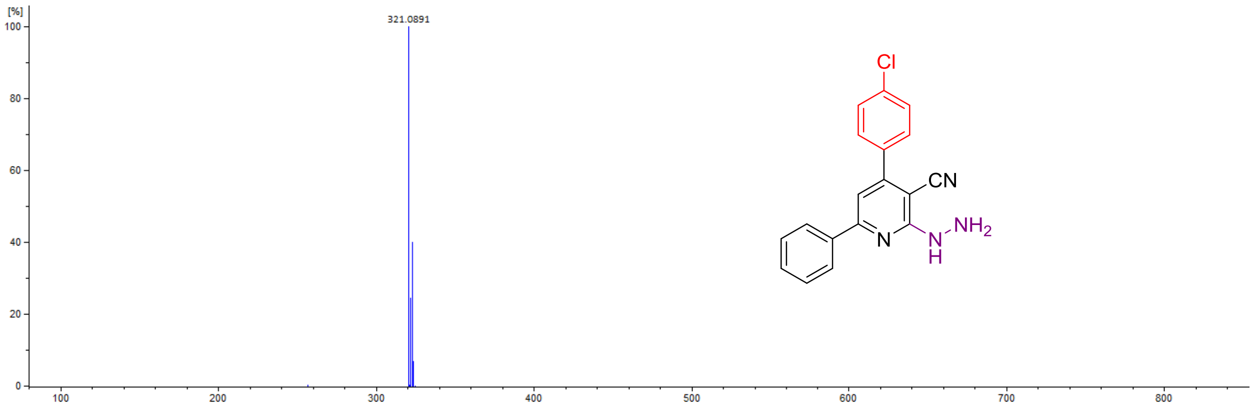

Figure S14. FTIR spectrum (nujol) of 2-hydrazino-3-cyanopyridine 20b

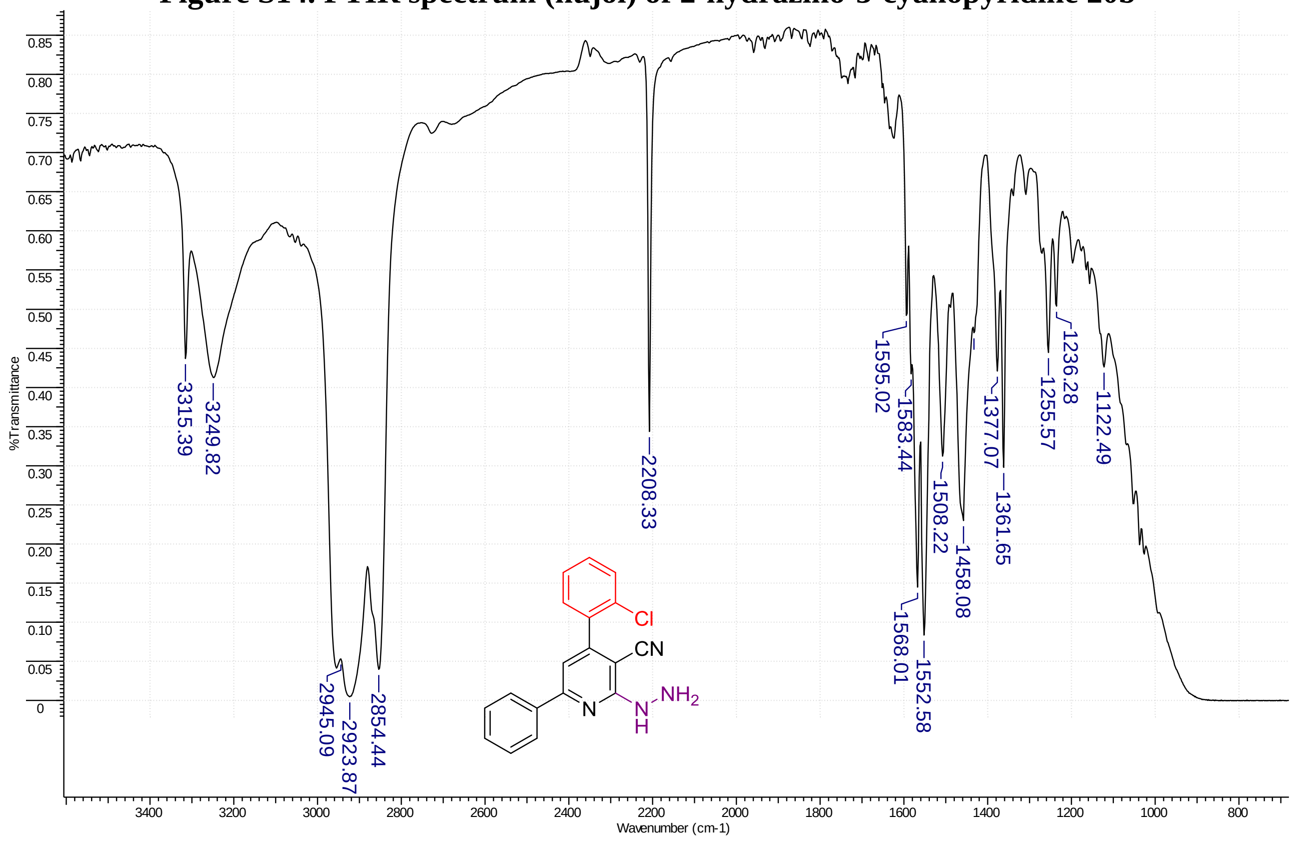

Figure S15. ^{1}H NMR spectrum of 2-hydrazino-3-cyanopyridine 20b, DMSO-d_6 (400 MHz)

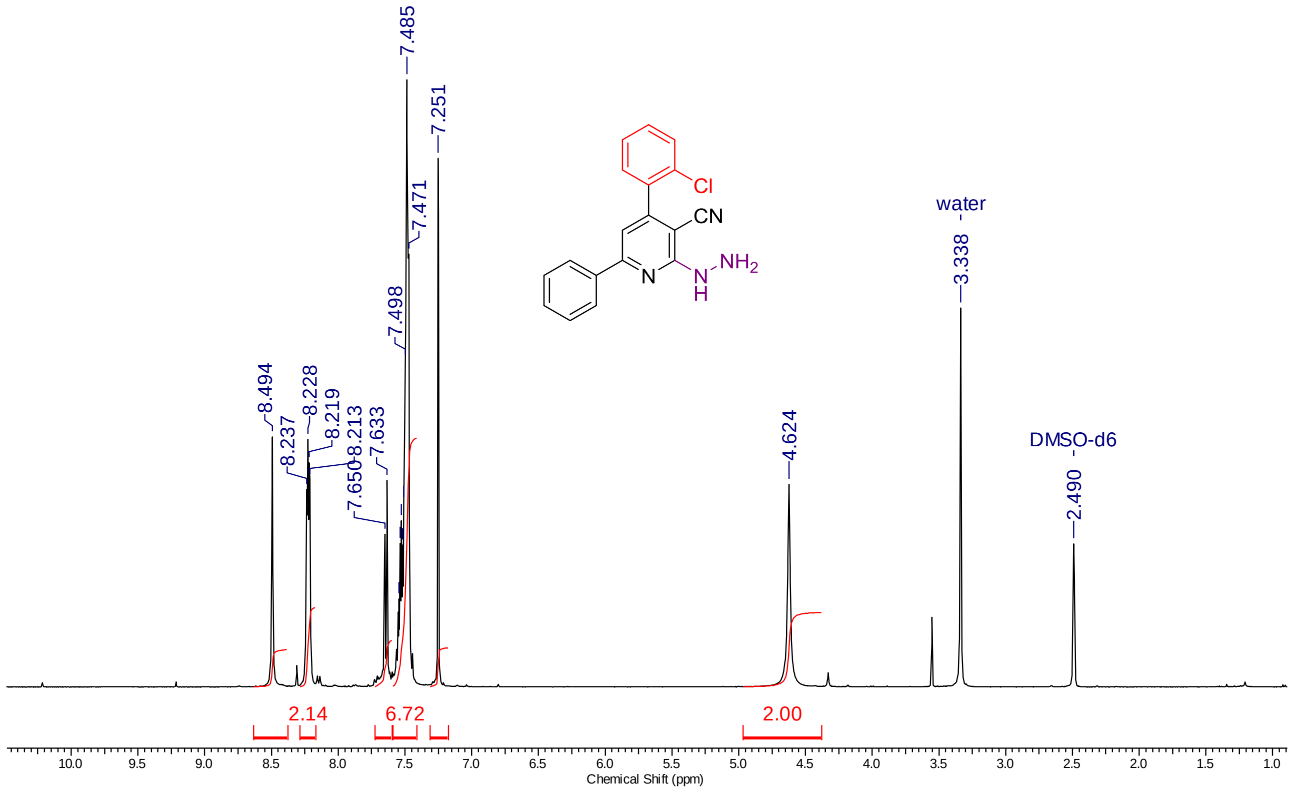

Figure S16. ^{13}C NMR spectrum of 2-hydrazino-3-cyanopyridine 20b, DMSO-d_6 (101 MHz)

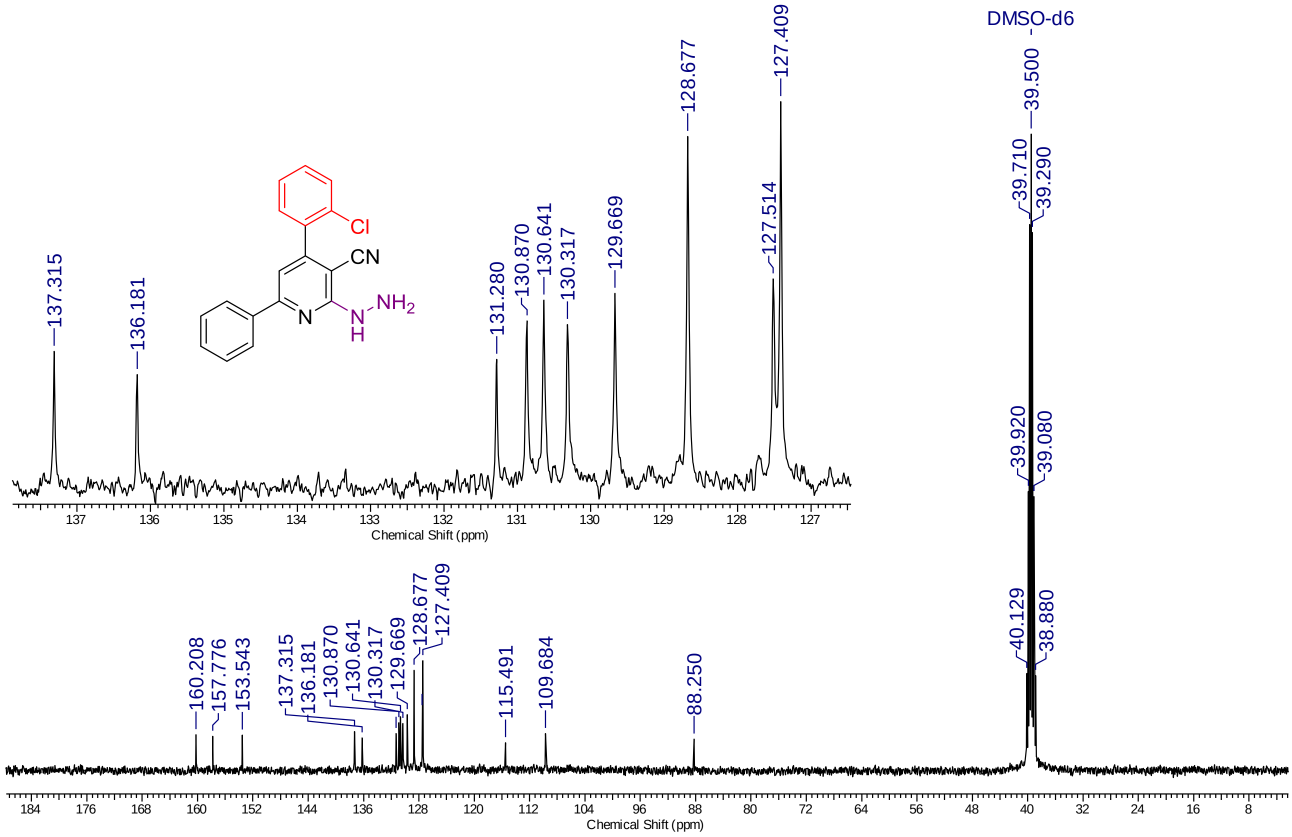

Figure S17. HRMS spectrum of 2-hydrazino-3-cyanopyridine 20b

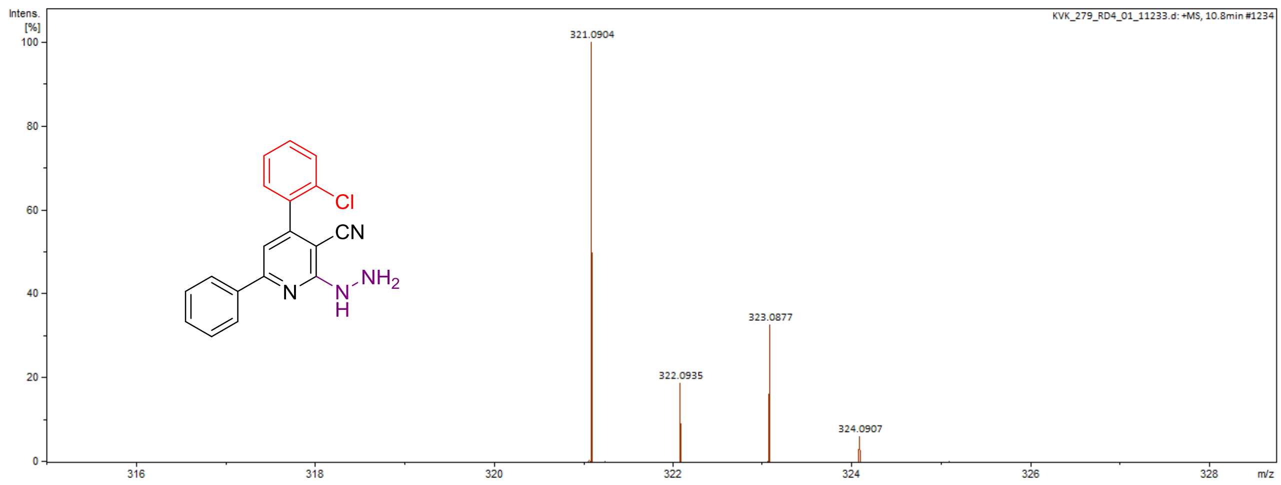

Figure S18. FTIR spectrum (nujol) of 2-hydrazino-3-cyanopyridine 20c

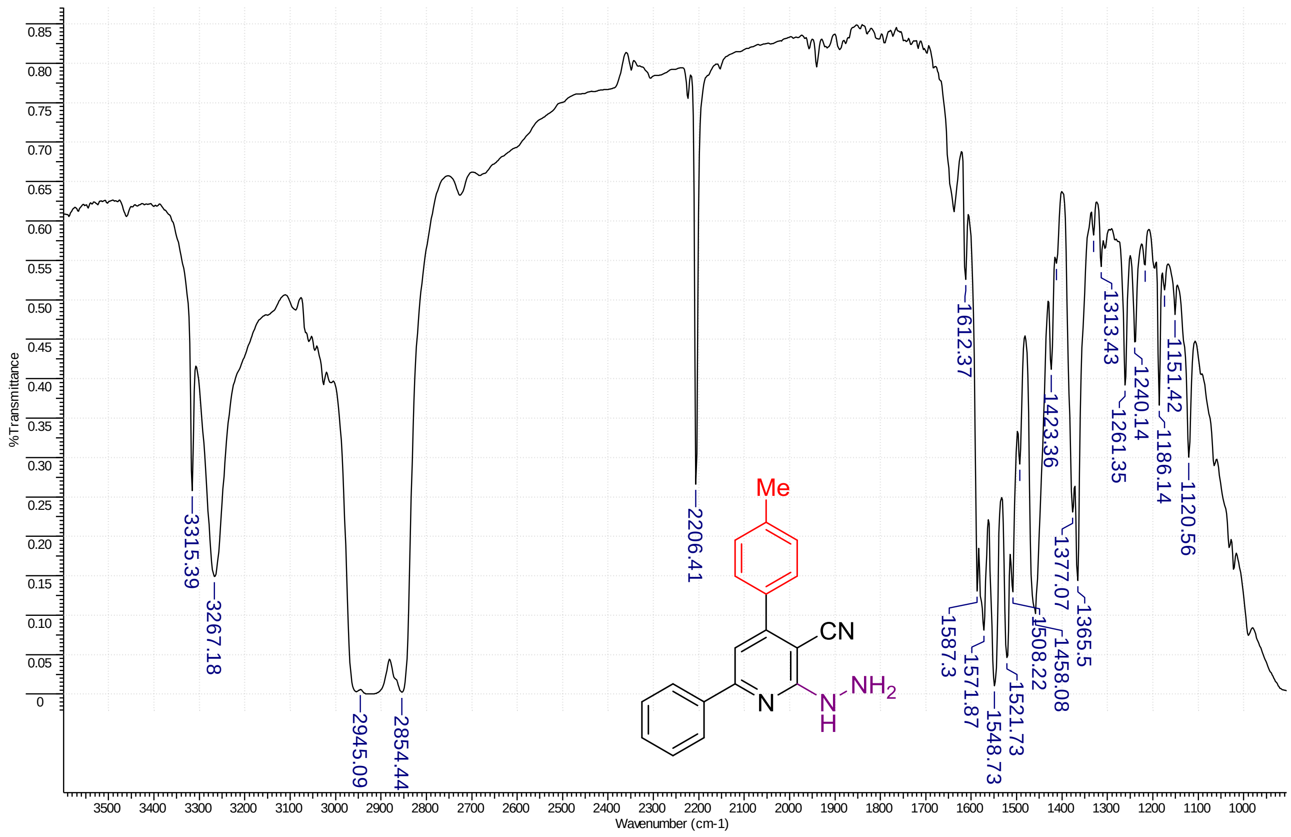

Figure S19. ^{1}H NMR spectrum of 2-hydrazino-3-cyanopyridine 20c, DMSO-d_6 (400 MHz)

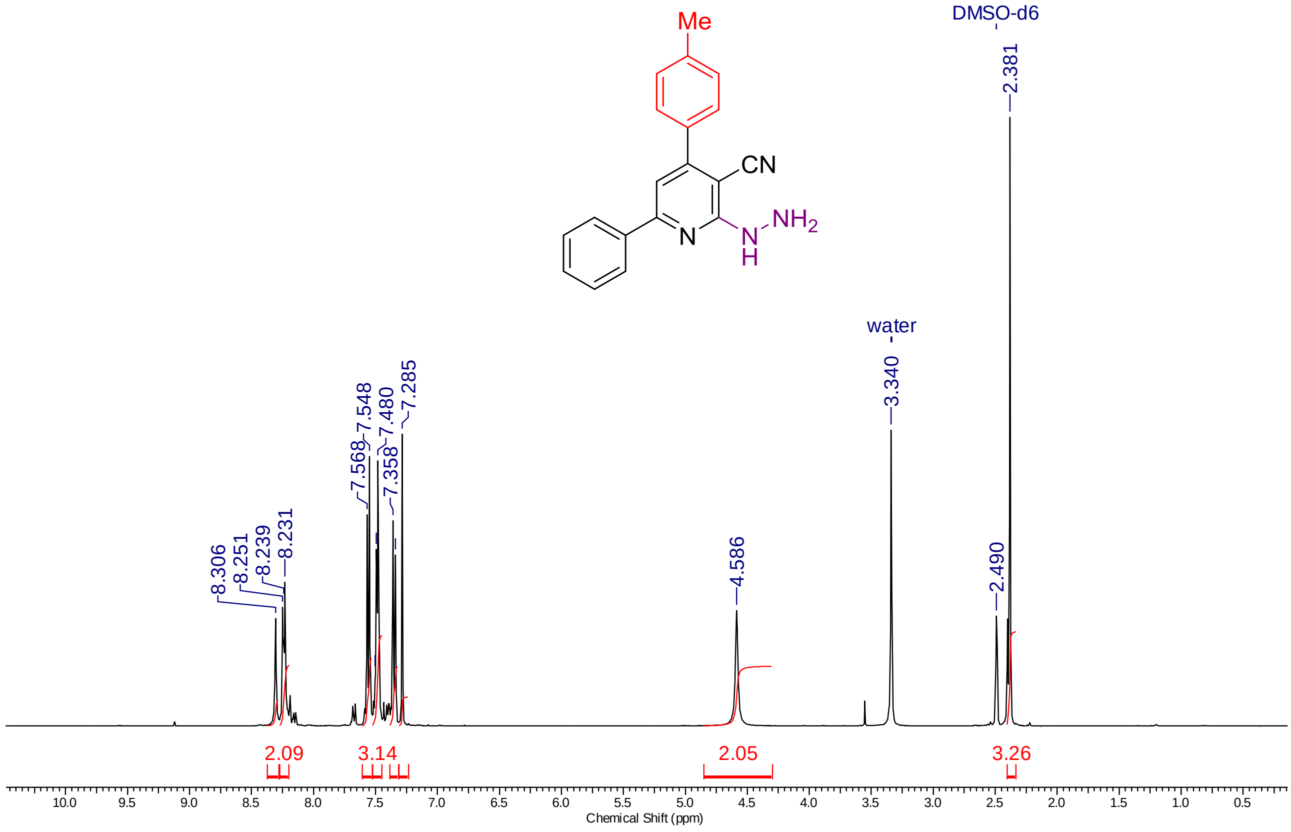

Figure S20. ^{13}C NMR spectrum of 2-hydrazino-3-cyanopyridine 20c, DMSO-d_6 (101 MHz)

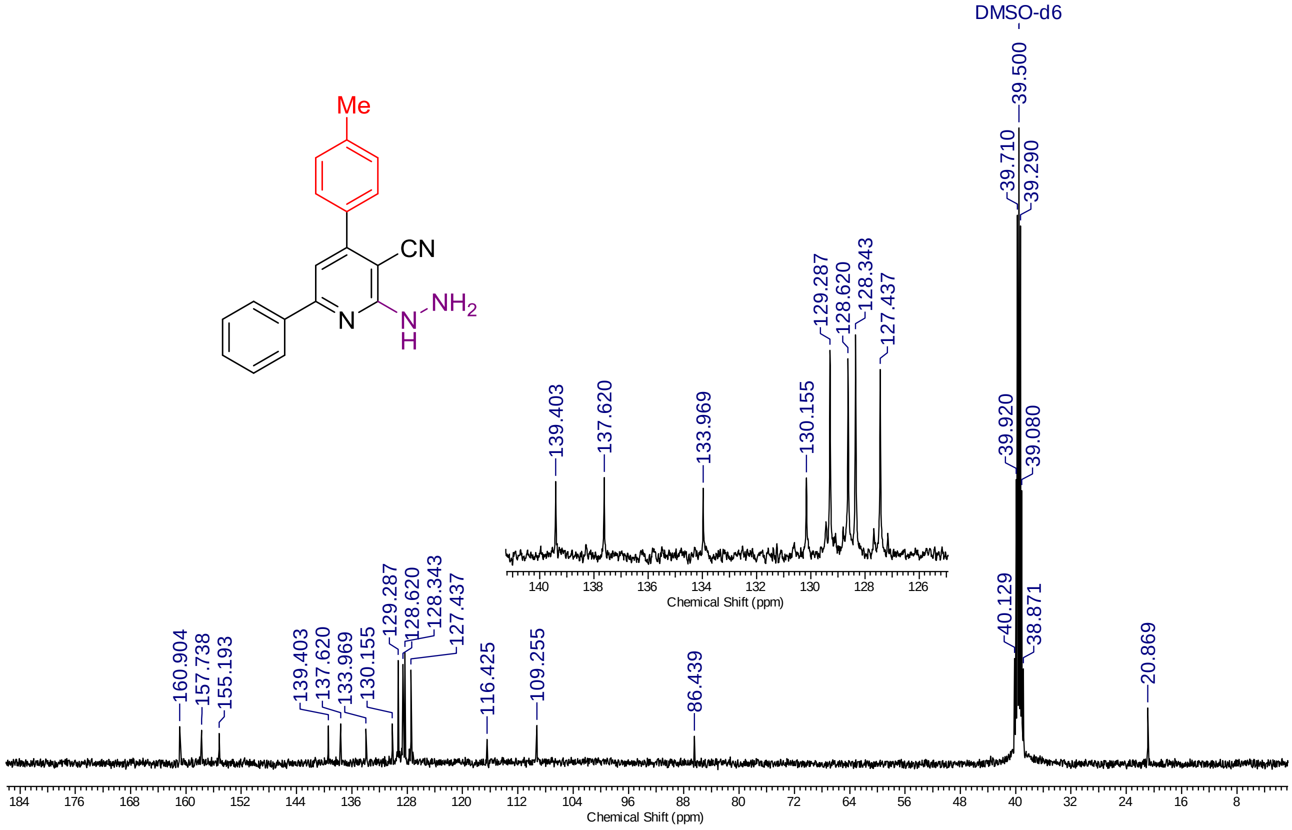

Figure S21. FTIR spectrum (nujol) of 2-hydrazino-3-cyanopyridine 20d

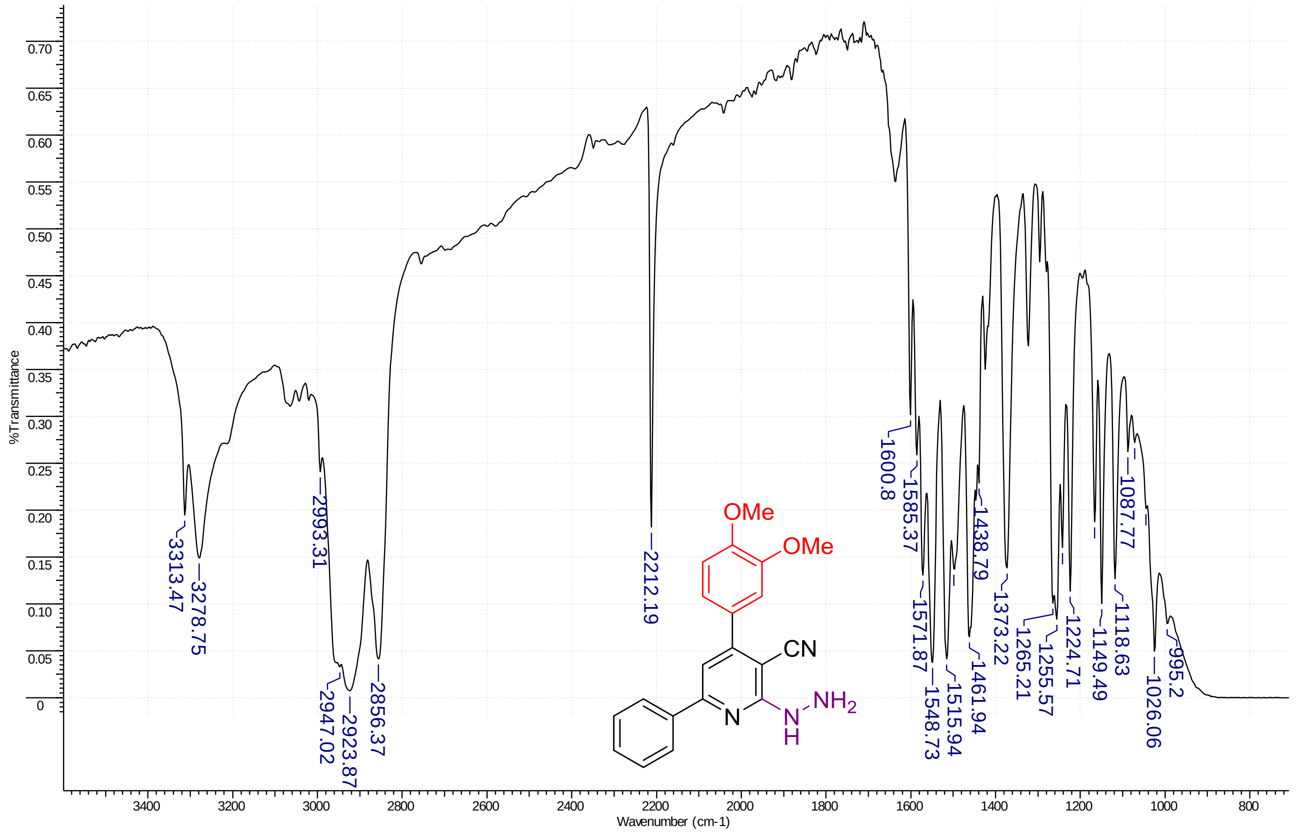

Figure S22. [1]**H NMR spectrum of 2-hydrazino-3-cyanopyridine 20d, DMSO-d$_6$ (400 MHz)**

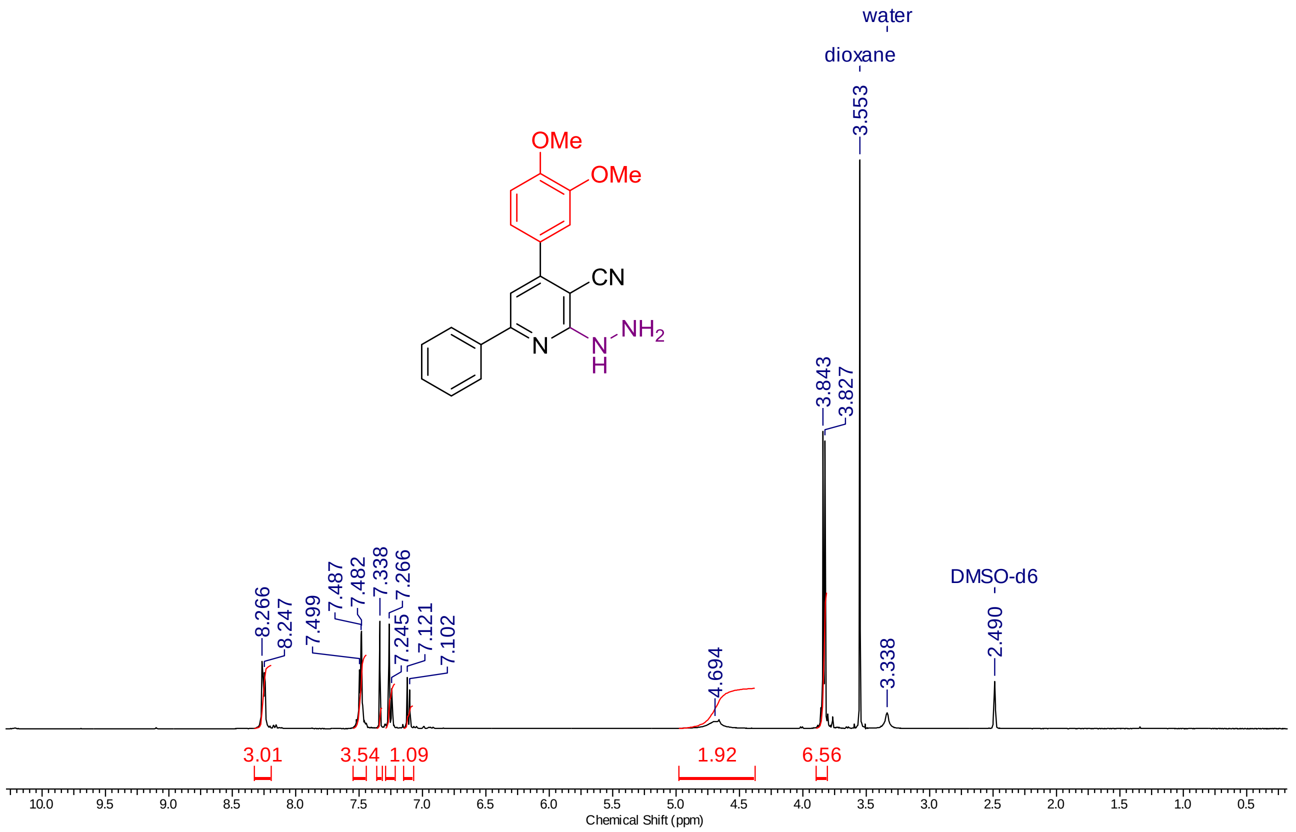

Figure S23. ^{13}C NMR spectrum of 2-hydrazino-3-cyanopyridine 20d, DMSO-d$_6$ (101 MHz)

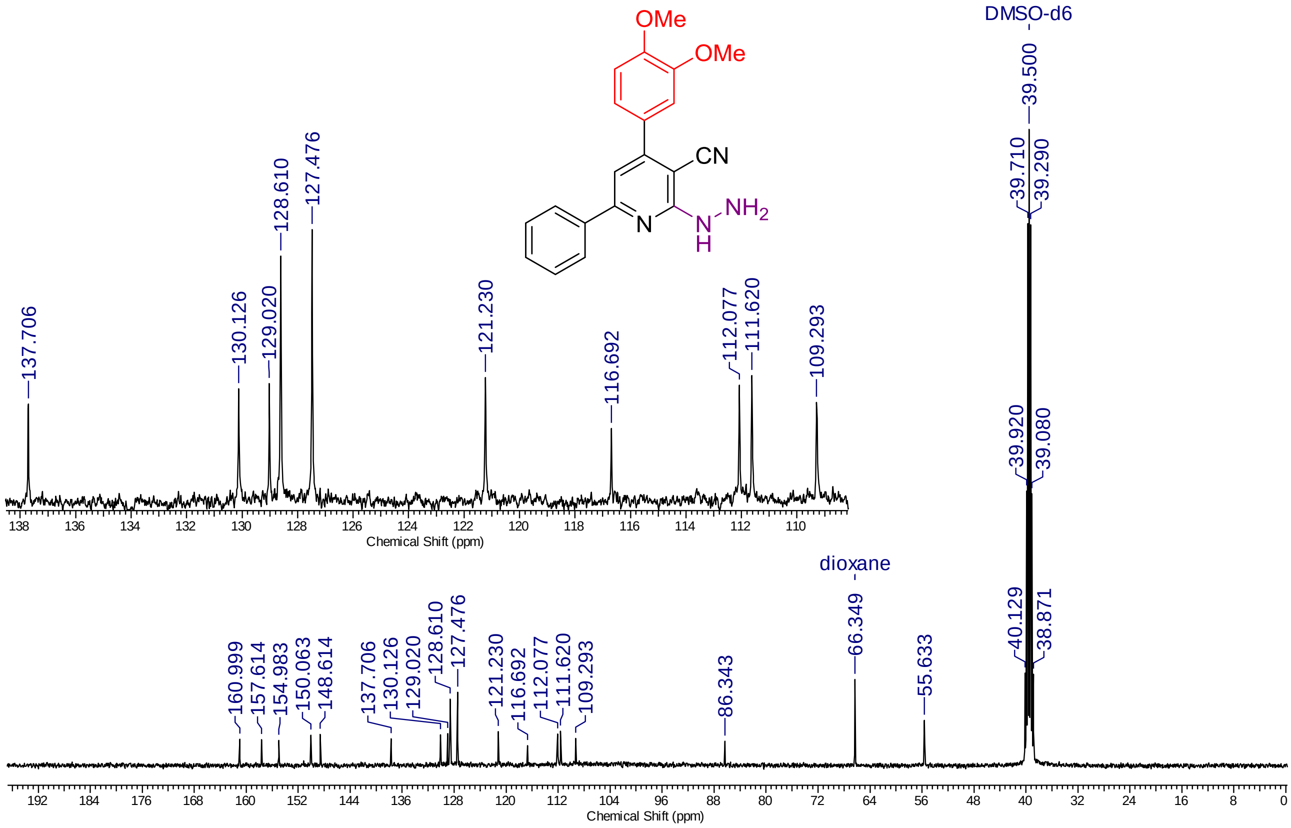

Figure S24. HRMS spectrum of 2-hydrazino-3-cyanopyridine 20d

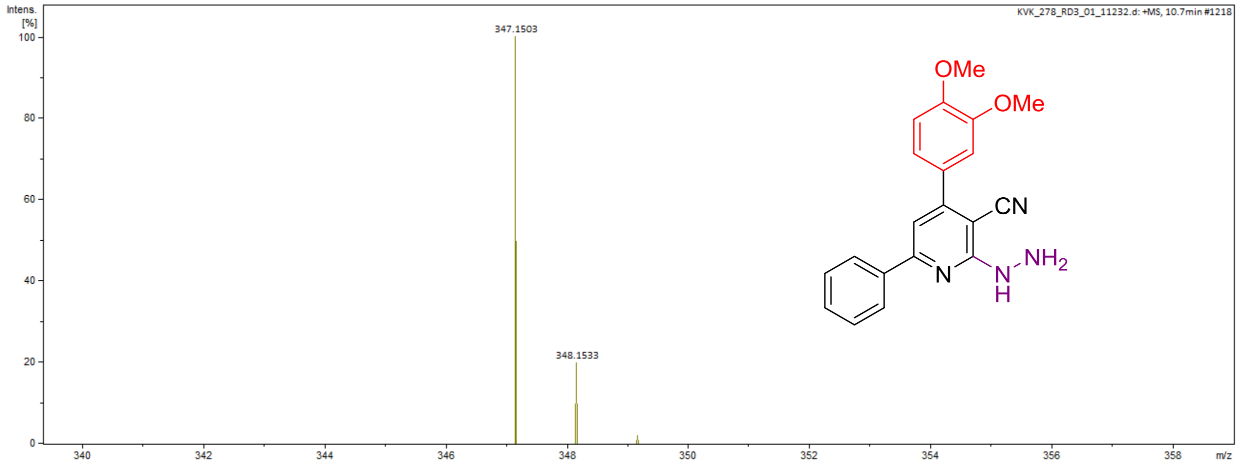

Figure S25. FTIR spectrum (nujol) of 2-hydrazino-3-cyanopyridine 20e

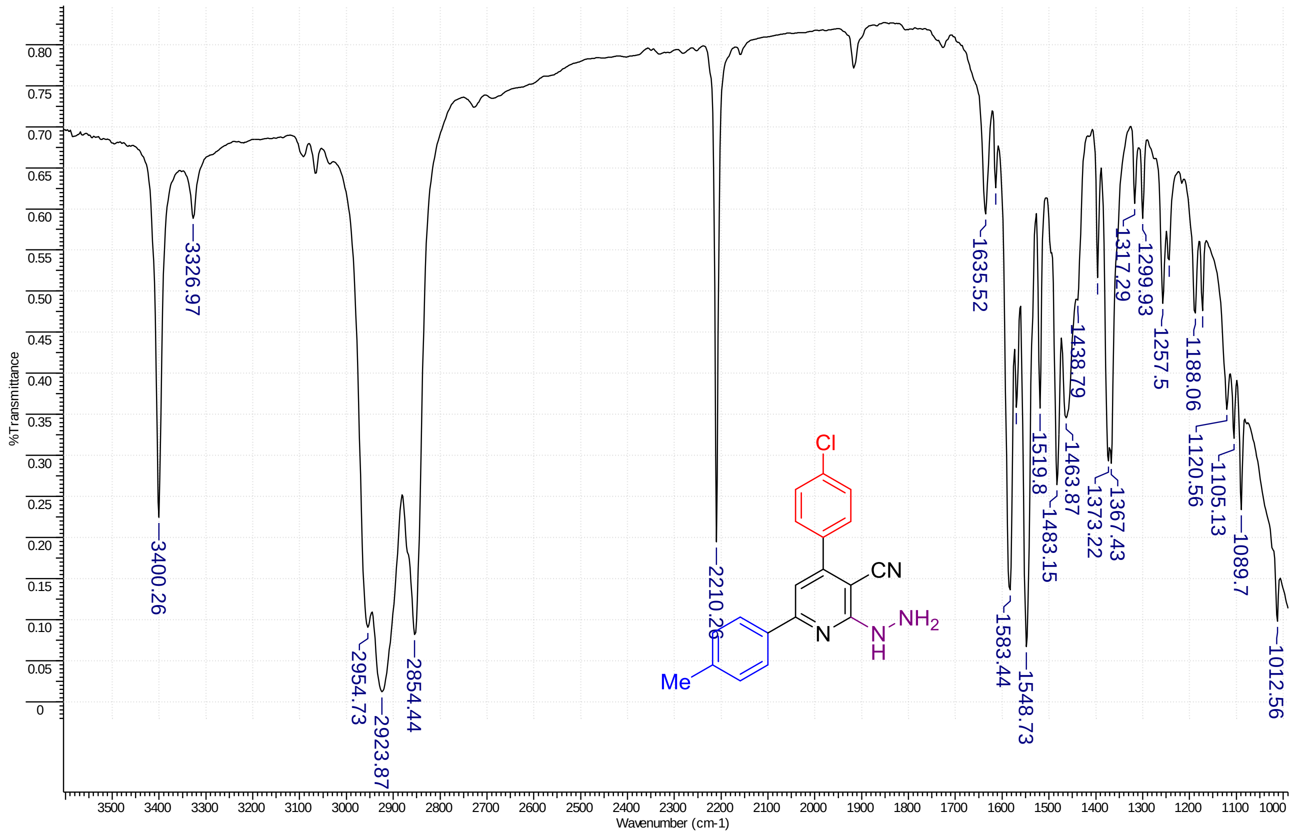

Figure S26. ^{1}H NMR spectrum of 2-hydrazino-3-cyanopyridine 20e, DMSO-d_6 (400 MHz)

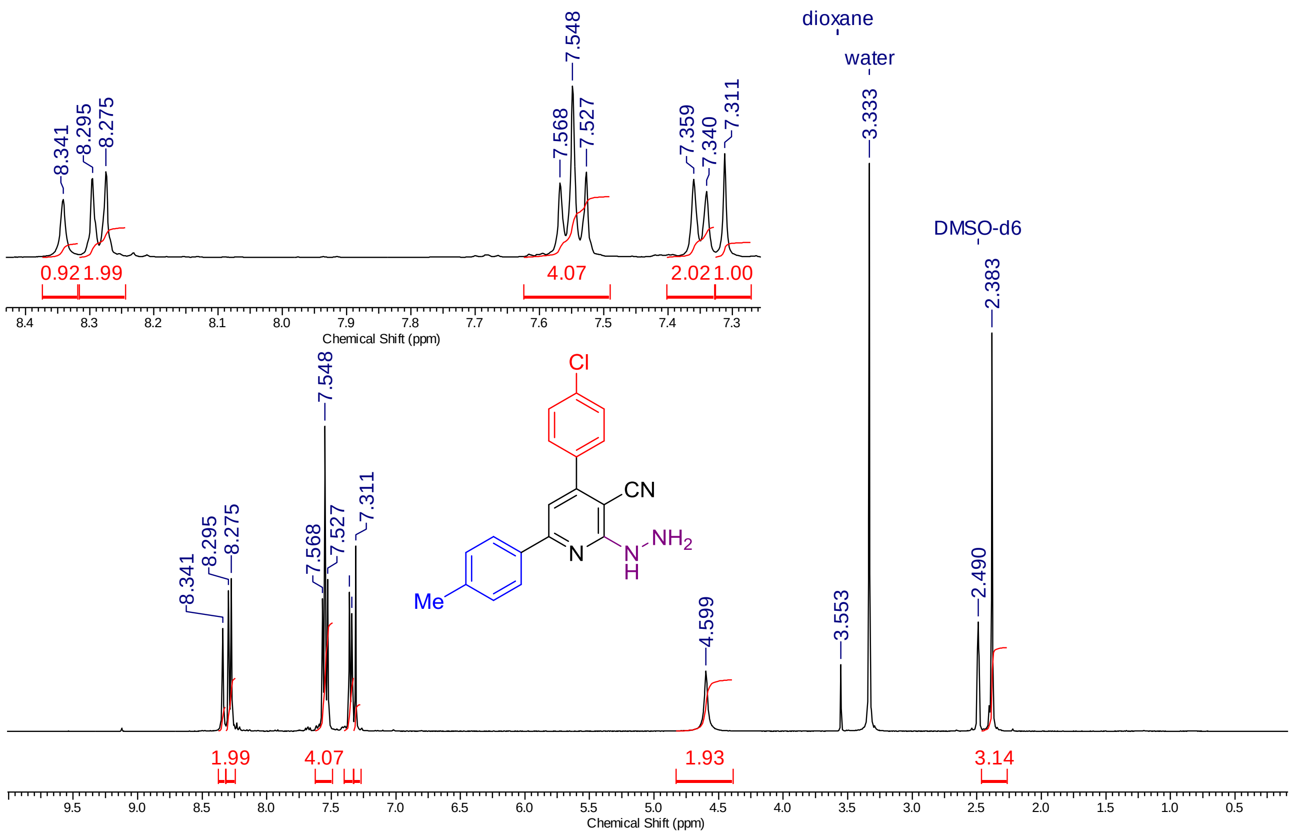

Figure S27. ^{13}C NMR spectrum of 2-hydrazino-3-cyanopyridine 20e, DMSO-d_6 (101 MHz)

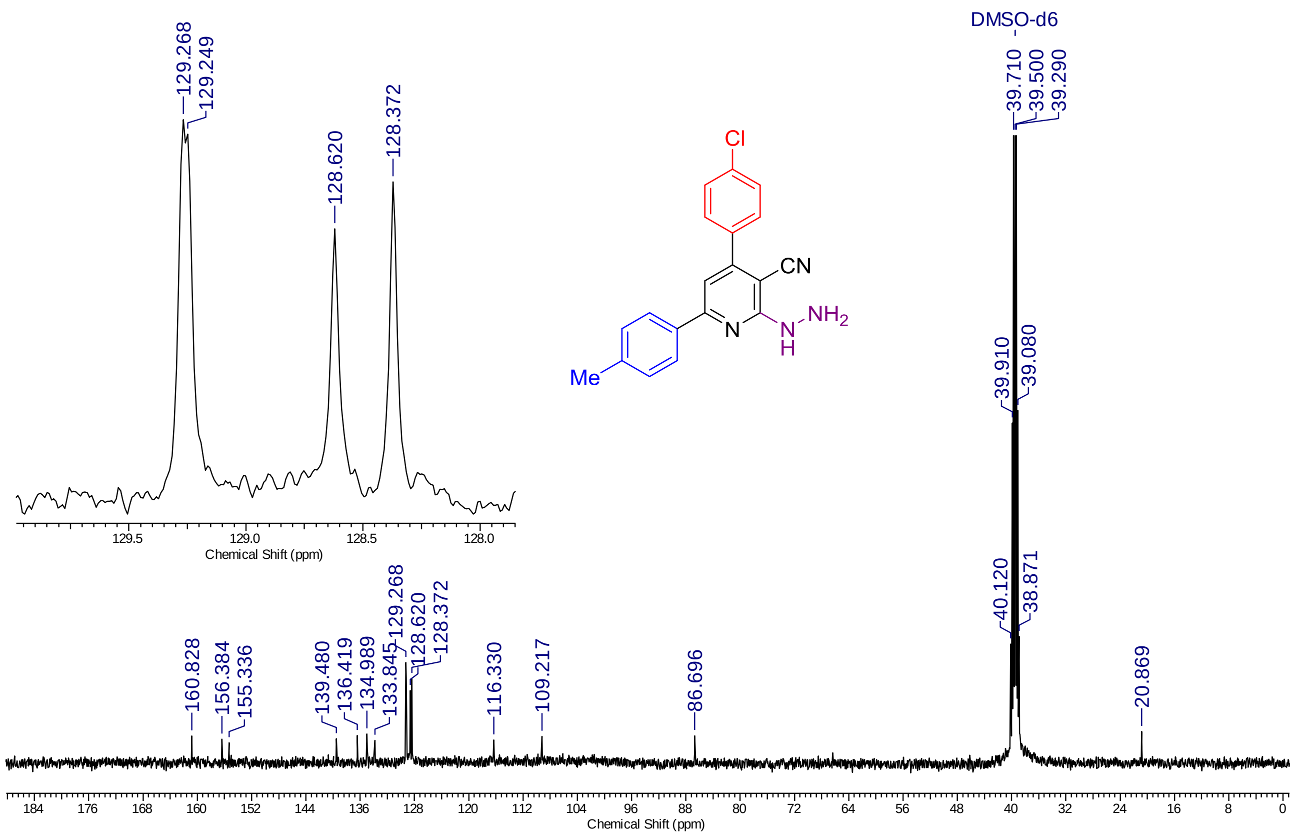

Figure S28. HRMS spectrum of 2-hydrazino-3-cyanopyridine 20e

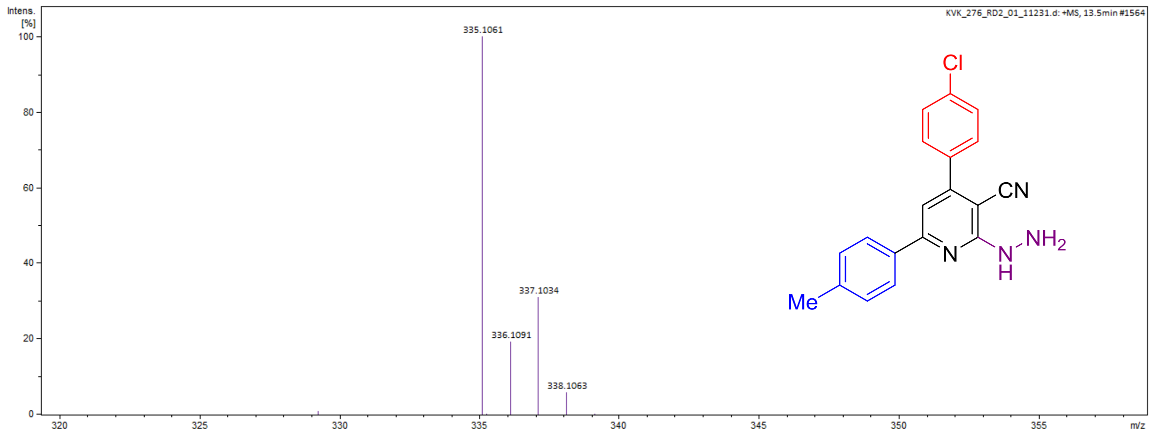

Figure S29. FTIR spectrum (nujol) of 2-hydrazino-3-cyanopyridine 20f

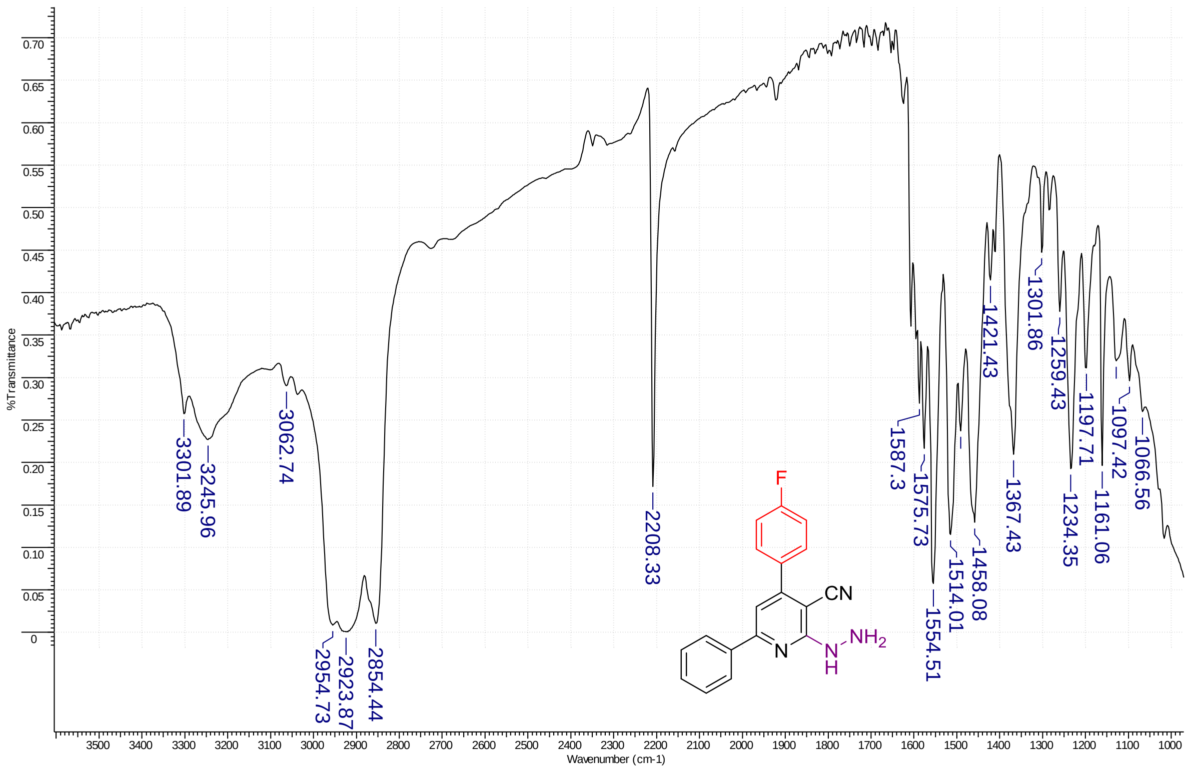

Figure S30. ^{1}H NMR spectrum of 2-hydrazino-3-cyanopyridine 20f, DMSO-d_6 (400 MHz)

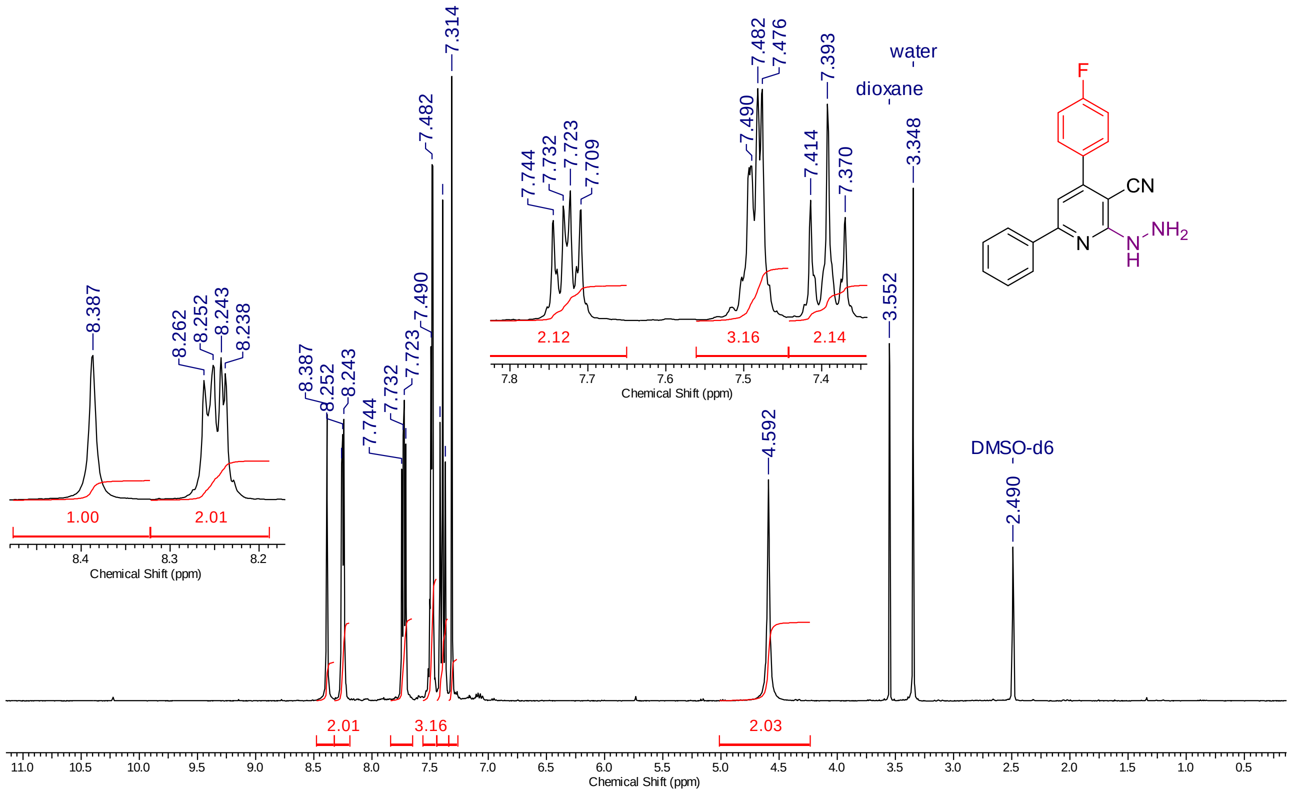

Figure S31. ^{13}C **NMR spectrum of 2-hydrazino-3-cyanopyridine 20f, DMSO-**d_6 **(101 MHz)**

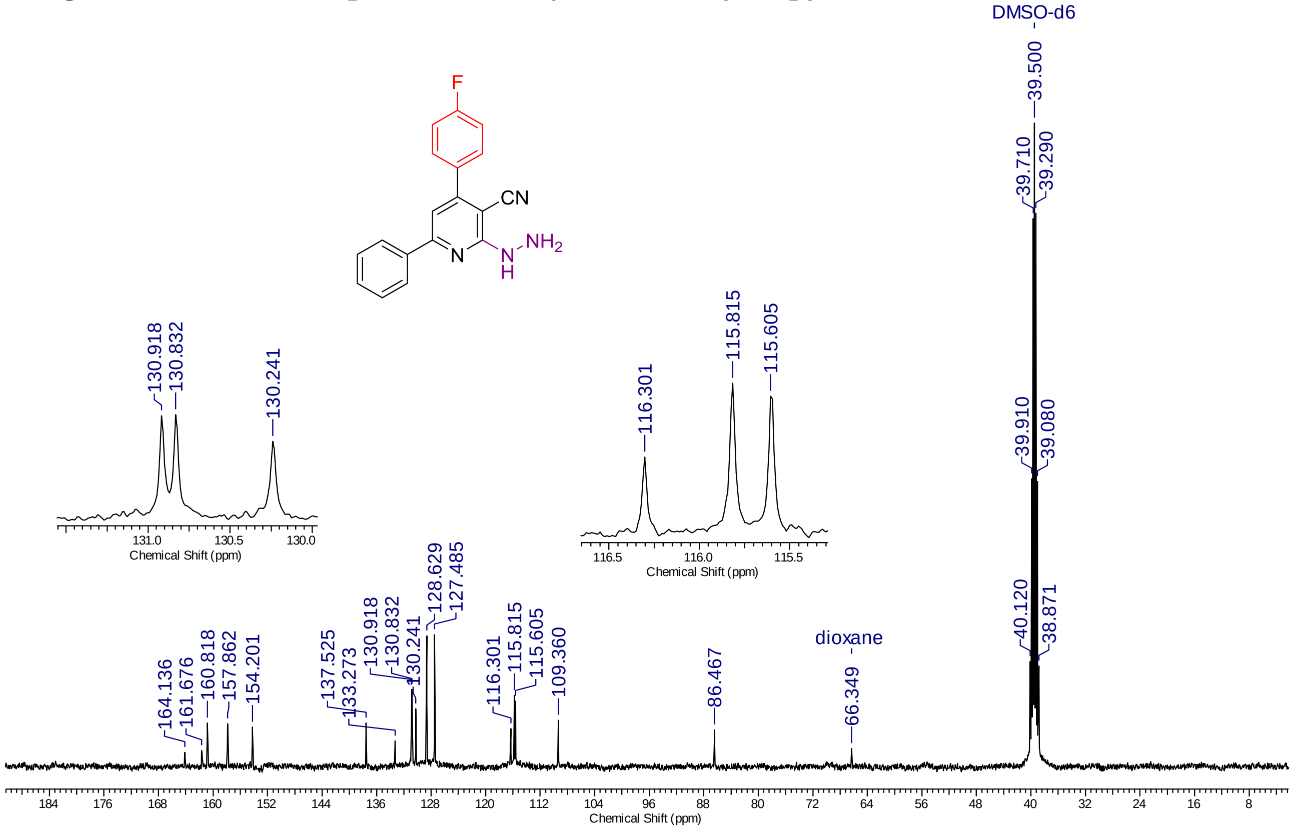

Figure S32. HRMS spectrum of 2-hydrazino-3-cyanopyridine 20f

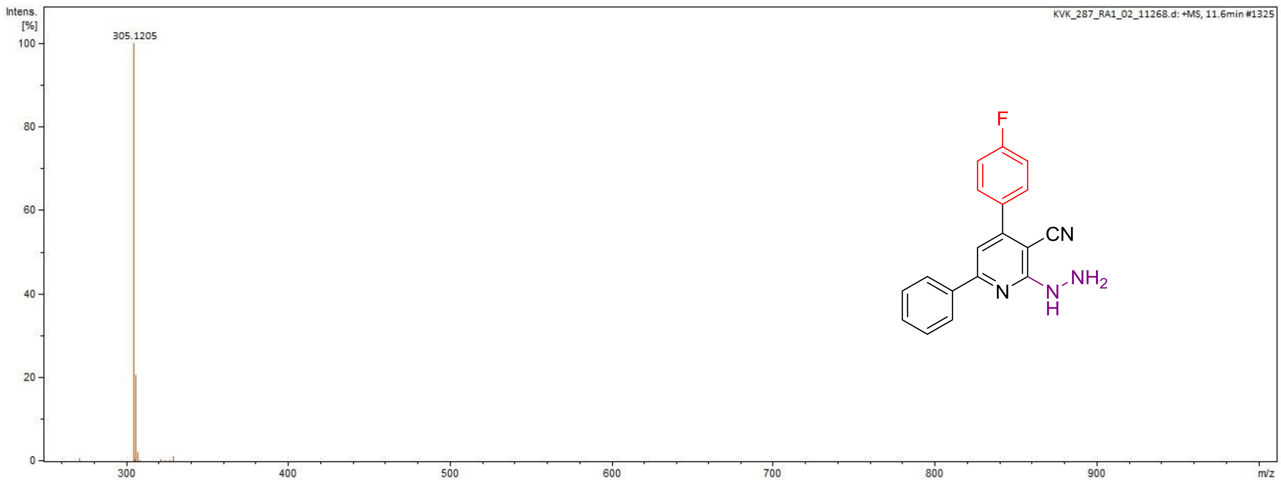

Figure S33. FTIR spectrum (nujol) of 2-hydrazino-3-cyanopyridine 20g

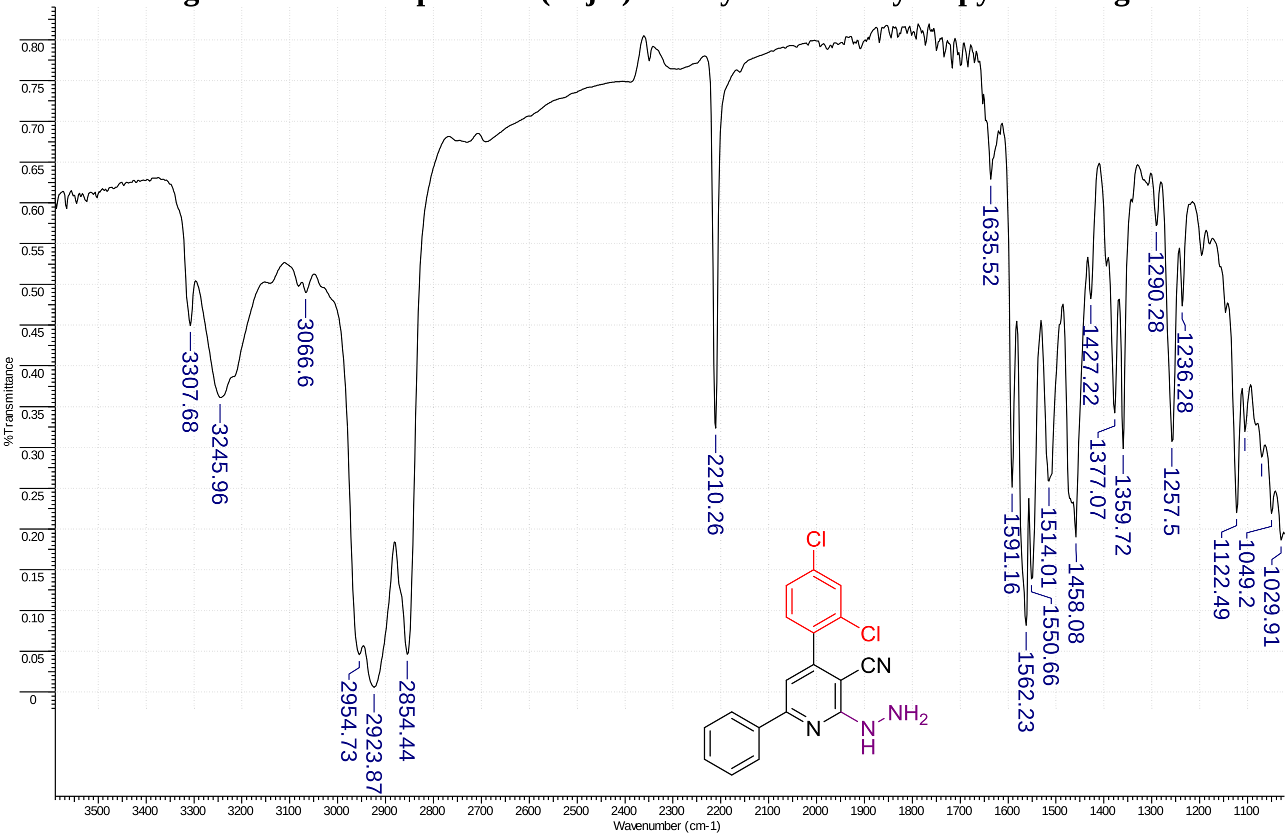

Figure S34. ^{1}H NMR spectrum of 2-hydrazino-3-cyanopyridine 20g, DMSO-d_6 (400 MHz)

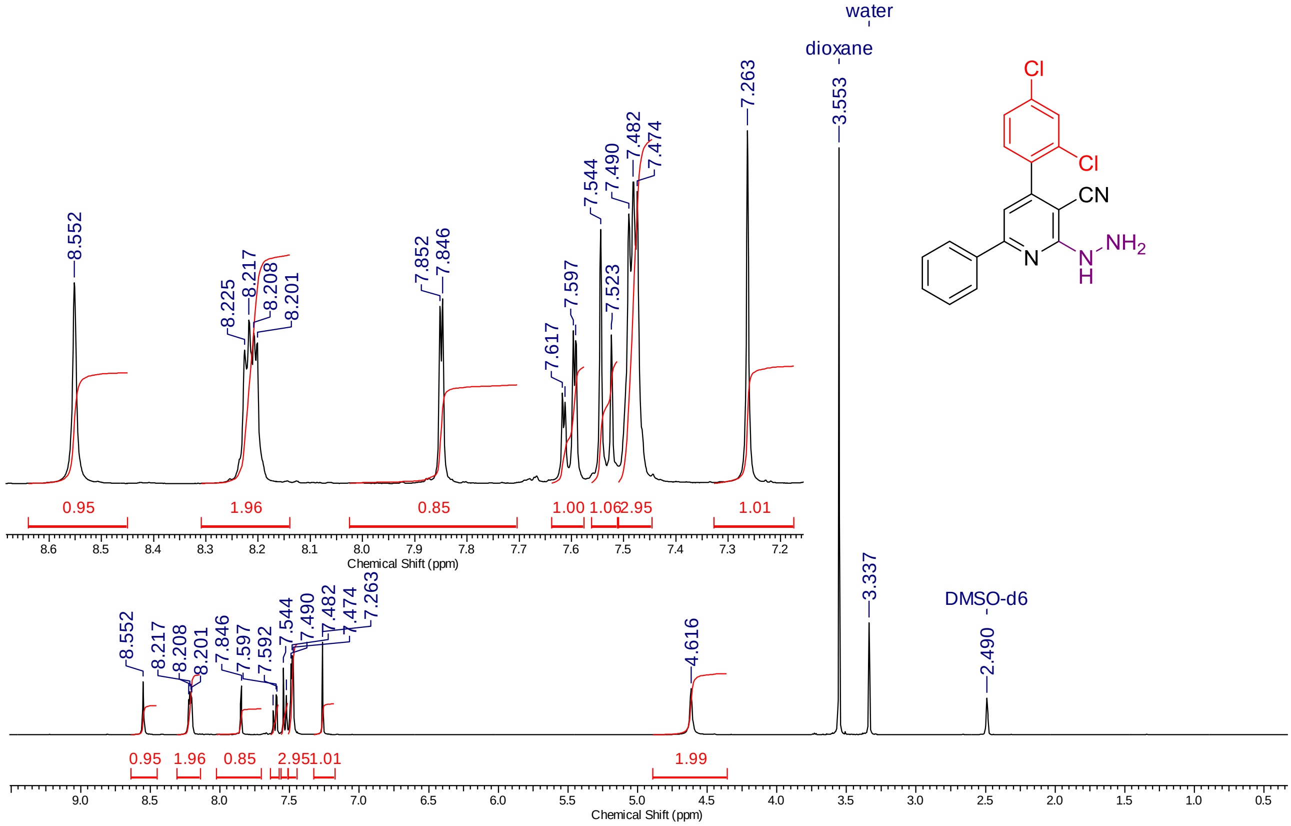

Figure S35. ^{13}C **NMR spectrum of 2-hydrazino-3-cyanopyridine 20g, DMSO-d$_6$ (101 MHz)**

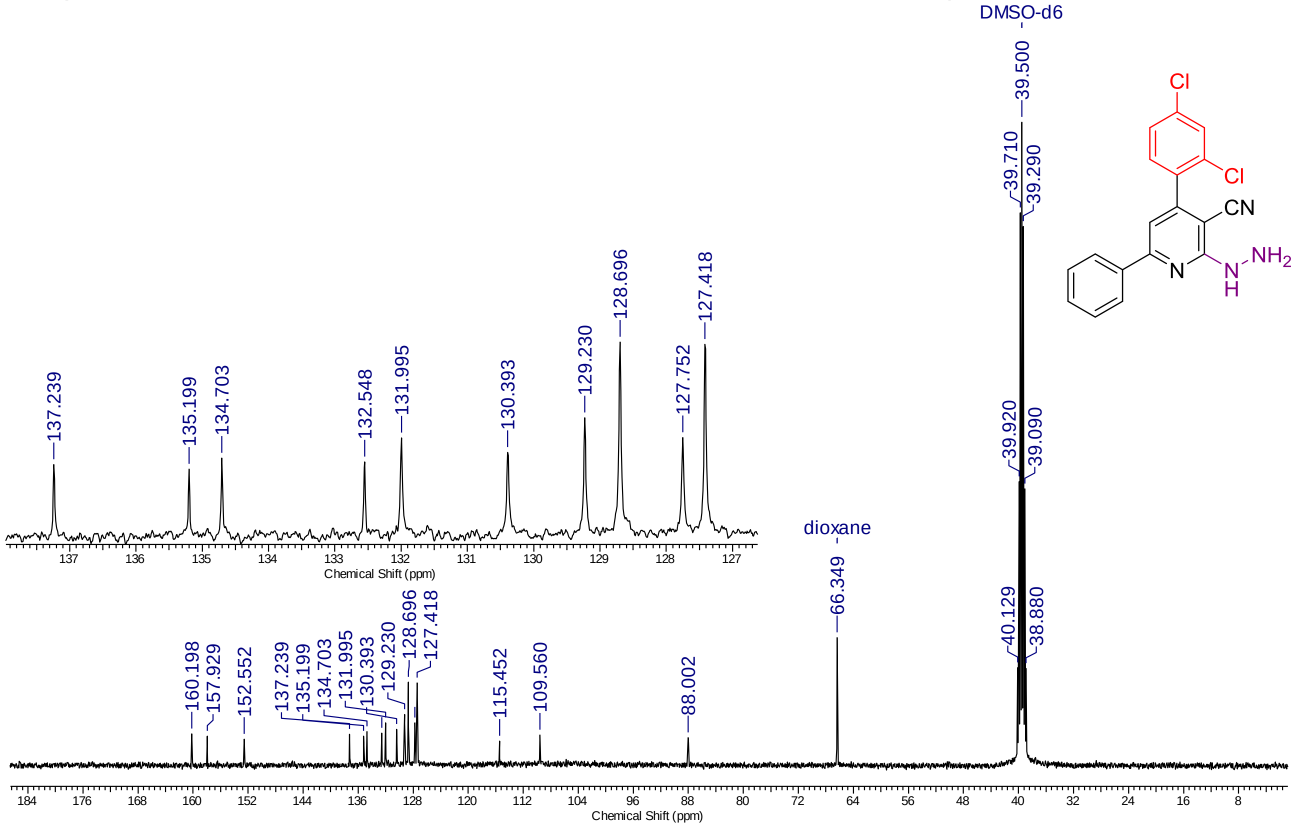

Figure S36. HRMS spectrum of 2-hydrazino-3-cyanopyridine 20g

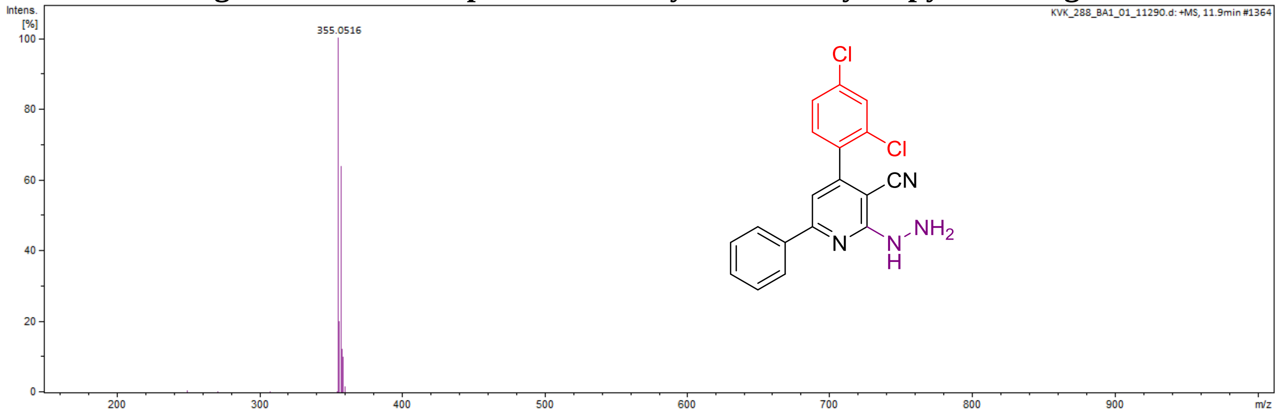

Figure S37. HRMS spectrum of 2-hydrazino-3-cyanopyridine 20h

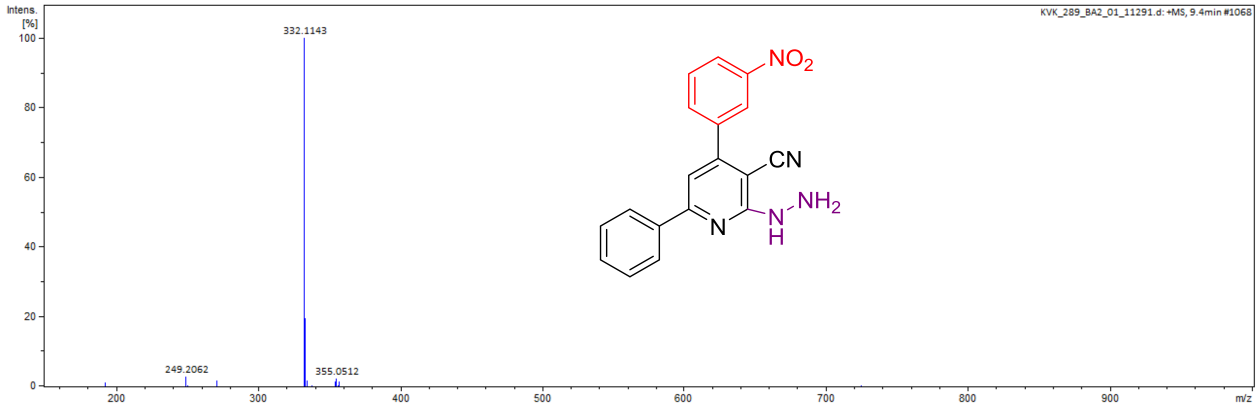

Figure S38. FTIR spectrum (nujol) of 2-hydrazino-3-cyanopyridine 20h

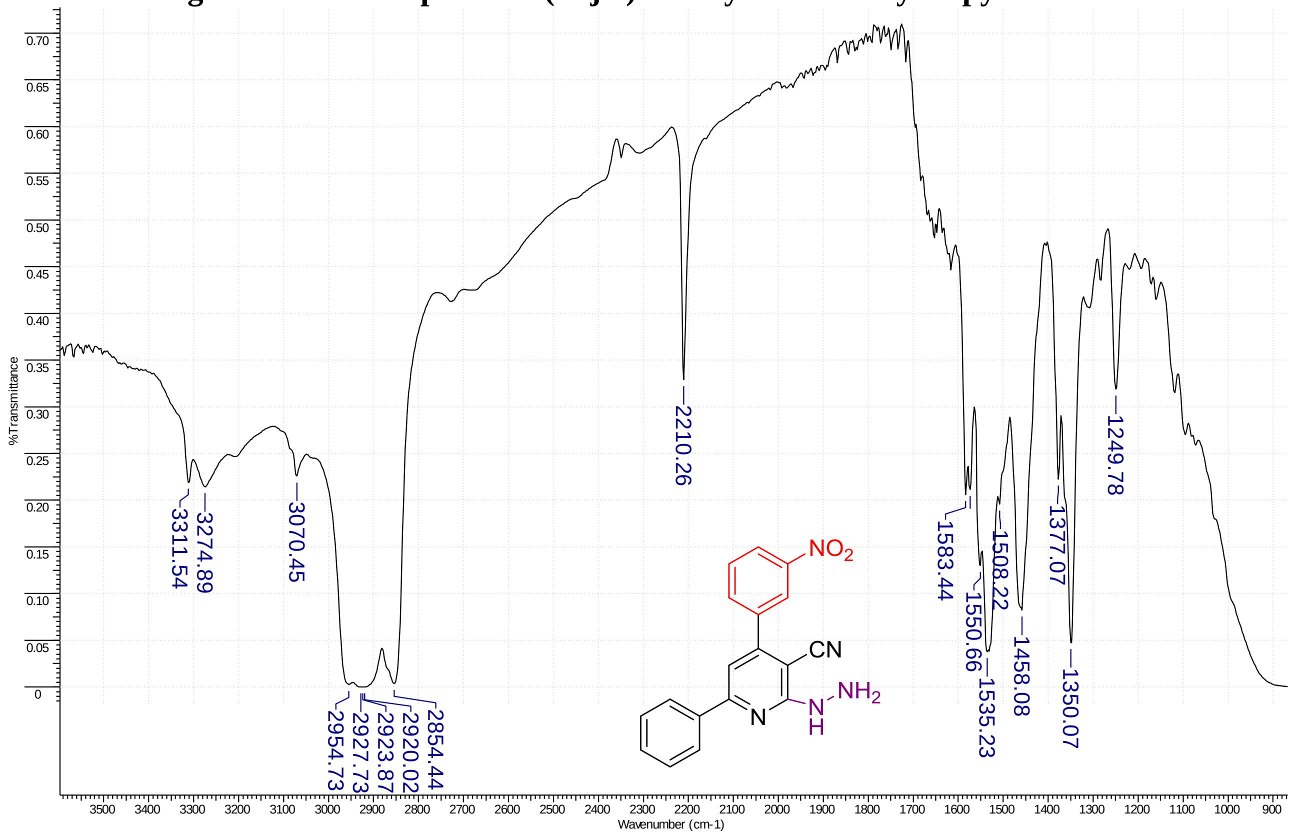

Figure S39. FTIR spectrum (nujol) of 2-hydrazino-3-cyanopyridine 20i

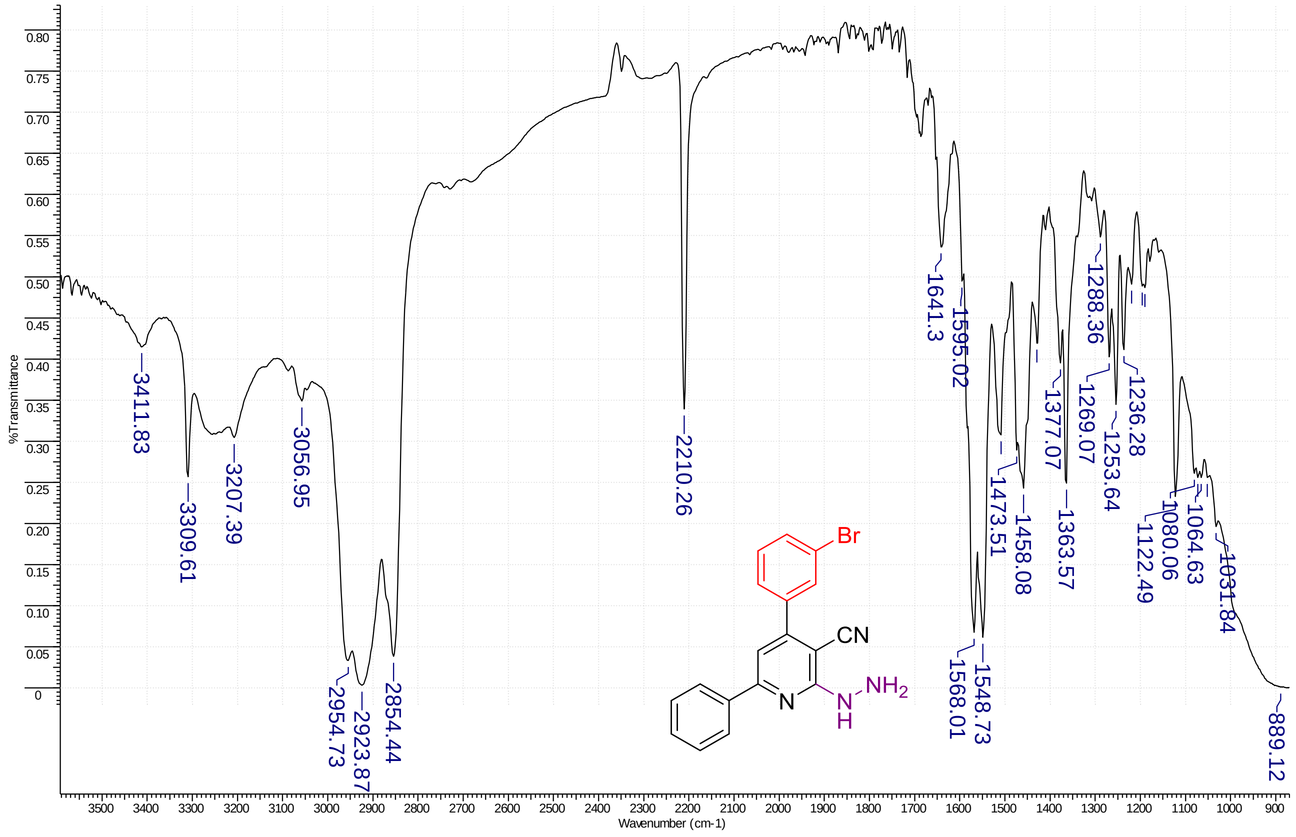

Figure S40. HRMS spectrum of 2-hydrazino-3-cyanopyridine 20i

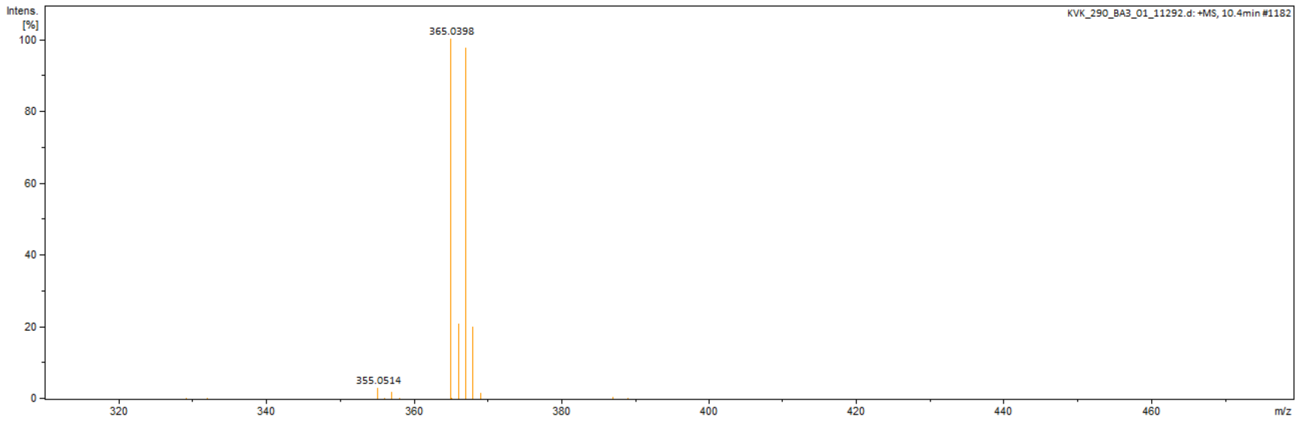

Figure S41. FTIR spectrum (nujol) of 2-hydrazino-3-cyanopyridine 20j

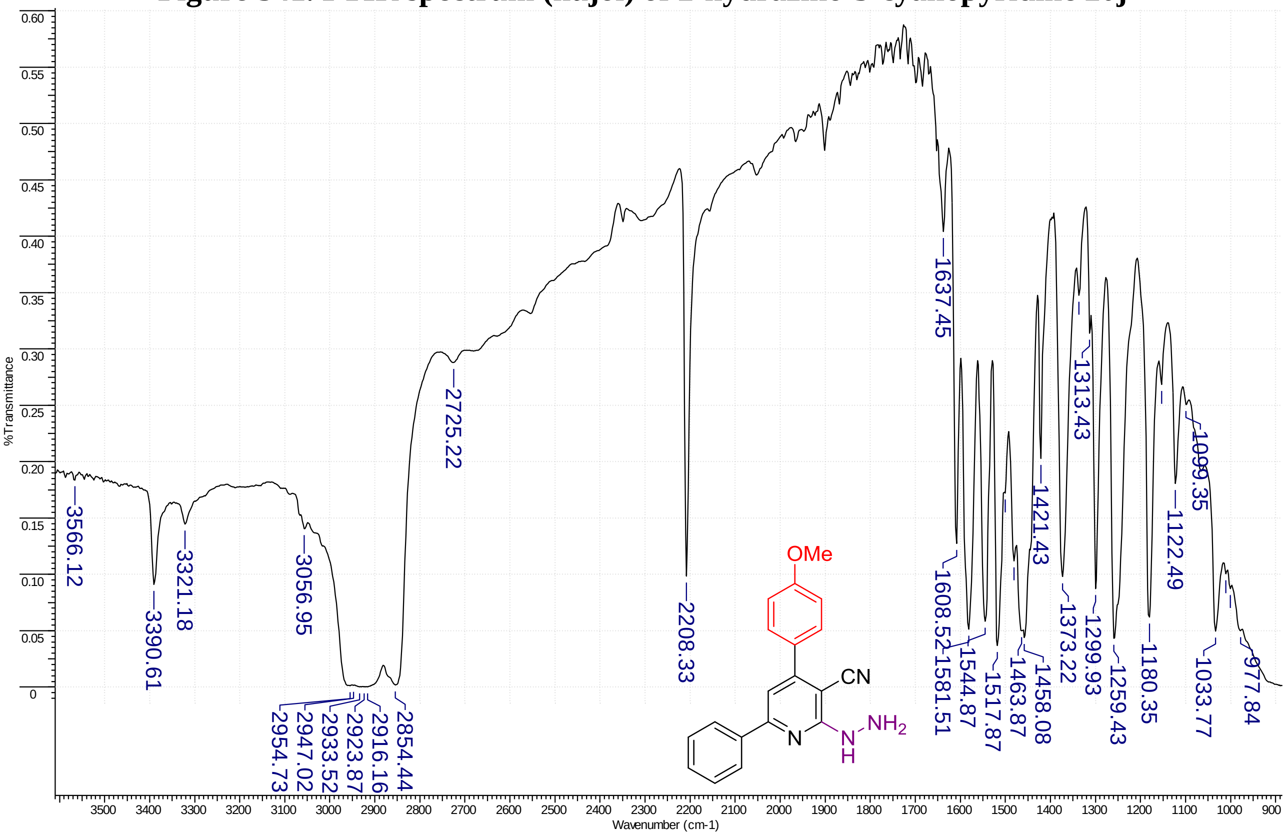

Figure S42. HRMS spectrum of 2-hydrazino-3-cyanopyridine 20j

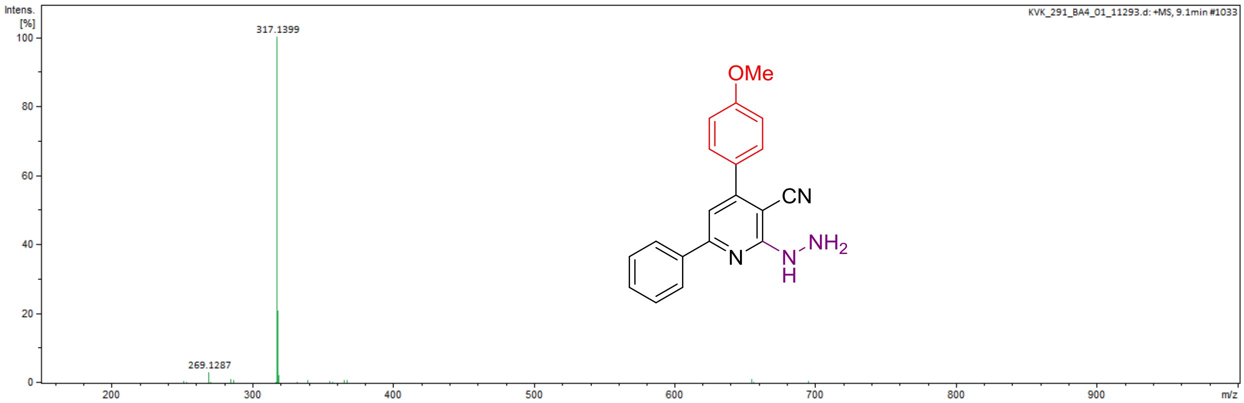

Figure S43. ¹H NMR spectrum of 2-hydrazino-3-cyanopyridine 20j, DMSO-d_6 (400 MHz)

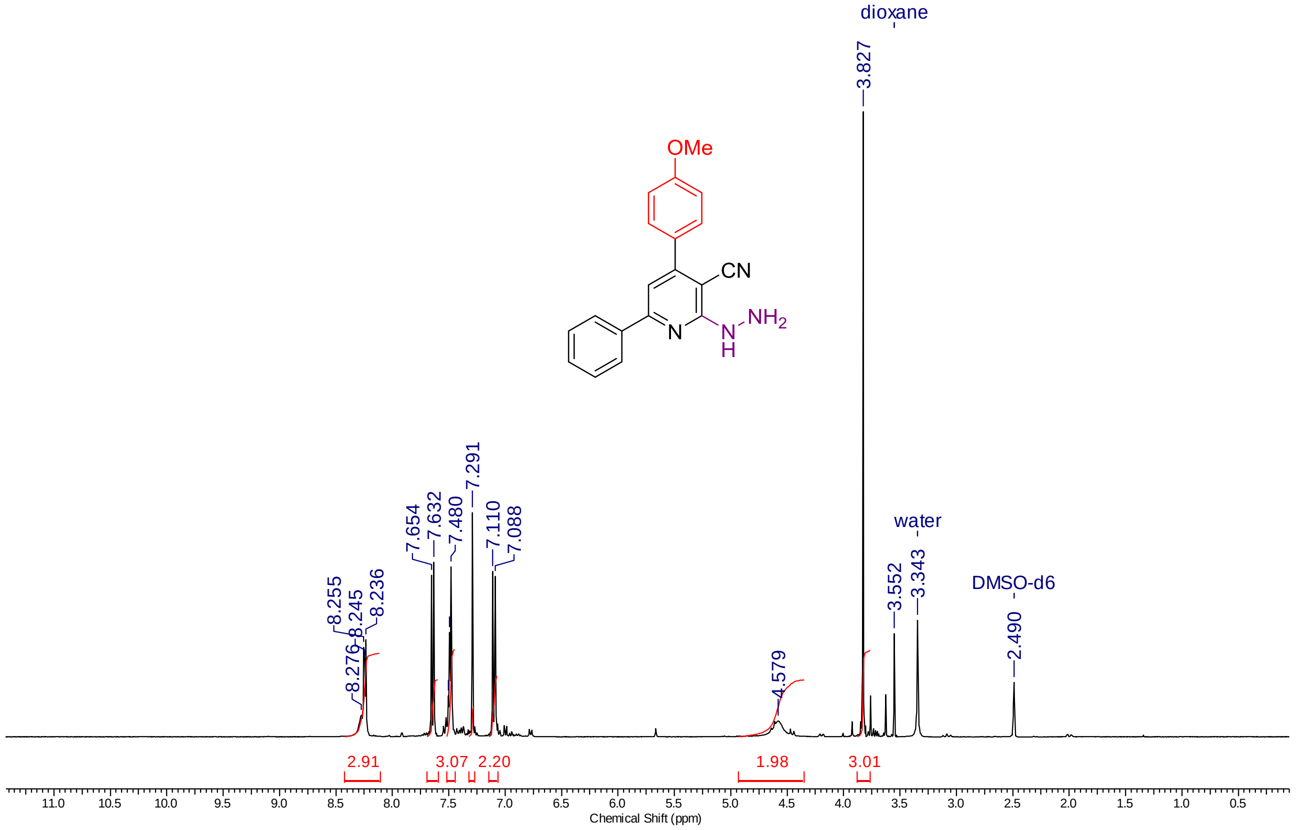

Figure S44. ¹³C NMR spectrum of 2-hydrazino-3-cyanopyridine 20j, DMSO-d_6 (101 MHz)

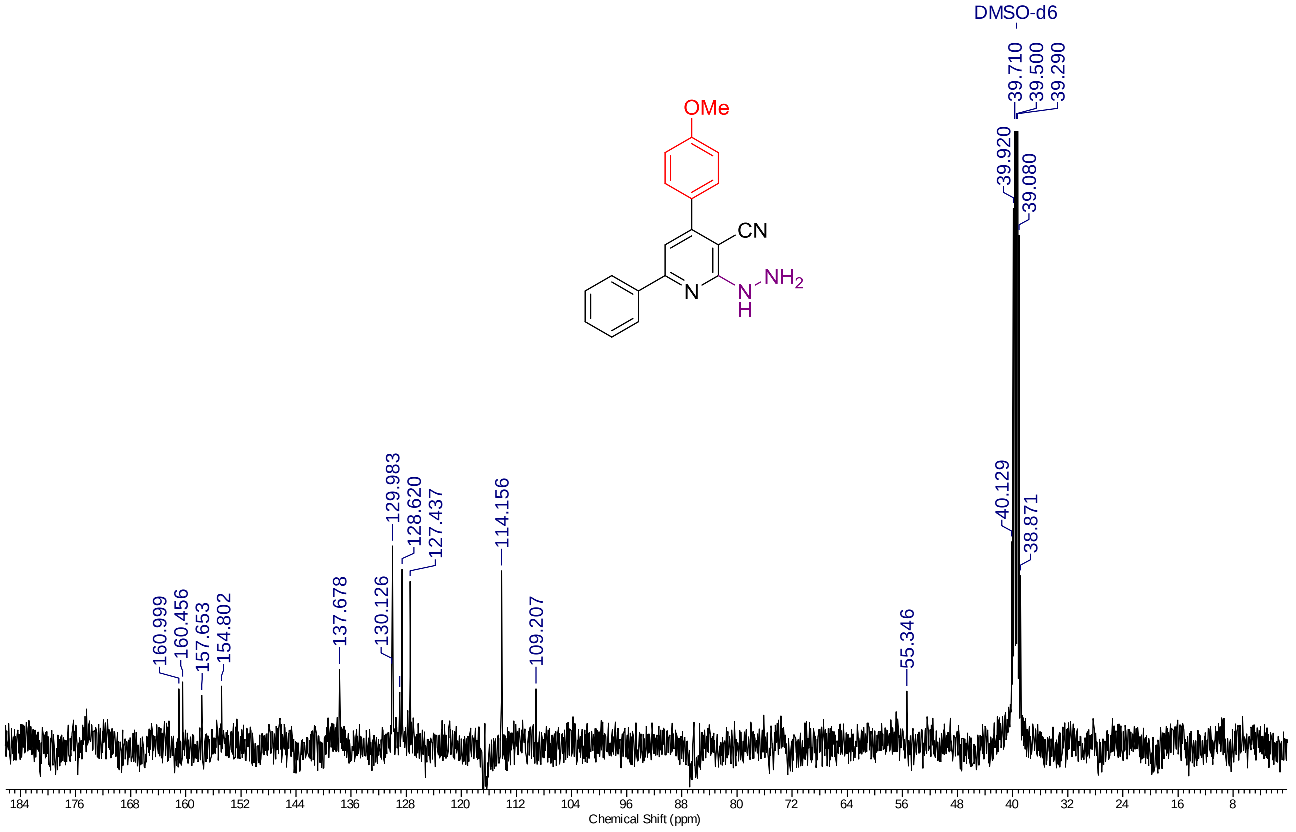

Figure S45. FTIR spectrum (nujol) of hydrazone 21{*1*}

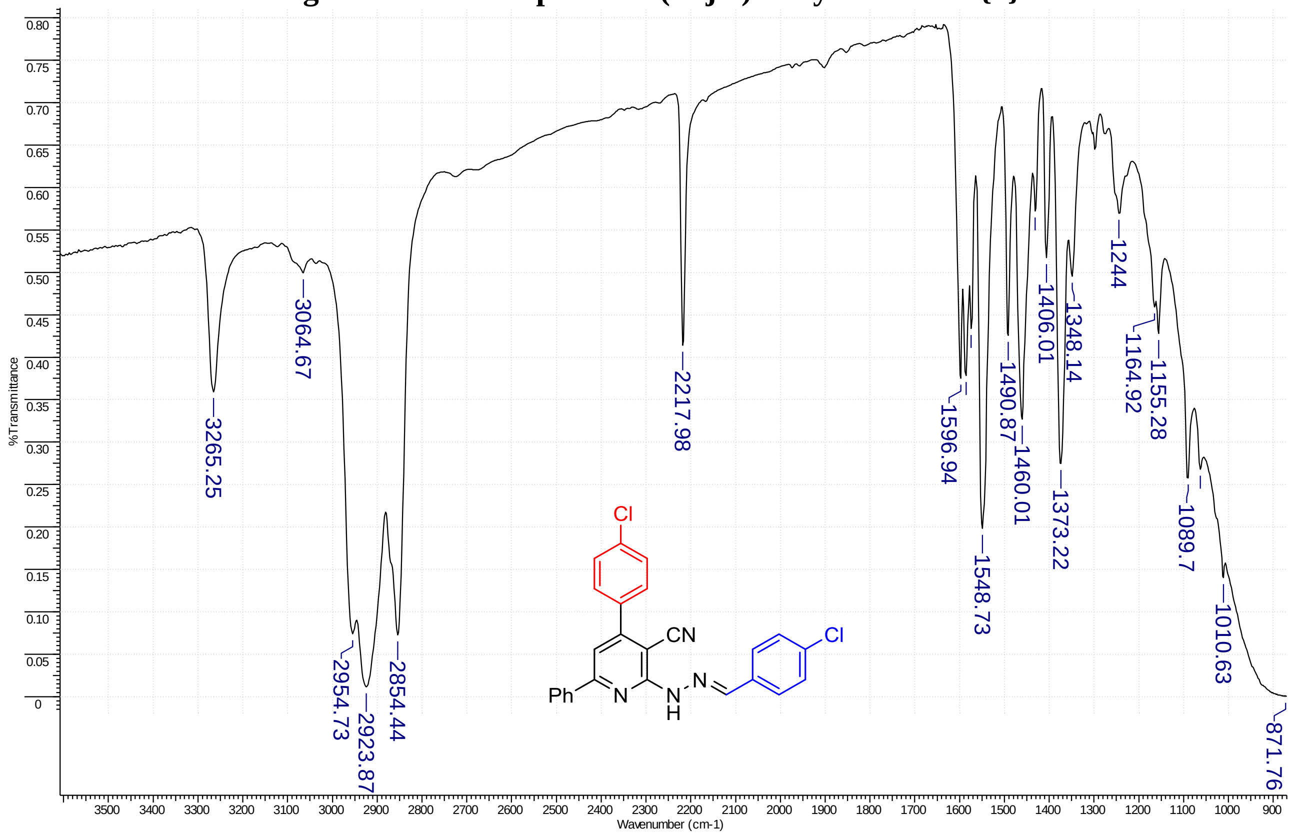

Figure S46. ^{1}H NMR spectrum of hydrazone 21{*1*}, DMSO-d_6 (400 MHz)

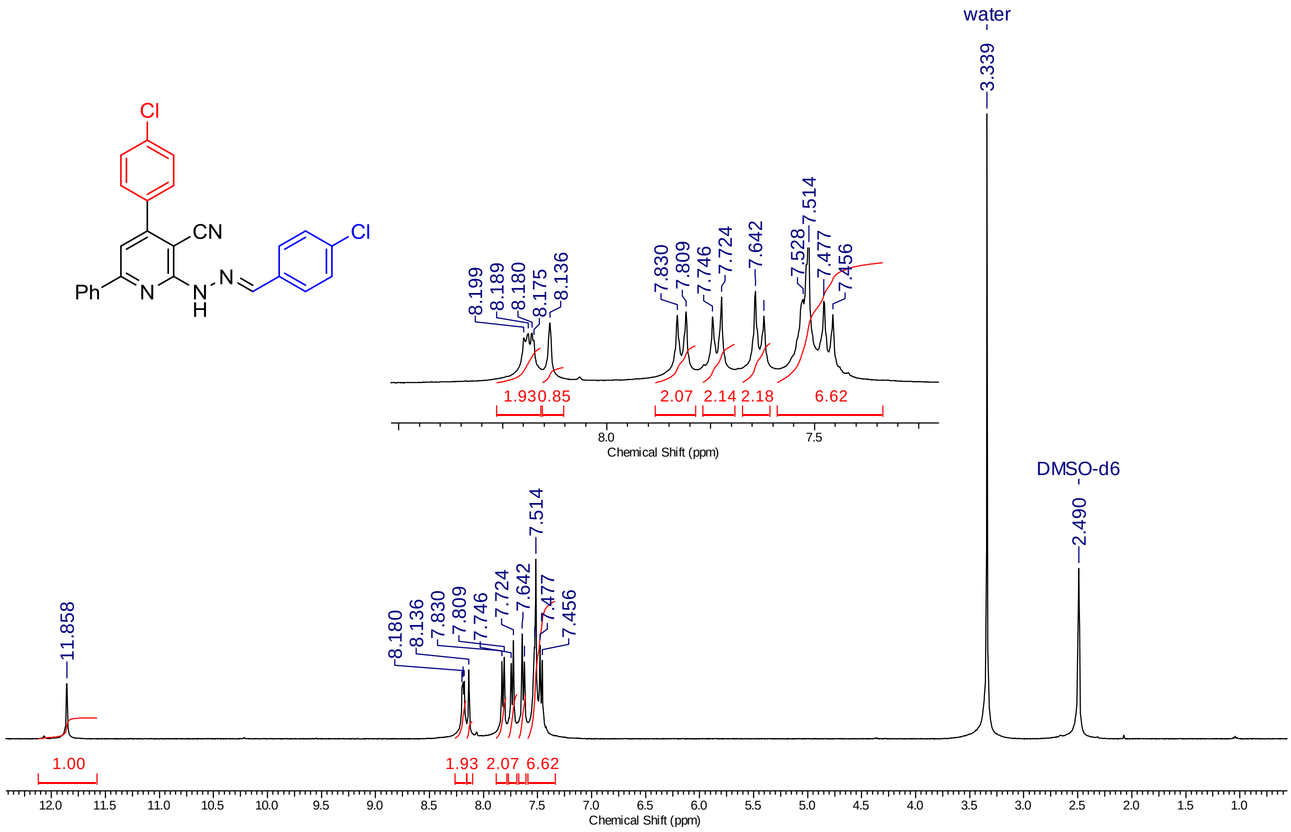

Figure S47. HRMS spectrum of hydrazone 21{*1*}

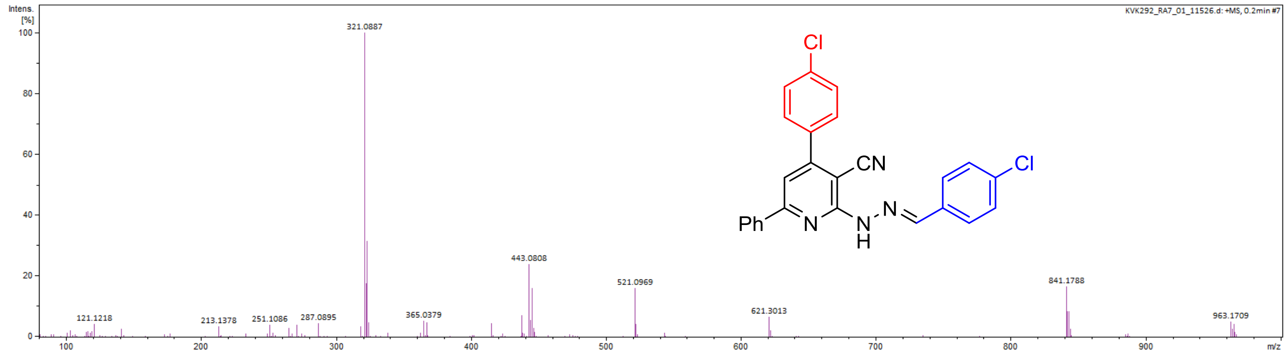

Figure S48. FTIR spectrum (nujol) of hydrazone 21{*3*}

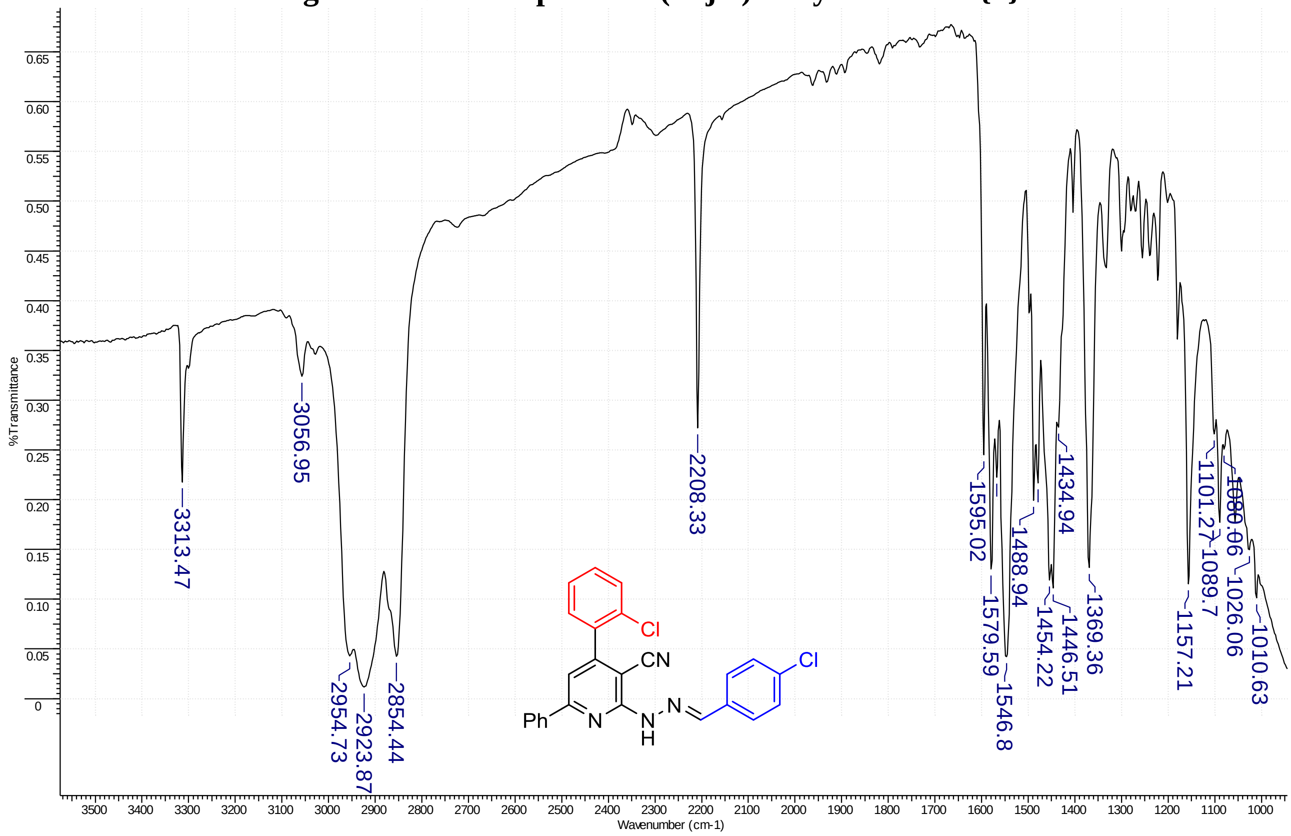

Figure S49. HRMS spectrum of hydrazone 21{*3*}

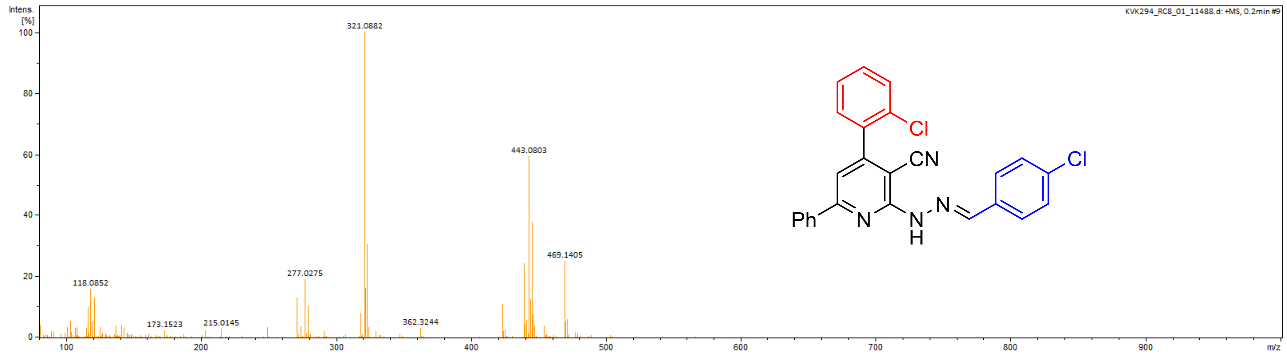

Figure S50. ^{1}H **NMR spectrum of hydrazone 21{*3*}, DMSO-d$_6$ (400 MHz)**

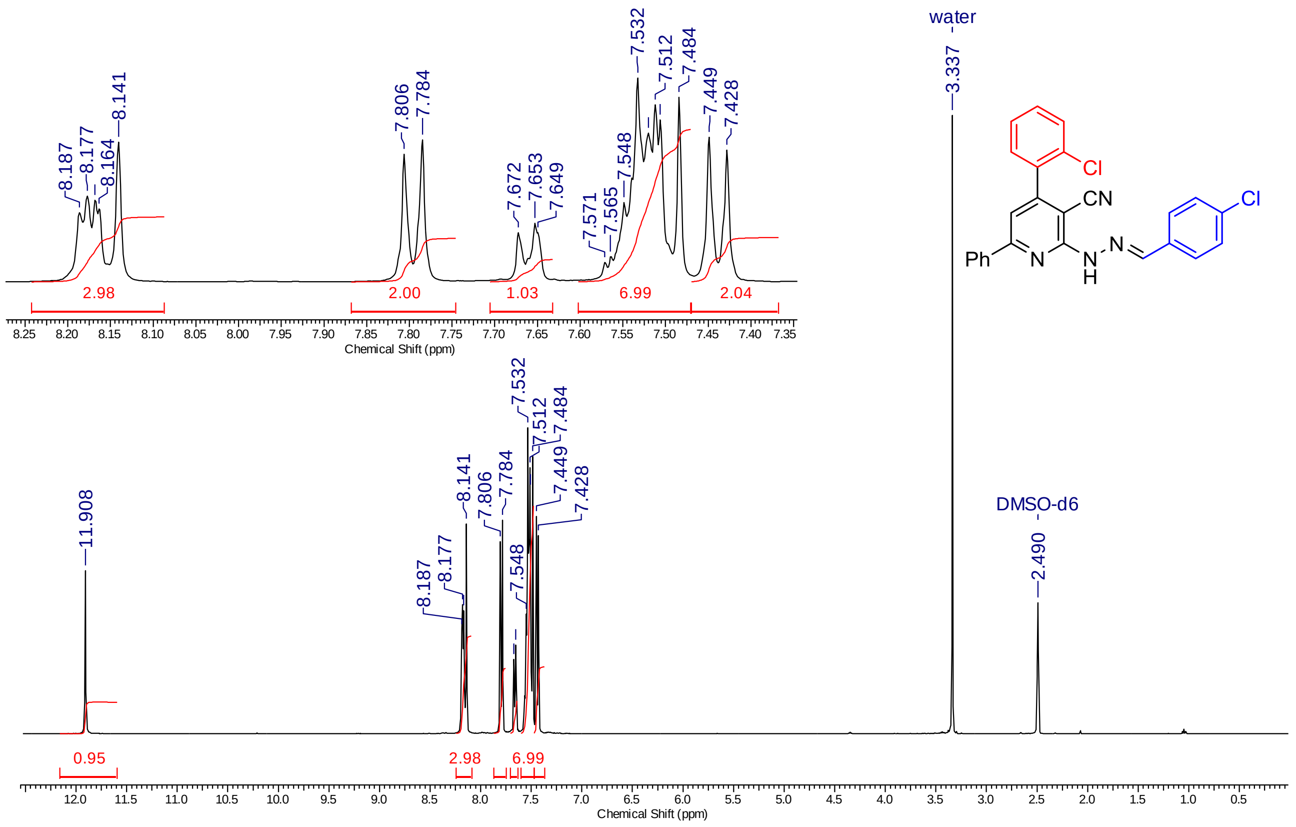

Figure S51. ^{13}C **NMR spectrum of hydrazone 21{*3*}, DMSO-d$_6$ (101 MHz)**

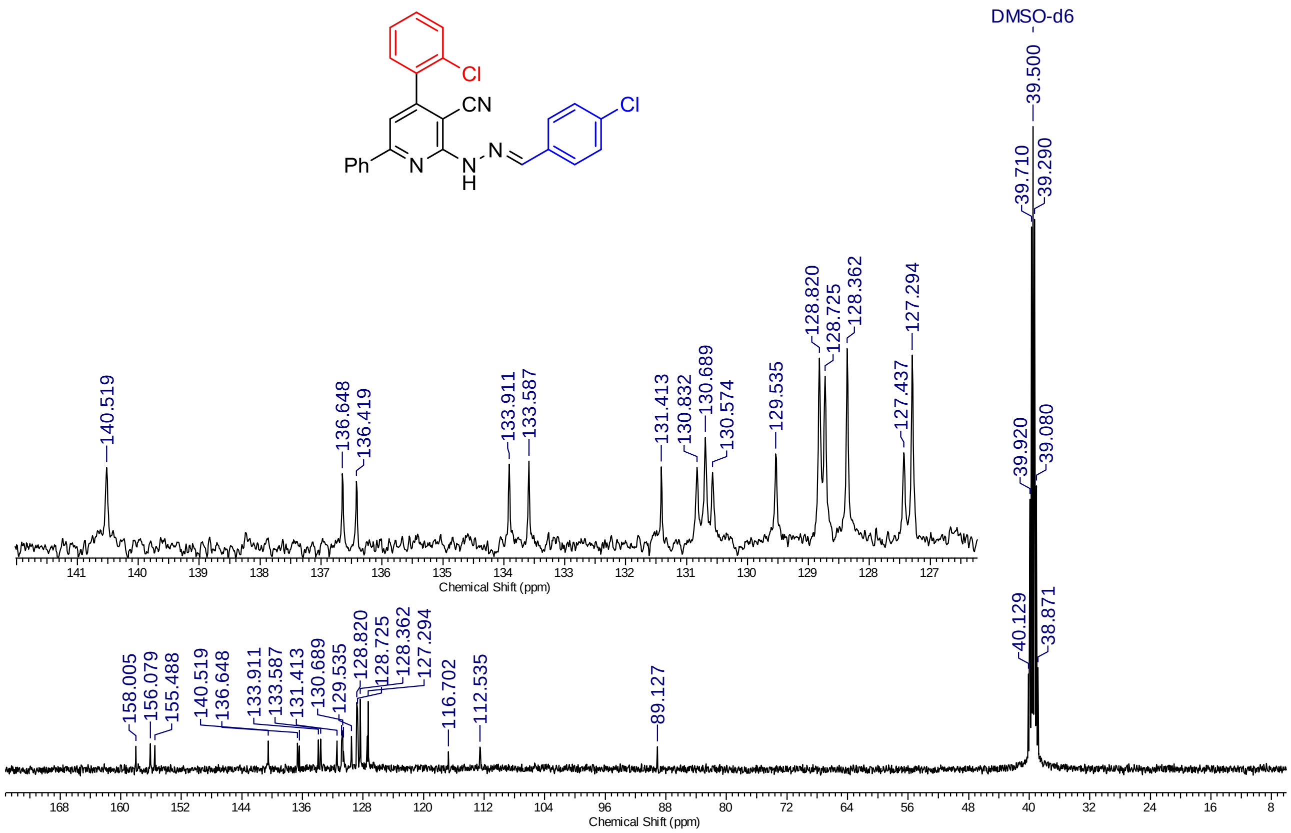

Figure S52. FTIR spectrum (nujol) of hydrazone 21{*4*}

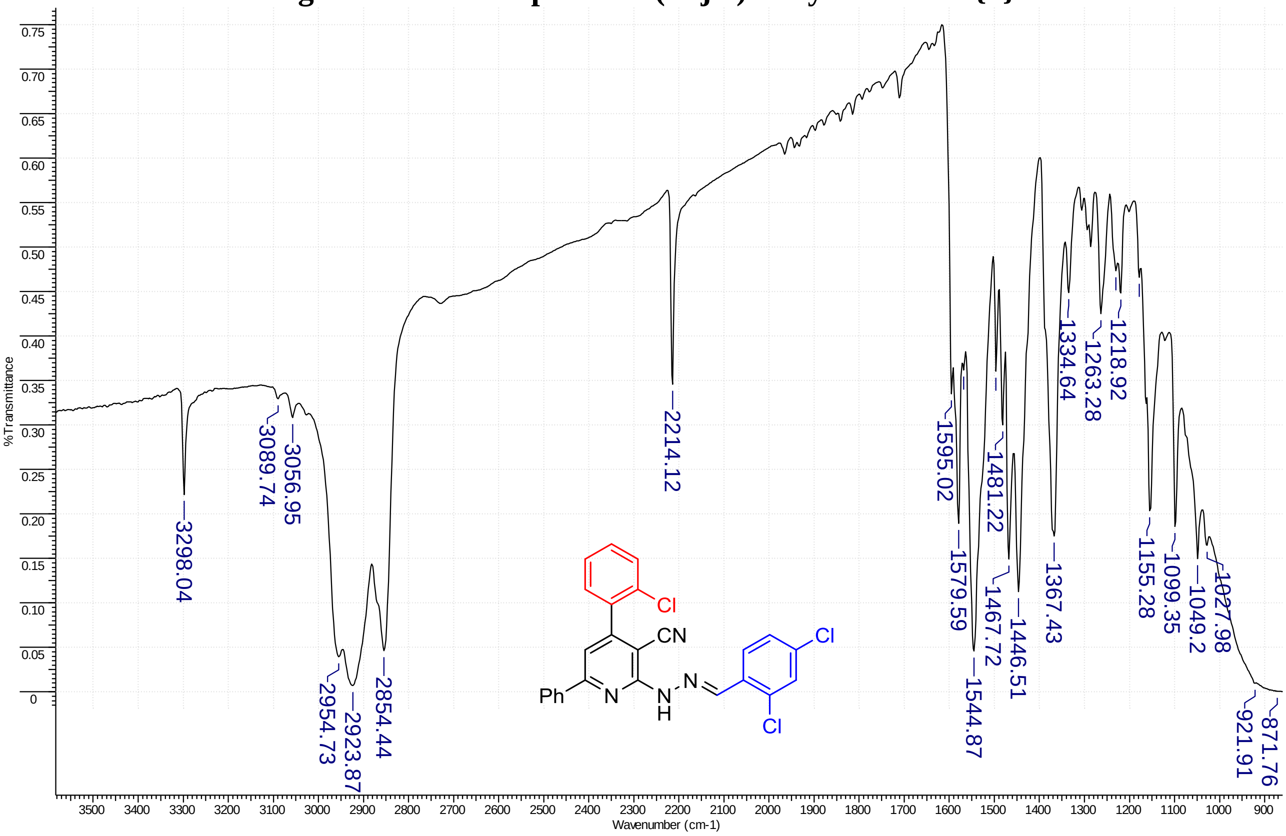

Figure S53. ^{1}H NMR spectrum of hydrazone 21{*4*}, acetone-d$_6$ (400 MHz)

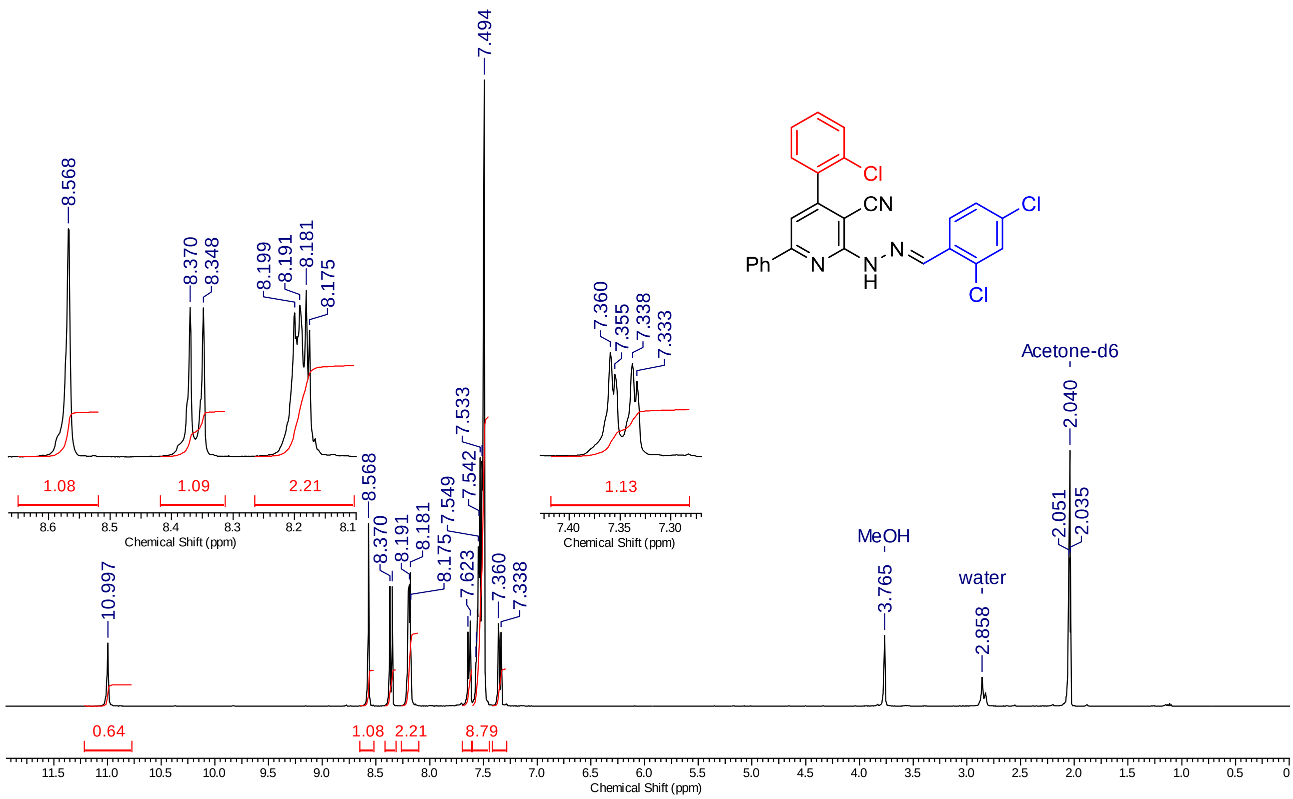

Figure S54. ^{13}C NMR spectrum of hydrazone 21{*4*}, DMSO-d_6 (101 MHz)

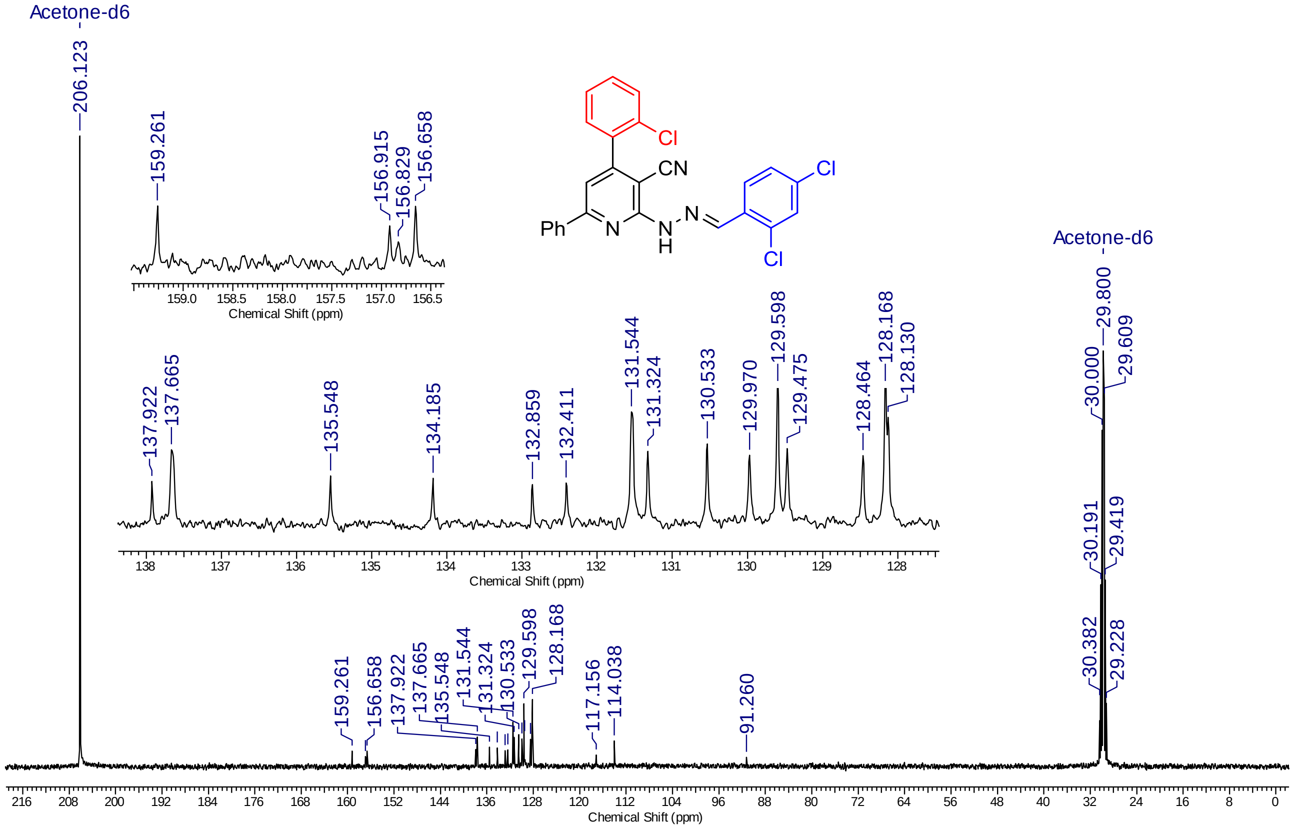

Figure S55. HRMS spectrum of hydrazone 21{*4*}

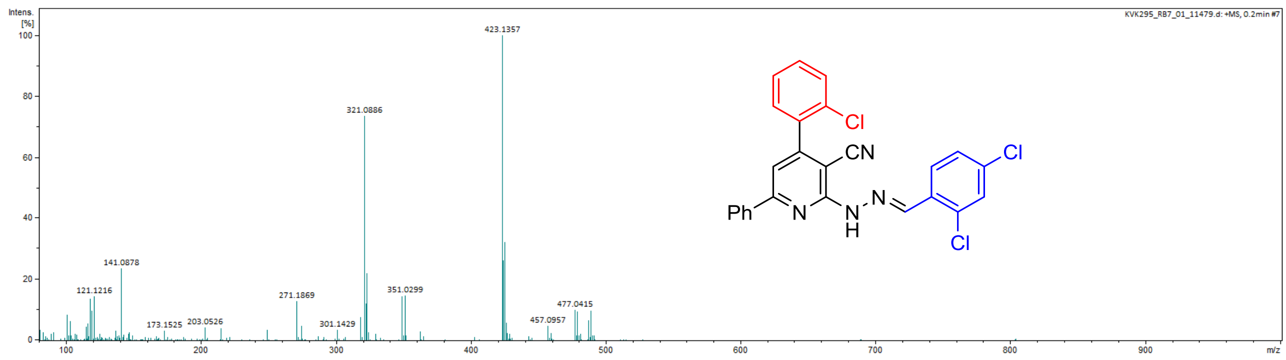

Figure S56. HRMS spectrum of hydrazone 21{*5*}

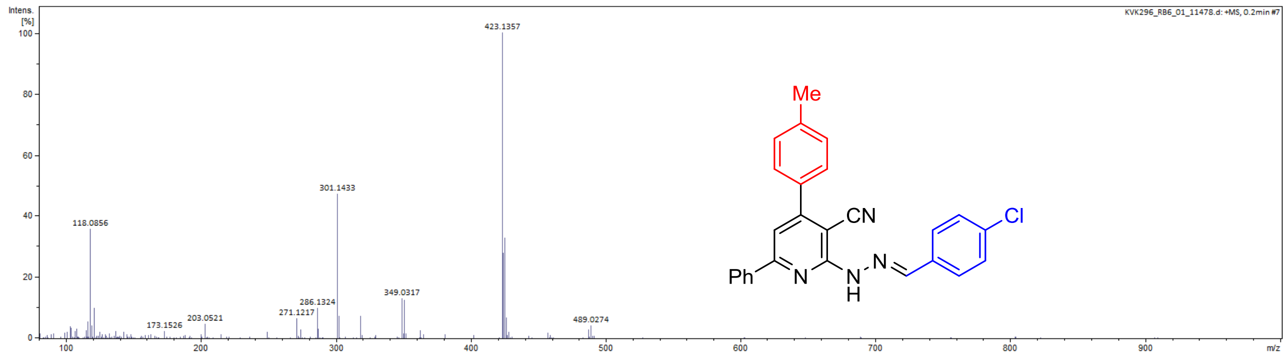

Figure S57. FTIR spectrum (nujol) of hydrazone 21{*5*}

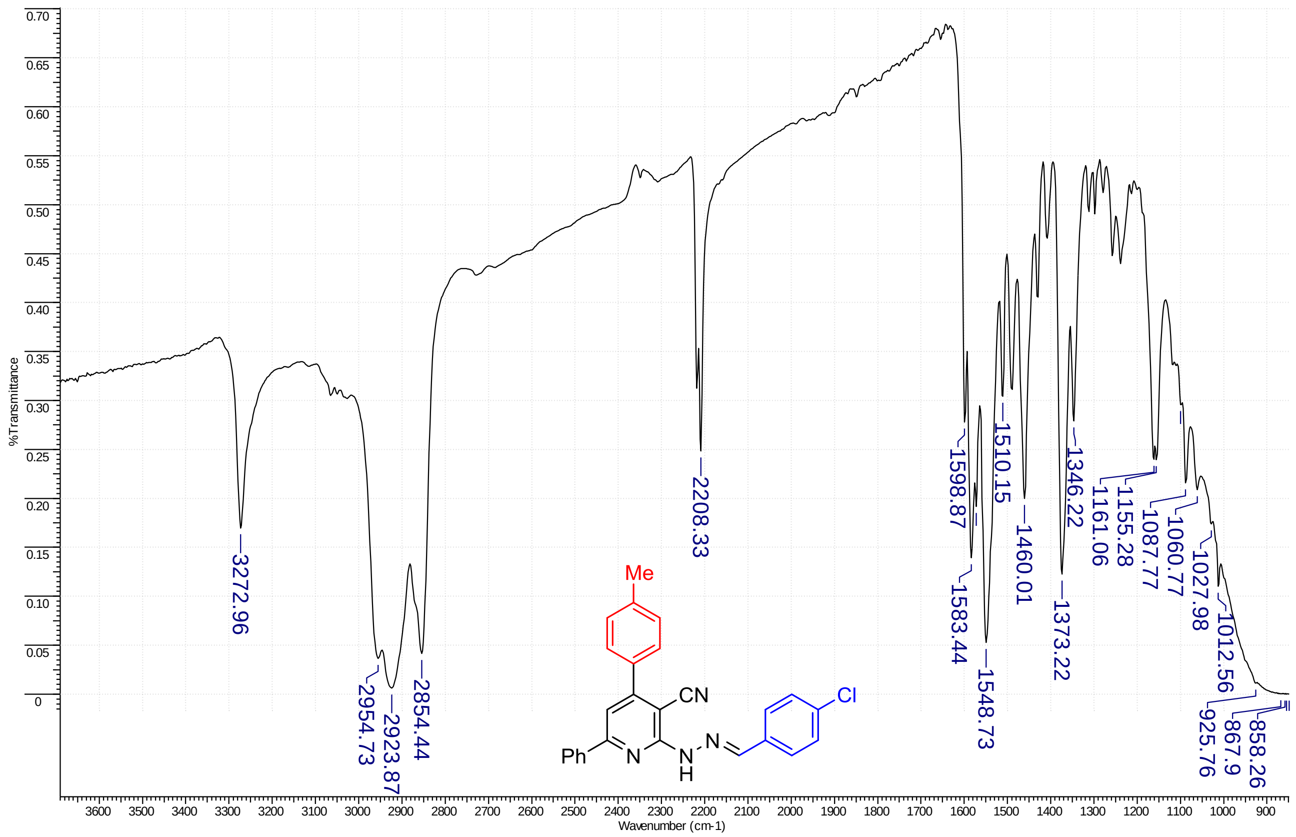

Figure S58. FTIR spectrum (nujol) of hydrazone 21{*6*}

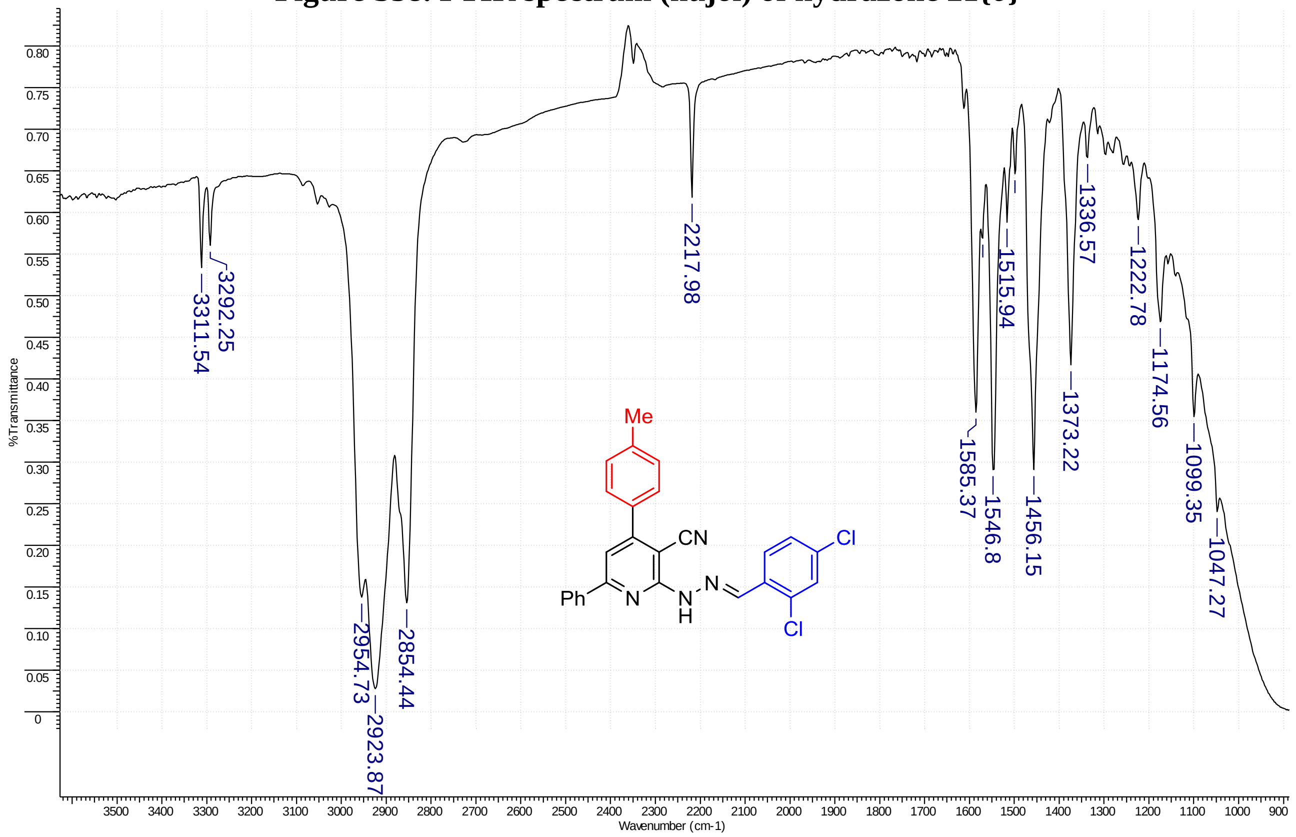

Figure S59. ^{1}H NMR spectrum of hydrazone 21{*6*}, DMSO-d_6 (400 MHz)

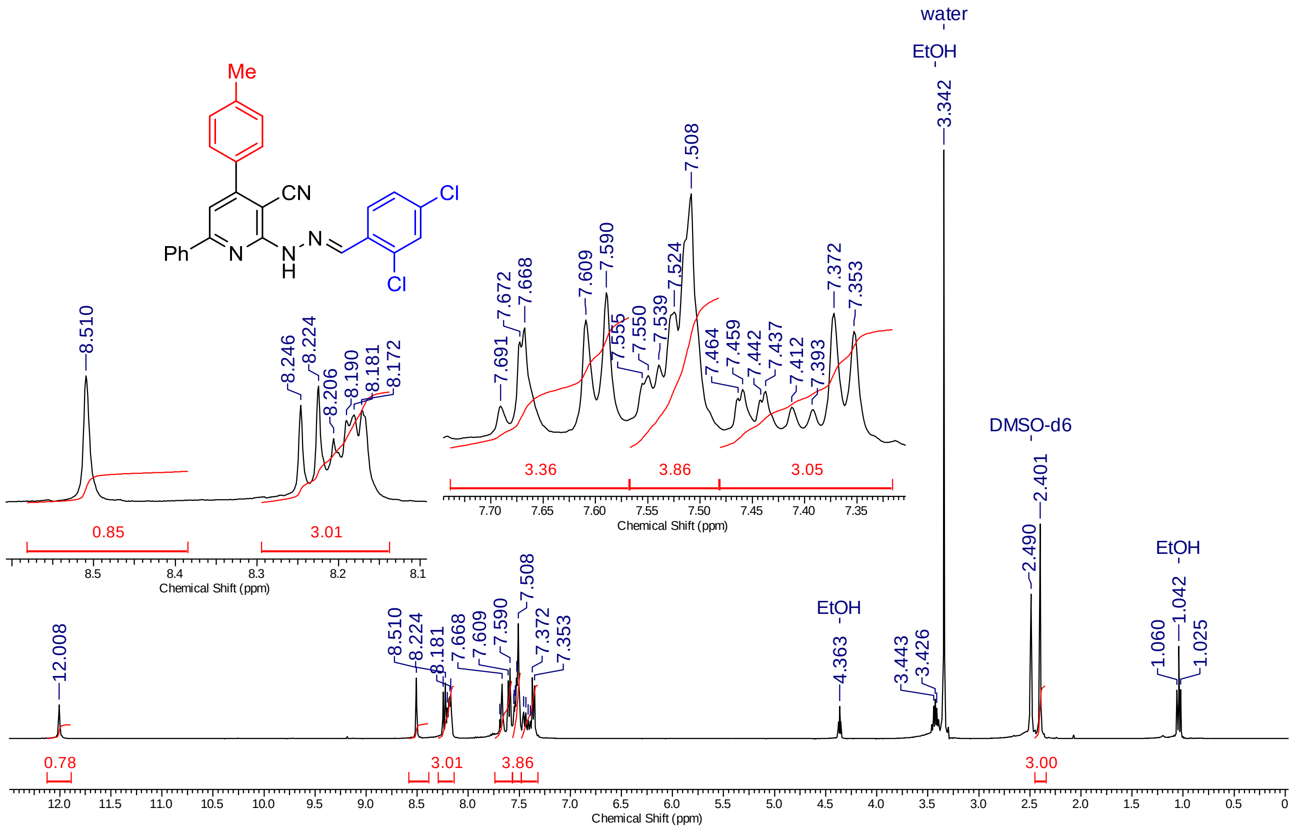

Figure S60. HRMS spectrum of hydrazone 21{*6*}

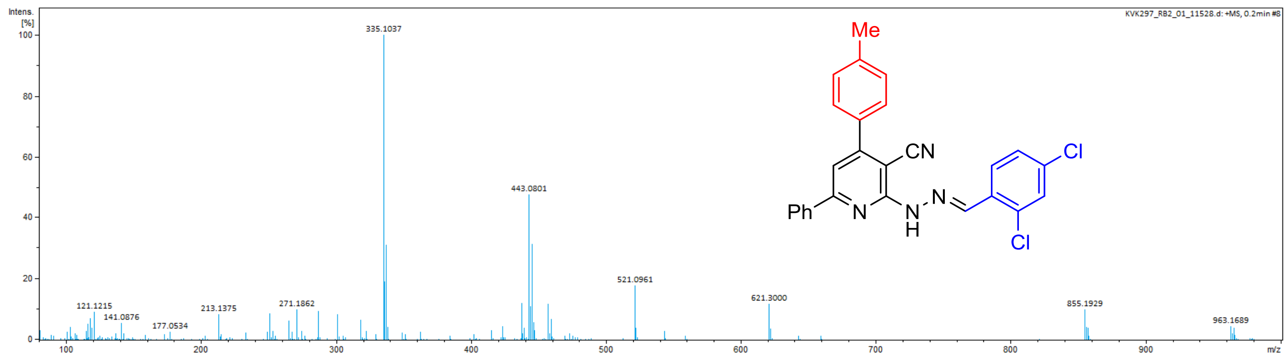

Figure S61. FTIR spectrum (nujol) of hydrazone 21{*7*}

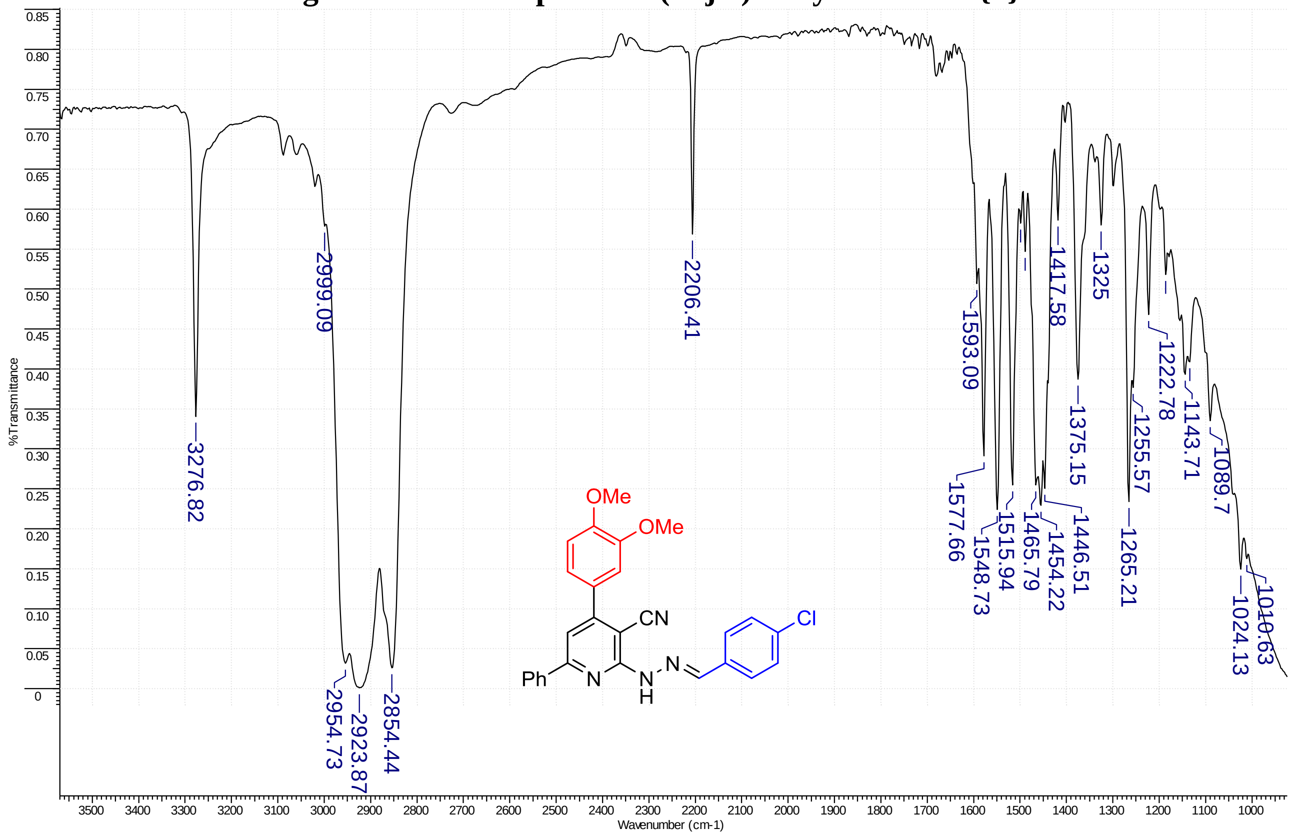

Figure S62. ^{1}H NMR spectrum of hydrazone 21{*7*}, DMSO-d_6 (400 MHz)

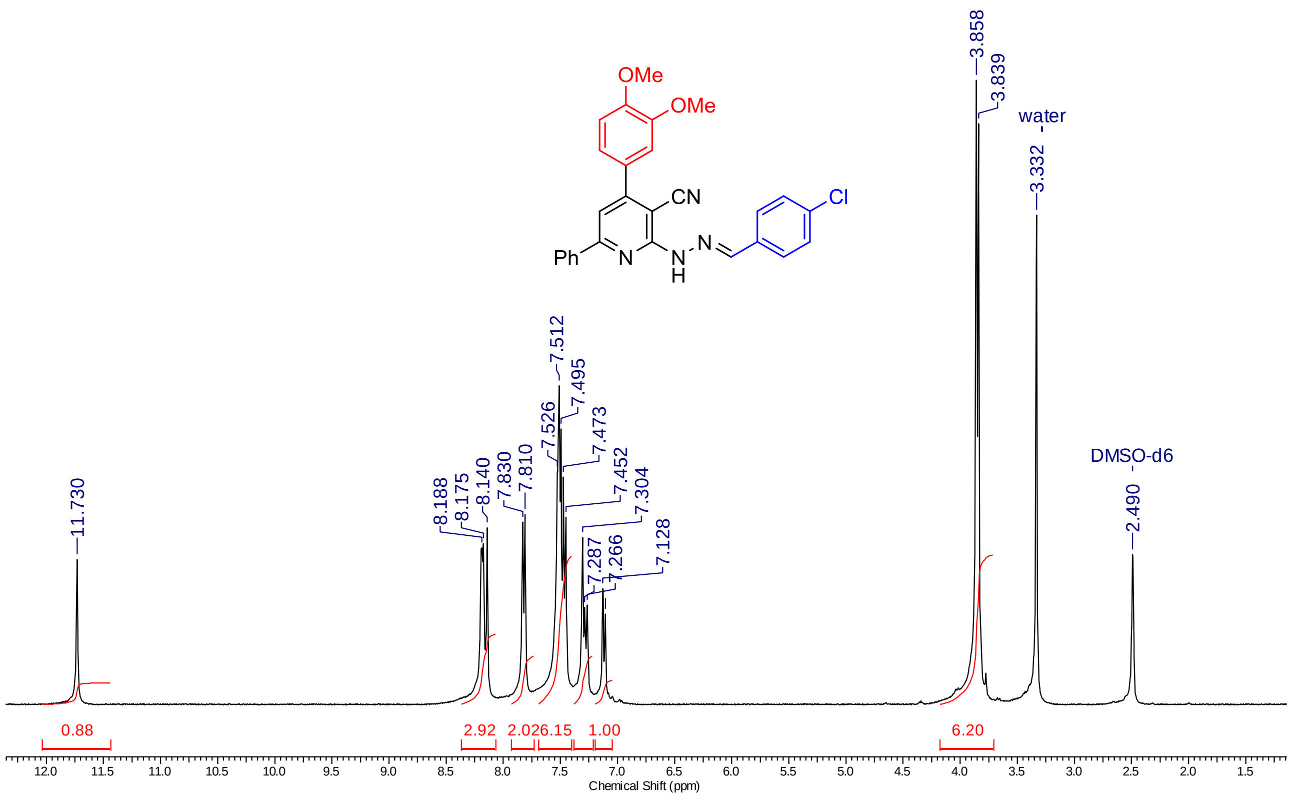

Figure S63. HRMS spectrum of hydrazone 21{*7*}

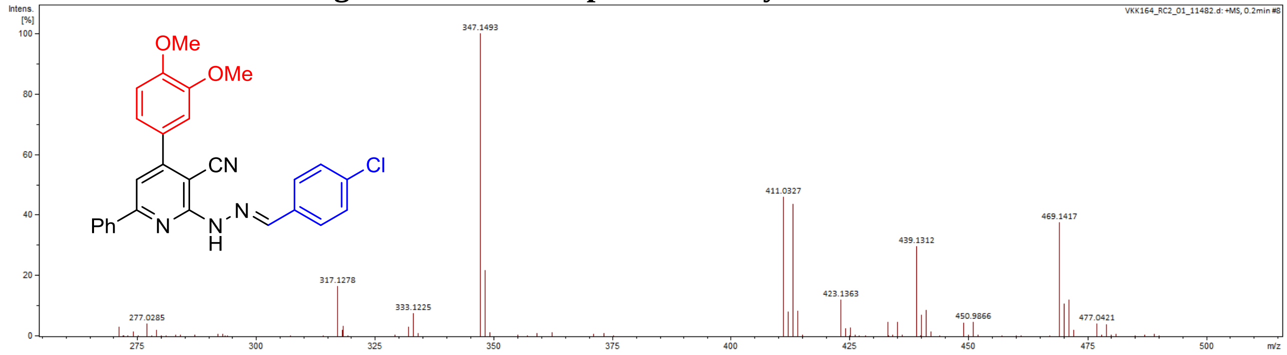

Figure S64. FTIR spectrum (nujol) of hydrazone 21{*8*}

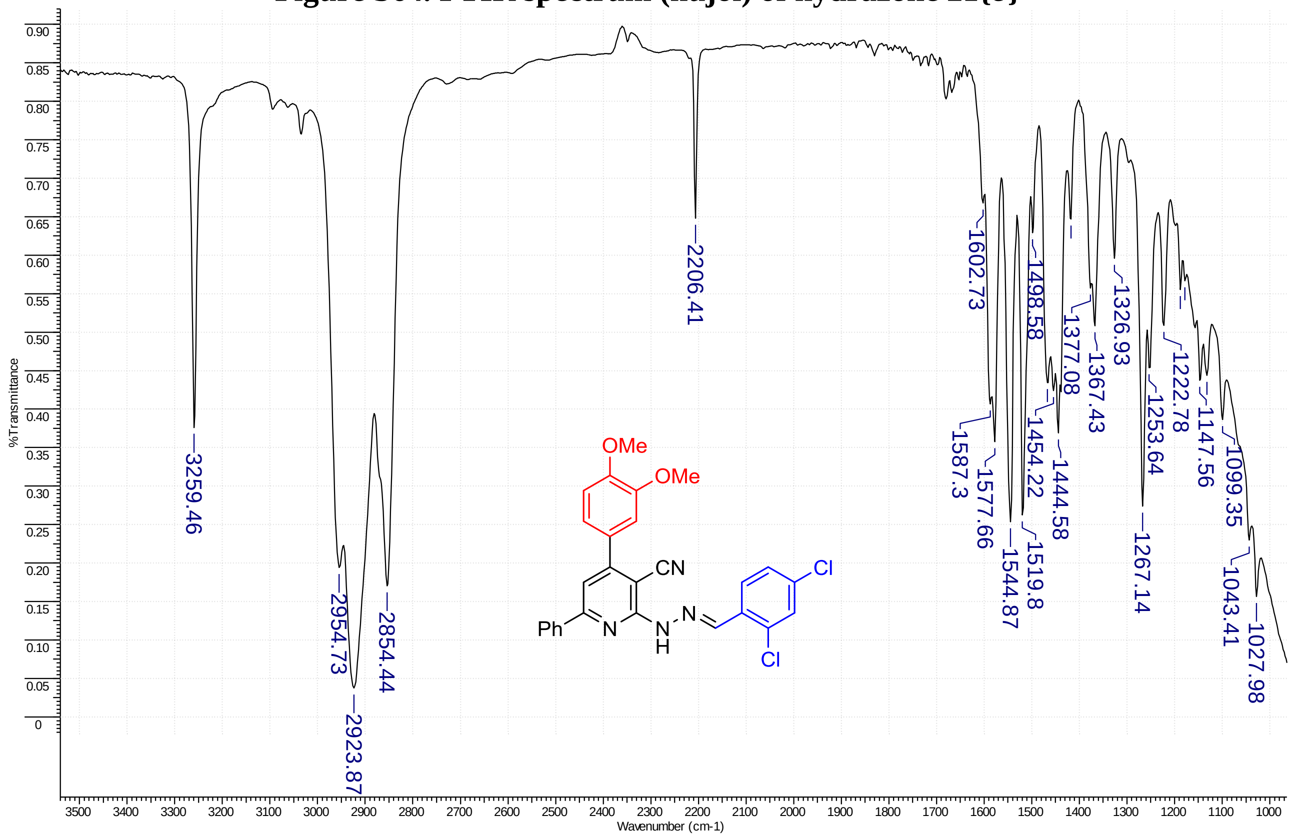

Figure S65. HRMS spectrum of hydrazone 21{*8*}

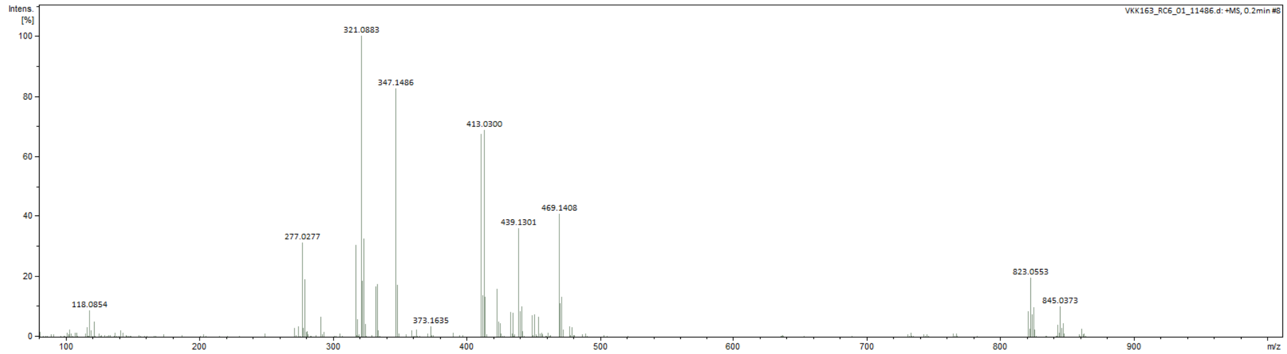

Figure S66. [1]**H NMR spectrum of hydrazone 21{*8*}, DMSO-d$_6$ (400 MHz)**

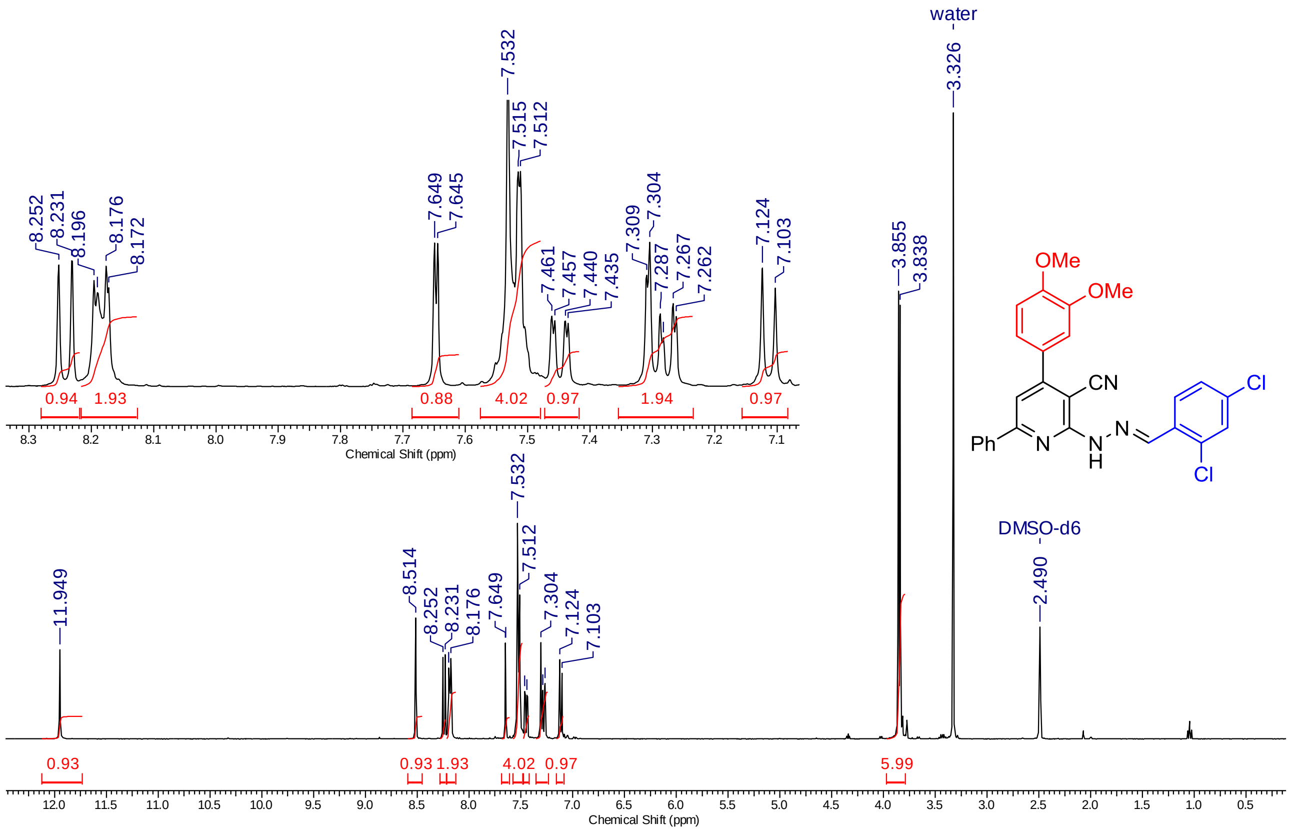

Figure S67. [13]**C NMR spectrum of hydrazone 21{*8*}, DMSO-d$_6$ (101 MHz)**

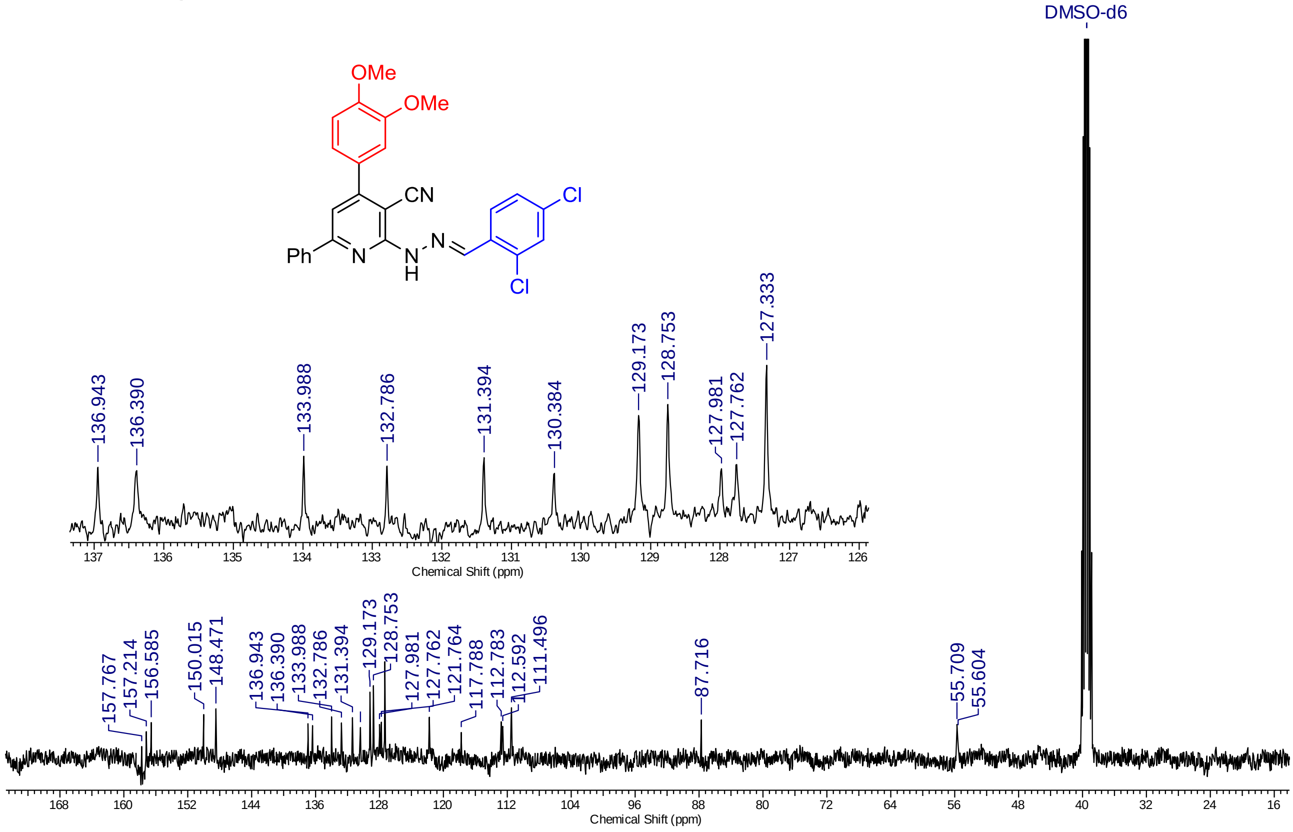

Figure S68. FTIR spectrum (nujol) of hydrazone 21{*9*}

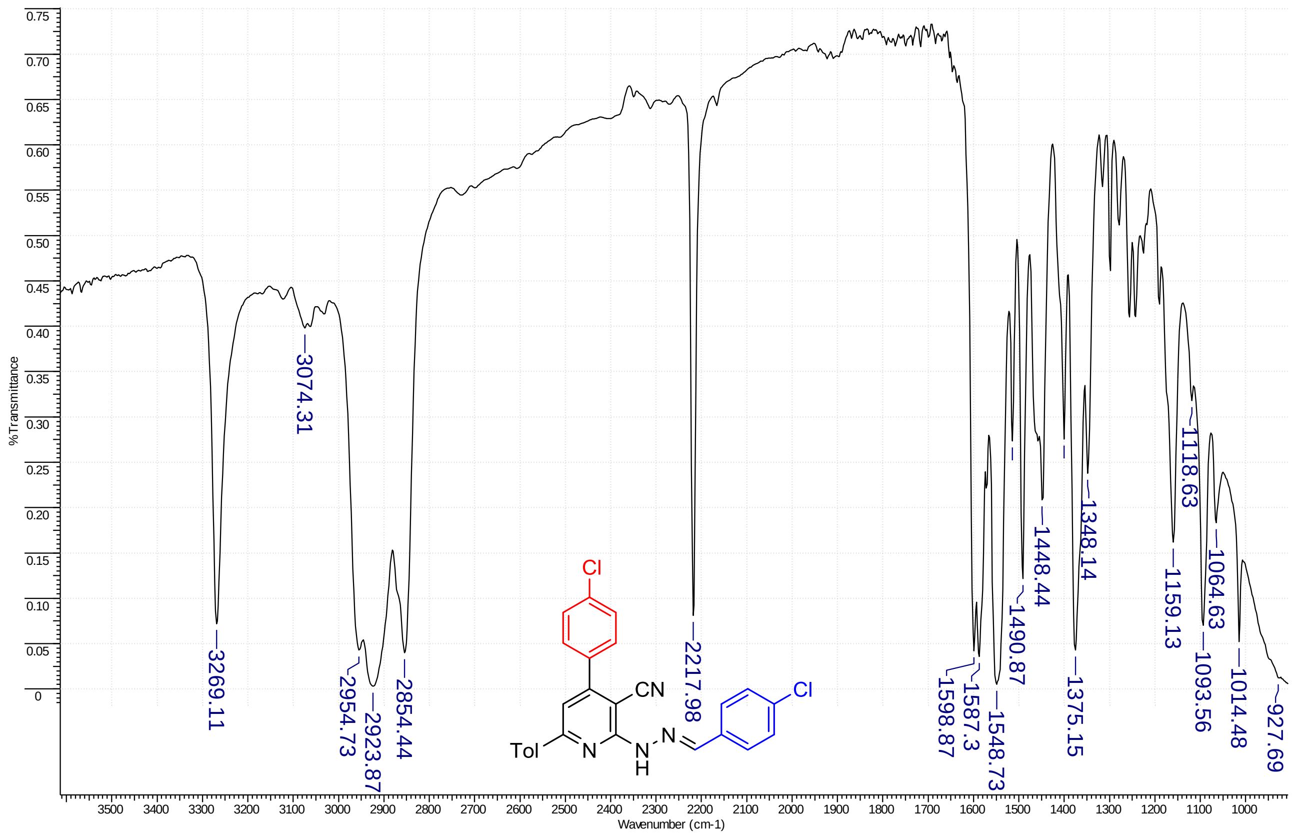

Figure S69. ^{1}H NMR spectrum of hydrazone 21{*9*}, DMSO-d$_6$ (400 MHz)

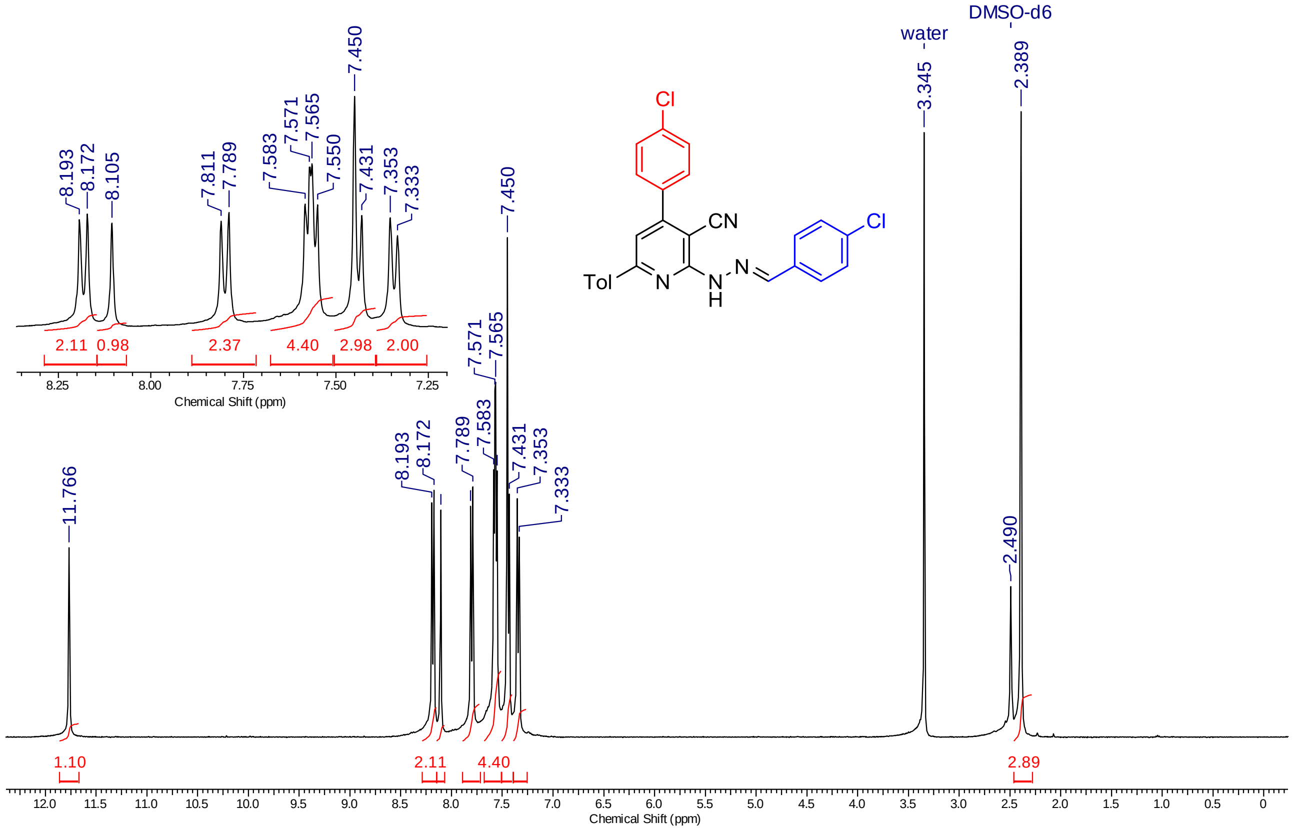

Figure S70. ^{13}C NMR spectrum of hydrazone 21{*9*}, DMSO-d_6 (101 MHz)

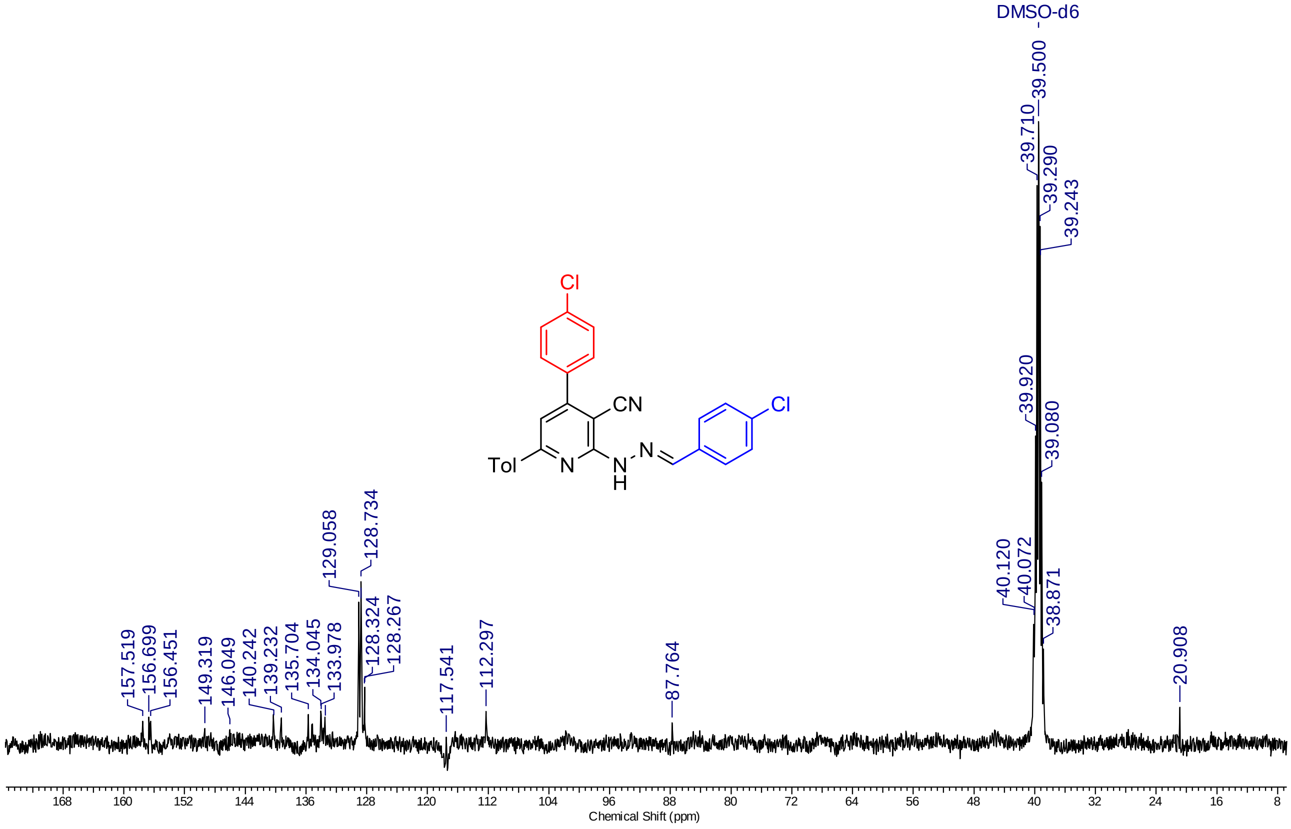

Figure S71. HRMS spectrum of hydrazone 21{*9*}

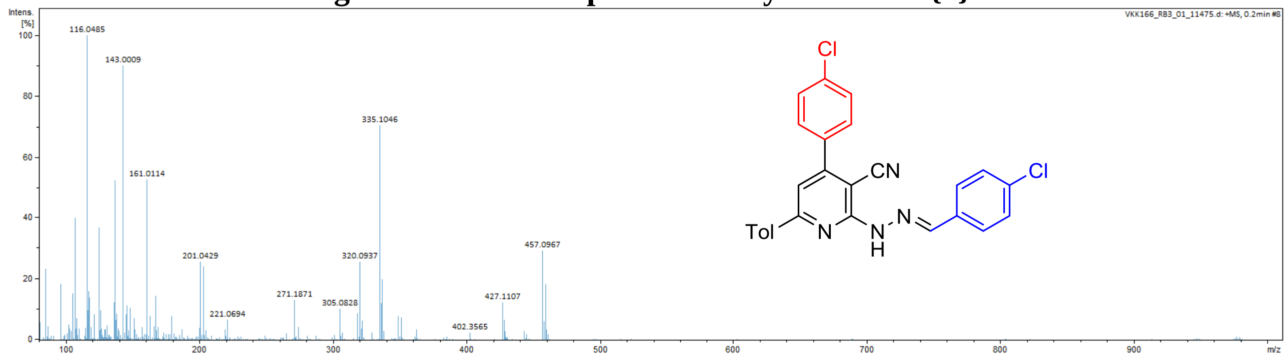

Figure S72. FTIR spectrum (nujol) of hydrazone 21{*11*}, solvate with EtOH 1 : 1

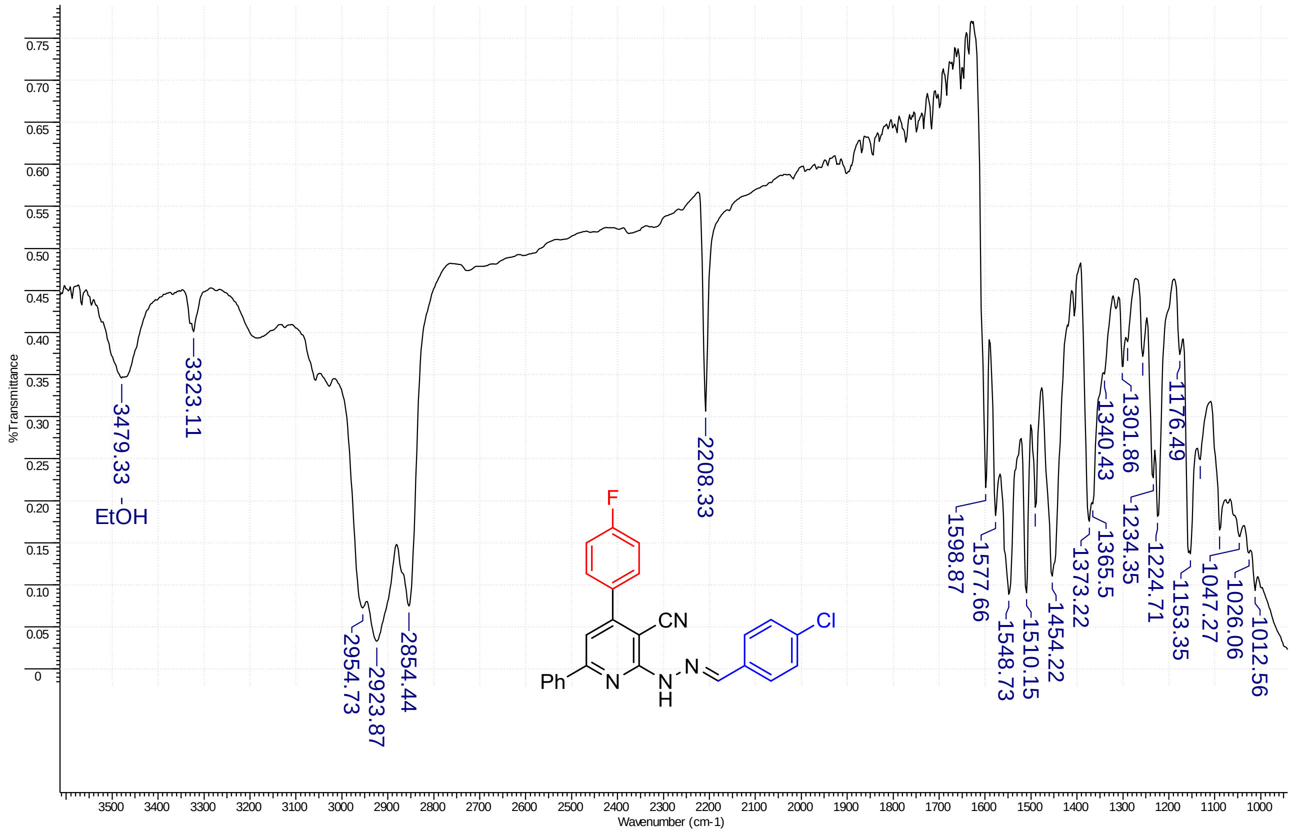

Figure S73. ^{1}H NMR spectrum of hydrazone 21{*11*}, solvate with EtOH 1 : 1, DMSO-d_6 (400 MHz)

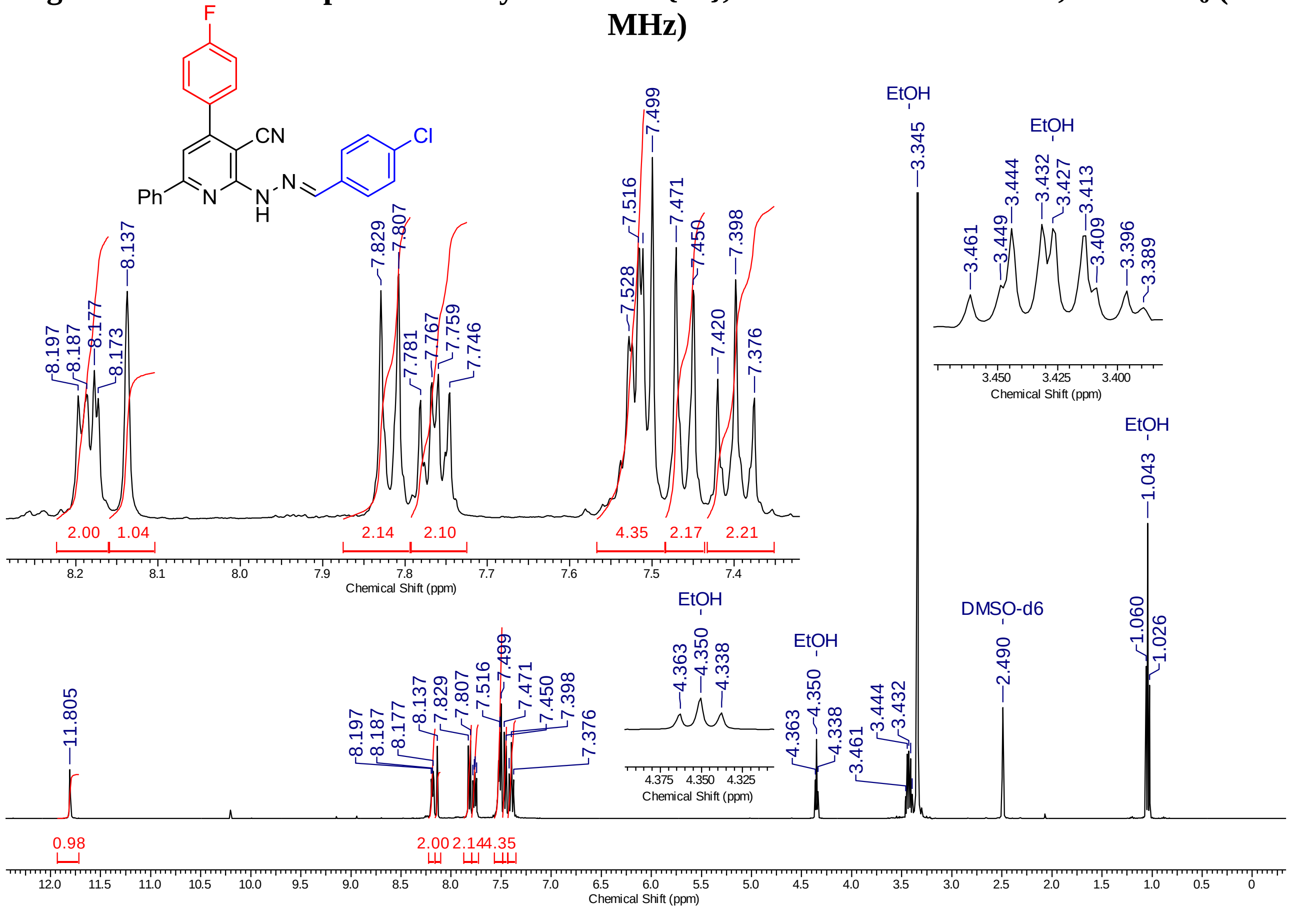

Figure S74. ^{13}**C NMR spectrum of hydrazone 21{*11*}, solvate with EtOH 1 : 1, DMSO-d$_6$ (101 MHz)**

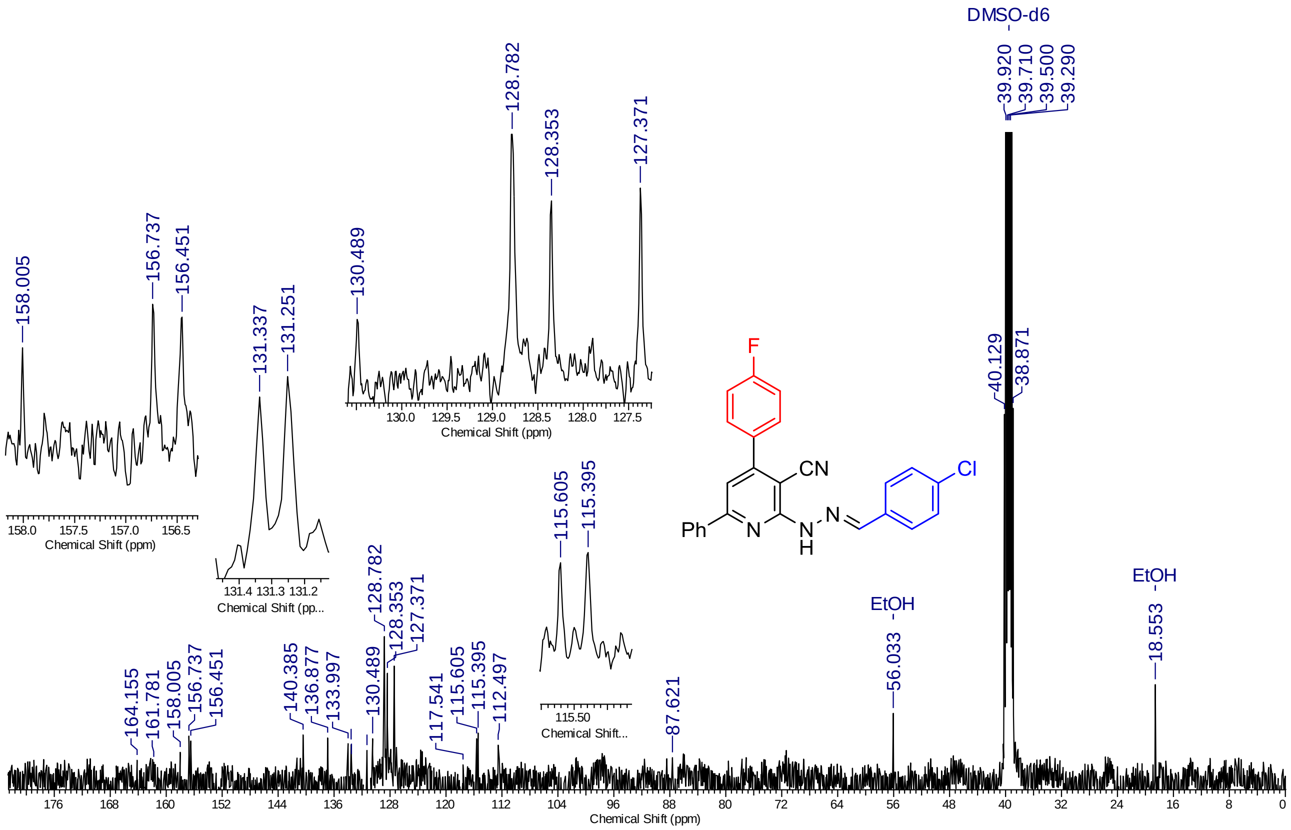

Figure S75. HRMS spectrum of hydrazone 21{*11*}

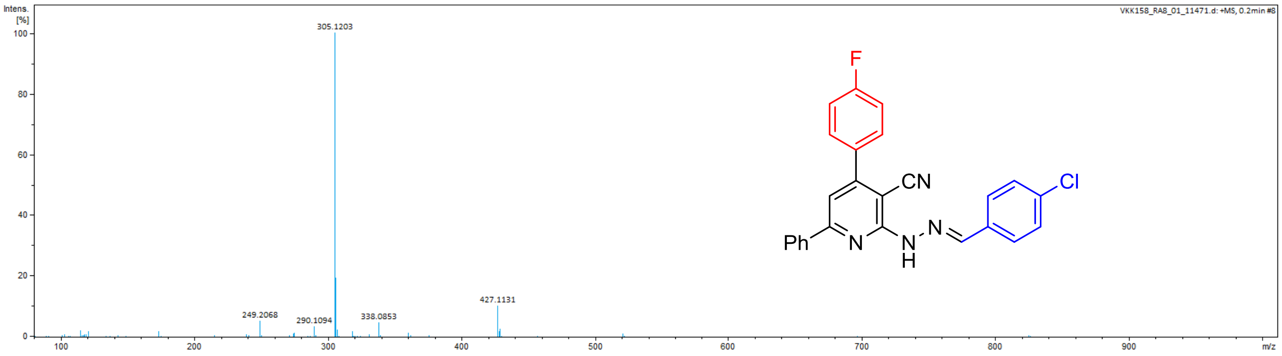

Figure S76. FTIR spectrum (nujol) of hydrazone 21{*12*}, solvate with EtOH 1 : 1

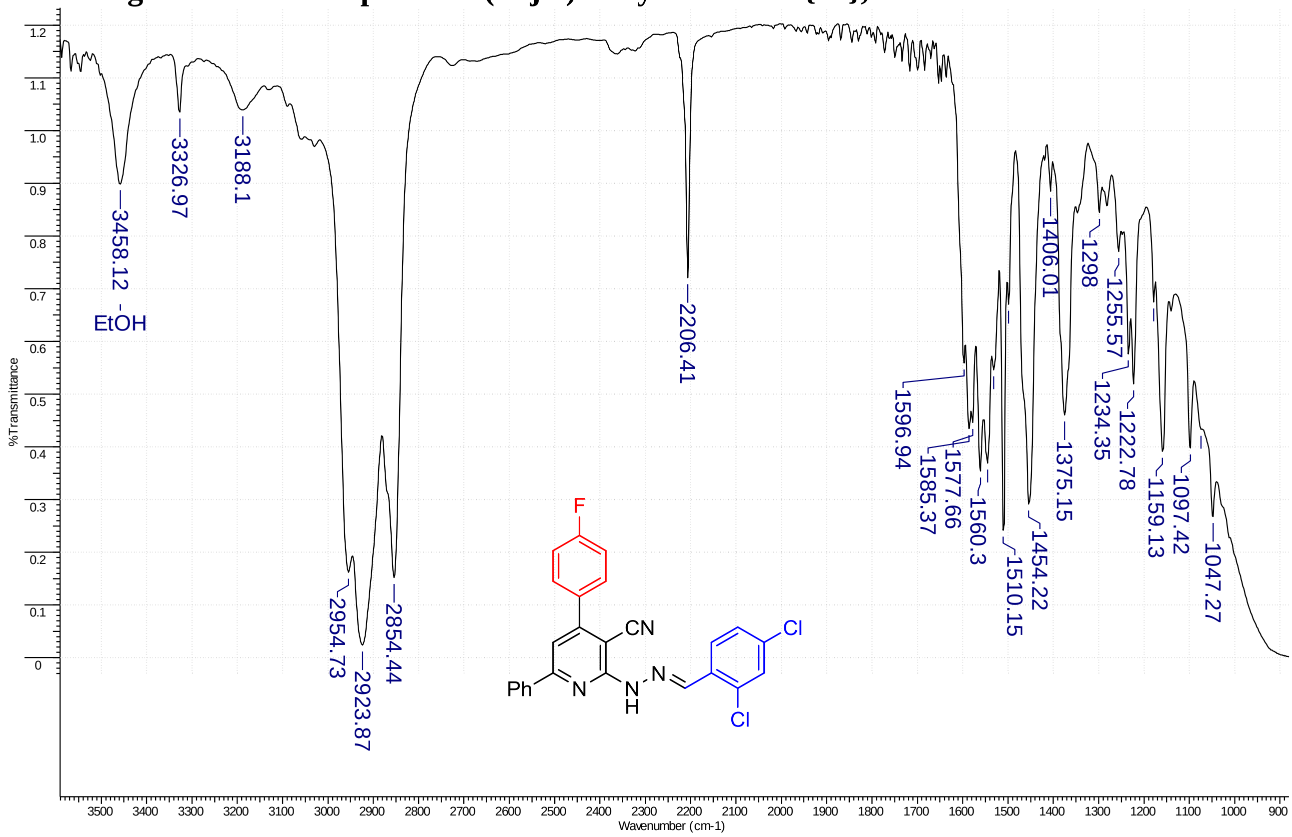

Figure S77. ^{1}H NMR spectrum of hydrazone 21{*12*}, solvate with EtOH 1 : 1, DMSO-d_6 (400 MHz)

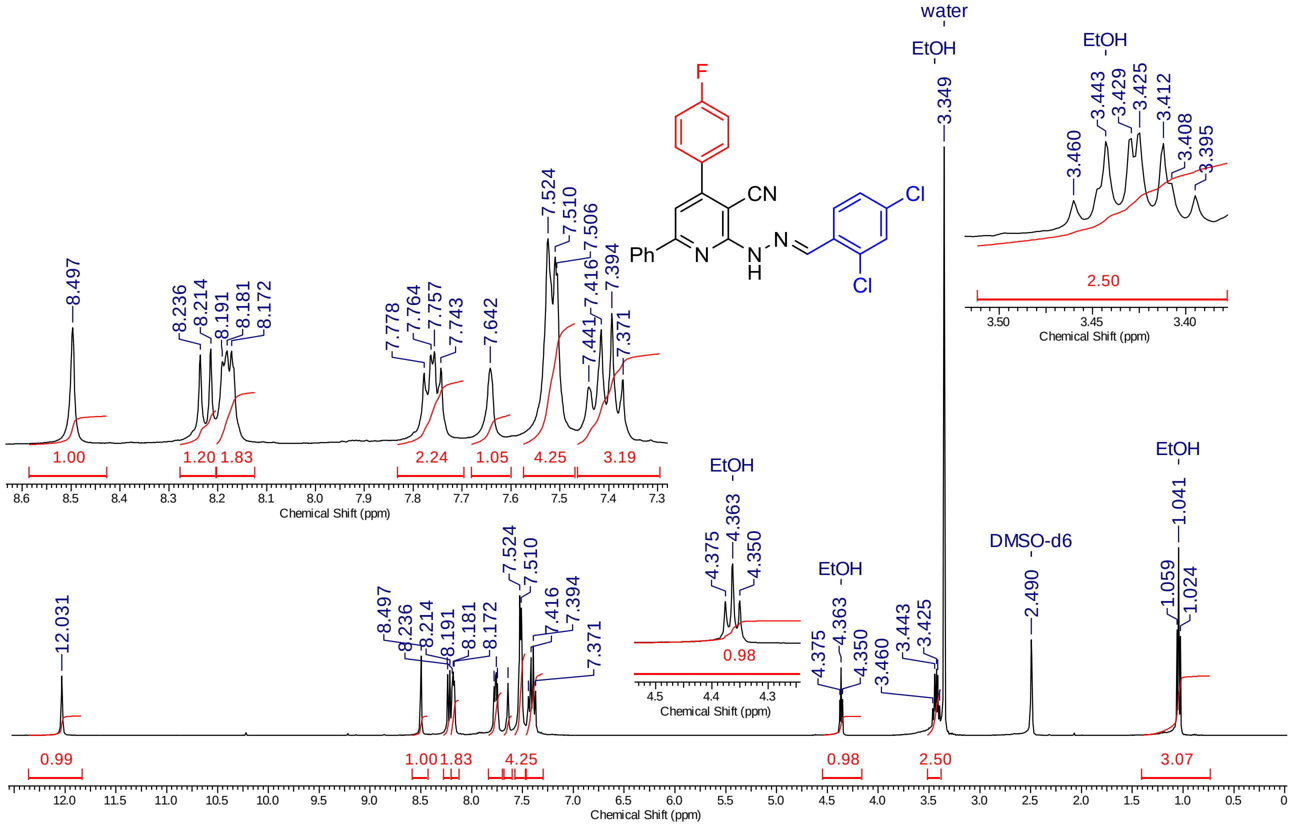

Figure S78. ^{13}**C NMR spectrum of hydrazone 21{*12*}, solvate with EtOH 1 : 1, DMSO-d$_6$ (101 MHz)**

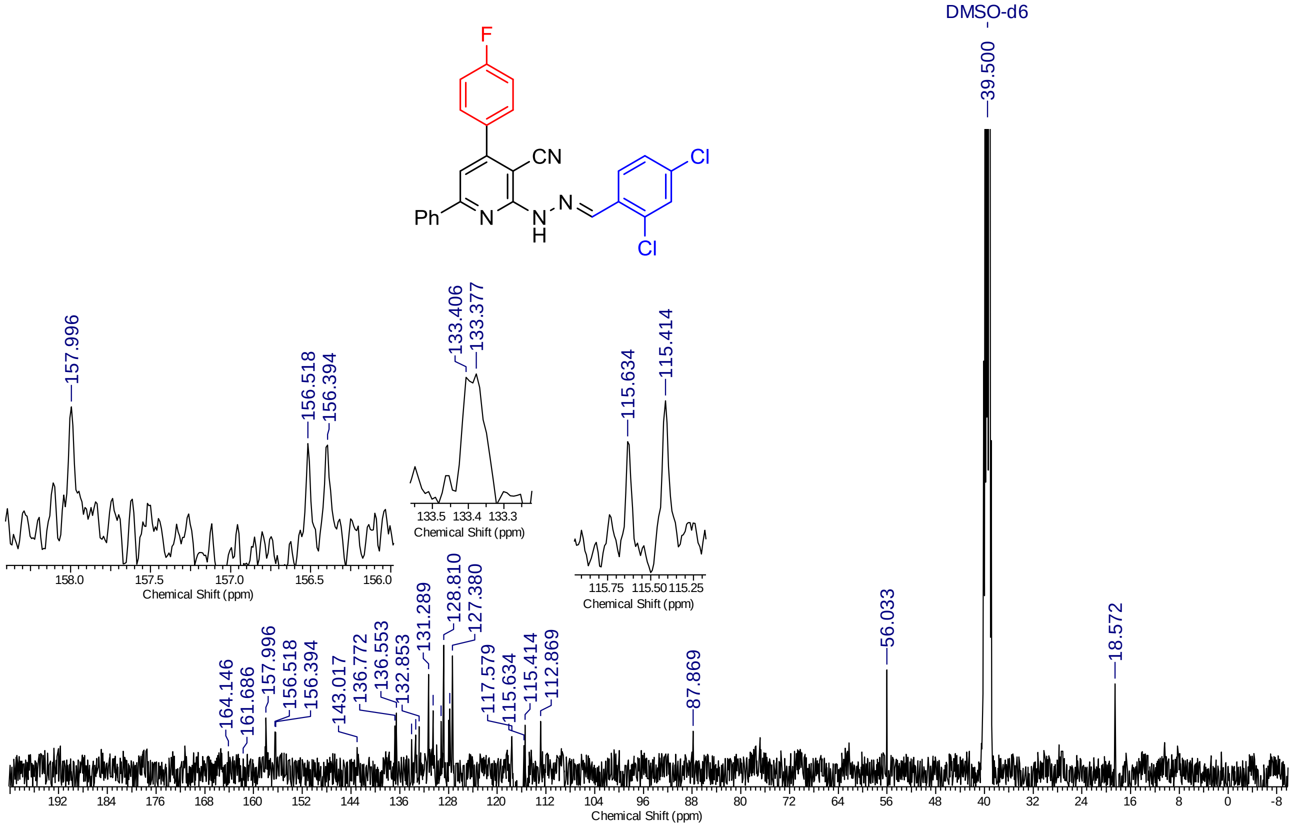

Figure S79. HRMS spectrum of hydrazone 21{*12*}

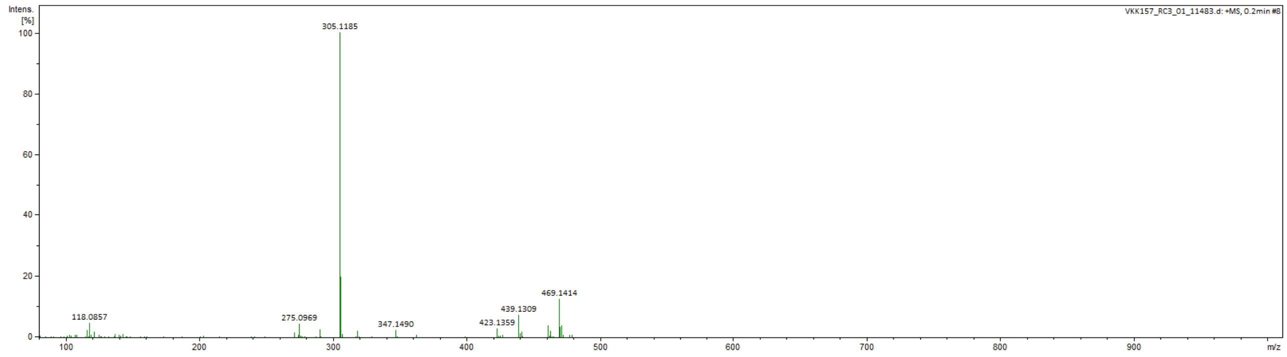

Figure S80. FTIR spectrum (nujol) of hydrazone 21{*13*}

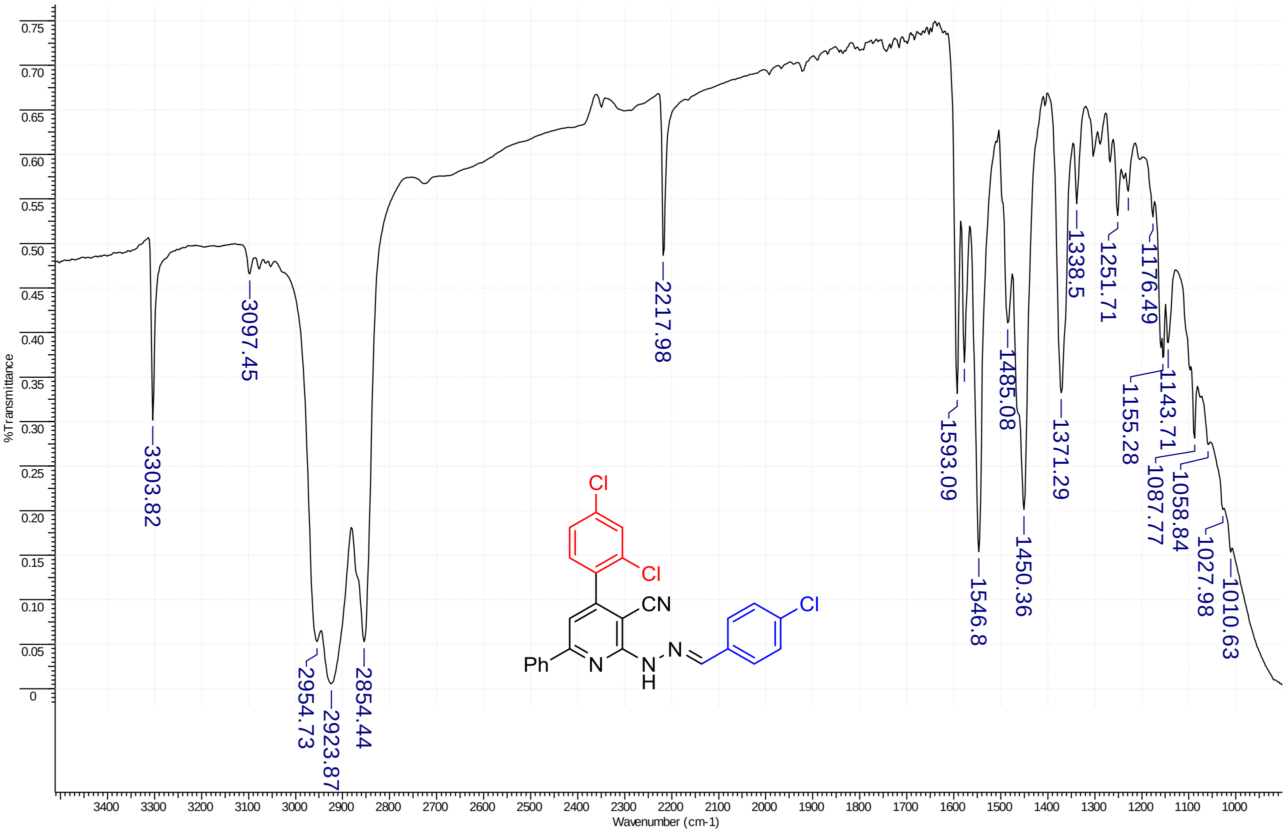

Figure S81. ^{1}H NMR spectrum of hydrazone 21{*13*}, DMSO-d$_6$ (400 MHz)

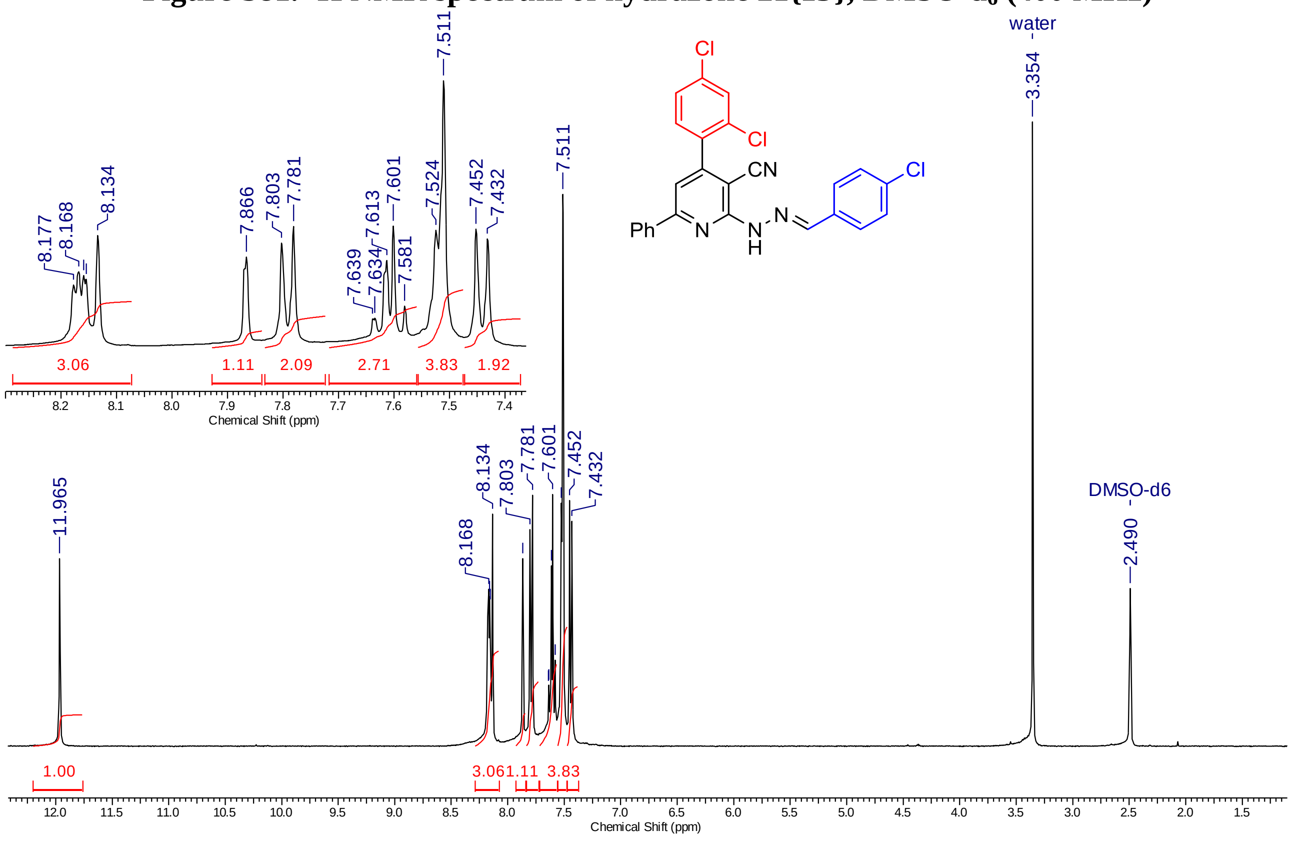

Figure S82. ^{13}C NMR spectrum of hydrazone 21{*13*}, DMSO-d_6 (101 MHz)

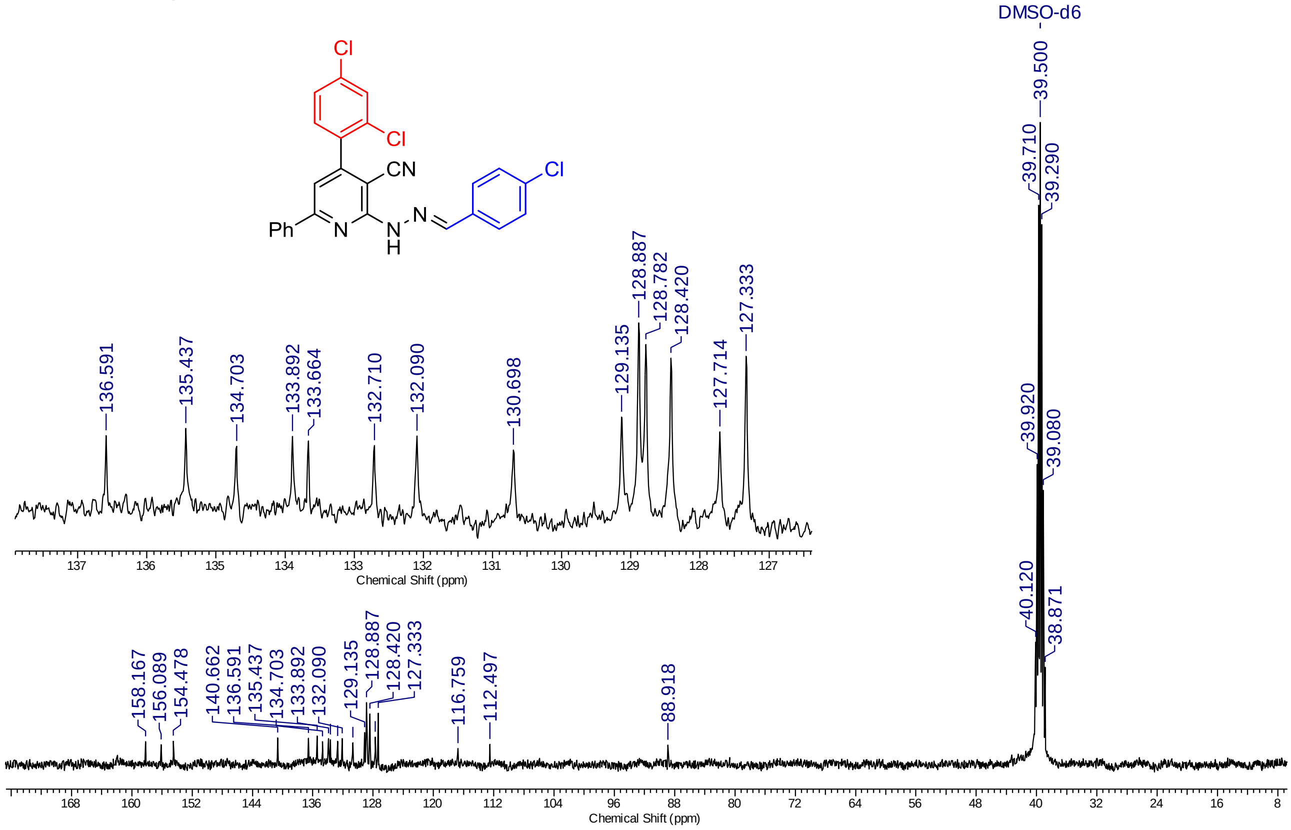

Figure S83. HRMS spectrum of hydrazone 21{*13*}

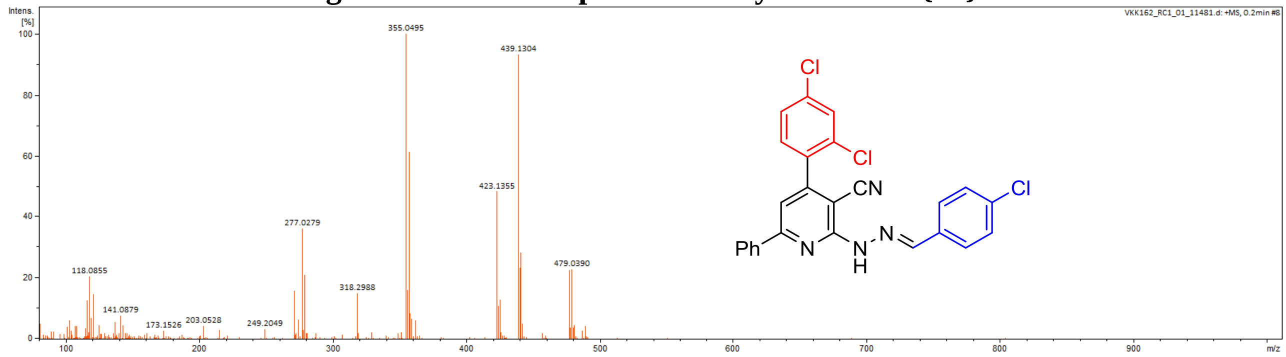

Figure S84. HRMS spectrum of hydrazone 21{*14*}

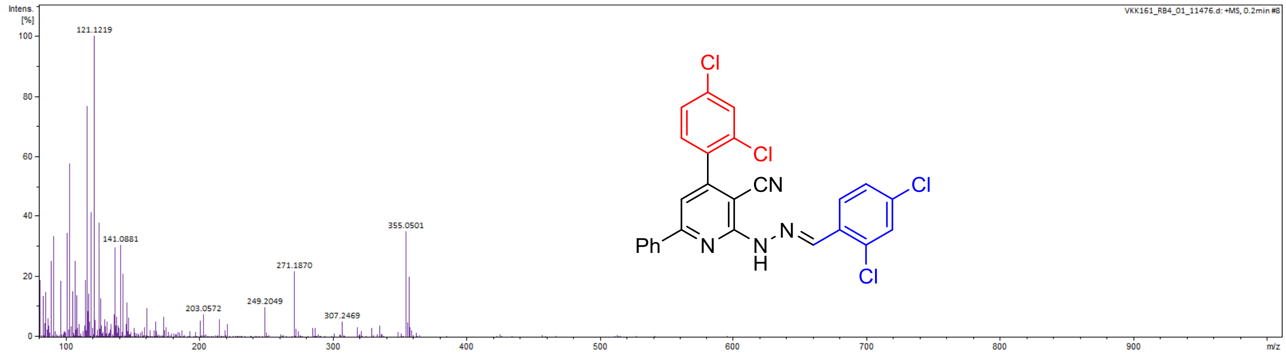

Figure S85. FTIR spectrum (nujol) of hydrazone 21{*14*}, solvate with dioxane 1 : 1,

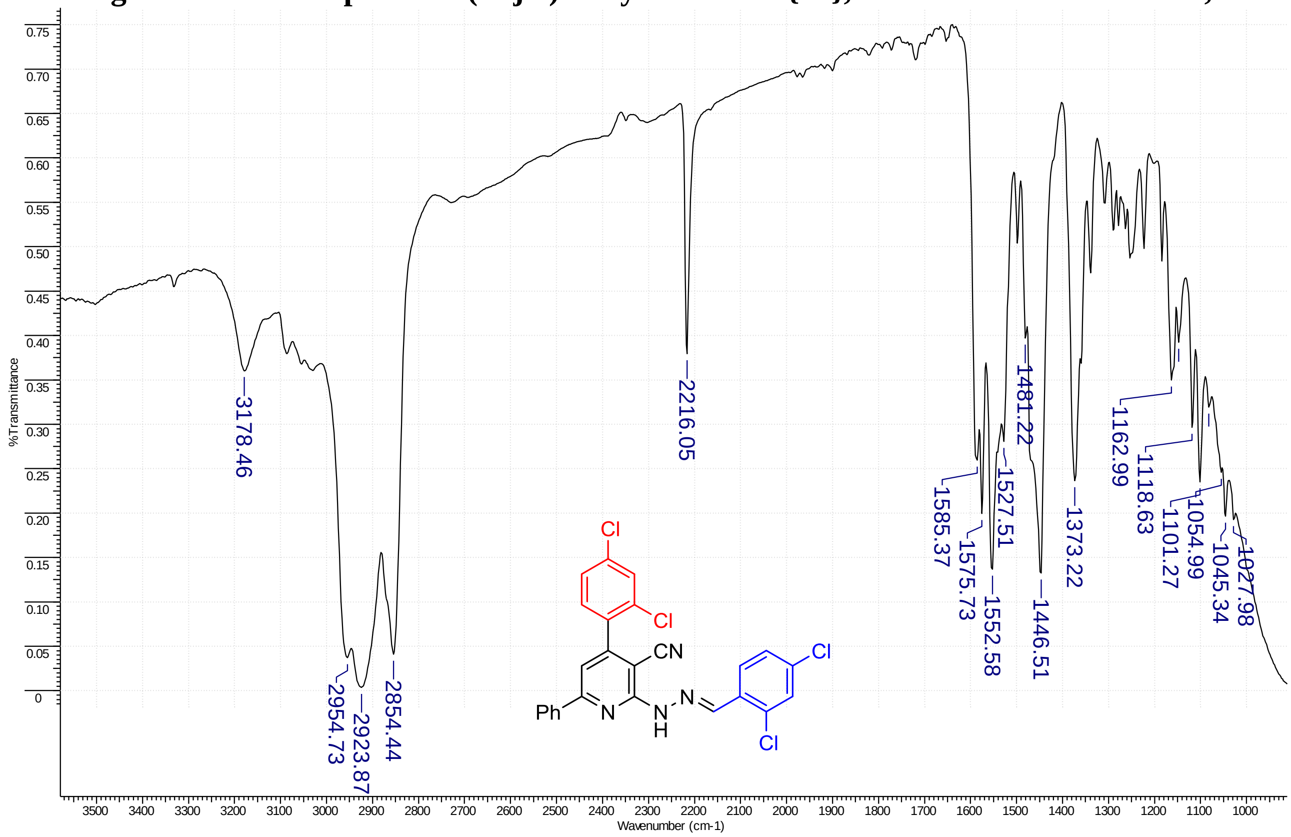

Figure S86. ^{1}H NMR spectrum of hydrazone 21{*14*}, solvate with dioxane 1 : 1, DMSO-d$_6$ (400 MHz)

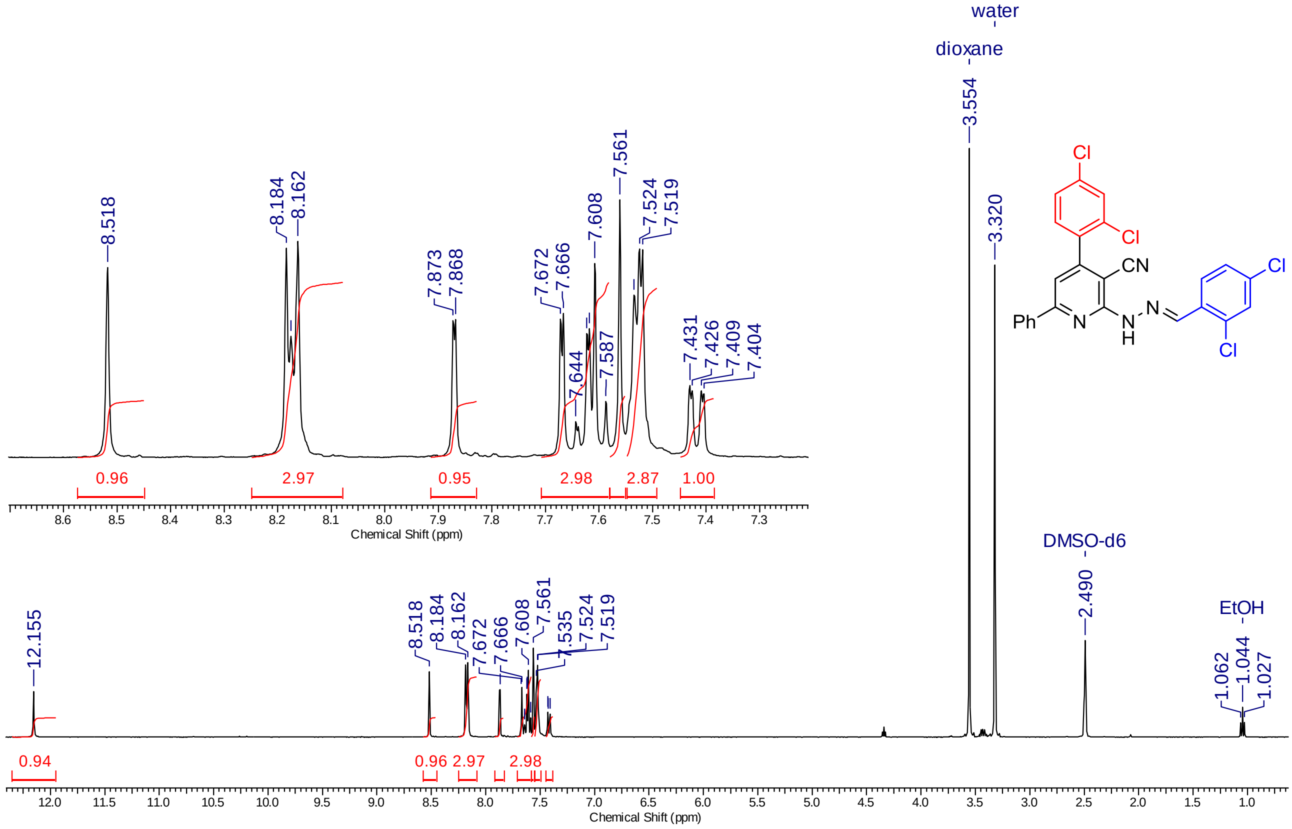

Figure S87. ^{13}C NMR spectrum of hydrazone 21{*14*}, solvate with dioxane 1 : 1, DMSO-d$_6$ (101 MHz)

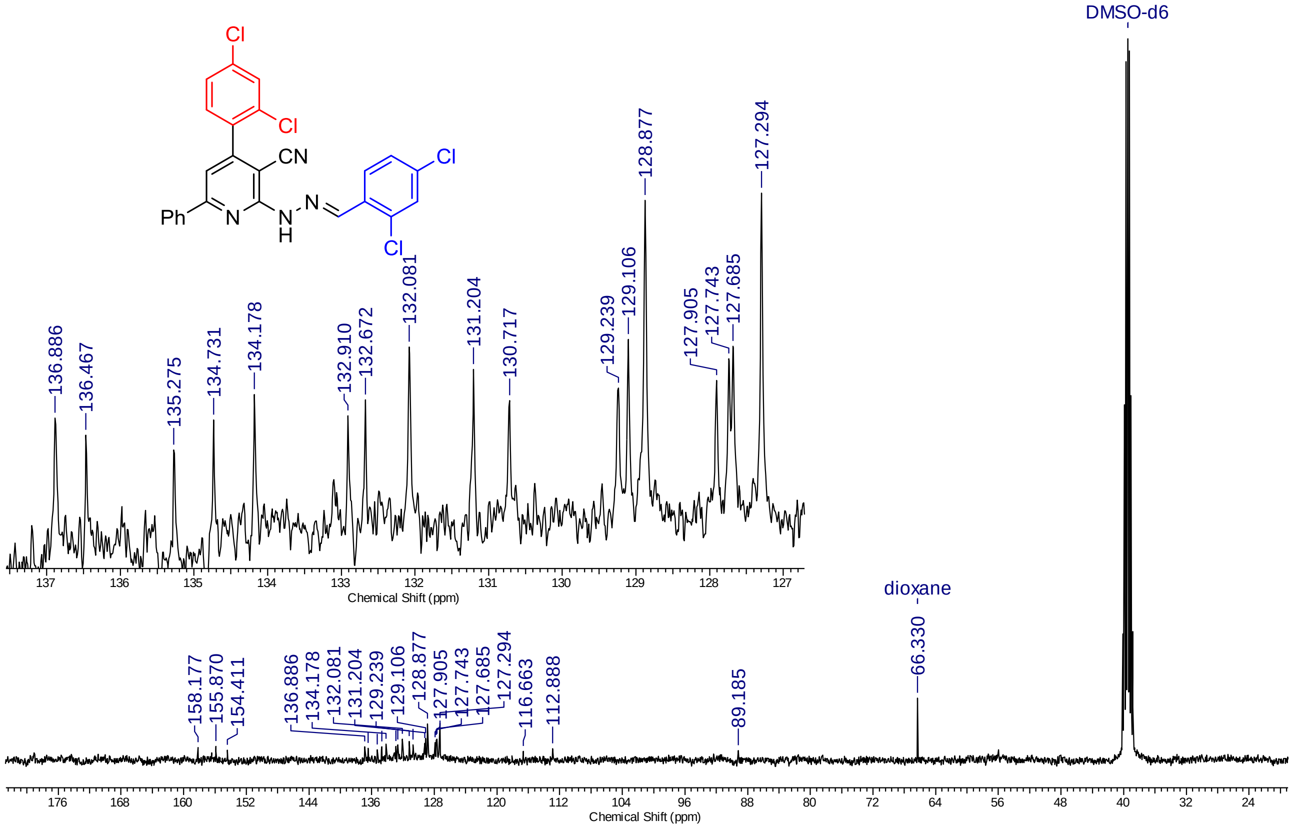

Figure S88. FTIR spectrum (nujol) of hydrazone 21{*15*}

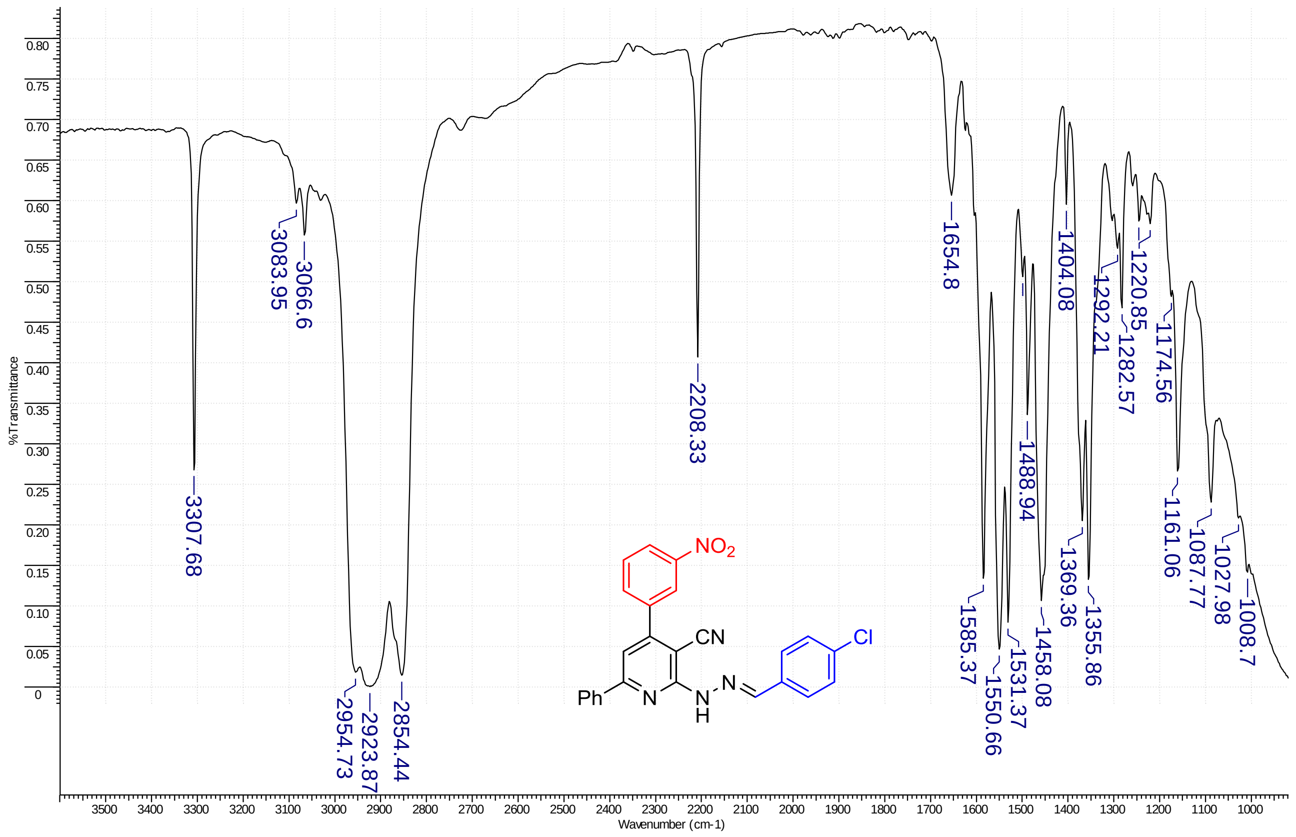

Figure S89. ^{1}H NMR spectrum of hydrazone 21{*15*}, acetone-d$_6$ (400 MHz)

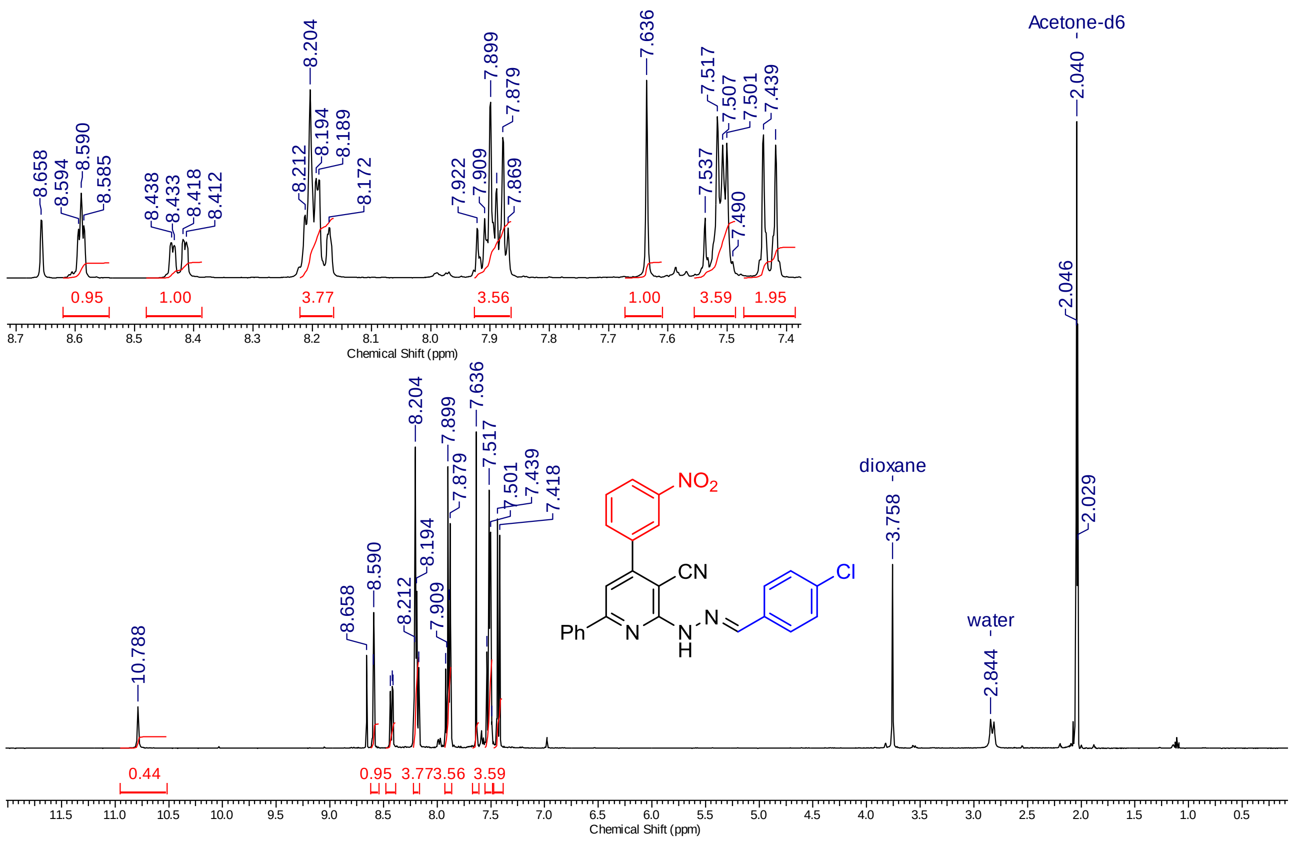

Figure S90. ^{13}C NMR spectrum of hydrazone 21{*15*}, acetone-d$_6$ (101 MHz)

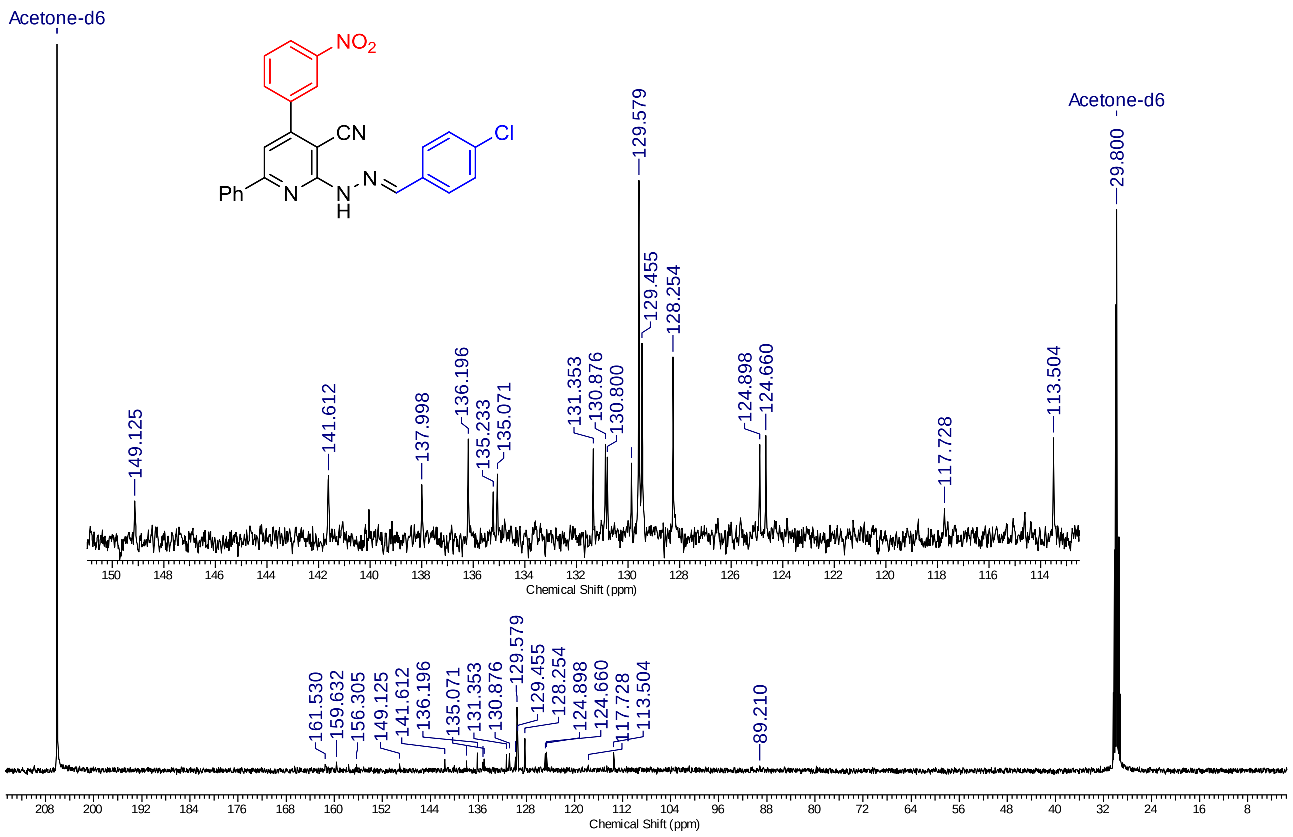

Figure S91. HRMS spectrum of hydrazone 21{*15*}

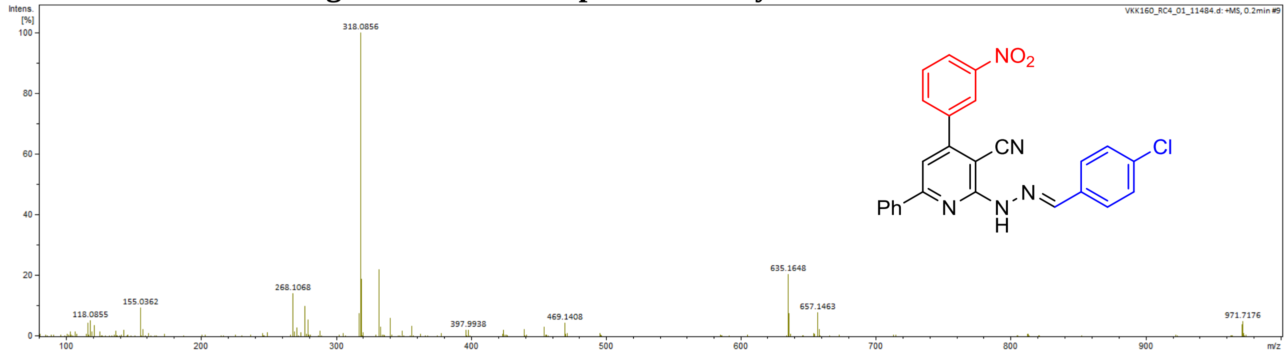

Figure S92. FTIR spectrum (nujol) of hydrazone 21{*16*}

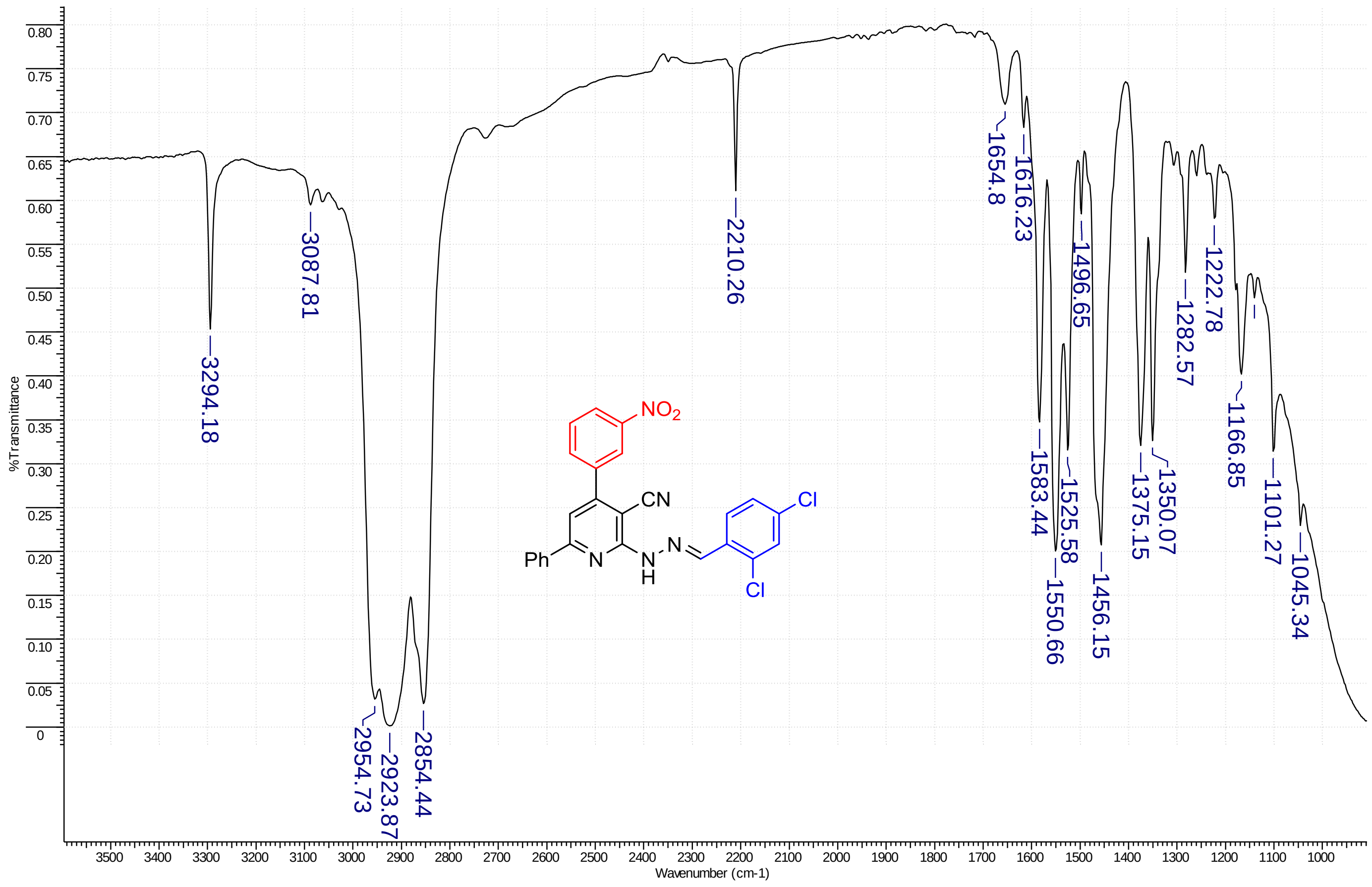

Figure S93. HRMS spectrum of hydrazone 21{*16*}

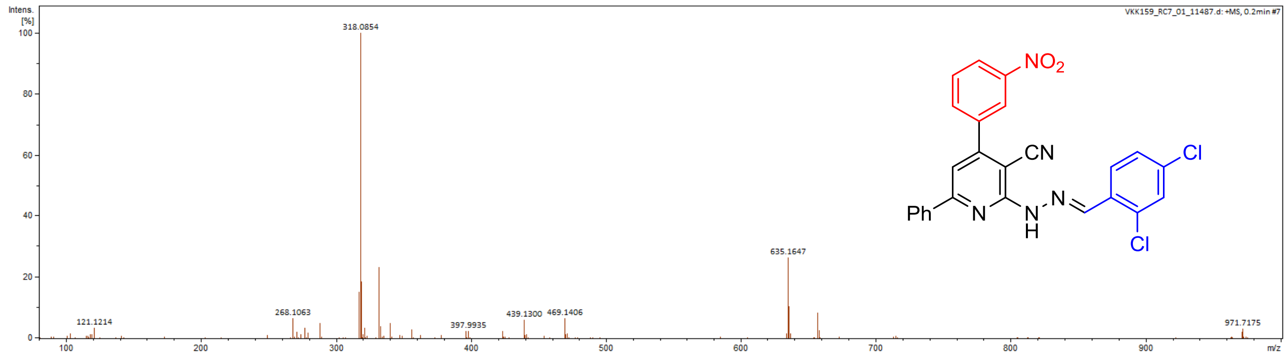

Figure S94. FTIR spectrum (nujol) of hydrazone 21{*17*}

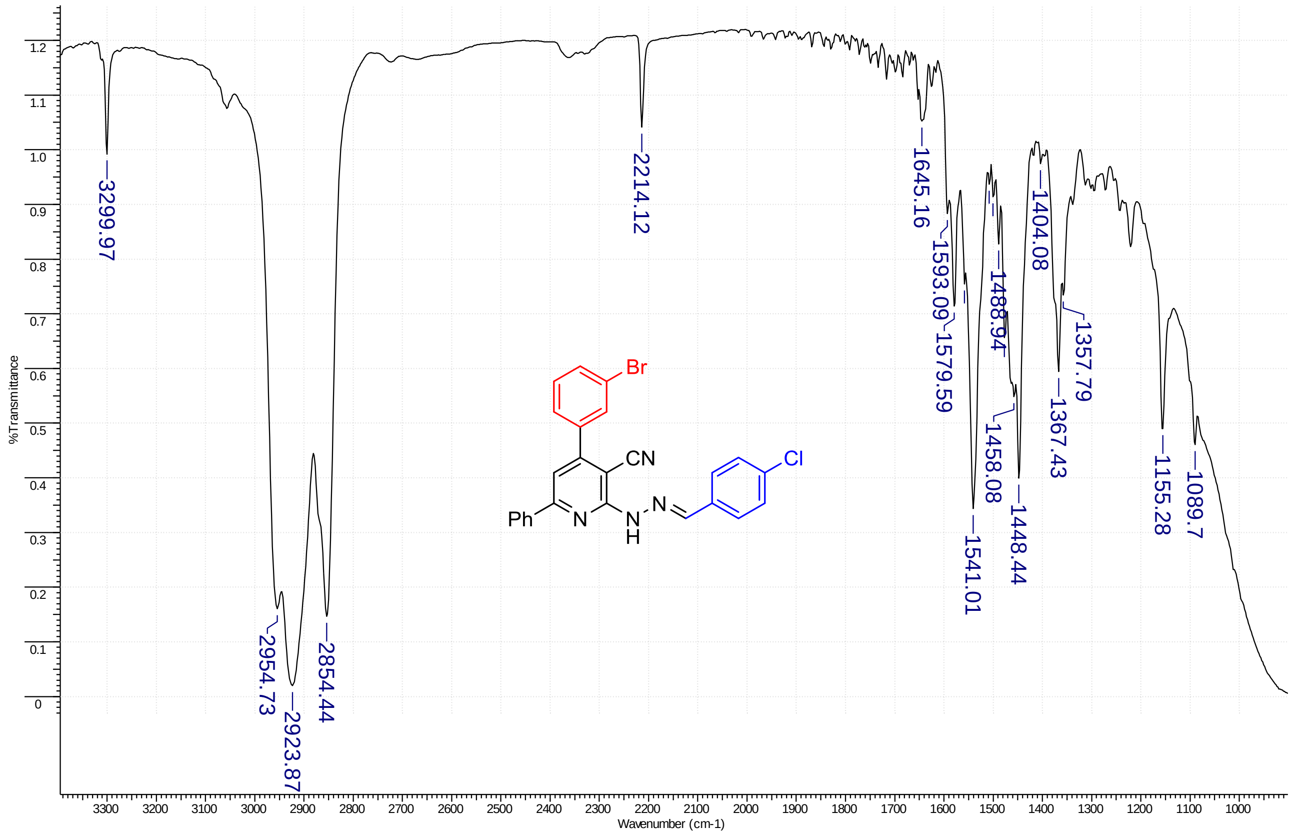

Figure S95. FTIR spectrum (nujol) of hydrazone 21{*19*}

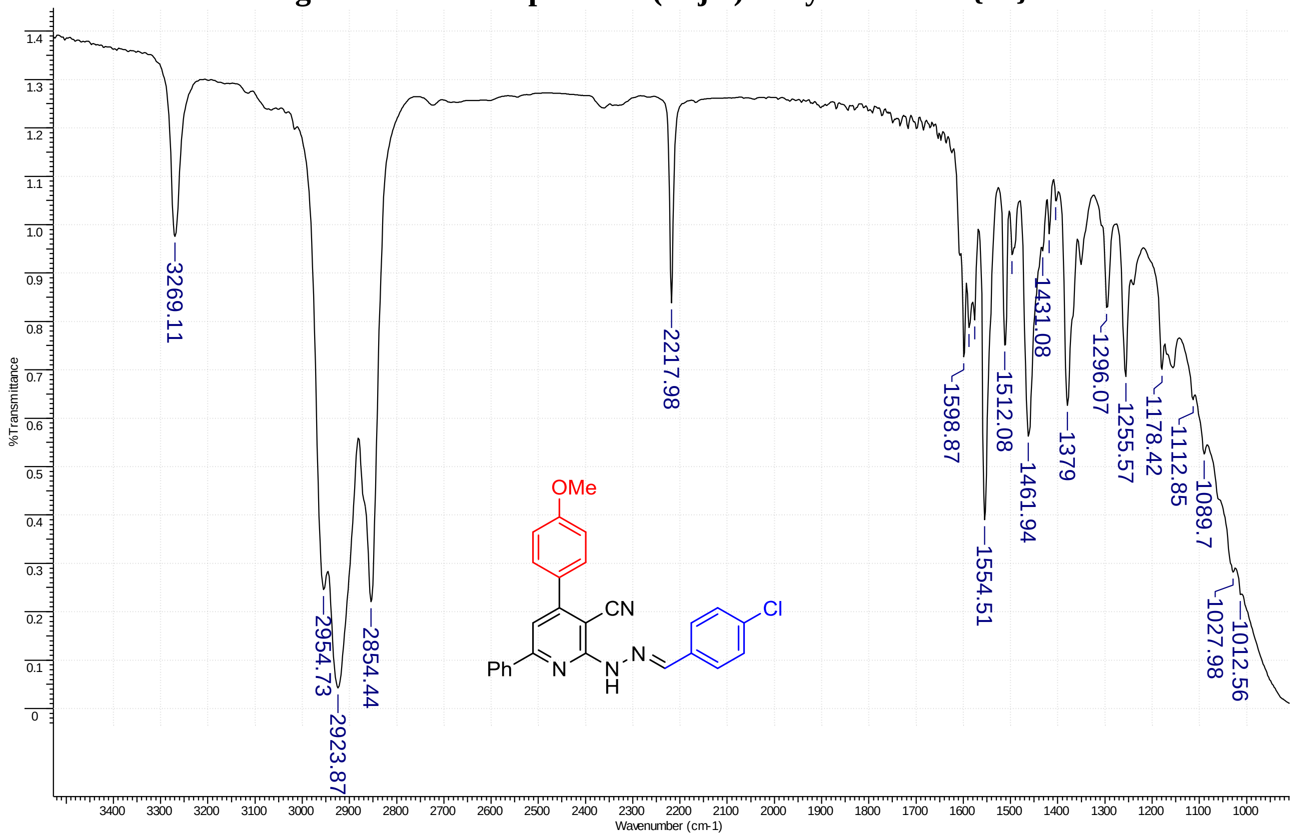

Figure S96. ^{1}H NMR spectrum of hydrazone 21{*19*}, DMSO-d_6 (400 MHz)

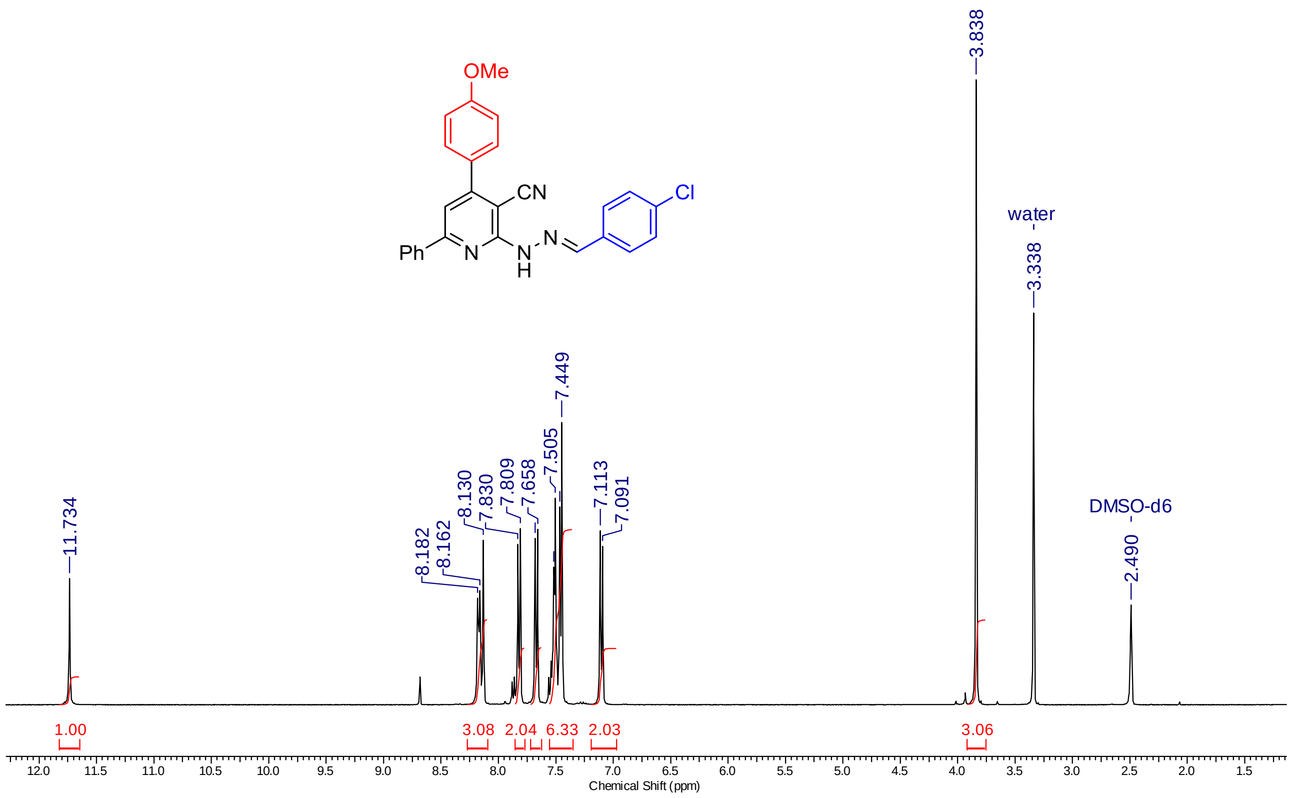

Figure S97. ^{13}C NMR spectrum of hydrazone 21{*19*}, DMSO-d_6 (101 MHz)

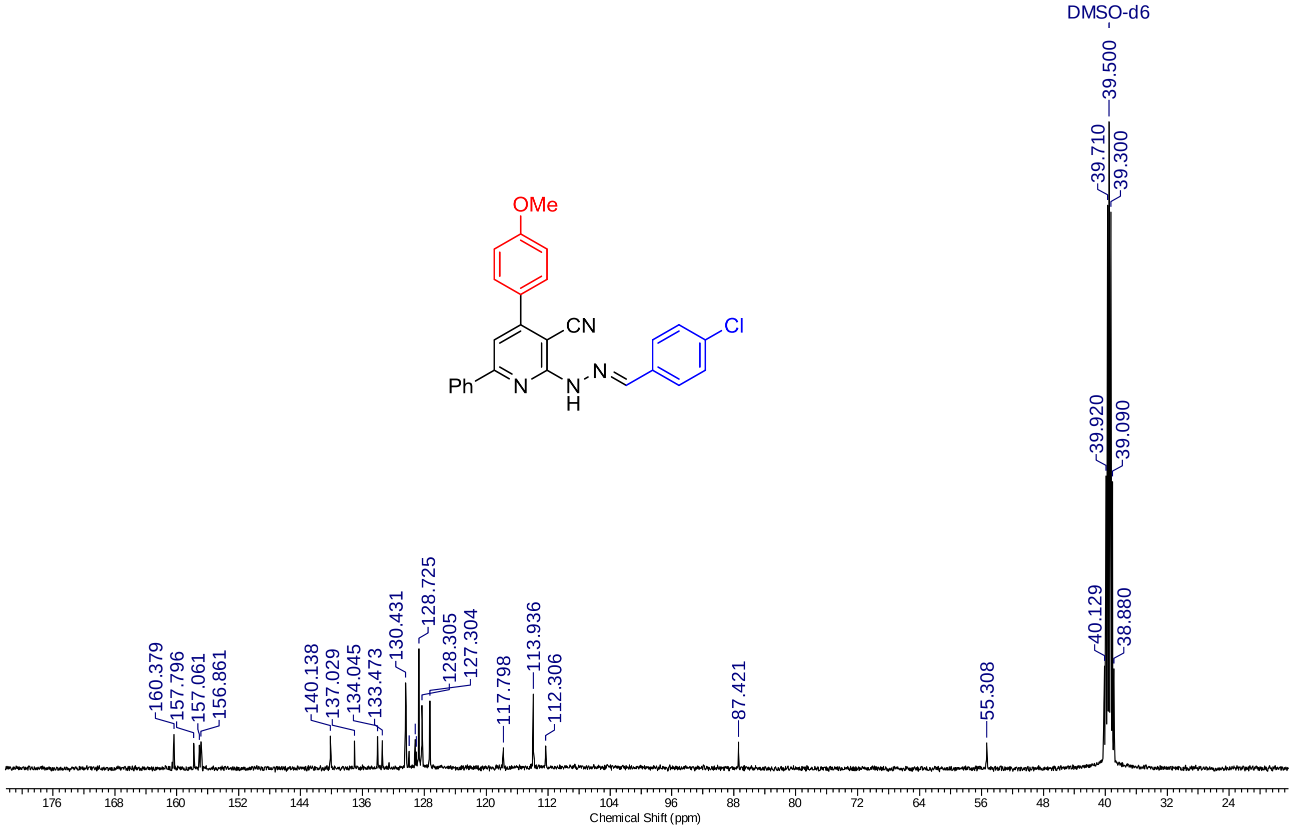

Figure S98. HRMS spectrum of hydrazone 21{*19*}

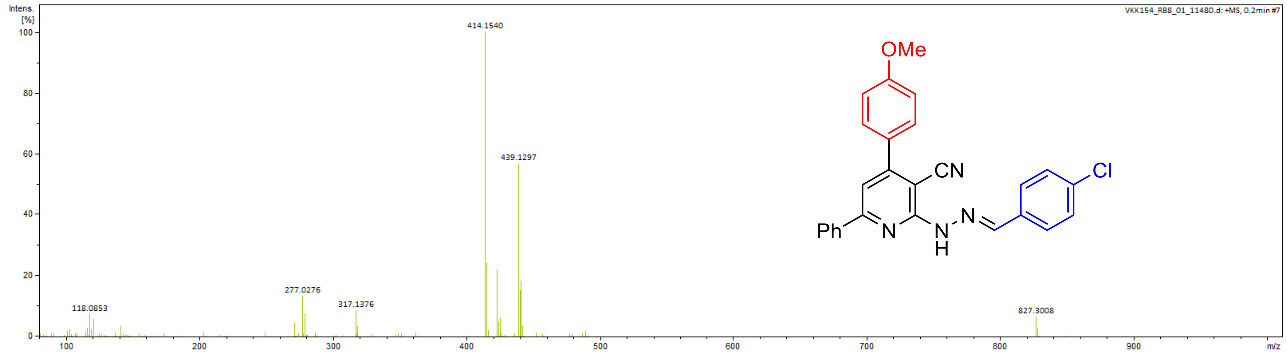

Figure S99. FTIR spectrum (nujol) of hydrazone 21{*20*}

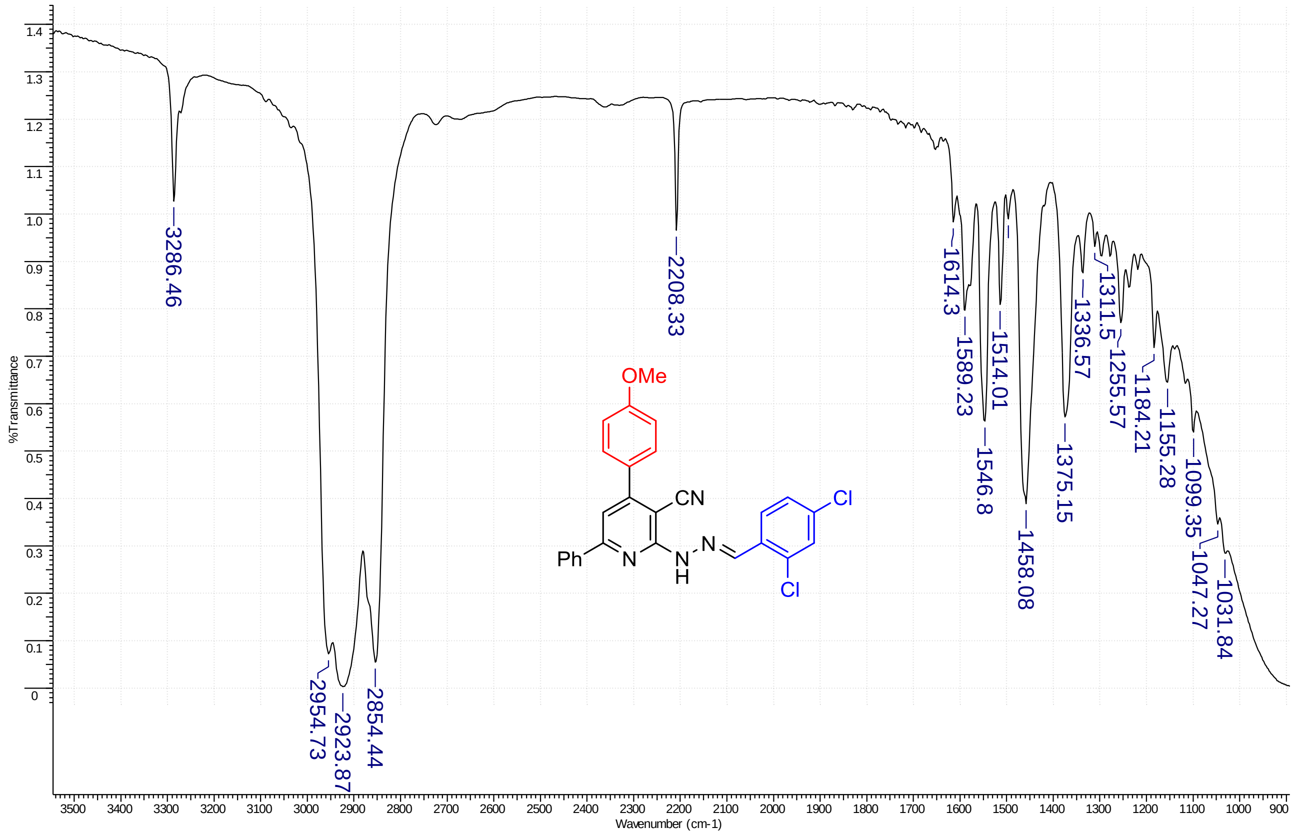

Figure S100. ^{1}H NMR spectrum of hydrazone 21{*20*}, DMSO-d$_6$ (400 MHz)

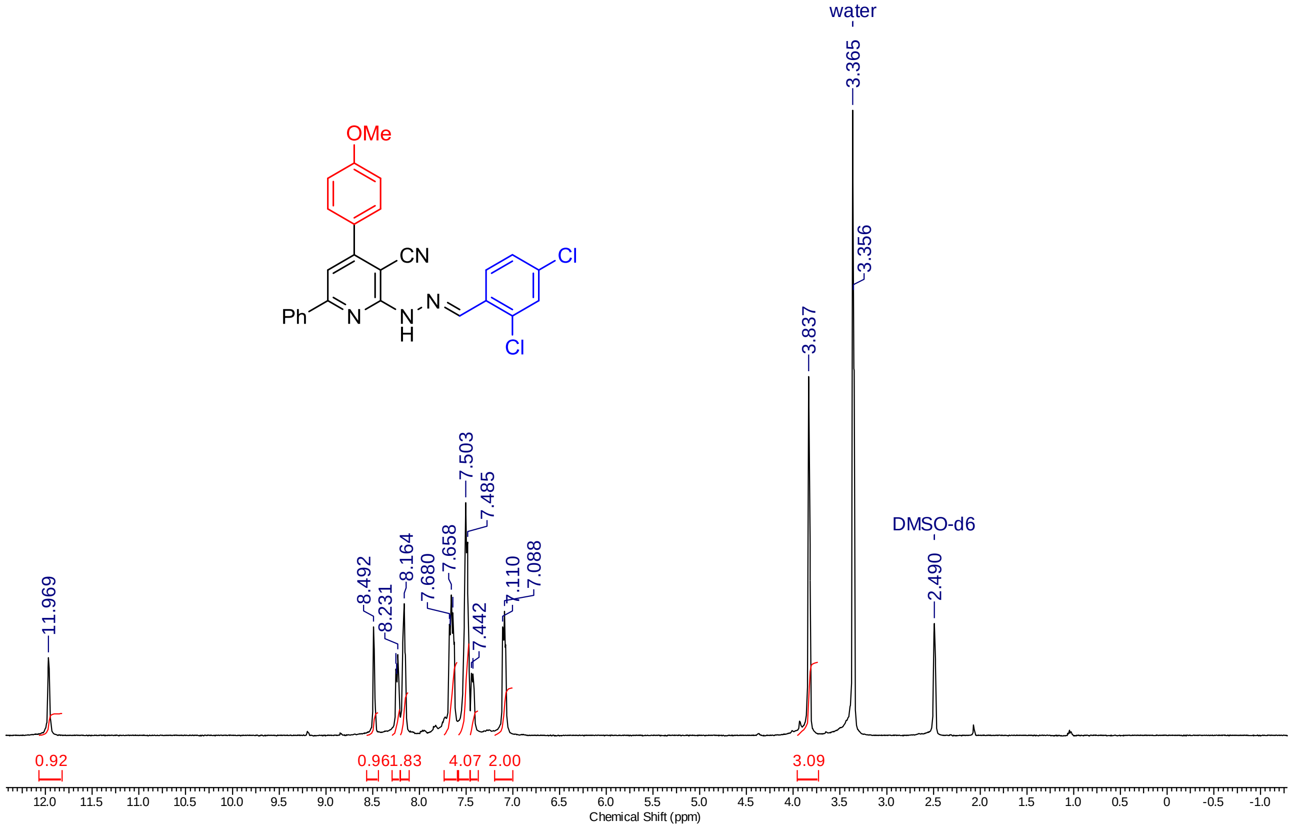

Figure S101. ^{13}**C NMR spectrum of hydrazone 21{*20*}, DMSO-d**$_6$ **(101 MHz)**

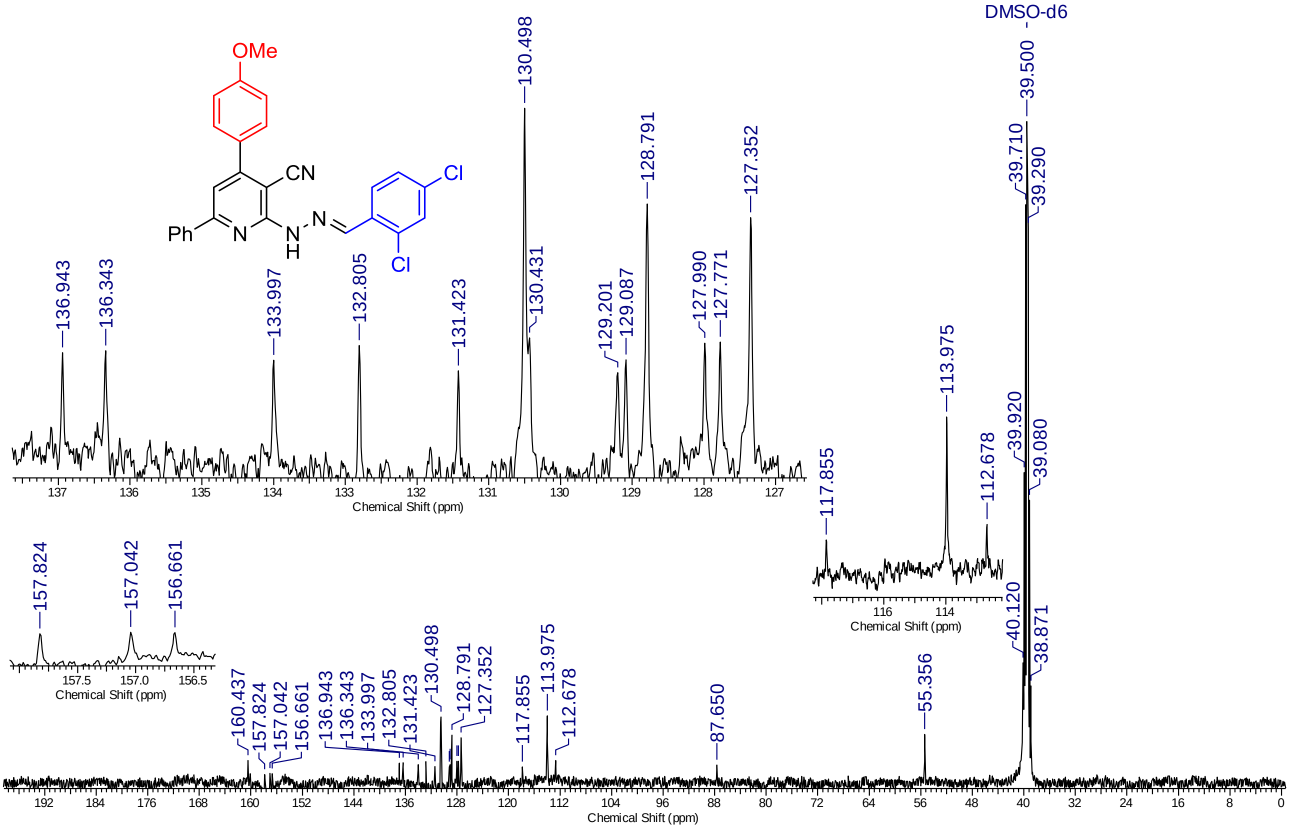

Figure S102. HRMS spectrum of hydrazone 21{*20*}

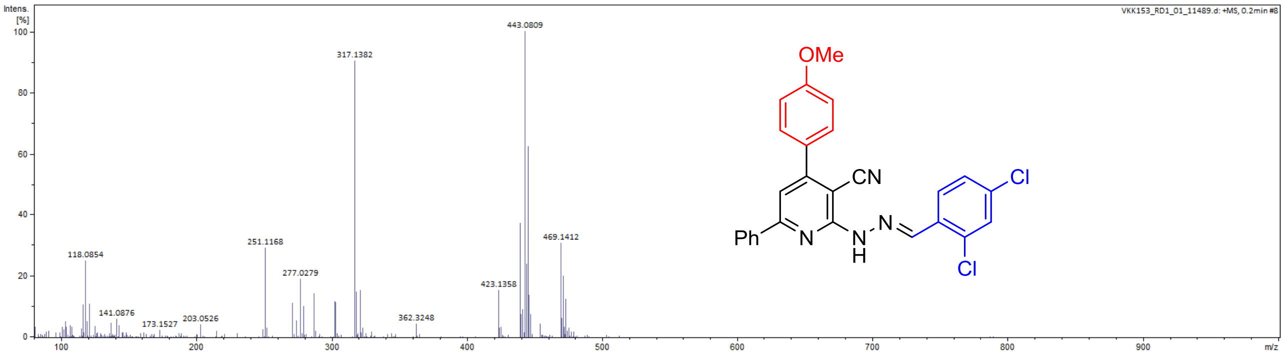

Figure S103. ORTEP drawing of X-ray structure for 2-bromo-4-(4-chlorophenyl)-6-phenylnicotinonitrile (15a) with 50% probability (CCDC deposition number 2499675)

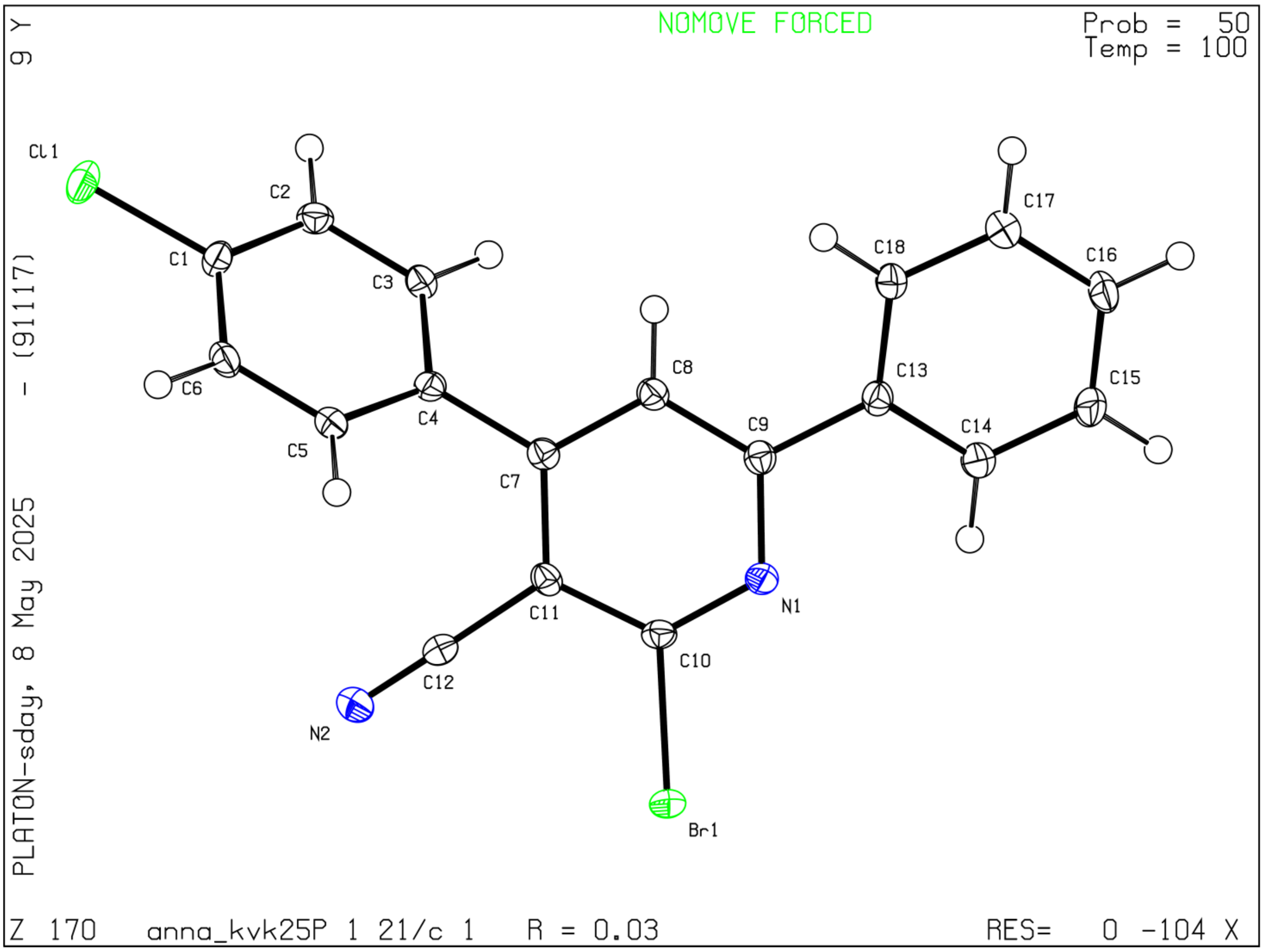

Figure S104. Microphoto images of the single crystal of 2-bromo-4-(4-chlorophenyl)-6-phenylnicotinonitrile 15a

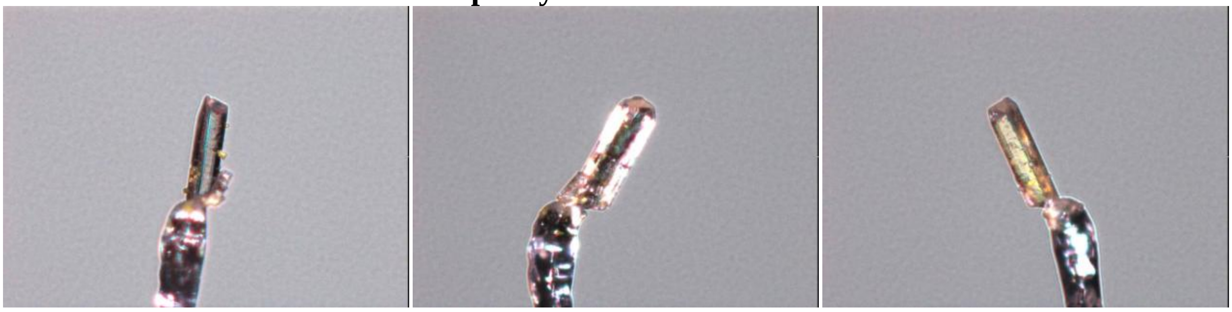

Table S1. Crystal data and structure refinement for 2-bromo-4-(4-chlorophenyl)-6-phenylnicotinonitrile (15a)

Identification code	**15a**
Empirical formula	$C_{18}H_{10}BrClN_2$
Formula weight	369.64
Temperature/K	100.00(11)
Crystal system	monoclinic
Space group	$P2_1/c$
a/Å	15.2876(4)

b/Å	7.21330(10)
c/Å	15.3758(4)
α/°	90
β/°	118.885(3)
γ/°	90
Volume/Å^3	1484.61(7)
Z	4
ρ_{calc}g/cm^3	1.654
μ/mm^{-1}	5.392
F(000)	736.0
Crystal size/mm^3	0.37 × 0.103 × 0.076
Radiation	Cu Kα (λ = 1.54184)
2Θ range for data collection/°	6.604 to 152.484
Index ranges	$-19 \leq h \leq 19$, $-8 \leq k \leq 9$, $-19 \leq l \leq 19$
Reflections collected	10591
Independent reflections	3098 [R_{int} = 0.0382, R_{sigma} = 0.0316]
Data/restraints/parameters	3098/0/199
Goodness-of-fit on F^2	1.100
Final R indexes [I>=2σ (I)]	R_1 = 0.0323, wR_2 = 0.0855
Final R indexes [all data]	R_1 = 0.0332, wR_2 = 0.0865
Largest diff. peak/hole / e Å^{-3}	0.41/-0.97

Table S2. Fractional Atomic Coordinates (×10^4) and Equivalent Isotropic Displacement Parameters (Å^2×10^3) for 2-bromo-4-(4-chlorophenyl)-6-phenylnicotinonitrile (15a). U_{eq} is defined as 1/3 of of the trace of the orthogonalised U_{IJ} tensor.

Atom	*x*	*y*	*z*	U(eq)
Br1	2543.3(2)	3079.7(3)	4029.1(2)	18.68(10)
Cl1	8777.1(4)	4616.0(8)	9976.0(4)	24.84(14)
N1	2363.8(12)	4402(2)	5605.5(12)	14.7(3)
N2	5281.4(14)	2659(3)	5405.1(14)	20.6(4)
C8	3699.6(14)	4991(3)	7253.7(14)	13.7(4)
C12	4743.2(15)	3232(3)	5660.0(15)	15.2(4)
C18	2091.4(15)	6536(3)	7627.4(15)	15.6(4)
C13	1897.0(14)	5391(3)	6817.9(15)	14.5(4)
C11	4077.2(14)	3921(3)	5997.5(14)	13.6(4)
C7	4420.4(14)	4508(3)	6982.5(14)	12.9(4)
C9	2684.3(14)	4927(3)	6559.6(15)	13.8(4)
C10	3040.5(15)	3911(3)	5359.7(14)	14.0(4)
C5	6204.9(15)	5231(3)	7470.7(15)	14.2(4)
C4	5495.5(14)	4575(3)	7721.3(14)	13.1(4)
C3	5815.0(14)	3983(3)	8698.3(14)	14.4(4)
C17	1330.6(17)	7009(3)	7828.0(17)	19.7(4)
C2	6822.0(15)	3994(3)	9399.0(14)	15.9(4)
C1	7513.2(14)	4613(3)	9122.4(15)	16.5(4)
C14	928.4(15)	4694(3)	6233.8(16)	18.6(4)
C6	7213.8(15)	5258(3)	8169.0(15)	15.7(4)
C15	170.4(16)	5172(3)	6445.6(16)	22.7(4)
C16	365.9(16)	6331(3)	7239.0(17)	22.0(4)

Table S3. Anisotropic Displacement Parameters ($Å^2×10^3$) for 2-bromo-4-(4-chlorophenyl)-6-phenylnicotinonitrile (15a). The Anisotropic displacement factor exponent takes the form: $-2\pi^2[h^2a^{*2}U_{11}+2hka^*b^*U_{12}+…]$.

Atom	U_{11}	U_{22}	U_{33}	U_{23}	U_{13}	U_{12}
Br1	16.36(14)	28.64(15)	10.13(14)	-1.44(7)	5.67(10)	-0.08(7)
Cl1	11.5(2)	38.7(3)	19.2(3)	2.0(2)	3.39(19)	0.45(19)
N1	14.0(7)	18.4(8)	12.2(8)	0.9(6)	6.8(6)	-0.8(6)
N2	19.4(8)	27.6(9)	16.4(8)	-0.3(7)	9.9(7)	2.7(7)
C8	14.0(9)	14.6(8)	13.8(9)	-0.1(7)	7.7(7)	-0.1(7)
C12	14.5(9)	17.5(9)	11.3(9)	0.2(7)	4.3(8)	0.2(7)
C18	13.3(9)	17.5(9)	17.0(9)	-0.5(8)	8.1(8)	-1.1(7)
C13	12.6(9)	17.0(9)	14.3(9)	3.1(7)	6.8(7)	0.5(7)
C11	14.5(9)	14.7(8)	14.1(9)	1.6(7)	9.0(7)	0.8(7)
C7	14.2(9)	12.6(8)	12.7(9)	2.0(7)	7.3(7)	0.8(7)
C9	13.3(9)	13.4(8)	15.2(9)	-0.1(7)	7.3(8)	-0.9(7)
C10	15.7(9)	15.7(9)	10.5(8)	0.0(7)	6.3(7)	-0.9(7)
C5	15.2(9)	15.4(8)	14.1(9)	-0.5(7)	8.6(7)	0.2(7)
C4	13.4(9)	12.7(8)	12.7(9)	-1.6(7)	6.0(7)	0.5(7)
C3	14.2(9)	15.8(9)	15.3(9)	0.1(7)	8.9(7)	0.3(7)
C17	17.7(10)	23.9(10)	19.3(10)	-1.1(8)	10.4(9)	0.8(8)
C2	17.7(9)	17.8(9)	12.3(9)	0.5(7)	7.3(8)	1.1(7)
C1	10.6(9)	19.7(9)	15.8(9)	-2.2(7)	3.7(8)	0.7(7)
C14	15.7(9)	24.5(10)	16.9(9)	-2.1(8)	8.9(8)	-3.2(8)
C6	14.7(9)	17.7(9)	18.5(9)	-1.5(7)	11.1(8)	-0.7(7)
C15	12.9(9)	36.9(12)	19.1(10)	-1.3(9)	8.4(8)	-4.8(8)
C16	14.8(9)	33.4(11)	21.9(10)	2.2(9)	12.2(8)	2.4(9)

Table S4. Bond Lengths for 2-bromo-4-(4-chlorophenyl)-6-phenylnicotinonitrile (15a).

Atom	Atom	Length/Å	Atom	Atom	Length/Å
Br1	C10	1.9028(19)	C11	C7	1.407(3)
Cl1	C1	1.7337(19)	C11	C10	1.404(3)
N1	C9	1.357(3)	C7	C4	1.480(3)
N1	C10	1.310(3)	C5	C4	1.397(3)
N2	C12	1.146(3)	C5	C6	1.390(3)
C8	C7	1.398(3)	C4	C3	1.403(3)
C8	C9	1.396(3)	C3	C2	1.389(3)
C12	C11	1.436(3)	C17	C16	1.394(3)
C18	C13	1.401(3)	C2	C1	1.390(3)
C18	C17	1.382(3)	C1	C6	1.388(3)
C13	C9	1.477(3)	C14	C15	1.390(3)
C13	C14	1.402(3)	C15	C16	1.387(3)

Table S5. Bond Angles for 2-bromo-4-(4-chlorophenyl)-6-phenylnicotinonitrile (15a)

Atom	Atom	Atom	Angle/°	Atom	Atom	Atom	Angle/°
C10	N1	C9	117.67(17)	N1	C10	C11	125.40(18)
C9	C8	C7	120.61(18)	C11	C10	Br1	118.84(14)
N2	C12	C11	178.7(2)	C6	C5	C4	120.75(18)
C17	C18	C13	120.24(19)	C5	C4	C7	121.44(17)
C18	C13	C9	121.47(18)	C5	C4	C3	118.89(18)
C18	C13	C14	119.04(19)	C3	C4	C7	119.67(17)
C14	C13	C9	119.48(18)	C2	C3	C4	120.73(18)
C7	C11	C12	122.15(18)	C18	C17	C16	120.6(2)
C10	C11	C12	120.25(18)	C3	C2	C1	119.10(18)
C10	C11	C7	117.47(17)	C2	C1	Cl1	120.19(16)
C8	C7	C11	117.28(18)	C6	C1	Cl1	118.52(16)
C8	C7	C4	120.40(17)	C6	C1	C2	121.29(18)
C11	C7	C4	122.30(17)	C15	C14	C13	120.2(2)
N1	C9	C8	121.53(18)	C1	C6	C5	119.20(18)
N1	C9	C13	116.01(17)	C16	C15	C14	120.4(2)
C8	C9	C13	122.45(18)	C15	C16	C17	119.6(2)
N1	C10	Br1	115.75(14)				

Table S6. Torsion Angles for 2-bromo-4-(4-chlorophenyl)-6-phenylnicotinonitrile (15a)

A	B	C	D	Angle/°	A	B	C	D	Angle/°
Cl1	C1	C6	C5	178.75(15)	C9	N1	C10	C11	-1.5(3)
C8	C7	C4	C5	-139.90(19)	C9	C8	C7	C11	-1.5(3)
C8	C7	C4	C3	39.6(3)	C9	C8	C7	C4	-179.91(17)
C12	C11	C7	C8	-174.26(18)	C9	C13	C14	C15	-177.70(19)
C12	C11	C7	C4	4.1(3)	C10	N1	C9	C8	1.6(3)
C12	C11	C10	Br1	-2.8(2)	C10	N1	C9	C13	-177.44(17)
C12	C11	C10	N1	175.80(19)	C10	C11	C7	C8	1.6(3)
C18	C13	C9	N1	-157.30(18)	C10	C11	C7	C4	179.97(17)
C18	C13	C9	C8	23.7(3)	C5	C4	C3	C2	-1.9(3)
C18	C13	C14	C15	1.4(3)	C4	C5	C6	C1	0.4(3)
C18	C17	C16	C15	0.0(3)	C4	C3	C2	C1	0.4(3)
C13	C18	C17	C16	1.0(3)	C3	C2	C1	Cl1	-179.16(15)
C13	C14	C15	C16	-0.4(3)	C3	C2	C1	C6	1.5(3)
C11	C7	C4	C5	41.7(3)	C17	C18	C13	C9	177.37(19)
C11	C7	C4	C3	-138.71(19)	C17	C18	C13	C14	-1.8(3)
C7	C8	C9	N1	-0.1(3)	C2	C1	C6	C5	-1.9(3)
C7	C8	C9	C13	178.85(18)	C14	C13	C9	N1	21.8(3)
C7	C11	C10	Br1	-178.75(14)	C14	C13	C9	C8	-157.18(19)
C7	C11	C10	N1	-0.1(3)	C14	C15	C16	C17	-0.3(3)
C7	C4	C3	C2	178.56(18)	C6	C5	C4	C7	-178.98(18)
C9	N1	C10	Br1	177.20(14)	C6	C5	C4	C3	1.5(3)

Table S7. Hydrogen Atom Coordinates (Å×10[4]) and Isotropic Displacement Parameters (Å[2]×10[3]) for 2-bromo-4-(4-chlorophenyl)-6-phenylnicotinonitrile (15a).

Atom	*x*	*y*	*z*	U(eq)
H8	3902.31	5365.97	7916.44	16
H18	2748.44	6988.95	8040.11	19
H5	5995.37	5662.93	6815.66	17
H3	5337.17	3569.25	8882.37	17
H17	1466.21	7802.93	8371.84	24
H2	7035.48	3584.2	10058.47	19
H14	790.02	3893.52	5691.88	22
H6	7692.6	5712.39	7995.84	19
H15	-485.16	4702.13	6044.7	27
H16	-154.25	6661.22	7380.04	26

Experimental

Single crystals of $C_{18}H_{10}BrClN_2$ (2-bromo-4-(4-chlorophenyl)-6-phenylnicotinonitrile (**15a**) were isolated form mother liquor (AcOH solution). A suitable crystal was selected and studied on a SuperNova, Dual, Cu at home/near, AtlasS2 diffractometer. The crystal was kept at 100.00(11) K during data collection. Using Olex2 [1], the structure was solved with the SHELXT [2] structure solution program using Intrinsic Phasing and refined with the SHELXL [3] refinement package using Least Squares minimisation.

1. Dolomanov, O.V., Bourhis, L.J., Gildea, R.J, Howard, J.A.K. & Puschmann, H. (2009), J. Appl. Cryst. 42, 339-341.
2. Sheldrick, G.M. (2015). Acta Cryst. A71, 3-8.
3. Sheldrick, G.M. (2015). Acta Cryst. C71, 3-8.

Crystal structure determination of 2-bromo-4-(4-chlorophenyl)-6-phenylnicotinonitrile (15a)

Crystal Data for $C_{18}H_{10}BrClN_2$ (M =369.64 g/mol): monoclinic, space group $P2_1/c$ (no. 14), a = 15.2876(4) Å, b = 7.21330(10) Å, c = 15.3758(4) Å, β = 118.885(3)°, V = 1484.61(7) Å^3, Z = 4, T = 100.00(11) K, μ(Cu Kα) = 5.392 mm^{-1}, $Dcalc$ = 1.654 g/cm^3, 10591 reflections measured (6.604° ≤ 2Θ ≤ 152.484°), 3098 unique (R_{int} = 0.0382, R_{sigma} = 0.0316) which were used in all calculations. The final R_1 was 0.0323 (I > 2σ(I)) and wR_2 was 0.0865 (all data).

Figure S105. ORTEP drawings of X-ray structures for co-crystallized (*2S*,3R*,4S*,6R)-3-benzoyl-5-bromo-4-hydroxy-4-phenyl-2,6-di-*p*-tolylcyclohexane-1,1-dicarbonitrile 17-Br (upper image) and (*2S*,3R*,4S*,6R**)-3-benzoyl-4-hydroxy-4-phenyl-2,6-di-*p*-tolylcyclohexane-1,1-dicarbonitrile 17 (bottom image), solvate with AcOH. Ellipsoids are given with 50% probability (CCDC deposition number 2499676).**

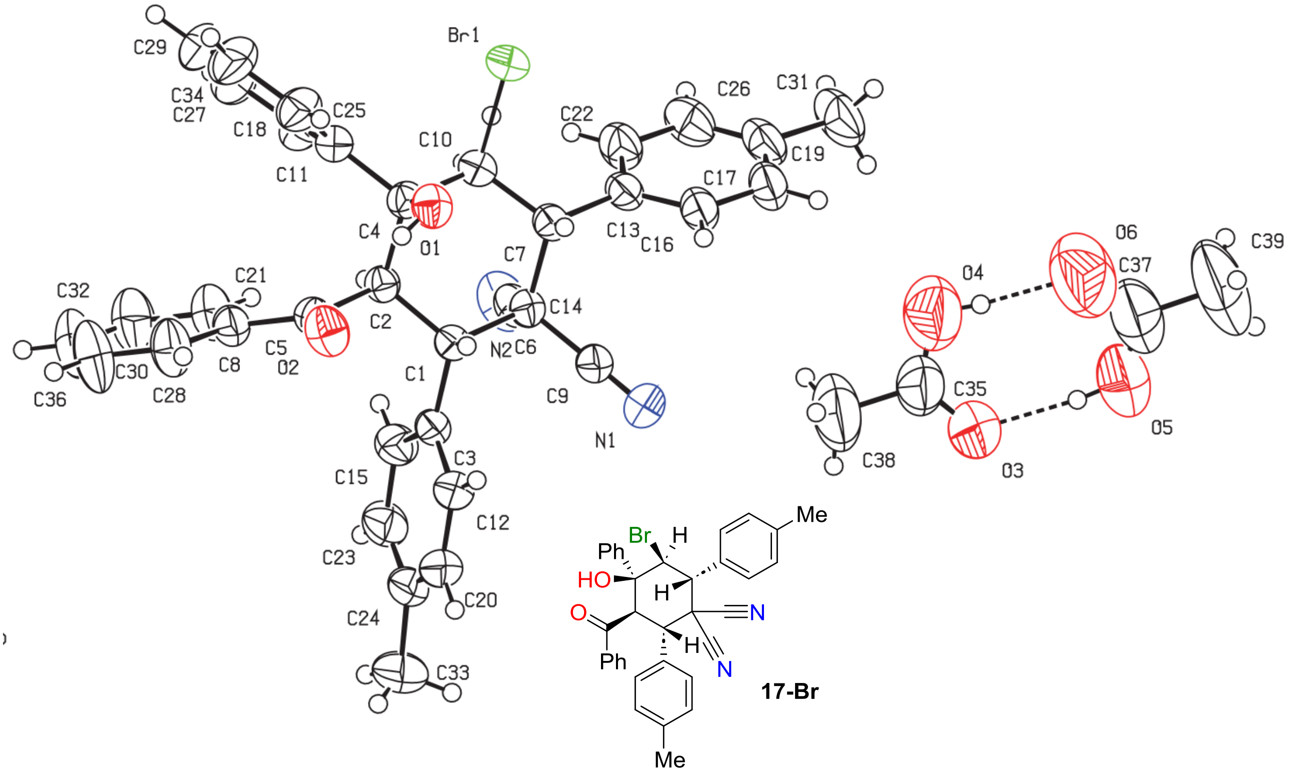

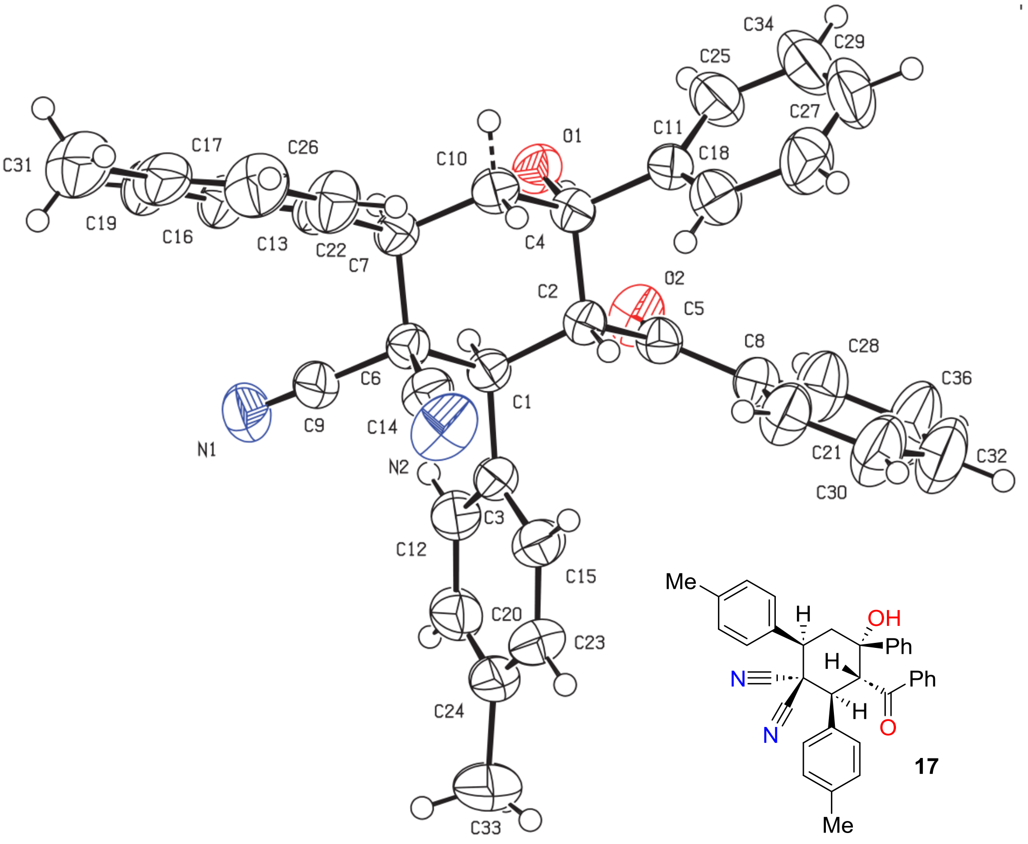

Figure S106. Microphoto images of the single crystal of 17 + 17-Br co-crystals

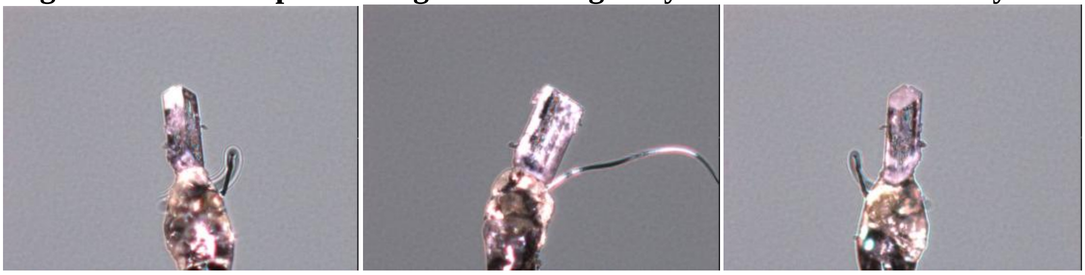

Table S8. Crystal data and structure refinement for co-crystals of cyclohexanols 17 + 17-Br

Identification code	**17 + 17-Br**
Empirical formula	$C_{39}H_{37.39}Br_{0.61}N_2O_6$
Formula weight	678.84
Temperature/K	293(2)
Crystal system	triclinic
Space group	P-1
a/Å	11.7101(2)
b/Å	12.1777(2)
c/Å	13.1548(3)
α/°	105.5935(16)
β/°	95.2886(16)
γ/°	91.2975(15)
Volume/Å^3	1796.97(6)
Z	2
ρ_{calc}g/cm^3	1.255
μ/mm^{-1}	1.436
F(000)	709.0
Crystal size/mm^3	0.31 × 0.14 × 0.11
Radiation	Cu Kα (λ = 1.54184)
2Θ range for data collection/°	7.014 to 152.508
Index ranges	$-14 \leq h \leq 14$, $-15 \leq k \leq 13$, $-16 \leq l \leq 16$
Reflections collected	26097
Independent reflections	7469 [R_{int} = 0.0156, R_{sigma} = 0.0143]
Data/restraints/parameters	7469/1/444
Goodness-of-fit on F^2	1.144
Final R indexes [I>=2σ (I)]	R_1 = 0.0527, wR_2 = 0.1359
Final R indexes [all data]	R_1 = 0.0538, wR_2 = 0.1366
Largest diff. peak/hole / e Å^{-3}	0.64/-0.42

Table S9. Fractional Atomic Coordinates (×10^4) and Equivalent Isotropic Displacement Parameters (Å^2×10^3) for co-crystals of cyclohexanols 17 + 17-Br. U_{eq} is defined as 1/3 of of the trace of the orthogonalised U_{IJ} tensor.

Atom	*x*	*y*	*z*	U(eq)
Br1	7557.5(3)	3969.5(4)	7329.2(3)	61.32(15)
O1	6642.1(13)	4337.8(11)	5210.1(12)	49.2(3)
O2	5951.0(16)	3601.4(13)	3085.8(13)	59.2(4)
O3	-387(3)	5881(4)	9045(2)	151.4(15)
O4	1436(3)	6329(4)	9522(3)	150.5(14)

O5	-880(4)	6988(6)	10953(4)	192(2)
O6	841(5)	7626(4)	11320(4)	187(2)
N1	2332.0(19)	3783(2)	5913.0(18)	73.4(6)
N2	4103(3)	690.3(19)	5692.0(19)	77.2(7)
C1	4551.0(15)	2868.7(15)	4466.6(14)	37.3(4)
C2	5758.7(16)	2459.1(15)	4258.2(14)	37.7(4)
C3	3610.2(16)	2276.2(17)	3609.5(14)	41.5(4)
C4	6685.2(16)	3158.3(16)	5142.3(15)	41.1(4)
C5	6035.1(17)	2642.5(17)	3192.2(15)	42.3(4)
C6	4275.1(16)	2802.2(16)	5599.2(15)	40.0(4)
C7	5218.4(16)	3469.9(17)	6502.4(14)	41.0(4)
C8	6438.8(18)	1714.7(19)	2342.2(16)	48.9(5)
C9	3163.8(18)	3330.2(19)	5788.4(16)	49.0(5)
C10	6396.1(17)	3040.2(18)	6221.7(15)	45.4(4)
C11	7868.2(17)	2710.2(19)	4899.7(16)	47.9(5)
C12	2838.4(18)	2939(2)	3202.1(17)	49.9(5)
C13	4919.1(18)	3418.8(18)	7589.7(15)	46.6(4)
C14	4159(2)	1603.6(18)	5645.1(16)	50.0(5)
C15	3466(2)	1094.7(19)	3224.1(18)	54.4(5)
C16	4228(2)	4232(2)	8143.1(17)	54.9(5)
C17	4240(2)	3326(3)	9564.5(18)	68.3(7)
C18	8079(2)	1577(2)	4825(2)	62.7(6)
C19	3882(2)	4176(2)	9112.0(19)	66.2(7)
C20	1934(2)	2436(2)	2461.4(19)	61.3(6)
C21	6521(3)	605(2)	2397(2)	66.3(7)
C22	5288(2)	2577(2)	8053.7(19)	63.7(6)
C23	2541(2)	607(2)	2488(2)	65.8(7)
C24	1755(2)	1271(3)	2110.5(18)	64.6(7)
C25	8729(2)	3402(3)	4711(2)	65.1(6)
C26	4956(3)	2542(3)	9030(2)	74.0(7)
C27	9137(3)	1153(3)	4553(2)	81.3(9)
C28	6776(3)	1992(3)	1450(2)	74.1(8)
C29	9985(2)	1863(4)	4377(3)	89.9(11)
C30	6924(3)	-220(3)	1577(3)	87.4(10)
C31	3846(3)	3262(3)	10611(2)	94.4(11)
C32	7241(4)	65(3)	701(3)	98.0(11)
C33	712(3)	736(4)	1361(3)	98.9(12)
C34	9782(2)	2975(4)	4460(3)	86.5(10)
C35	605(4)	5887(4)	8827(3)	99.9(11)
C36	7170(4)	1167(3)	637(3)	104.1(12)
C37	-128(7)	7541(5)	11579(4)	134(2)
C38	904(5)	5331(5)	7750(4)	149(2)
C39	-405(7)	8134(6)	12666(4)	201(3)

Table S10. Anisotropic Displacement Parameters (Å^2×10^3) for co-crystals of cyclohexanols 17 + 17-Br. The Anisotropic displacement factor exponent takes the form: -2π^2[h^{2}a*^{2}U$_{11}$+2hka*b*U$_{12}$+…].

Atom	U_{11}	U_{22}	U_{33}	U_{23}	U_{13}	U_{12}
Br1	47.3(2)	83.5(3)	43.4(2)	4.49(18)	-6.44(15)	0.64(18)
O1	55.2(8)	39.0(7)	51.3(8)	7.8(6)	8.6(7)	-1.7(6)
O2	80.7(11)	51.7(9)	52.5(9)	24.1(7)	13.9(8)	6.5(8)
O3	108(2)	253(4)	80.0(18)	20(2)	13.4(16)	50(3)
O4	131(3)	175(4)	129(3)	5(3)	45(2)	-18(2)
O5	148(4)	303(6)	94(3)	-9(3)	38(3)	16(4)
O6	203(5)	169(4)	146(4)	-33(3)	37(3)	-22(3)
N1	50.4(12)	100.1(17)	62.3(13)	7.1(12)	9.3(9)	17.1(11)
N2	115(2)	54.6(12)	66.9(14)	23.7(10)	16.7(13)	-7.0(12)
C1	39.0(9)	37.0(8)	35.8(9)	10.0(7)	3.2(7)	1.5(7)
C2	40.3(9)	36.9(9)	36.6(9)	10.8(7)	5.1(7)	2.7(7)
C3	40.0(9)	48.7(10)	34.2(9)	8.9(8)	3.6(7)	-0.2(8)
C4	39.0(9)	40.7(9)	42.6(10)	9.8(8)	4.3(7)	1.1(7)
C5	42.6(10)	44.5(10)	41.2(10)	14.0(8)	5.4(8)	-0.2(8)
C6	41.6(9)	40.8(9)	37.9(9)	10.5(7)	6.5(7)	1.9(7)
C7	43.0(10)	43.8(10)	35.2(9)	9.3(7)	3.9(7)	1.0(8)
C8	48.4(11)	55.9(12)	40.9(10)	9.6(9)	8.4(8)	0.4(9)
C9	42.1(11)	61.8(12)	40.4(10)	8.9(9)	5.8(8)	0.3(9)
C10	43.6(10)	52.5(11)	38.7(10)	11.2(8)	0.9(8)	3.3(8)
C11	39.7(10)	58.6(12)	41.1(10)	6.8(9)	2.3(8)	3.8(9)
C12	47.8(11)	57.2(12)	44.4(11)	13.8(9)	2.1(8)	5.2(9)
C13	48.6(11)	54.0(11)	35.5(9)	10.4(8)	2.6(8)	-1.4(9)
C14	60.8(13)	49.0(11)	41.1(10)	13.7(8)	7.3(9)	-5.1(9)
C15	55.9(12)	49.6(11)	51.3(12)	4.9(9)	-0.4(10)	-0.4(9)
C16	61.6(13)	59.7(13)	40.8(11)	8.0(9)	9.5(9)	2.7(10)
C17	75.8(17)	88.5(18)	36.9(11)	12.1(11)	6.5(11)	-18.4(14)
C18	50.7(13)	66.0(14)	68.0(15)	11.8(12)	4.5(11)	15.0(11)
C19	68.4(15)	81.0(17)	43.7(12)	5.2(11)	14.7(11)	-1.6(13)
C20	46.6(12)	86.5(17)	49.2(12)	17.0(12)	-0.9(9)	9.2(11)
C21	85.0(18)	52.5(13)	60.5(14)	7.9(11)	25.1(13)	1.4(12)
C22	76.6(16)	73.2(15)	45.9(12)	22.5(11)	8.8(11)	14.2(13)
C23	63.2(15)	61.7(14)	58.3(14)	-5.4(11)	1.3(11)	-12.1(11)
C24	46.7(12)	92.4(19)	43.0(11)	0.3(12)	1.4(9)	-7.6(12)
C25	45.3(12)	84.2(17)	66.6(15)	21.4(13)	8.6(11)	-2.4(11)
C26	91(2)	87.8(19)	50.0(13)	32.7(13)	3.2(13)	3.7(16)
C27	70.5(18)	94(2)	72.4(18)	7.9(15)	3.7(14)	39.2(16)
C28	94(2)	77.9(17)	57.4(15)	22.3(13)	31.2(14)	10.7(15)
C29	49.3(15)	146(3)	74.5(19)	25(2)	17.4(13)	33.9(18)
C30	115(3)	60.5(16)	79(2)	-2.5(14)	36.2(19)	4.6(16)
C31	112(3)	127(3)	44.6(14)	23.4(16)	16.8(15)	-21(2)
C32	124(3)	86(2)	74(2)	-8.0(17)	46(2)	7(2)
C33	63.1(18)	136(3)	73(2)	-4.1(19)	-16.9(15)	-14.9(18)
C34	45.1(14)	134(3)	85(2)	36(2)	15.9(13)	4.4(16)

Table S10. Anisotropic Displacement Parameters ($Å^2×10^3$) for co-crystals of cyclohexanols 17 + 17-Br. The Anisotropic displacement factor exponent takes the form: -$2\pi^2[h^2a^{*2}U_{11}+2hka^*b^*U_{12}+…]$.

Atom	U_{11}	U_{22}	U_{33}	U_{23}	U_{13}	U_{12}
C35	102(3)	126(3)	78(2)	31(2)	28(2)	31(2)
C36	145(3)	108(3)	63.2(18)	16.5(18)	55(2)	10(2)
C37	188(6)	131(4)	83(3)	18(3)	33(4)	51(4)
C38	172(5)	184(5)	91(3)	23(3)	57(3)	46(4)
C39	321(10)	181(6)	91(3)	0(4)	60(5)	77(6)

Table S11. Bond Lengths for co-crystals of cyclohexanols 17 + 17-Br.

Atom	Atom	Length/Å	Atom	Atom	Length/Å
Br1	C10	1.983(2)	C8	C28	1.393(3)
O1	C4	1.417(2)	C11	C18	1.386(3)
O2	C5	1.218(2)	C11	C25	1.381(3)
O3	C35	1.222(5)	C12	C20	1.380(3)
O4	C35	1.273(5)	C13	C16	1.387(3)
O5	C37	1.203(8)	C13	C22	1.384(3)
O6	C37	1.224(7)	C15	C23	1.389(3)
N1	C9	1.134(3)	C16	C19	1.391(3)
N2	C14	1.131(3)	C17	C19	1.381(4)
C1	C2	1.534(2)	C17	C26	1.375(4)
C1	C3	1.518(3)	C17	C31	1.512(3)
C1	C6	1.576(2)	C18	C27	1.393(4)
C2	C4	1.564(3)	C20	C24	1.373(4)
C2	C5	1.539(3)	C21	C30	1.389(4)
C3	C12	1.390(3)	C22	C26	1.387(3)
C3	C15	1.392(3)	C23	C24	1.384(4)
C4	C10	1.532(3)	C24	C33	1.512(3)
C4	C11	1.530(3)	C25	C34	1.382(4)
C5	C8	1.481(3)	C27	C29	1.378(5)
C6	C7	1.578(3)	C28	C36	1.379(4)
C6	C9	1.477(3)	C29	C34	1.358(5)
C6	C14	1.481(3)	C30	C32	1.369(5)
C7	C10	1.528(3)	C32	C36	1.371(5)
C7	C13	1.521(3)	C35	C38	1.475(5)
C8	C21	1.378(3)	C37	C39	1.488(6)

Table S12. Bond Angles for co-crystals of cyclohexanols 17 + 17-Br.

Atom	Atom	Atom	Angle/°	Atom	Atom	Atom	Angle/°
C2	C1	C6	110.19(14)	C25	C11	C18	118.6(2)
C3	C1	C2	114.44(15)	C20	C12	C3	120.8(2)
C3	C1	C6	111.86(15)	C16	C13	C7	119.29(19)
C1	C2	C4	111.26(14)	C22	C13	C7	122.9(2)
C1	C2	C5	108.43(15)	C22	C13	C16	117.7(2)
C5	C2	C4	108.01(15)	N2	C14	C6	177.9(3)
C12	C3	C1	118.77(18)	C23	C15	C3	120.1(2)
C12	C3	C15	118.08(19)	C13	C16	C19	121.0(2)
C15	C3	C1	123.13(18)	C19	C17	C31	120.6(3)
O1	C4	C2	110.17(15)	C26	C17	C19	117.9(2)
O1	C4	C10	105.69(15)	C26	C17	C31	121.6(3)
O1	C4	C11	111.77(16)	C11	C18	C27	120.1(3)
C10	C4	C2	109.14(15)	C17	C19	C16	121.0(2)
C11	C4	C2	109.04(15)	C24	C20	C12	121.6(2)
C11	C4	C10	110.96(16)	C8	C21	C30	120.7(3)
O2	C5	C2	117.20(17)	C13	C22	C26	120.8(2)
O2	C5	C8	120.68(18)	C24	C23	C15	121.5(2)
C8	C5	C2	122.07(17)	C20	C24	C23	117.9(2)
C1	C6	C7	112.19(15)	C20	C24	C33	120.9(3)
C9	C6	C1	106.87(16)	C23	C24	C33	121.2(3)
C9	C6	C7	108.87(16)	C11	C25	C34	120.7(3)
C9	C6	C14	108.38(17)	C17	C26	C22	121.6(3)
C14	C6	C1	111.30(15)	C29	C27	C18	120.2(3)
C14	C6	C7	109.11(16)	C36	C28	C8	120.4(3)
C10	C7	C6	108.90(15)	C34	C29	C27	119.7(3)
C13	C7	C6	111.25(16)	C32	C30	C21	120.0(3)
C13	C7	C10	114.95(16)	C30	C32	C36	120.0(3)
C21	C8	C5	123.7(2)	C29	C34	C25	120.7(3)
C21	C8	C28	118.6(2)	O3	C35	O4	122.1(4)
C28	C8	C5	117.7(2)	O3	C35	C38	121.3(5)
N1	C9	C6	176.2(3)	O4	C35	C38	116.5(4)
C4	C10	Br1	108.23(13)	C32	C36	C28	120.3(3)
C7	C10	Br1	107.37(13)	O5	C37	O6	120.6(5)
C7	C10	C4	113.19(16)	O5	C37	C39	118.6(7)
C18	C11	C4	120.0(2)	O6	C37	C39	120.8(8)
C25	C11	C4	121.4(2)				

Table S13. Torsion Angles for co-crystals of cyclohexanols 17 + 17-Br.

A	B	C	D	Angle/°	A	B	C	D	Angle/°
O1	C4	C10	Br1	-59.93(17)	C6	C1	C3	C15	-75.6(2)
O1	C4	C10	C7	59.0(2)	C6	C7	C10	Br1	176.48(12)
O1	C4	C11	C18	178.69(19)	C6	C7	C10	C4	57.1(2)
O1	C4	C11	C25	-3.8(3)	C6	C7	C13	C16	-87.3(2)
O2	C5	C8	C21	-178.0(2)	C6	C7	C13	C22	91.4(3)
O2	C5	C8	C28	3.5(3)	C7	C13	C16	C19	176.3(2)
C1	C2	C4	O1	-57.36(19)	C7	C13	C22	C26	-177.5(2)
C1	C2	C4	C10	58.26(19)	C8	C21	C30	C32	-0.1(5)
C1	C2	C4	C11	179.62(15)	C8	C28	C36	C32	-0.5(6)
C1	C2	C5	O2	54.8(2)	C9	C6	C7	C10	-171.80(16)
C1	C2	C5	C8	-127.95(19)	C9	C6	C7	C13	60.5(2)
C1	C3	C12	C20	-176.65(19)	C10	C4	C11	C18	61.0(3)
C1	C3	C15	C23	175.8(2)	C10	C4	C11	C25	-121.5(2)
C1	C6	C7	C10	-53.7(2)	C10	C7	C13	C16	148.3(2)
C1	C6	C7	C13	178.58(15)	C10	C7	C13	C22	-33.0(3)
C2	C1	C3	C12	-130.74(19)	C11	C4	C10	Br1	61.42(18)
C2	C1	C3	C15	50.7(3)	C11	C4	C10	C7	-179.69(16)
C2	C1	C6	C7	54.39(19)	C11	C18	C27	C29	1.3(4)
C2	C1	C6	C9	173.65(16)	C11	C25	C34	C29	1.1(5)
C2	C1	C6	C14	-68.2(2)	C12	C3	C15	C23	-2.8(3)
C2	C4	C10	Br1	-178.40(12)	C12	C20	C24	C23	-2.8(4)
C2	C4	C10	C7	-59.5(2)	C12	C20	C24	C33	175.4(3)
C2	C4	C11	C18	-59.3(2)	C13	C7	C10	Br1	-57.95(19)
C2	C4	C11	C25	118.2(2)	C13	C7	C10	C4	-177.33(16)
C2	C5	C8	C21	4.9(3)	C13	C16	C19	C17	1.7(4)
C2	C5	C8	C28	-173.7(2)	C13	C22	C26	C17	0.9(5)
C3	C1	C2	C4	176.72(15)	C14	C6	C7	C10	70.1(2)
C3	C1	C2	C5	58.1(2)	C14	C6	C7	C13	-57.6(2)
C3	C1	C6	C7	-177.10(15)	C15	C3	C12	C20	2.0(3)
C3	C1	C6	C9	-57.8(2)	C15	C23	C24	C20	1.9(4)
C3	C1	C6	C14	60.3(2)	C15	C23	C24	C33	-176.2(3)
C3	C12	C20	C24	0.8(4)	C16	C13	C22	C26	1.2(4)
C3	C15	C23	C24	0.9(4)	C18	C11	C25	C34	-0.4(4)
C4	C2	C5	O2	-65.9(2)	C18	C27	C29	C34	-0.7(5)
C4	C2	C5	C8	111.4(2)	C19	C17	C26	C22	-1.8(4)
C4	C11	C18	C27	176.8(2)	C21	C8	C28	C36	0.8(5)
C4	C11	C25	C34	-178.0(2)	C21	C30	C32	C36	0.4(6)
C5	C2	C4	O1	61.53(19)	C22	C13	C16	C19	-2.5(4)
C5	C2	C4	C10	177.15(15)	C25	C11	C18	C27	-0.7(4)
C5	C2	C4	C11	-61.5(2)	C26	C17	C19	C16	0.5(4)
C5	C8	C21	C30	-179.0(3)	C27	C29	C34	C25	-0.5(5)
C5	C8	C28	C36	179.4(3)	C28	C8	C21	C30	-0.5(4)
C6	C1	C2	C4	-56.19(19)	C30	C32	C36	C28	-0.1(7)
C6	C1	C2	C5	-174.83(14)	C31	C17	C19	C16	-179.2(3)
C6	C1	C3	C12	103.0(2)	C31	C17	C26	C22	177.9(3)

Table S14. Hydrogen Atom Coordinates (Å×10^4) and Isotropic Displacement Parameters (Å^2×10^3) for co-crystals of cyclohexanols 17 + 17-Br.

Atom	*x*	*y*	*z*	U(eq)
H1	6582.72	4425.41	4611.19	74
H4	1185.13	6638.54	10085.04	226
H1A	4566.96	3678.49	4483.78	45
H2	5783.92	1646.51	4225.16	45
H7	5219.89	4273.38	6497.96	49
H10A	6418.51	2243.26	6217.59	54
H10B	6978.34	3464.93	6766.63	54
H12	2932.17	3730.43	3431.21	60
H15	3988.82	630.92	3459.43	65
H16	3992.85	4823.63	7862.14	66
H18	7514.22	1099.8	4957.46	75
H19	3401.63	4719.48	9460.41	79
H20	1434.63	2896.7	2193.58	74
H21	6305.09	406.28	2989.64	80
H22	5763.87	2029.31	7706.56	76
H23	2448.8	-184.2	2244.52	79
H25	8599.05	4162.59	4753.02	78
H26	5223.48	1975.35	9330.31	89
H27	9270.88	389.21	4489.77	98
H28	6735.34	2739.19	1402.4	89
H29	10694.16	1581.77	4202.49	108
H30	6977.44	-966.37	1622.21	105
H31A	4042.53	3973.49	11137.09	142
H31B	4215.78	2656.62	10830.4	142
H31C	3028.58	3117.1	10529.02	142
H32	7505.74	-488.85	150	118
H33B	618.48	1095.97	795.41	148
H33A	42.4	835.26	1740.22	148
H33C	812.52	-64.27	1072.85	148
H34	10356.69	3455.53	4347.31	104
H36	7387.67	1358.54	41.48	125
H38A	1209.79	5896.18	7447.63	223
H38B	228.55	4955.91	7314.54	223
H38C	1468.15	4779.66	7786.04	223
H39C	-1195.47	7964.7	12731.85	302
H39B	-277.76	8943	12790.33	302
H39A	77.72	7877.42	13177.68	302
H5	-760(90)	6700(80)	10320(30)	320(50)

Table S15. Atomic Occupancy for co-crystals of cyclohexanols 17 + 17-Br.

Atom	*Occupancy*	Atom	*Occupancy*	Atom	*Occupancy*
Br1	0.61	H10B	0.39		

Experimental

Single crystals of $C_{39}H_{37.39}Br_{0.61}N_2O_6$ **17 + 17-Br** were crystallized by slow evaporation of AcOH solution. A suitable crystal was selected and studied on a SuperNova, Dual, Cu at home/near, AtlasS2 diffractometer. The crystal was kept at 293(2) K during data collection. Using Olex2 [1], the structure was solved with the SHELXT [2] structure solution program using Intrinsic Phasing and refined with the SHELXL [3] refinement package using Least Squares minimisation.

1. Dolomanov, O.V., Bourhis, L.J., Gildea, R.J, Howard, J.A.K. & Puschmann, H. (2009), J. Appl. Cryst. 42, 339-341.
2. Sheldrick, G.M. (2015). Acta Cryst. A71, 3-8.
3. Sheldrick, G.M. (2015). Acta Cryst. C71, 3-8.

Crystal structure determination of co-crystals of cyclohexanols 17 + 17-Br

Crystal Data for $C_{39}H_{37.39}Br_{0.61}N_2O_6$ (M =678.84 g/mol): triclinic, space group P-1 (no. 2), a = 11.7101(2) Å, b = 12.1777(2) Å, c = 13.1548(3) Å, α = 105.5935(16)°, β = 95.2886(16)°, γ = 91.2975(15)°, V = 1796.97(6) Å^3, Z = 2, T = 293(2) K, μ(Cu Kα) = 1.436 mm^{-1}, $Dcalc$ = 1.255 g/cm^3, 26097 reflections measured (7.014° ≤ 2Θ ≤ 152.508°), 7469 unique (R_{int} = 0.0156, R_{sigma} = 0.0143) which were used in all calculations. The final R_1 was 0.0527 (I > 2σ(I)) and wR_2 was 0.1366 (all data).

Refinement model description

Number of restraints - 1, number of constraints - unknown.
Details:

```
1. Fixed Uiso
 At 1.2 times of:
  All C(H) groups, All C(H,H) groups
 At 1.5 times of:
  All C(H,H,H) groups, All O(H) groups
2. Restrained distances
 H5-O5
 0.82 with sigma of 0.02
3. Others
 Fixed Sof: Br1(0.61) H10B(0.39)
4.a Ternary CH refined with riding coordinates:
 C1(H1A), C2(H2), C7(H7)
4.b Secondary CH2 refined with riding coordinates:
 C10(H10A,H10B)
4.c Aromatic/amide H refined with riding coordinates:
 C12(H12), C15(H15), C16(H16), C18(H18), C19(H19), C20(H20), C21(H21),
 C22(H22), C23(H23), C25(H25), C26(H26), C27(H27), C28(H28), C29(H29), C30(H30),
  C32(H32), C34(H34), C36(H36)
4.d Idealised Me refined as rotating group:
 C31(H31A,H31B,H31C), C33(H33B,H33A,H33C), C38(H38A,H38B,H38C), C39(H39C,H39B,
 H39A)
4.e Idealised tetrahedral OH refined as rotating group:
 O1(H1), O4(H4)
```
